# Nucleophagy removes cytotoxic trapped PARP1

**DOI:** 10.1038/s41556-026-01961-5

**Published:** 2026-06-02

**Authors:** Gwendoline Hoslett, Sara Tribble, Pauline Lascaux, Stelios Koukouravas, Ignacio Torrecilla, Wei Song, Cynthia X. Hou, Junyi Li, Martín González-Fernández, Giuliana De Gregoriis, Rebecca A. Dagg, Darragh O’Brien, Andrea Pierangelini, Thibaud Martial, Alvin Wei Tian Ng, Nuno Raimundo, Ira Milosevic, Raimundo Freire, Yuliang Li, Sven Rottenberg, Dragomir B. Krastev, Christopher J. Lord, Madalena Tarsounas, Kristijan Ramadan

**Affiliations:** 1 The MRC Weatherall Institute of Molecular Medicine, Department of Oncology, John Radcliffe Hospital, University of Oxford, Oxford, UK; 2 Institute of Animal Pathology, Vetsuisse Faculty, University of Bern, Bern, Switzerland; 3 Bern Center for Precision Medicine and Cancer Therapy Research Cluster, Department for Biomedical Research, University of Bern, Bern, Switzerland; 4 Department of Oncology, University of Oxford, Oxford, UK; 5 Centre for Medicines Discovery, Nuffield Department of Medicine, University of Oxford, Oxford, UK; 6 Centre for Human Genetics, Nuffield Department of Medicine, University of Oxford, Oxford, UK; 7 Population and Global Health Programme, Lee Kong Chian School of Medicine (LKCMedicine), Nanyang Technological University, Singapore, Singapore; 8 Department of Cell and Molecular Systems, Penn State College of Medicine, and Penn State Cancer Institute, Hershey, PA USA; 9 Multidisciplinary Institute of Aging, Center for Innovative Biomedicine and Biotechnology, University of Coimbra, Coimbra, Portugal; 10 Unidad de Investigación, Hospital Universitario de Canarias, Instituto de Investigación Sanitaria de Canarias (IISC)/FIISC, La Laguna, Spain; 11 Instituto de Tecnologías Biomédicas, Campus Ciencias de la Salud, Universidad de La Laguna, La Laguna, Spain; 12 Universidad Fernando Pessoa Canarias, Santa Maria de Guia, Spain; 13 Department of Interventional Medicine and Minimally Invasive Oncology, The Second Qilu Hospital of Shandong University, Jinan, P. R. China; 14 Precision Oncology Laboratory, The Breast Cancer Now Toby Robins Research Centre, The Institute of Cancer Research, London, UK; 15 Cancer Discovery and Regenerative Medicine Programme, LKCMedicine, Nanyang Technological University, Singapore, Singapore

**Keywords:** Cancer therapeutic resistance, Macroautophagy, DNA damage response, Breast cancer

## Abstract

Poly(ADP-ribose) polymerase (PARP) inhibitors (PARPi) induce cytotoxicity in homologous recombination repair (HR)-deficient (HRD) cancers by trapping PARP1 on chromatin, thereby causing irreparable replication-associated DNA damage. Although increased clearance of trapped PARP1 from chromatin reduces the sensitivity of cancer cells to PARPi, details surrounding this process remain unclear. PARPi exposure is known to cause increased autophagy flux, whereas autophagy inhibition can hypersensitize cells to PARPi. Our study reveals that trapped PARP1 is cleared via nucleophagy, with the selective autophagy receptor TEX264 and its partner segregase p97 (also known as VCP) orchestrating this process. TEX264 interacts directly with trapped PARP1, linking it to the autophagosomal protein LC3 for degradation. Disrupting this pathway, either chemically or genetically, increases PARP1 trapping, resulting in protein aggregates, DNA damage and cell lethality, ultimately re-sensitizing PARPi-resistant cells. We conclude that nucleophagy serves a cytoprotective role by targeting PARPi-induced trapped PARP1 for degradation.

## Abstract (web summary)

Hoslett et al. show that trapped PARP1 is cleared from the nucleus by nucleophagy involving the autophagy receptor TEX264 and p97. Inactivation of nucleophagy resensitizes acquired resistance of BRCA1-deficient cells to PARP inhibitors.

## Main

In 20 years of clinical trials^1^, six PARP inhibitors (PARPi) have been approved for the treatment of homologous recombination repair (HR)-deficient (HRD) breast, prostate, pancreatic and ovarian cancers^2–4^. This represents the first synthetic lethality-targeted therapy, whereby HRD- cells, most commonly those with mutations in BRCA1 or BRCA2, display up to 1,000-fold more selectivity to PARPi than their wild-type (WT) counterparts^5–7^. PARPi target the key DNA damage repair (DDR) enzyme PARP1, involved in single-strand break (SSB) response^8^, replication fork stability^9^ and ligation of Okazaki fragments^10^.

All clinically approved PARPi are nicotinamide analogues that bind the catalytic site of PARP1 (ref. ^7^). Synthetic lethality was first attributed to catalytic inhibition of PARP1, leading to accumulation of SSBs that collapse into irreparable double-strand breaks (DSBs) during S phase in HRD-cancers^5–7^. However, the discovery that cytotoxicity of different PARPi does not directly correlate with catalytic inhibitory potency, and that loss of PARP1 confers resistance^11^, revealed an additional cytotoxic mechanism: upon PARP inhibition, PARP1 becomes tightly bound to DNA, a phenomenon known as PARP1 trapping^12,13^. The trapping potency of each PARPi strongly correlates with its cytotoxicity, demonstrating the importance of this mechanism for PARPi response^13–15^. Talazoparib is the most potent clinically approved PARP1 trapper^7,14,16^, used as a monotherapy to treat human epidermal growth factor receptor 2 (HER2)-negative, BRCA-mutated locally advanced or metastatic breast cancer, and in clinical trials for other cancers^3^. Trapping of PARP1 on DNA damage sites and unligated Okazaki fragments is thought to cause collisions with the replisome and fork stalling, which, in HRD or BRCA1/2-defective cells, collapse into unrepaired DSBs, leading to cell death^4,12–14^.

Despite their early promise, PARPi resistance, both acquired and de novo, is a major clinical concern^8,17,18^. In ovarian cancer, approximately 40% of patients with germline BRCA mutations show no response to olaparib^18–20^ or rucaparib^4,21^. This underscores the limited predictive power of current biomarkers, as deleterious BRCA/HRD mutations do not always reflect real-time HR function, therapy sensitivity or resistance dynamics^4,22–24^. The only clinically well-validated resistance mechanisms are BRCA/HR gene reversions and epigenetic regulation that restores HR, observed in hundreds of patients^8,25–27^. Pre-clinical evidence also indicates (1) non-reversion mutations that restore HR in BRCA1-defective cancers via loss of DNA end resection inhibitors^28–31^; (2) restored fork stability through inhibiting nuclease recruitment^32^; (3) loss of poly(ADP-ribose) glycohydrolase expression^33^; (4) upregulation of the ABCB1 efflux transporter, which limits PARPi accumulation^34–36^; and (5) PARP1 mutations that abolish trapping^11,37^. Greater insight into trapped PARP1 biology is necessary to define clinically relevant resistance mechanisms and improve therapeutic outcomes.

One area of interest is the proteolytic processing of trapped PARP1. The metalloprotease SPRTN^38^ and the unfoldase/segregase p97 (also known as VCP or Cdc48)^39^ extract trapped PARP1 from chromatin. Via its cofactor UFD1, p97 recognizes trapped PARP1 that has been SUMOylated and ubiquitylated by PIAS4 and RNF4, respectively, and extracts it from chromatin through its central pore^39^. p97, a promising druggable target with an inhibitor in clinical trials, resolves various protein-induced DNA lesions, including TOP1cc^40^ and trapped PARP1 (refs. ^39,41^), whose covalent or non-covalent association with DNA causes cytotoxic replisome blockade^42^. Given the broad roles of p97 in chromatin-associated processes, including DNA repair^43–48^, defining its regulation by cofactors and its downstream processing is crucial for therapeutic development. Our group recently uncovered an autophagy-dependent pathway by which p97 processes TOP1cc lesions from chromatin^49^. This pathway requires the p97 cofactor and selective autophagy receptor TEX264, which bridges topoisomerase inhibitor-induced TOP1cc to autophagosomes for degradation, preventing cytotoxic protein aggregates, limiting genome instability and shaping colorectal cancer responses to the topoisomerase I inhibitor Irinotecan.

Bulk autophagy is a cellular homeostasis pathway, best characterized under starvation, in which substrates are engulfed by elongating double-membrane phagophores that form autophagosomes, which then fuse with hydrolase-containing lysosomes^50^. Selective autophagy targets specific cargo via ATG8-family proteins on the growing phagophore, such as lipidated LC3, often using selective autophagy receptors (SARs) like TEX264 (refs. ^49,51,52^), which recognize specific substrates and bridge them to ATG8-family proteins^53,54^. Clinical trials with autophagy inhibitors chloroquine and hydroxychloroquine, mainly in combination therapies, show positive results, but the development of additional inhibitors is limited by tolerability^55^. All four US Food and Drug Administration (FDA)-approved PARPi upregulate autophagy, confirming cytoprotection in multiple cell lines and patient-derived xenografts^56–63^, but the role of autophagy in PARPi response requires further clarification for clinical translation.

To advance this, we investigated how trapped PARP1 is cleared from chromatin. We show that PARPi-induced autophagy is cytoprotective and interacts with trapped PARP1. Autophagy clears trapped PARP1 via the SAR TEX264 and its partner p97, which remove trapped PARP1 from chromatin and deliver it to the lysosome. Disrupting this pathway causes accumulation of cytotoxic PARP1 aggregates and genome instability, increasing cell lethality, including in PARPi-resistant cells. These findings support a TEX264-mediated nucleophagy pathway that promotes survival of HRD, particularly BRCA1-deficient mammalian cells and may have clinical relevance.

## Results

### Autophagy is upregulated upon PARPi treatment, with a cytoprotective effect

**Fig. 1:**
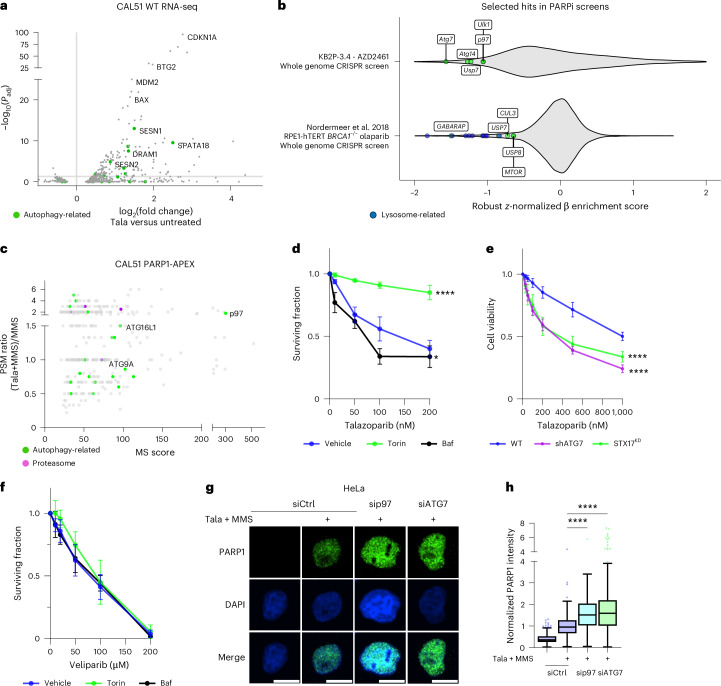
PARPi-induced upregulation of autophagy has a cytoprotective, trapping-related effect. **a**, Volcano plot showing differential gene expression in CAL51 cells comparing talazoparib (Tala; 100 nM, 24 h) to untreated cells by RNA-seq. Differential gene expression analysis was performed using DESeq2, with *P* values adjusted (*P*_adj_) for multiple testing using the Benjamini–Hochberg false discovery rate (FDR) method. The grey line indicates an adjusted *P* value of 0.05. **b**, Violin plots showing selected autophagy-related depletion hits from whole-genome CRISPR screens performed in mouse KB2P-3.4 (BRCA2-knockout) cells (top) and in human RPE-hTERT *BRCA1*^−/−^ cells (bottom) treated with AZD2461 and olaparib, respectively^31,76^. **c**, Mass spectrometry data from ref. ^39^ of PARP1 interactions enriched under PARP1-trapping conditions (Tala + MMS) by PARP1 WT–APEX2–eGFP proximity labelling. PSM, peptide-spectrum matches. **d**, Colony-formation assay in HeLa cells treated with talazoparib and bafilomycin A1 (Baf; 25 nM) or torin-1 (Torin; 150 nM) for 24 h. *n* = 3 biological replicates; mean ± s.e.m.; two-way analysis of variance (ANOVA) with Šídák’s multiple comparisons test. **e**,**f**, Cell viability measured by resazurin assay in WT HeLa cells, doxycycline (Dox)-inducible HeLa cells depleted of ATG7 with shRNA (shATG7), or HeLa cells with stable knockdown (KD) of syntaxin-17 (STX17), treated with either talazoparib (**e**) or veliparib (**f**) for 24 h, followed by 48 h recovery. *n*  = 3 biological replicates; mean ± s.e.m.; two-way ANOVA with Šídák’s multiple comparisons test. **g**, Immunofluorescence with detergent pre-extraction to detect trapped PARP1 foci in cells depleted for the indicated proteins using siRNA and treated with talazoparib and MMS. siCtrl, control siRNA. Scale bars, 10 μm. **h**, Quantification of immunstaining in **g** from two biological replicates and *n* = 371 (siCtrl untreated); *n* = 172 (siCtrl Tala + MMS); *n* = 148 (sip97 Tala + MMS); *n* = 164 (siATG7 Tala + MMS) cells. Normalization was performed by dividing each point by the mean of siCtrl treated within each replicate. Data are shown as box-and-whisker plots with median (centre line), interquartile range (IQR; box limits, 25th–75th percentiles), and whiskers extending to 1.5 × IQR; outliers are shown as individual points. Statistical analysis was performed using one-way ANOVA with Šídák’s multiple comparisons test. NS, not significant; *P ≤ 0.05; **P ≤ 0.01; ***P ≤ 0.001; ****P ≤ 0.0001. Exact *P* values and source numerical data are available in Source Data. Source data

**Extended Data Fig. 1:**
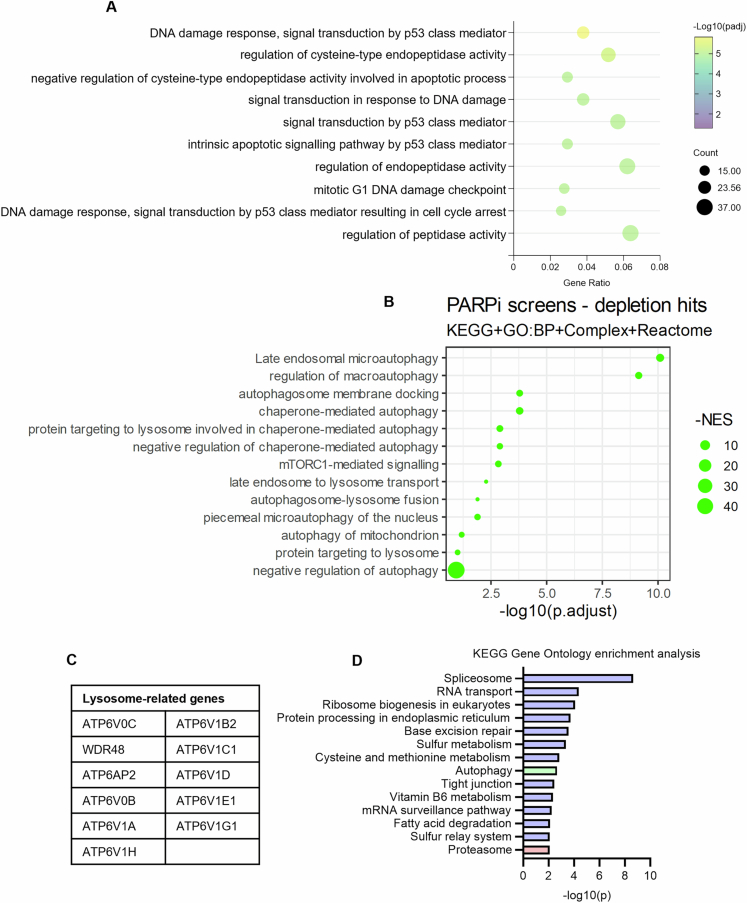
Functional pathway analysis in PARPi response. (**a**) Gene set enrichment analysis from RNA-seq of genes differentially expressed after treatment with talazoparib (100 nM, 24 h). KEGG pathway enrichment analysis was performed on differentially expressed genes identified by DESeq2 (FDR < 0.05) using Enrichr. Gene ratio represents the proportion of input genes associated with each pathway. *P* values were adjusted for multiple testing using the Benjamini–Hochberg method and are indicated by colour. The number of genes differentially expressed (gene count) is indicated by bubble size. (**b**) Functional enrichment analysis combined from two whole-genome PARPi CRISPR screens, shown in Fig. 1b ref. ^31,76^. Functional Enrichment analysis was performed using the R package *clusterProfiler* on the KEGG Pathways, Reactome, Gene Ontology: Biological Process and Complex databases. (**c**) List of lysosome-related genes shown by blue dots in Fig. 1b. (**d**) KEEG pathway enrichment analysis of PARP1 proximity labelling mass spectrometry data from Krastev et al. (2022)^39^, performed using Enrichr. Terms were filtered for −log(*P*) values ≥ 2, and ranked from top to bottom by significance, with autophagy appearing 8^th^ and proteasome appearing 14^th^.

PARPi induce trapping of PARP1 on chromatin, the proposed major mechanism of PARPi cytotoxicity^12,13,15,64,65^. Recent work has suggested that trapped PARP1 can be extracted from chromatin by the enzymatic activities of ATPase p97 and SPRTN protease, similar to other DNA–protein crosslinks (DPCs)^38,39,66,67^, potentially contributing to PARPi resistance. Through an RNA sequencing (RNA-seq) approach, we explored which protein homeostasis systems may be differentially expressed upon PARPi treatment, suggesting their potential involvement in trapped PARP1 repair. We performed RNA-seq on triple-negative breast cancer (TNBC) CAL51 cells after 24 h of treatment with potent PARP1-trapping talazoparib. As expected, we observed upregulation of genes related to DDR, replication stress, apoptosis, G1/S cell cycle checkpoint and mitotic checkpoint (Extended Data Fig. 1a and Supplementary Table 1). Top hits include CDKN1A and BTG2, involved in p53-dependent G1/S checkpoint DDR signalling^68^, MDM2, a p53 regulator that also has p53-independent roles in DNA synthesis and repair regulation^69^ and BAX, a pro-apoptotic protein^70^ (Fig. 1a and Supplementary Table 2). By probing with a comprehensive gene list for the autophagy pathway^71^, we observed upregulation of 31 autophagy-related genes upon treatment. Significantly upregulated genes included SESN1, SESN2 and DRAM1 (Fig. 1a and Supplementary Table 2), which are transcriptionally activated by p53 under genotoxic stress to promote autophagy^72^. In accordance with this, numerous studies show upregulation of autophagy in cells and patient-derived xenografts treated with PARPi^56–63,73^. We also probed with a Gene Ontology (GO) term list for proteasome-mediated ubiquitin-dependent catabolic processes (GO:0043161). Only 15 genes were differentially expressed (7 significantly upon treatment), but there was no differential expression with a GO list specific for the proteasome complex itself (GO:0000502). In fact, most proteasome-associated genes identified were E3 ligases, components of E3 ligases or E2 conjugating enzymes well established in DDR and cell cycle regulation in response to stress, such as MDM2, FBXW7 and UBE2C (Supplementary Table 2). Core proteasome protein transcripts are some of the most consistently expressed in human cancer cells^74^. While their lack of differential expression is unsurprising, few genes known to positively regulate the proteasome (five genes from GO:1901800) were differentially expressed. This suggests that PARPi may not induce proteasomal degradation to the same extent as autophagy.

To further explore whether autophagy is important in cellular response to PARPi, we explored two previously published whole-genome CRISPR screens for loss of autophagy factors affecting sensitivity to PARPi. One screen was performed in *Brca2*^−/−^ *Trp53*^−/−^ mouse mammary KB2P-3.4 tumour cells (ref. ^75^) treated with the PARPi AZD-2461 (ref. ^76^), while the other was performed in human RPE-hTERT *TP53*^−/−^*BRCA1*^−/−^ cells treated with olaparib^31^. Following functional enrichment analysis across four databases, we identified several significantly enriched gene sets related to autophagy among the sensitivity candidates (Extended Data Fig. 1b), suggesting that loss of these genes confers sensitivity to PARPi. Regulation of macroautophagy emerged as one of the most significantly enriched pathways. We found that loss of a handful of core autophagy factors significantly sensitizes cells to PARPi. These include genes encoding ATG7, ATG14, ULK1 and GABARAP. Loss of autophagy regulators USP7 (ref. ^77^), USP8 (refs. ^78,79^), CUL3 (ref. ^80^) and p97 ^81^ implicate cell sensitivity to PARPi (Fig. 1b). Several of the depleted genes are involved in lysosome biogenesis and function, or are subunits of v-ATPase, which is key in autophagic degradation (Fig. 1b and Extended Data Fig. 1c). From these data, impaired autophagy seems to cause increased sensitivity to two different PARPi and in cell lines with either BRCA1 or BRCA2 deficiency, which constitute the majority of tumours treated with PARPi in the clinic.

To further understand the role of autophagy in PARPi response, we turned to mass spectrometry data from our previous work^39^, which identified trapped PARP1-interacting proteins defined by PARP1–APEX proximity labelling. Interestingly, in gene set enrichment analysis, autophagy appeared as the eighth most significant gene set enriched under PARP1-trapping conditions (Extended Data Fig. 1d). To explore this, we probed this dataset with the same autophagy gene list used when analysing RNA-seq data and identified 22 autophagy-related proteins interacting with trapped PARP1 (Fig. 1c and Supplementary Table 3). Alongside many autophagy regulators, we identified ATG9A and ATG16L1, two key regulators of autophagosome biogenesis, indicating the proximity of trapped PARP1 with the core autophagy machinery. Despite a lack of enrichment in our RNA-seq data, the proteasome ranked 14th by significance in gene set enrichment analysis (Extended Data Fig. 1d), with four proteasome subunits (PSMA6, PSMB5, PSMD2 and PSMD12) identified in the PARP1 interactome (Fig. 1c). Proteasomal degradation of trapped PARP1 in basal conditions is well characterized^82–84^. The presence of so many core autophagy genes in the trapped PARP1 proteome indicates a potential direct role of autophagy in regulating chromatin-bound PARP1. When considering this alongside its upregulation by RNA-seq (Fig. 1a) and in previous literature^57^, as well as the PARPi-sensitizing effect of autophagy gene loss observed in CRISPR screens (Fig. 1b and Extended Data Fig. 1b), autophagy seems an attractive target for further exploration.

**Extended Data Fig. 2:**
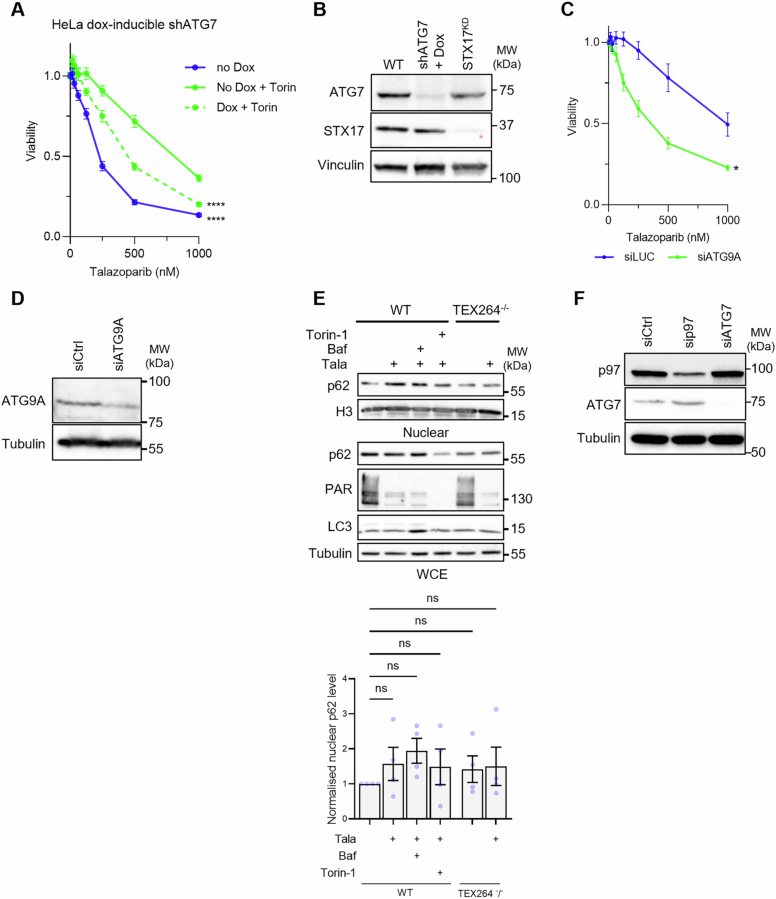
Additional data supporting Fig. 1. (**a**) Cell viability measured by resazurin assay in dox-inducible shATG7 HeLa cells treated with talazoparib with or without torin-1 (150 nM) for 24 h, followed by 48 h recovery. *n* = 3 independent biological replicates, mean ± s.e.m.; statistical analysis was performed using two-way ANOVA with Dunnett’s multiple comparisons test. (**b**) Immunoblot validating depletion of indicated proteins for Fig. 1e and f. Cell lines are HeLa WT (left lane), HeLa doxycycline-inducible shATG7 (middle lane) and HeLa STX17^KD^ (right lane). (**c**) Cell viability measured by resazurin assay in HeLa cells with depletion of ATG9A using siRNA, treated with talazoparib for 24 h, followed by 48 h recovery. *n* = 3 independent biological replicates, mean ± s.e.m.; statistical analysis was performed using two-sided unpaired t-test. (**d**) Immunoblot validating depletion of ATG9A in fig C. Representative image of three biological replicates. (**e**) Cellular fractionation of HeLa WT and TEX264^−/−^ cells treated with the indicated drugs for 3 h. The lower panel shows quantification from 4 biological repeats. mean ± s.e.m.; statistical analysis was performed using one-way ANOVA with Dunnett’s multiple comparisons test. (**f**), Immunoblotting validating depletion of p97 and ATG7 for experiments in Fig. 1g and h. Representative image of three biological replicates. Statistical analyses are indicated in each panel. Statistical significance was defined as: ns, not significant; *P < 0.05; **P < 0.01; ***P < 0.001; ****P < 0.0001. Exact *P* values, source numerical data and unprocessed blots are available in Source Data. Source data

We began by exploring how modulation of autophagy impacts cellular response to PARPi. We treated cells with talazoparib in combination with either bafilomycin A1, to impede autophagy through inhibition of lysosome acidification, or torin-1, to boost autophagic flux through mTOR inhibition. Torin-1 treatment caused a striking resistance to talazoparib, while inhibition of autophagy with bafilomycin A1 increased sensitivity (Fig. 1d). As mTOR has multiple functions, we combined torin-1 treatment with depletion of ATG7, an E1-like enzyme involved in conjugating LC3 to the membrane during phagophore formation and expansion^50,55^ (Extended Data Fig. 2a). Resistance to talazoparib was partially but significantly reversed by ATG7 depletion, suggesting that PARPi resistance induced by torin-1 is very likely due to increased autophagy flux. We confirmed the association of autophagy and PARPi genetically through autophagy inhibition either by depletion of ATG7 with short hairpin RNA (shRNA) or knockdown of syntaxin-17, an autophagosomal SNARE protein that regulates multiple autophagic processes, including autophagosome membrane fusion with the lysosome^85,86^. Depletion of either of these autophagy factors increased the sensitivity of cells treated with talazoparib (Fig. 1e and Extended Data Fig. 2b). Depletion of ATG9A, a lipid scramblase involved in phagophore formation and identified as an interactor of PARP1 (Fig. 1c), also caused increased sensitivity to talazoparib (Extended Data Fig. 2c,d). While the reversal of torin-1-induced PARPi resistance by ATG7 depletion may be merely an additive effect, we have demonstrated PARPi hypersensitivity by autophagy inhibition using both genetic and chemical tools. This is in line with our findings from the CRISPR screen data (Fig. 1b and Extended Data Fig. 1b) and various recent studies showing increasing sensitivity to PARPi by autophagy inhibition^56,57,59–62^. This suggests, as previously seen, that upregulation of autophagy upon PARPi treatment has a cytoprotective effect.

PARPi sensitivity boosted by autophagy impairment has been previously demonstrated, and various explanations have been suggested. These include PARPi-induced upregulation of PTEN to promote cytoprotective autophagy in response to PARPi-induced ROS^62^ and nuclear localization of p62, indirectly causing an upregulated HRR pathway^87^. Under conditions that induce or block trapped PARP1 delivery to the lysosome, we did not observe a considerable and consistent change in the nuclear localization of p62 (Extended Data Fig. 2e), indicating an alternative role of autophagy.

Notably, we did not observe the same cytoprotective effect of autophagy when cells were treated with veliparib, a PARPi that inhibits PARP1 catalytic activity but causes only weak, if any, trapping^14,16^, with neither bafilomycin A1 nor torin-1 treatment affecting cell sensitivity (Fig. 1f). This indicates that the observed effect is linked to PARP1 trapping. Using detergent pre-extraction to remove soluble proteins, but not those that are tightly bound to chromatin, we visualized trapped PARP1 on chromatin by immunofluorescence under trapping conditions, when cells were treated with talazoparib and a low dose of the alkylating agent methyl methanesulfonate (MMS)^39^ (Fig. 1g,h and Extended Data Fig. 2f). PARP1 is only visualized on chromatin under trapping conditions, confirming the efficacy of this assay for detecting specifically trapped PARP1. Trapped PARP1 levels were significantly increased (∼1.7-fold) upon autophagy inhibition by depletion of ATG7 (Fig. 1h), similar to p97-depleted cells, which are known to accumulate trapped PARP1 (ref. ^39^). Altogether, this suggests that autophagy upregulation upon PARPi treatment has a cytoprotective effect by restricting accumulated trapped PARP1.

### Trapped PARP1 is processed by autophagy

**Fig. 2:**
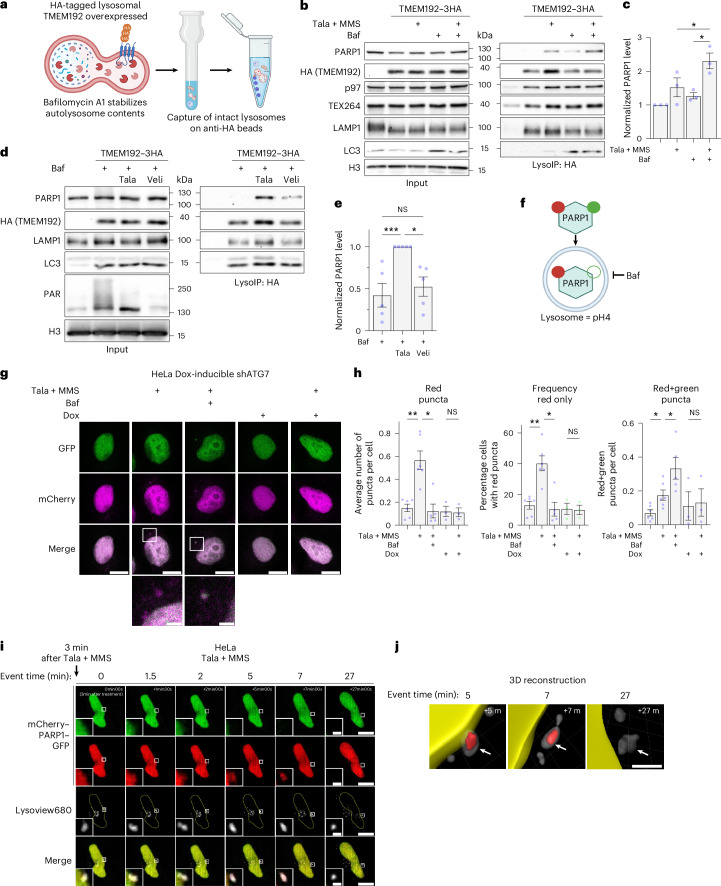
Trapped PARP1 is processed by selective autophagy. **a**, Schematic of the LysoIP workflow, whereby TMEM192–3HA is stably expressed in cells treated with bafilomycin A1, then immunoprecipitated to isolate intact lysosomes. HA, haemagglutinin. Panel **a** created in BioRender; Torrecilla, I. https://biorender.com/55xhb64 (2026). **b**, LysoIP in HeLa cells treated with the indicated drugs for 3 h, showing the accumulation of PARP1 in the lysosome under trapping conditions. **c**, Quantification of PARP1 levels in **b**, normalized to untreated cells. *n* = 3 biological replicates; mean ± s.e.m.; one-way ANOVA with Šídák’s multiple comparisons test. **d**, LysoIP in HeLa cells treated with either talazoparib (Tala; 200 nM) or veliparib (Veli; 5 µM) for 3 h. **e**, Quantification of PARP1 levels in **d** normalized to talazoparib-treated cells. *n* = 5 biological replicates; mean ± s.e.m.; one-way ANOVA with Šídák’s multiple comparisons test. **f**, Schematic showing the methodology of the mCherry–GFP reporter assay. PARP1 tagged with mCherry and GFP is transiently expressed in cells and visualized by either fixed or live imaging. GFP is quenched in acidic environments, so only the red signal is detected in the lysosome. This can be reversed by treatment with bafilomycin A1, which neutralizes the lysosome. **g**, Fixed images from the mCherry–PARP1–GFP reporter assay in HeLa Dox-inducible shATG7 cells treated with the indicated drugs for 3 h. Scale bars, 10 µm. Zoomed images show puncta indicated by white boxes; scale bar, 2 µm. **h**, Quantification of mCherry–GFP puncta in **g**. *n* = 6 (Tala + MMS); *n* = 5 (Tala + MMS + Baf); or *n* = 3 (Dox and Tala + MMS + Dox) biological replicates; mean ± s.e.m.; mixed-effect model with Šídák’s multiple comparisons test. **i**, Representative *z*-projected images from live-cell imaging of HeLa cells transfected with the mCherry–PARP1–GFP reporter fusion protein (19 h after transfection) and stained with LysoView680. Time stamps indicate the elapsed time from the start of imaging for the event shown; the times of talazoparib and MMS addition are indicated in brackets in the first panel. Degradation of the lysosomal substrate occurred at 27 min. Scale bars, 10 µm; inset scale bars, 1 µm. **j**, Imaris 3D rendering at 5 min, 7 min and 27 min of the nucleus and surrounding lysosomes as categorized by mCherry–PARP1–GFP and LysoView680, respectively. Arrows mark the same lysosome as shown in **i**. Yellow, nucleus; near transparent, lysosomes; red, mCherry–PARP1–GFP in acidic environment (GFP quenched). Scale bar, 2 µm. NS, not significant; *P ≤ 0.05; **P ≤ 0.01; ***P ≤ 0.001; ****P ≤ 0.0001. Exact *P* values, source numerical data and unprocessed blots are available in Source Data. Source data

**Extended Data Fig. 3:**
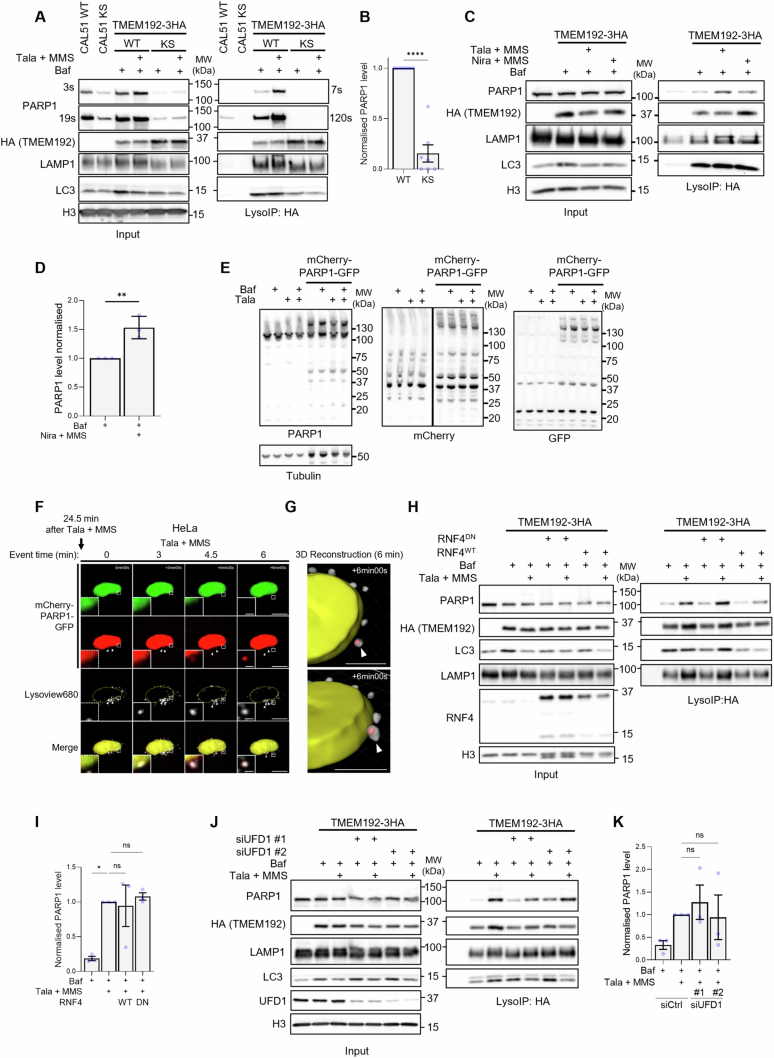
Additional data supporting Fig. 2 and Fig. 3. (**a**) LysoIP in CAL51 cells stably expressing either PARP1^WT^ or DNA-binding mutant PARP1del.p.119K120S (PARP1^KS^) treated with the indicated drugs for 3 h. (**b**) Quantification of (A). Lysosomal PARP1 signal intensity was normalized by dividing by the input PARP1 signal and lysoIP HA signal. *n* = 7 independent biological replicates; mean ± s.e.m.; statistical analysis was performed using a two-sided unpaired t-test. (**c**) LysoIP in HeLa cells treated with the indicated drugs, including niraparib (500 nM) for 3 h. (**d**) Quantification of PARP1 levels in (**c**), normalized to bafilomycin A1-treated. *n* = 3 independent biological replicates; mean ± s.e.m.; statistical analysis was performed using a two-sided unpaired t-test. (**e**) Immunoblot showing HeLa cells either untransfected or transfected with mCherry–PARP1–GFP (mCh-P1-GFP) for 24 h, then treated with the indicated drugs for 3 h. Representative images of two biological replicates (**f**) Representative z-projected images from live-cell imaging of HeLa cells transfected with the mCherry–PARP1–GFP reporter fusion protein (19 h post-transfection) and stained with LysoView680. Time stamps indicate the elapsed time from the start of imaging for the event shown; the times of Talazoparib and MMS addition are indicated in brackets in the first panel. Imaging was stopped before complete degradation of the lysosomal content. Scale bar, 10 µm; inset scale bar, 1 µm. Arrows mark two earlier events in the same nucleus occurring 15 minutes and 18 min after talazoparib and MMS addition, respectively. The third event (denoted by the square) occurs at 24min30s after the addition of talazoparib and MMS addition. (**g**) Two different orientations of the Imaris 3D rendering at 6 minutes of the nucleus and surrounding lysosomes as categorized by mCherry–PARP1–GFP and LysoView680, respectively. Arrows mark the same lysosome as shown in (**f**). Yellow, nucleus; Near transparent, lysosomes; Red, mCherry–PARP1–GFP in acidic environment (GFP quenched). Scale bar, 5 µm. (**h**) LysoIP in HeLa cells with overexpression of either RNF4 WT (RNF4^WT^) or RNF4 dominant-negative M136A + R177A mutant (RNF4^DN^) treated with the indicated drugs for 3 h. (**i**) Quantification of PARP1 levels in (**f**), normalized to the treated control. *n* = 3 independent biological replicates; mean ± s.e.m.; statistical analysis was performed using one-way ANOVA with Šídák’s multiple comparisons test. (**j**) LysoIP in HeLa cells depleted of UFD1 using two different siRNA sequences treated with the indicated drugs for 3 h. (**k**) Quantification of PARP1 levels in (H), normalized to the treated control. *n* = 3 independent biological replicates; mean ± s.e.m. Statistical analysis was performed using one-way ANOVA with Šídák’s multiple comparisons test. Statistical analyses are indicated in each panel. Statistical significance was defined as: ns, not significant; *P < 0.05; **P < 0.01; ***P < 0.001; ****P < 0.0001. Exact *P* values, source numerical data and unprocessed blots are available in Source Data. Source data

As trapped PARP1 levels were increased upon autophagy inhibition and early-stage autophagy core machinery was found in proximity to trapped PARP1 by mass spectrometry (Fig. 1c), we hypothesized that trapped PARP1 could be cleared by autophagy. To explore this, intact lysosomes were isolated from HeLa and CAL51 cells expressing lysosomal transmembrane protein TMEM192–3HA by a method known as LysoIP (immunoprecipitation of intact lysosomes) to analyse lysosomal contents^88^ (Fig. 2a) in control and PARP1-trapping conditions. PARP1 was localized in the lysosome and accumulated considerably under PARP1-trapping conditions in both HeLa and CAL51 cells (Fig. 2b,c and Extended Data Fig. 3a,b). Lysosomal PARP1 levels upon trapping were increased further by treatment with bafilomycin A1, stabilizing its contents, further confirming that PARP1 localizes in the lysosome (Fig. 2b,c). PARP1 was more extensively localized to the lysosome when treated with the strong PARP1-trapping inhibitors talazoparib and niraparib, compared with veliparib, a weak trapper of PARP1 (Fig. 2d,e and Extended Data Fig. 3c,d). In CAL51 cells expressing either WT PARP1 (PARP1^WT^) or a trapping-impaired mutant PARP1^del.p.119K120S^ (PARP1^KS^)^11^, only PARP1^WT^ was localized to the lysosome (Extended Data Fig. 3a,b). Whole-cell levels of PARP1^KS^ were lower than PARP1^WT^, but even when its lysosomal level was normalized against this across seven biological repeats, we observed a robust reduction in lysosomal PARP1^KS^ compared with PARP1^WT^, highlighting that only trapped PARP1 is processed in this way. To further validate this, we used a modified version of the well-recognized mCherry–GFP autophagy reporter assay, whereby cells were transfected with a PARP1 construct tagged with mCherry and green fluorescent protein (GFP). This construct is well expressed and remains intact during treatment with talazoparib and bafilomycin A1 (Extended Data Fig. 3e). In most cellular compartments, including the nucleus, both mCherry and GFP will fluoresce and colocalize. However, in the acidic environment of the lysosome, GFP is quenched, so only the mCherry signal is observed^89^ (Fig. 2f). The PARP1 reporter is predominantly localized in the nucleus, where the red and green signals were observed most strongly and colocalized. However, under trapping conditions, cytosolic red puncta of PARP1 formed (Fig. 2g,h), indicating its localization to the lysosome. These fluoresced both red and green when bafilomycin A1 was added to neutralize the lysosomal pH and prevent quenching of the GFP signal, confirming the specificity of this assay for lysosome-localized PARP1 (Fig. 2g,h). Depletion of ATG7 reduced the number of cytosolic puncta to background levels seen without treatment, further confirming that PARP1 exits the nucleus in an autophagy-dependent manner upon trapping. To further confirm the localization of PARP1 to the lysosome after treatment, we visualized mCherry–GFP-tagged PARP1 in cells stained with a lysosome dye by live imaging. Within minutes of treatment, lysosomes approached the nuclear periphery and strongly colocalized with the PARP1 signal as it exited the nucleus (Fig. 2i,j, Extended Data Fig. 3f,g and Supplementary Videos 1 and 2). Puncta emerged from the nucleus and accumulated over the 1-h assay. They localized with lysosomes for ~20 min before degradation and lysosome dispersal. Rendering enhanced separation of the green signal from the background and improved lysosome visualization. Together, this further confirms that PARP1 localizes to the lysosome under PARP1-trapping conditions.

**Fig. 3:**
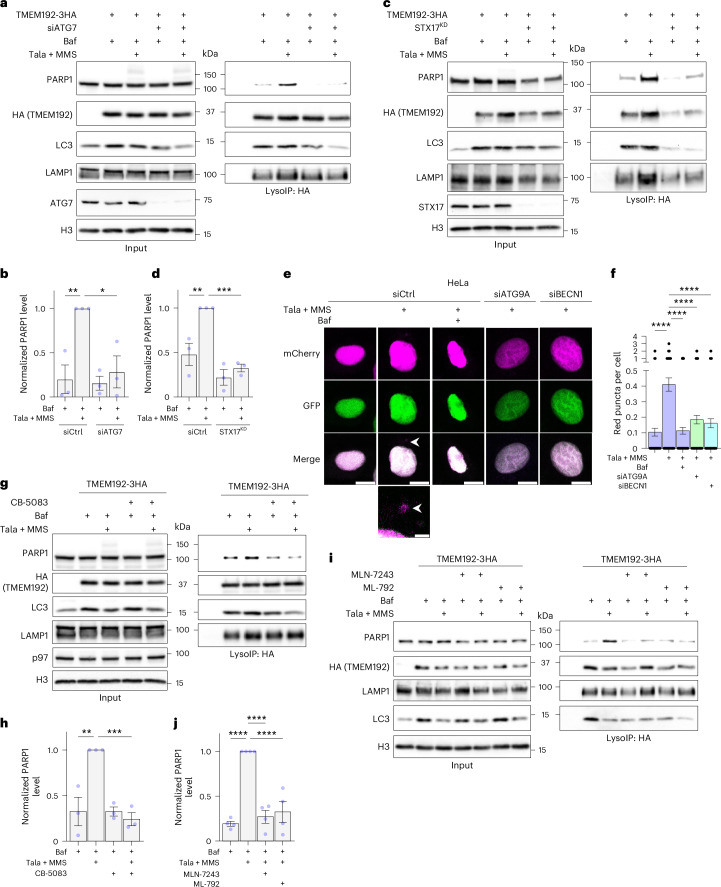
Trapped PARP1 is processed by p97, ubiquitin and SUMO-dependent autophagy. **a**, LysoIP in HeLa cells with depletion of ATG7 by siRNA. **b**, Quantification of PARP1 levels in **a**, normalized to the treated control. **c**,**d**, As in **a** and **b**, but in either WT cells or cells with stable knockdown of syntaxin-17 (STX17^KD^). **e**, Fixed images from the mCherry–PARP1–GFP reporter assay in HeLa cells depleted of either ATG9A (siATG9A) or beclin-1 (siBECN1) with siRNA and treated with the indicated drugs for 3 h. Scale bars,10 µm. Zoomed image shows puncta indicated by a white arrowhead; scale bar, 2 µm. The brightness and contrast in the zoom panel has been adjusted compared with the larger images to better show the puncta. **f**, Quantification of mCherry–GFP puncta in **e**. **g**, LysoIP in HeLa cells treated with talazoparib and MMS, combined with p97 inhibitor CB-5083 (10 μM). **h**, Quantification of PARP1 levels in **g**, normalized to the treated control. **i**, LysoIP in HeLa cells treated with the indicated drugs, including ubiquitination inhibitor MLN-7243 (5 μM) or SUMOylation inhibitor ML-792 (1 μM). **j**, Quantification of PARP1 levels in **i**, normalized to the treated control. Quantification graphs in **b**,**d**,**f**,**h** present three independent replicates and in **j** present four independent replicates; mean ± s.e.m.; one-way ANOVA with Šídák’s multiple comparisons test. *P ≤ 0.05; **P ≤ 0.01; ***P ≤ 0.001; ****P ≤ 0.0001. Exact *P* values, source numerical data and unprocessed blots are available in Source Data. Source data

To confirm whether this lysosomal localization of trapped PARP1 is dependent on autophagy, we depleted ATG7 and assessed lysosomal PARP1 levels by LysoIP and the mCherry–PARP1–GFP reporter assay. Depletion of ATG7 almost entirely abolished both the recruitment of PARP1 to the lysosome under trapping conditions (Fig. 3a,b) and the accumulation of red PARP1 puncta (Fig. 2g,h). We observed a similar effect when autophagy was inhibited through depletion of syntaxin-17 (Fig. 3c,d), ATG9A or beclin-1 (Fig. 3e,f). As these proteins have distinct functions at different stages of autophagy, this demonstrates that PARP1 is processed by autophagy under trapping conditions.

### Autophagosomal processing of trapped PARP1 is dependent on p97, ubiquitination and SUMOylation

Having demonstrated that processing of trapped PARP1 occurs via selective autophagy, we next wanted to explore whether this occurs downstream of the previously described p97-dependent extraction of trapped PARP1 from chromatin^39^. Besides its chromatin role in removing various DNA-associated substrates, p97 was also implicated in autophagy and moves between the cytoplasm and nucleus depending on cellular need^90–93^. However, the role of p97 in autophagy remains to be fully understood. Using LysoIP to assess PARP1 levels in the lysosome under trapping conditions, we inhibited p97 with the specific and clinically relevant inhibitor CB-5083 (ref. ^41^). CB-5083 treatment significantly reduced the accumulation of PARP1 in lysosomes (Fig. 3g,h), suggesting a role for p97 in mediating this process.

p97 is recruited to trapped PARP1 partly through SUMOylation and subsequent ubiquitylation^39^, with the latter often involved in selective autophagy. Inhibition of either ubiquitination or SUMOylation caused reduced lysosomal engulfment of PARP1 under trapping conditions (Fig. 3i,j). Trapped-PARP1 ubiquitination has previously been shown to be mediated by the SUMO-targeted E3 ubiquitin ligase RNF4 (ref. ^39^). Modulating RNF4 activity, either by expressing its dominant-negative, enzymatically inactive M136A + R177A variant (RNF4^DN^) or by overexpressing WT (RNF4^WT^), did not affect lysosomal PARP1 levels (Extended Data Fig. 3h,i). This negates the role of RNF4-mediated ubiquitination of PARP1 in the autophagic processing of trapped PARP1. Instead, RNF4-mediated ubiquitination of trapped PARP1 likely mediates its proteasomal degradation, as has been described for most other substrates^94–96^. In the p97–RNF4 pathway previously described, p97 is recruited to ubiquitinated PARP1 by cofactor UFD1 (ref. ^39^). As with RNF4 modulation, depletion of UFD1 did not affect trapped PARP1 levels in the lysosome (Extended Data Fig. 3j,k). Altogether, this suggests that the previously described RNF4–UFD1–p97 pathway acts separately from the autophagy-mediated processing of trapped PARP1. p97 is still involved but must rely on other unknown SUMO and/or ubiquitin ligases and p97 cofactor(s) acting to facilitate this pathway.

### Selective autophagy of trapped PARP1 is TEX264-dependent but nuclear pore-independent

**Fig. 4:**
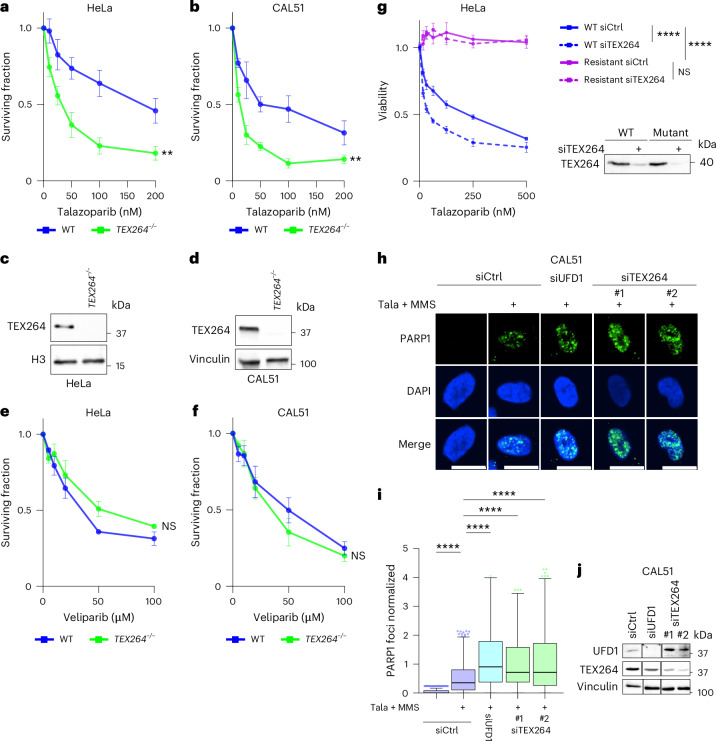
TEX264 acts as a regulator of PARP1 trapping. **a**,**b**, Colony-formation assay in WT and *TEX264*^−/−^ HeLa (**a**) and CAL51 (**b**) cells treated with talazoparib for 24 h. *n* = 5 (**a**) and *n* = 3 (**b**) biological replicates; mean ± s.e.m.; two-sided paired *t*-test. **c**,**d**, Immunoblotting validating knockout of TEX264 in HeLa (**c**) and CAL51 (**d**) cells. **e**,**f**, As in **a** and **b**, but treated with veliparib instead of talazoparib. *n* = 3 (**e**) and *n* = 4 (**f**) biological replicates; mean ± s.e.m. **g**, Cell viability by resazurin assay in HeLa cells expressing either PARP1^WT^ or DNA-binding mutant PARP1^(p.43delIM;44F>I)^, which are resistant to PARPi. Cells were depleted of TEX264 with siRNA (siTEX264) and treated with talazoparib for 24 h, followed by 48 h of recovery. *n* = 3 biological replicates; mean ± s.e.m.; two-way ANOVA. Immunoblot validates PARP1 expression and TEX264 depletion. **h**, Images of trapped PARP1 foci visualized by immunofluorescence with detergent pre-extraction after talazoparib and MMS treatment in CAL51 cells depleted of either UFD1 (siUFD1) or TEX264 (siTEX264) with siRNA. Scale bars, 10 µm. **i**, Quantification of PARP1 foci in **h**. *n* = 3 biological replicates. Box-and-whisker plots as in Fig. 1h. Statistical analysis was performed using one-way ANOVA with Šídák’s multiple comparisons test. **j**, Immunoblotting validating depletion of UFD1 and TEX264 for **h** and **i**. *P ≤ 0.05; **P ≤ 0.01; ***P ≤ 0.001; ****P ≤ 0.0001. Exact *P* values, source numerical data and unprocessed blots are available in Source Data. Source data

**Extended Data Fig. 4:**
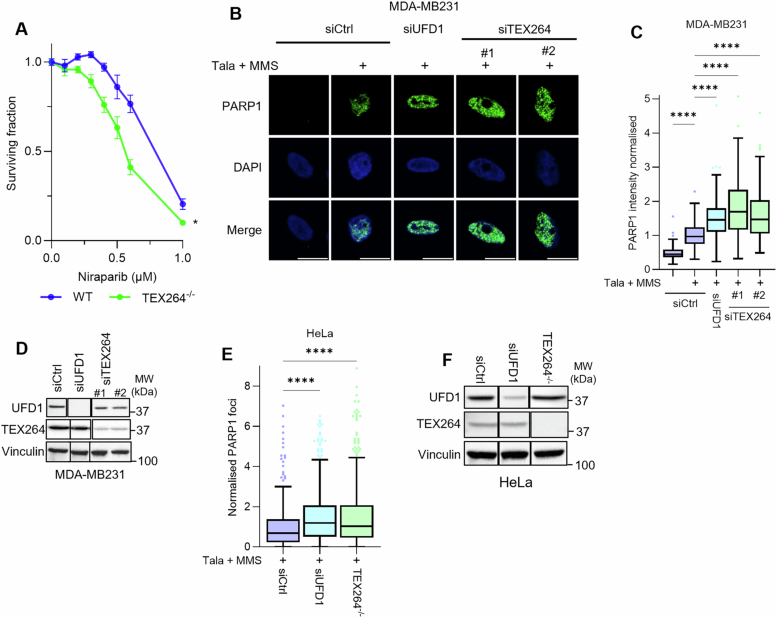
Additional data supporting Fig. 4. (**a**) Colony formation assay in HeLa WT and TEX264^−/−^ cells treated with niraparib for 24 h. Data represent three biological repeats; mean ± s.e.m.; statistical significance was assessed by two-sided paired t-test. (**b**) Images of trapped PARP1 as detected by pre-extraction immunofluorescence in MDA-MB231 cells depleted for indicated proteins and treated with talazoparib and MMS. Scale bar, 10 µm. (**c**) Quantification of trapped PARP1 levels in (**b**) from two biological repeats and *n* = 167 (siCtrl utd); *n* = 161 (siCtrl Tala + MMS); *n* = 116 (siUFD1 Tala + MMS); *n* = 111 (siTEX264 #1 Tala + MMS); and *n* = 92 (siTEX264 #2 Tala + MMS) cells. (**d**) Validation of depletion for (**b** and **c**) by immunoblotting. (**e**) Quantification of trapped PARP1 foci as detected by pre-extraction immunofluorescence in HeLa cells depleted of UFD1 by siRNA or with TEX264^−/−^ from 3 biological repeats and *n* = 393 (HeLa siCtrl Tala); *n* = 446 (HeLa siUFD1 #1 Tala); *n* = 275 (HeLa TEX264−/− Tala) cells. (**f**) Immunoblotting validating knockdown or knockout of proteins in (**e**). Graphs in (**c**) and (**e**) are shown as box-and-whisker plots with median (centre line), interquartile range (box limits, 25th–75th percentiles), and whiskers extending to 1.5× IQR; outliers are shown as individual points. Statistical analysis in (**c**) and (**e**) was performed using one-way ANOVA with Šídák’s multiple comparisons test. Statistical significance was defined as: ns, not significant; *P < 0.05; **P < 0.01; ***P < 0.001; ****P < 0.0001. Exact *P* values, source numerical data and unprocessed blots are available in Source Data. Source data

To further understand the processing of trapped PARP1, we wanted to identify the SAR responsible. TEX264 has recently been identified as a SAR for the endoplasmic reticulum under starvation conditions^51,52,97,98^, and as a p97 cofactor and SAR essential for the removal of TOP1cc from chromatin by autophagy^40,49^. Due to the similarity between TOP1cc and trapped PARP1 in blocking replication machinery, alongside the ability of TEX264 to act as both a SAR and p97 cofactor^40,49,97^, we explored whether TEX264 may act in this pathway. We tested TEX264-KO (^−/−^) cells for their sensitivity to talazoparib and niraparib and found that loss of TEX264 led to a profound increase in sensitivity of both HeLa and CAL51 cells (Fig. 4a–d and Extended Data Fig. 4a). Similar to the modulation of autophagy (Fig. 1d,f), this effect was not observed in cells treated with veliparib (Fig. 4e,f). This suggests that the function of TEX264 in protecting against PARPi-induced cell death is linked to trapped PARP1. To confirm this, TEX264 was depleted, and PARPi sensitivity was assessed in cells expressing either PARP1^WT^ or a mutant previously shown to induce PARPi resistance by preventing PARP-trapping (PARP1^(^^p.43^^delIM;44^^F^^>^^I^^)^)^11^. TEX264 depletion selectively hypersensitized PARP1^WT^ cells to talazoparib but not cells expressing the trapping-defective mutant (Fig. 4g). A considerable accumulation of trapped PARP1 was also observed after either depletion or knockout of TEX264 in three different human cell lines, including HeLa, CAL51 and MDA-MB231 (Fig. 4h–j and Extended Data Fig. 4b–f). The level of trapped PARP1 observed was equivalent to the depletion of a previously identified cofactor, UFD1, supporting that TEX264 acts as a physiologically relevant modulator of trapped PARP1 levels.

**Fig. 5:**
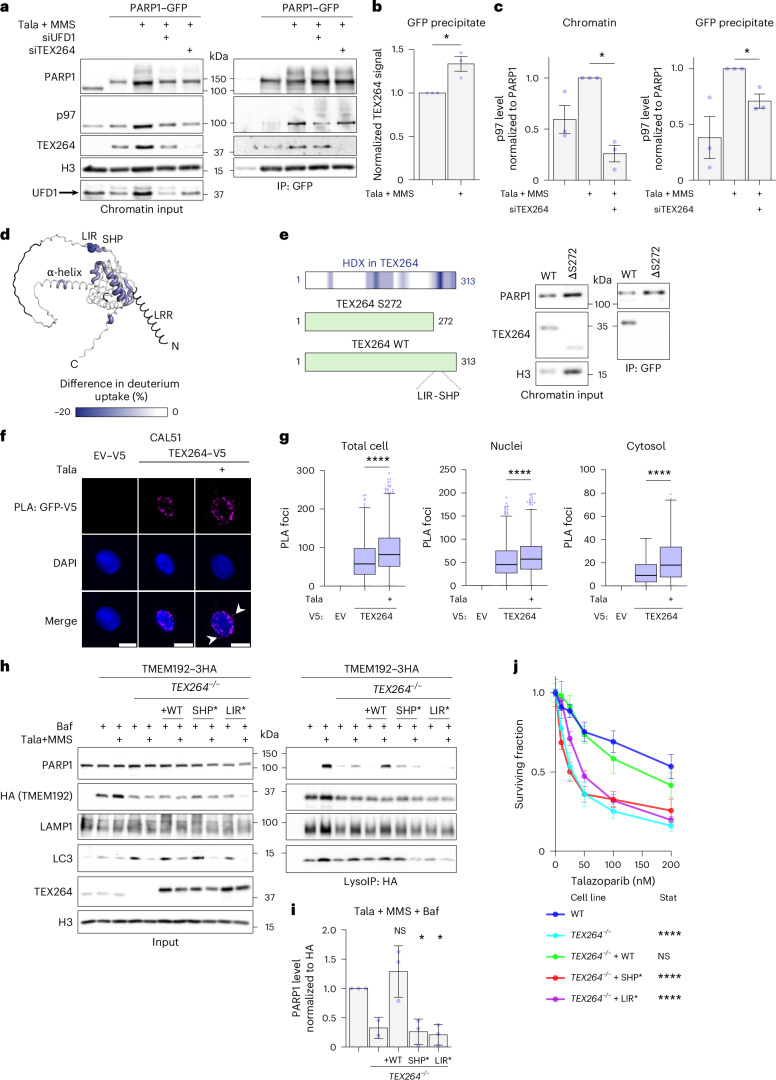
TEX264 serves as a p97-mediated selective autophagy receptor for trapped PARP1. **a**, Co-immunoprecipitation (co-IP) of GFP from chromatin of CAL51 cells stably expressing PARP1–GFP after treatment with talazoparib and MMS for 3 h. **b**, Quantification of TEX264 levels in the GFP-precipitate fraction in **a**, normalized to PARP1 signal to account for differences in binding to beads and to the untreated level. *n* = 3 biological replicates; mean ± s.e.m.; two-sided unpaired Student’s *t*-test. **c**, Quantification of p97 levels in chromatin (left) and GFP–PARP1 co-IP (right) in **a**, normalized to PARP1 levels. *n* = 3 biological replicates; mean ± s.e.m.; two-sided Student’s unpaired *t*-test. **d**, Structure of TEX264 with enlarged blue regions indicating altered deuterium uptake upon PARP1 binding in HDX-MS with 30 s of labelling. α-helix and C-terminal regions are labelled as areas with altered deuterium uptake. The heatmap indicates a 0–20% difference in deuterium uptake. **e**, Co-immunoprecipitation of GFP from chromatin of HeLa cells stably expressing PARP1–GFP after treatment with talazoparib and MMS for 3 h. Schematics (left) show the TEX264 variants compared with a map showing sites of interest highlighted by HDX-MS. **f**, PLA between GFP and V5 in cells stably expressing PARP1–GFP and either TEX264–V5 or empty vector (EV)–V5 after treatment with talazoparib for 3 h. Scale bars, 10 µm. **g**, Quantification of foci in **f** in whole cell, cytosol or nuclei. Data represent *n* = 3 biological replicates. Box-and-whisker plots as in Fig. 1h. Statistical analysis was performed using one-way ANOVA with Šídák’s multiple comparisons test. **h**, LysoIP in HeLa cells stably expressing TMEM192–3HA with or without *TEX264*^−/−^. TEX264–V5 variants (SHP* and LIR*) are transiently expressed where indicated. **i**, Quantification of PARP1 levels in **h**, *n* = 3 biological replicates, except for *TEX264*^−/−^ cells, which was included in two replicates, normalized to the WT control; mean ± s.e.m.; one-way ANOVA compared with WT. **j**, Colony-formation assay in WT CAL51 cells, *TEX264*^−/−^ cells or *TEX264*^−/−^ cells stably expressing indicated TEX264 variants, treated with talazoparib for 24 h. Six technical replicates from two biological replicates; mean ± s.e.m.; two-way ANOVA compared with WT. *P ≤ 0.05; **P ≤ 0.01; ***P ≤ 0.001; ****P ≤ 0.0001. Exact *P* values, source numerical data and unprocessed blots are available in Source Data. Source data

**Extended Data Fig. 5:**
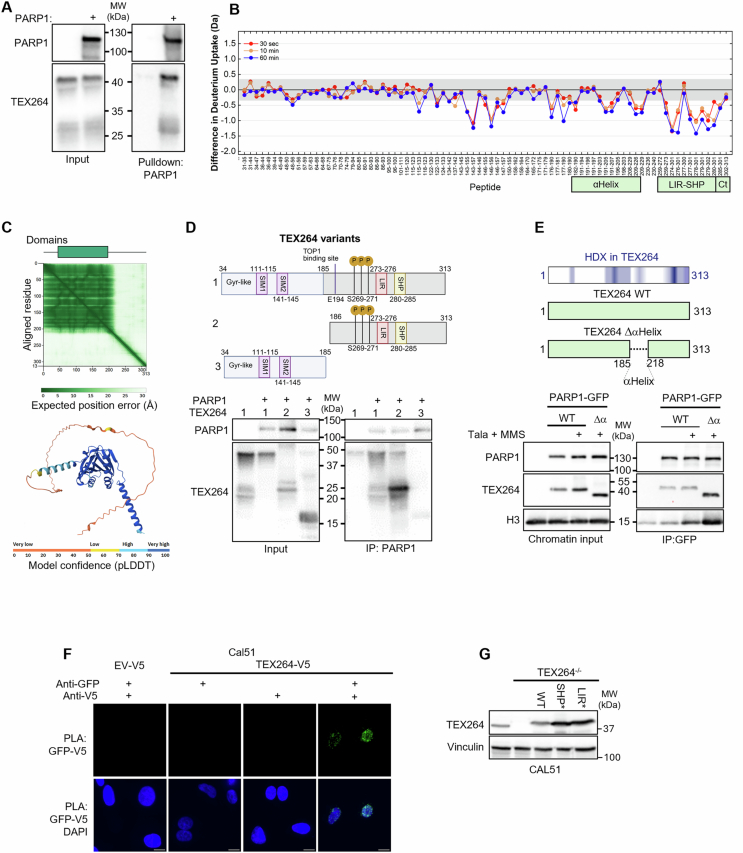
Additional data supporting Fig. 5. (**a**) *In vitro* pulldown of PARP1–GFP combined with TEX264, showing interaction between the two proteins. Representative images of three biological replicates. (**b**) Hydrogen-Deuterium eXchange (HDX-MS) data showing changes in deuterium exchange along the length of TEX264 when combined with PARP1 for 30 s, 10 mins or 60 mins. The green boxes below highlight potential interacting regions. (**c**) AlphaFold2-predicted structure of TEX264 (AF-Q9Y6I9-F1-v6) used for mapping HDX-MS data. The top panel shows the predicted aligned error (pAE) matrix. The bottom panel displays the structural model coloured according to pLDDT score. (**d**) *In vitro* pulldown of PARP1–GFP combined with TEX264^WT^ (variant 1) or variants including either the C-terminal (2) or N-terminal (3) regions. Variants 1 and 2 display interaction, while variant 3 does not. Representative images of three biological replicates. (**e**) Co-immunoprecipitation of GFP from the chromatin fraction of HeLa cells stably expressing PARP1–GFP after treatment with talazoparib and MMS for 3 h. Cells expressed either TEX264^WT^ or a variant lacking the α-helix region indicated in the schematic (above). The schematic shows the alignment of this variant to sites of interest highlighted by HDX-MS. Representative images of three biological replicates. (**f**) Control conditions for proximity ligation assay between GFP and V5 in cells stably expressing PARP1–GFP and either TEX264-V5 or empty vector (EV)-V5, shown in Fig. 5f. Either or both anti-GFP or anti-V5 antibodies are used as indicated. Scale bar is 10 µm. Representative images of three biological replicates. (**g**) Immunoblot validating the stable expression of TEX264 variants in TEX264^−/−^ CAL51 cells used in Fig. 5j. Representative images of three biological replicates. Source unprocessed blots are available in Source Data. Source data

To further understand the role of TEX264 in this pathway, we examined previously published TEX264 interactomes. In two interactomes, PARP1 was identified as one of the top hits^40,51^. In line with this, TEX264 co-immunoprecipitated from chromatin-bound GFP-tagged PARP1 (Fig. 5a). As expected from previous work^39^, p97 interacted with PARP1 specifically under trapping conditions (Fig. 5a). The interaction between TEX264 and chromatin-bound PARP1 also increased ~40% after treatment (Fig. 5a,b). Depletion of TEX264 reduced the levels of p97, on chromatin, and at trapped PARP1, similar to what has previously been observed with UFD1 depletion^39^ (Fig. 5a,c). This suggests that TEX264 acts as a p97 cofactor to aid p97 recruitment to trapped PARP1. To determine whether PARP1 and TEX264 interact directly, we expressed and purified GFP-PARP1 and TEX264 from an *Escherichia* *coli* expression system and performed an in vitro pulldown assay by isolating PARP1 on PARP1-trap beads. TEX264 directly binds to PARP1, but not to the PARP1-trap beads alone (Extended Data Fig. 5a). To explore this interaction further, we performed hydrogen-deuterium exchange mass spectrometry (HDX-MS), where a difference in deuterium uptake in TEX264 peptides indicates a region of interaction with PARP1 (Supplementary Tables 6 and 7). Multiple regions showed a big difference in deuterium uptake, most prominently in an α-helix immediately following the gyrase inhibitory-like (GyrI-like) domain, and from Ser272 to the C terminus, where the LC3-interacting region (LIR)^51,52^ and p97-interacting SHP motifs^40,99^ are located (Fig. 5d, Extended Data Fig. 5b,c and Supplementary Tables 3 and 4). An in vitro pulldown using either a fragment of TEX264 containing only the GyrI-like domain or the C-terminal half, or a TEX264 fragement containing both regions of interest, demonstrated that only the C-terminal half interacts with PARP1 (Extended Data Fig. 5d). We tested the importance of both regions for TEX264–PARP1 interaction in cells by expressing TEX264 lacking these regions (TEX264–272 and TEX264–Δα-helix) (Fig. 5e and Extended Data Fig. 5e). While loss of the α-helix had no impact on the interaction (Extended Data Fig. 5e), loss of the C terminus from S272 onwards completely abolished the interaction of TEX264 with chromatin-bound PARP1 (Fig. 5e). These findings confirmed that a motif in the extreme C terminus is required for the PARP1–TEX264 interaction.

To visualize the TEX264–PARP1 interaction beyond the chromatin context, we carried out proximity ligation assays (PLAs) between GFP-tagged PARP1 and V5-tagged TEX264 (Fig. 5f and Extended Data Fig. 5f). We were able to visualize this interaction and, consistent with our co-immunoprecipitation experiments, observed that it increased in the nucleus (Fig. 5f,g). The PARP1–TEX264 PLA signal is partially localized around the nuclear periphery, where TEX264 is known to localize due to its transmembrane N-terminal leucine-rich region^40^. Despite the predominantly nuclear localization of PARP1 (ref. ^100^), we observed a marked increase in PLA signal in the cytosol following PARPi treatment (Fig. 5f,g), further supporting our hypothesis that TEX264 acts as a SAR of trapped PARP1.

To explore the role of the LIR and SHP domains of TEX264 in the autophagosomal processing of trapped PARP1, we performed LysoIP in *TEX264*^−/−^ cells (Fig. 5h). Notably, the loss of TEX264 impaired the accumulation of PARP1 in lysosomes under trapping conditions. Complementation of the TEX264-null background with TEX264^WT^ restored lysosomal PARP1 levels. However, mutation of the LIR domain ablated lysosomal PARP1 (Fig. 5h,i). As the LIR is a crucial motif for bridging substrates to LC3 localized in the autophagosomal membrane^101^, this suggests that TEX264 acts as a SAR for trapped PARP1. Similar to p97 inhibition, mutation of the TEX264 SHP domain also impaired PARP1 accumulation in lysosomes under trapping conditions (Fig. 5h,i). Of note, the SHP* and LIR* TEX264 variants were also unable to rescue PARPi sensitivity in *TEX264*^−/−^ cells (Fig. 5j and Extended Data Fig. 5g), further confirming that these two TEX264 functions, SAR and p97 cofactor, work synergistically.

**Extended Data Fig. 6:**
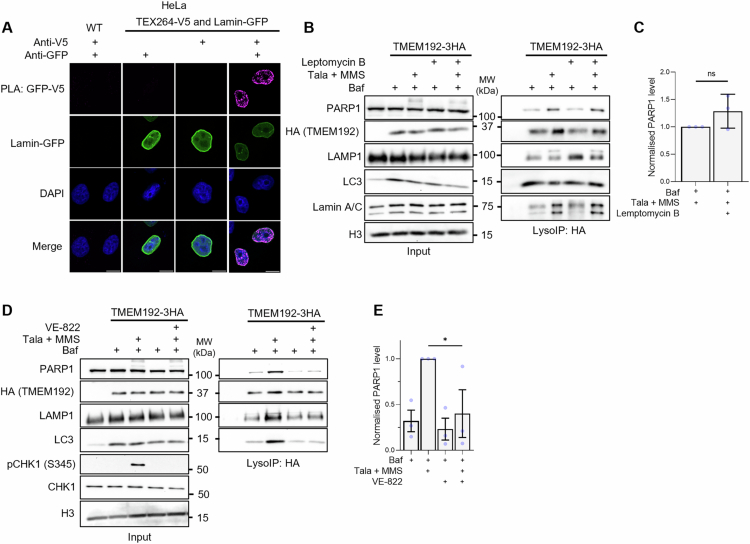
Additional data supporting Fig. 5. (**a**) PLA between GFP and V5 in HeLa cells stably expressing lamin-GFP and transiently expressing TEX264-V5. The PLA reaction was performed with anti-GFP and anti-V5 primary antibodies, where indicated, showing the specificity of the PLA signal for TEX264 and lamin A/C in proximity. Scale bar, 10 µm. (**b**) LysoIP in HeLa cells treated with the indicated drugs for 3 h, including nuclear pore inhibitor leptomycin B (10 nM). (**c**) Quantification of PARP1 levels in (**b**), normalized to treated control. n = 3 independent biological replicates; mean ± s.e.m.; statistical analysis was performed using two-sided unpaired t-test. (**d**) LysoIP in HeLa cells treated with the indicated drugs for 3 h, including ATM inhibitor VE-822 (1 µM). (**e**) Quantification of PARP1 levels in (**d**), normalized to the treated control. n = 3 independent biological replicates; mean ± s.e.m.; statistical analysis was performed using one-way ANOVA with Šídák’s multiple comparisons test; *P* = 0.0258. Source numerical data and unprocessed blots are available in Source Data. Source data

TEX264 localizes to the endoplasmic reticulum (ER) and to both the inner and outer nuclear membranes, as shown by electron microscopy^102^. We confirmed this localization using PLA between lamin A/C–GFP and TEX264–V5 (Extended Data Fig. 6a). However, how PARP1 is exported from the nucleus for autophagosomal processing remains unclear. Lamin A/C, another nucleophagy substrate^103,104^, is phosphorylated by ATR in response to DNA damage, promoting local nuclear envelope rupture^105,106^. Recent work on TEX264-dependent autophagosomal processing of TOP1cc has shown that ATR activity is required for the transport of TOP1 from the nucleus to lysosomes, but that this process does not depend on nuclear pore activity^49^. To determine whether this also applies to trapped PARP1 processing, LysoIP was performed under trapping conditions in cells treated with either ATR inhibitor VE-822 or nuclear pore inhibitor leptomycin B (Extended Data Fig. 6b–e). In line with previous work, leptomycin B did not affect lysosomal PARP1 levels (Extended Data Fig. 6b,c) while ATRi caused a significant reduction (Extended Data Fig. 6d,e). TEX264-dependent processing of TOP1cc was hypothesized to occur through ATR-induced local disruptions of the interphase nuclear membrane, likely via phosphorylation and removal of lamin A/C. Notably, under PARP-trapping conditions, lamin A/C was detected in lysosomes in a leptomycin B-independent manner (Extended Data Fig. 6b,c). This fits the model that trapped PARP1 exits the nucleus through ATR-induced local disruptions in lamin A/C architecture.

### Disruption of the p97–TEX264 autophagy axis causes PARPi-induced replication-associated DNA damage

**Extended Data Fig. 7:**
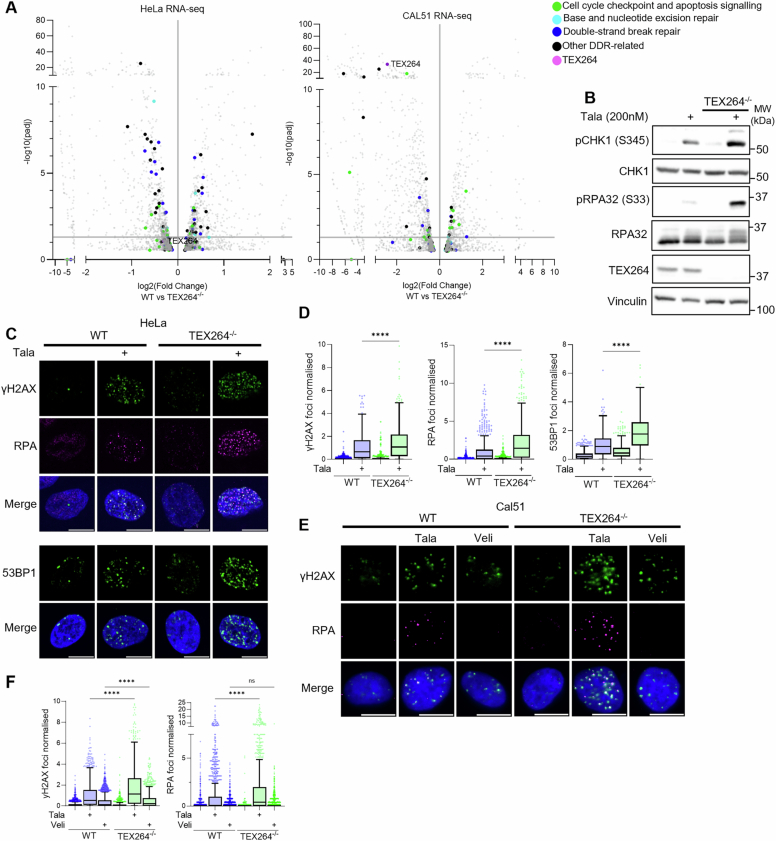
Disruption of TEX264 causes PARPi-induced replication-associated DNA damage. (**a**) Volcano plot showing differential gene expression in HeLa (left) or CAL51 (right) by RNA-seq. Comparison is between TEX264^−/−^ and WT cells after treatment with talazoparib (100 nM, 24 h). Genes relating to DNA damage response are coloured as shown. TEX264 is labelled. Differential gene expression analysis was performed using DESeq2, with *P* values adjusted for multiple testing using the Benjamini–Hochberg false discovery rate (FDR) method. The grey line shows the adjusted *P*-value of 0.05. (**b**) Immunoblotting of replication stress markers in HeLa WT and TEX264^−/−^ cells treated with talazoparib for 24 h. (**c**) RPA, 53BP1 and γH2AX foci in HeLa WT and TEX264^−/−^ cells after 24 hrs talazoparib treatment. Scale bar, 10 µm. (**d**) Quantification of foci in (**c**). Data are from three biological replicates and n = 1,035 (WT utd), n = 826 (WT Tala); n = 874 (TEX264−/− utd) and n = 724 (TEX264−/− Tala) cells. (**e**) γH2AX and RPA foci in CAL51 WT and TEX264^−/−^ cells treated with either talazoparib or veliparib (10 µM) for 24 hrs. Scale bar, 10 µm. (**f**) Quantification of foci in (**e**). Data are from 3 biological repeats and n = 461 (WT utd); n = 356 (WT Tala); n = 294 (WT Veli); n = 297 (TEX264−/− utd); n = 261 (TEX264−/− Tala) and n = 300 (TEX264−/− Veli) cells. Graphs in (**d**) and (**f**) are shown as box-and-whisker plots with median (centre line), interquartile range (box limits, 25th–75th percentiles), and whiskers extending to 1.5× IQR; outliers are shown as individual points. Statistical analyses in (**d**) and (**f**) were performed using one-way ANOVA with Šídák’s multiple comparisons test. Statistical significance was defined as: ns, not significant; *P < 0.05; **P < 0.01; ***P < 0.001; ****P < 0.0001. Exact *P* values, source numerical data and unprocessed blots are available in Source Data. Source data

Having established the role of p97–TEX264-mediated selective autophagy in removing PARPi-induced trapped PARP1, we next sought to explore how its disruption affects cells. Trapped PARP1 causes increased replication stress, DSBs^107–109^ and replication-associated single-stranded DNA gaps^110–112^ suspected to result from collisions between the replication fork and trapped PARP1, as well as PARP1 trapping on unligated Okazaki fragments. Analysis of RNA-seq data comparing WT and *TEX264*^−/−^ cells treated with talazoparib showed significant differential expression of 60 and 37 DDR-related genes in HeLa and CAL51 cells, respectively (Extended Data Fig. 7a and Supplementary Tables 4 and 5). This suggests that TEX264-deficient cells exhibit altered DDR under PARPi treatment. In accordance with this, we observed increased levels of phosphorylated (p) RPA and pCHK1 in *TEX264*^−/−^ cells in response to PARPi (Extended Data Fig. 7b), suggesting increased ATR signalling associated with replication stress. There were also heightened levels of the DDR markers phosphorylated H2AX (γ-H2AX), RPA and 53BP1, with 1.4-, 2.1- and 1.8-fold increases, respectively (Extended Data Fig. 7c,d). The same effect was observed in CAL51 cells (Extended Data Fig. 7e,f), but only in response to talazoparib and not veliparib, supporting that this DNA damage arises in response to accumulated trapped PARP1 when TEX264 fails to clear it from chromatin.

**Extended Data Fig. 8:**
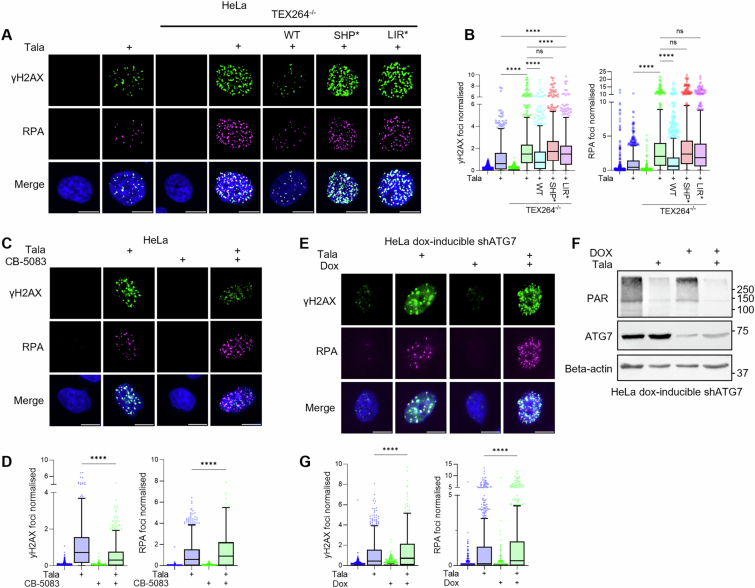
Disruption of p97 causes PARPi-induced replication-associated DNA damage. (**a**) γH2AX and RPA foci in HeLa WT, TEX264^−/−^ or TEX264^−/−^ cells stably expressing indicated TEX264 variants, treated with talazoparib for 24 hrs. (**b**) Quantification of foci in (**a**). Data are from four biological repeats and n = 340 (WT utd); n = 178 (WT Tala); n = 238 (TEX264−/− utd); n = 189 (TEX264−/− Tala); n = 138 (TEX264−/− + WT Tala); n = 138 (TEX264−/− + SHP Tala); and n = 150 (TEX264−/− + LIR Tala) cells. (**c**) γH2AX and RPA foci in HeLa WT cells treated with talazoparib for 24 hrs with or without CB-5083 (10 µM) for the last 4 hrs. (**d**) Quantification of foci in (**c**). Data are from three biological repeats and n = 456 (WT utd); n = 385 (WT Tala); n = 490 (WT CB-5083); and n = 340 (WT CB-5083 + Tala) cells. (**e**) γH2AX and RPA foci in HeLa doxycycline-inducible shATG7 cells treated with talazoparib for 24 hrs with or without doxycycline. (**f**) Immunoblot validating the reduction in PAR and ATG7 levels when doxycycline-inducible shATG7 cells are treated with talazoparib and doxycycline, respectively. (**g**) Quantification of foci in (**e**). Data are from three 3 biological repeats and n = 503 (utd); n = 385 (Tala); n = 584 (Dox utd); and n = 416 (Dox Tala) cells. Graphs in (**b**), (**d**) and (**g**) are shown as box-and-whisker plots with median (centre line), interquartile range (box limits, 25th–75th percentiles), and whiskers extending to 1.5× IQR; outliers are shown as individual points. Statistical analyses in (**b**), (**d**) and (**g**) were performed using one-way ANOVA with Šídák’s multiple comparisons test. Statistical significance was defined as: ns, not significant; *P < 0.05; **P < 0.01; ***P < 0.001; ****P < 0.0001. Exact *P* values, source numerical data and unprocessed blots are available in Source Data. Source data

As the role of TEX264 in repair of trapped PARP1 seems to depend on its dual function as a selective autophagy receptor and p97 cofactor, we next explored how the interruption of these functions affects cellular response to PARPi. In *TEX264*^−/−^ cells complemented with TEX264^WT^, talazoparib-induced RPA and γ-H2AX levels were restored to those observed in WT cells (Extended Data Fig. 8a,b). However, mutation of either the SHP or the LIR domains failed to restore RPA and γ-H2AX levels. We observed the same effect on DNA damage foci when we inhibited p97 with CB-5083 (Extended Data Fig. 8c,d) or autophagy through ATG7 depletion (Extended Data Fig. 8e–g). It is worth noting that we did not detect an increase in PARPi-induced γ-H2AX foci under p97i conditions, as CB-5083 is known to impair ATM kinase activity^113^, the central kinase for phosphorylation of H2AX in response to DNA damage^114^. Altogether, these findings complement our earlier data showing increased sensitivity to talazoparib upon chemical or genetic inhibition of autophagy (Fig. 1d,e).

### Autophagy is essential for clearing PARP1 aggregates induced by trapping

**Fig. 6:**
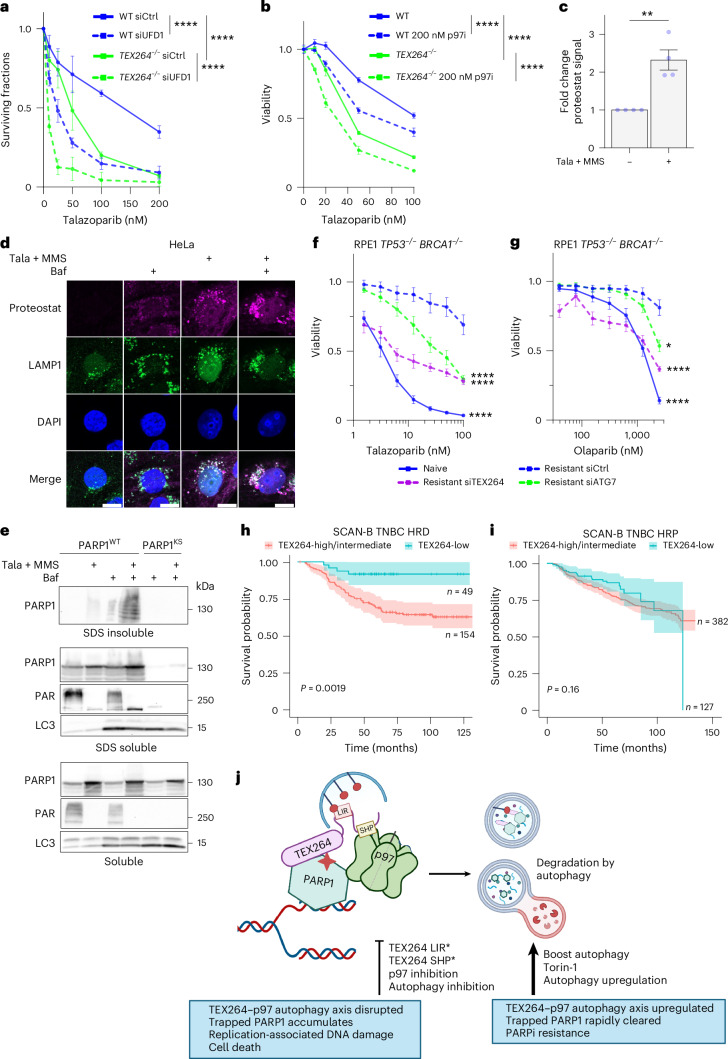
Loss of autophagy-dependent clearance of trapped PARP1 leads to accumulation of cytotoxic PARP1 aggregates and can overcome acquired PARPi resistance. **a**, Colony-formation assay in WT or *TEX264*^−/−^ HeLa cells depleted of UFD1 using siRNA and treated with talazoparib for 24 h. *n* = 3 biological replicates; mean ± s.e.m.; two-way ANOVA. **b**, Cell viability by resazurin assay in WT or *TEX264*^−/−^ HeLa cells treated with talazoparib with or without p97 inhibitor (p97i) CB-5083 (200 nM) for 24 h, followed by 48 h recovery. *n* = 3 biological replicates; mean ± s.e.m.; two-way ANOVA. **c**, Levels of protein aggregates measured by FACS using proteostat dye in cells treated with talazoparib and MMS for 2 h, then talazoparib alone for 18 h. Fold change is compared with the untreated. *n* = 4 biological replicates; mean ± s.e.m.; two-sided unpaired Student’s *t*-test. **d**, As in **c** but with aggregates visualized by immunofluorescence, showing accumulation of aggregates that colocalize with LAMP1 under trapping conditions when combined with bafilomycin A1 treatment. Scale bars, 10 µm. **e**, Fractionation of CAL51 cells stably expressing PARP1^WT^ or PARP1^KS^ into the soluble fraction, SDS-soluble fraction containing chromatin, and SDS-insoluble fraction containing protein aggregates. Immunoblotting determined PARP1 levels in each fraction. **f**,**g**, Cell viability measured by resazurin assay in RPE1 *TP53*^−/−^hTERT *BRCA1*^−/−^ cells, either naive or resistant to olaparib. Resistant cells are depleted by siRNA of TEX264 (siTEX264), ATG7 (siRNA) or a luciferase control (siCtrl). Treatment is with talazoparib (**f**) or olaparib (**g**) for 6 days. Twelve technical replicates across four biological replicates; mean ± s.e.m.; two-way ANOVA. **h**,**i**, Kaplan–Meier plots of SCAN-B TNBC (*n* = 712) in HRD (**h**) and HRP (**i**) tumours (*n* = 203 and 509, respectively). Homologous recombination status was annotated using a 228-gene expression signature^128^ and compared against TNBC from The Cancer Genome Atlas (TCGA)-BRCA (*n* = 312) with known HRD status. *P* values were calculated using a log-rank test. **j**, Model of the TEX264–p97–autophagy axis in trapped PARP1 repair. Inhibition of this process leads to cell death, whereas boosting this pathway results in cells developing resistance to PARPi. *P ≤ 0.05; **P ≤ 0.01; ***P ≤ 0.001; ****P ≤ 0.0001. Exact *P* values, source numerical data and unprocessed blots are available in Source Data. Source data

**Extended Data Fig. 9:**
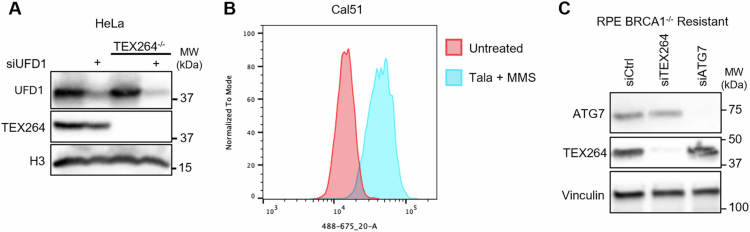
Aggregates formation by PARPi. (**a**) Immunoblot validating depletion of UFD1 and TEX264 with siRNA for experiments in Fig. 6a and b. Representative image of three biological replicates (**b**) A representative FACS plot from experiments in Fig. 6c showing the increase in proteostat signal in cells treated with talazoparib and MMS for 2 hours, followed by talazoparib for 18 hrs. (**c**) Immunoblot validating depletion of ATG7 and TEX264 with siRNA for experiments in Fig. 6f and g. Representative image of three biological replicates. FACS gating strategy, source numerical data and unprocessed blots are available in Source Data. Source data

Cells exhibit increased replication stress and DNA damage when trapped PARP1 is not efficiently cleared by TEX264-mediated autophagy. Previous studies have shown that p97, through its UFD1 cofactor, removes trapped PARP1 from chromatin via a proteasome-dependent pathway^39^. This raises the question of why the newly identified p97–TEX264-autophagy pathway is also important for cell survival. Notably, disruption of the p97–UFD1 pathway, either by UFD1 depletion or by CB-5083 treatment (p97i), further increased the sensitivity of *TEX264*^−/−^ cells to talazoparib (Fig. 6a, b and Extended Data Fig. 9a), supporting the notion that the p97-UFD1-proteasome pathway operates in parallel with the p97–TEX264-autophagy pathway. Moreover, this suggests that TEX264 may also have a role in processing trapped PARP independently of p97 (Fig. 6b). This also highlights that the clearance of PARP1 from chromatin by p97 alone is insufficient to promote cell survival. One of the key functions of autophagy is the clearance of protein aggregates^115^, and misfolded p97 substrates are thought to accumulate as aggregates^116,117^ if not properly processed. In fact, camptothecin, a TOP1 inhibitor, was previously shown to induce TOP1 aggregation at doses at which clearance of TOP1cc depends on autophagy^49^. Using a dye to stain aggregated proteins, ProteoStat, we confirmed by both FACS and immunofluorescence that aggregates accumulated during sustained talazoparib treatment (Fig. 6c,d and Extended Data Fig. 9b). Aggregate accumulation persisted when autophagy was inhibited with bafilomycin A1, and these aggregates colocalized with LAMP1 (Fig. 6d), supporting the conclusion that PARPi-induced aggregates are degraded by autophagy. Purification of insoluble aggregates showed that they contained high levels of PARP1, and that aggregated PARP1 increased substantially when talazoparib treatment was combined with bafilomycin A1 (Fig. 6e). Of note, PARP1 aggregates did not form in cells expressing PARP1^KS^, a variant that cannot bind DNA/chromatin (Fig. 6e). Overall, these findings indicate that autophagosomal processing of trapped PARP1 is key to preventing the accumulation of cytotoxic PARP1 aggregates and that PARP1 aggregation depends on PARP1 binding/trapping to chromatin.

### The p97–TEX264 autophagy axis is relevant to PARPi resistance

**Extended Data Fig. 10:**
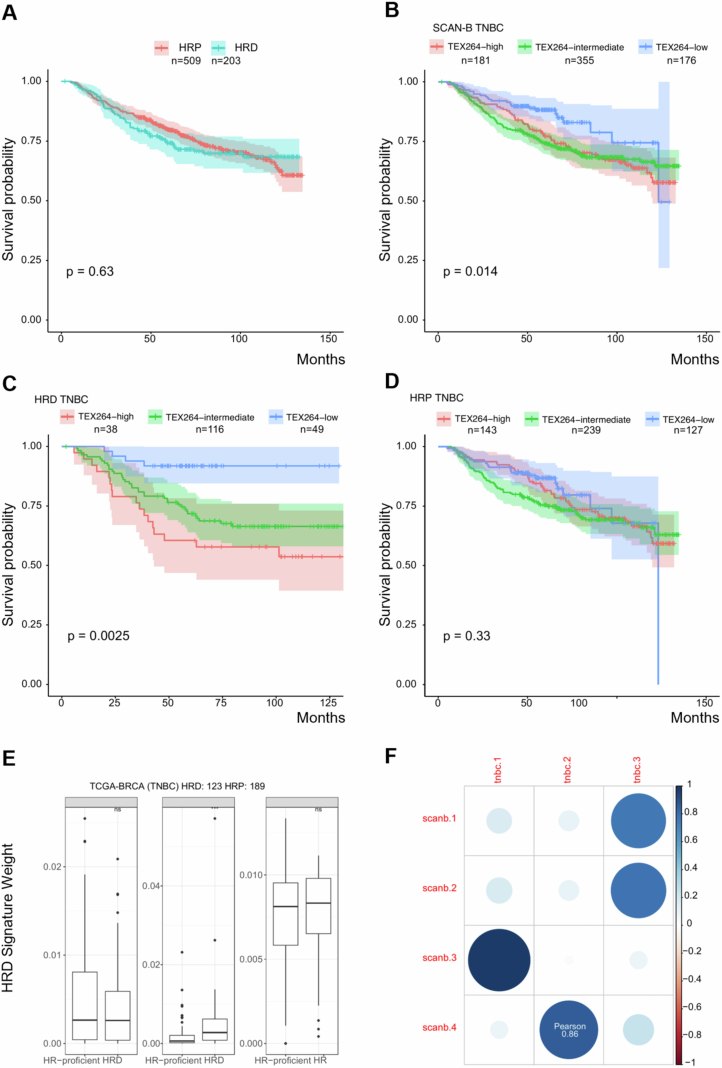
The p97–TEX264 autophagy axis is relevant to PARPi resistance. (**a**) Kaplan–Meier plot (with 95% Confidence interval) of homologous recombination repair-deficient (HRD) compared with homologous recombination repair-proficient (HRP) TNBC from the SCAN-B cohort. (**b**) Kaplan–Meier plot of SCAN-B TNBC by *TEX264* expression based on the highest, lowest and intermediate expression. (**c**) Kaplan–Meier plot of HRD TNBC based on *TEX264* expression. (**d**) Kaplan–Meier plot of HRP TNBC based on *TEX264* expression. (**e**) HRD signature weights in the TCGA-BRCA TNBC with tnbc.2 correlated with HRD status. Graph is shown as box-and-whisker plots with median (centre line), interquartile range (box limits, 25th–75th percentiles), and whiskers extending to 1.5× IQR; outliers are shown as individual points. Statistical analyses by two-sided Wilcoxon test (*P* = 0.00053). (**f**) Correlation matrix of extracted HRD gene expression signatures of the SCAN-B cohort and TCGA-BRCA with scan-b.4 matching the HRD signature (tnbc. 2) identified in the TCGA-BRCA cohort.

PARPi resistance is a major clinical concern. To explore whether TEX264-mediated selective autophagy (nucleophagy) of trapped PARP1 may be relevant in the context of PARPi resistance, we used RPE1 *TP53*^−/−^ hTERT *BRCA1*^−/−^ cells that had acquired resistance to olaparib following prolonged exposure. When compared with olaparib-naive cells, we observed a resistance to both talazoparib (Fig. 6f) and olaparib (Fig. 6g), as expected. Of note, depletion of either TEX264 or ATG7 in resistant cells considerably re-sensitized them to both PARPi, with the strongest effect observed with the more potent trapper talazoparib (Fig. 6f,g and Extended Data Fig. 9c). This suggests that impairing the clearance of trapped PARP1 by TEX264-mediated selective autophagy could partially overcome PARPi resistance. Given that the TEX264–nucleophagy axis is important for genome stability and cell survival in response to trapped PARP1, and that BRCA1-deleted yet PARPi-resistant cells are hypersensitive to TEX264 inactivation, we asked whether this pathway has clinical relevance. We analysed RNA-seq data from the SCAN-B (Sweden Cancerome Analysis Network – Breast)^118^ cohort of 7,743 patients with breast cancer to examine the relationship between TEX624 expression and the homologous recombination status of these cancers. We focused on TNBC as previous reports have observed that survival trends and homologous recombination status differ between TNBC and HER2^+^ or ER^+^ subtypes^118,119^. Although there were no overall survival differences between patients with homologous recombination-proficient (HRP) and homologous recombination-deficient (HRD) TNBC breast cancer (Extended Data Fig. 10a), we observed a strong and significant survival difference in patients with HRD related to TEX264 expression (Fig. 6h and Extended Data Fig. 10b,c), which was absent in patients with HRP (Fig. 6i and Extended Data Fig. 10d). Patients with HRD breast cancer with low TEX264 messenger RNA expression showed approximately 28% better long-term survival, for example, at 10 years. These clinical correlational data support our findings that a functional TEX264-mediated nucleophagy pathway (for example, high TEX264 expression) promotes breast cancer cell survival and is consequently associated with reduced overall patient survival (Fig. 6j).

## Discussion

Understanding how trapped PARP1 is regulated is key to elucidating resistance. We identify a direct role for selective autophagy in response to PARPi (Fig. 6j). The selective autophagy receptor TEX264 works with LC3 and the ATPase p97 to process trapped PARP1 from chromatin and deliver it to lysosomes. This depends on SUMOylation and ubiquitination, but not on the SUMO-dependent E3 ligase RNF4, previously implicated in proteasomal degradation of trapped PARP1 (ref. ^39^). Mutations disrupting TEX264 interaction with LC3 (LIR) or p97 (SHP) impair PARP1 delivery to lysosomes, increase PARPi-induced DNA damage and sensitize cells to talazoparib, indicating that TEX264 functions as both an autophagy receptor and a p97 cofactor.

TEX264-driven selective autophagy of PARP1 is tightly coupled to PARPi-induced PARP1 trapping and aggregate formation, enabling lysosomal translocation (Fig. 2b–d). A PARP1 mutant unable to bind chromatin does not form aggregates, cannot be delivered to lysosomes and fails to kill cancer cells in *TEX264*^−/−^ cells (Fig. 4g, 6e and Extended Data Fig. 3a and b).

Other substrates of nucleophagy, such as lamin B1 and lamin A/C, interact with LC3 and are degraded by autophagy in response to oncogenic or genotoxic stress, inducing senescence^103,104^. Similarly, TOP2-DPCs and damaged DNA are detected in nuclear buds after etoposide treatment in an autophagy-dependent manner^120^, underscoring the role of selective autophagy in processing nuclear material under genotoxic stress. Our work on TOP1cc shows that mammalian cells use the p97–TEX264 system to remove DNA lesions to lysosomes^49^. We therefore propose that TEX264-mediated selective autophagy of DNA lesions (nucleophagy) is a specialized DNA repair pathway for tightly bound chromatin protein lesions prone to aggregation, such as TOP1cc and trapped PARP1 (Fig. 6d,e).

For both TOP1cc^49^ and trapped PARP1, autophagic processing of DNA lesions is independent of the nuclear pore (Extended Data Fig. 6b,c). Although the mechanism of nuclear envelope transit is not fully understood, dynamins promote nuclear envelope remodelling and budding of vesicles containing damaged DNA for autophagic clearance^121^. Consistent with this, we observed nuclear envelope budding in talazoparib- and MMS-treated cells, where trapped PARP1 exits the nucleus and merges with lysosomes (Fig. 2i,j, Supplementary Videos 1 and 2 and Extended Data Fig. 3f,g). Similar mechanisms have been reported in yeast, where a dynamin 1-like protein mediates the removal of nuclear and inner nuclear membrane cargos to lysosomes^122^. Alternatively, the autophagy process we identified could represent microautophagy^123^, in which lysosomes would directly engulf PARP1 without its previous sequestration in an autophagosome. Visualization of the phagophore by electron microscopy surrounding trapped PARP1, would clarify whether trapped PARP1 is processed by the selective autophagy (nucleophagy) pathway we propose or by microautophagy.

Autophagy has context-dependent roles in cancer^55^, and its function in the PARPi setting is similarly debated^57^. Our data, together with most literature^56–63^, indicate that PARPi induces autophagy as a protective mechanism (Fig. 1). PARPi generates reactive oxygen species (ROS) and upregulates PTEN, a negative regulator of mTOR, thereby enhancing autophagy, which clears ROS and whose inhibition sensitizes cells to olaparib through ROS accumulation. Increased autophagy also promotes homologous recombination, increasing BRCA1 and RAD51 recruitment to PARPi-induced lesions^87,124^. Although we do not define a new mechanism of PARPi-induced autophagy, we reveal a direct cytoprotective role of nucleophagy in processing trapped PARP1.

Pre-clinical studies in cell lines and organoids show that combining PARPi and autophagy inhibitors is a promising strategy^57^, and autophagy upregulation is associated with olaparib resistance in multiple cancer types, where its inhibition re-sensitizes resistant cells^63,125^. However, targeting autophagy is challenging due to its pleiotropic effects during tumourigenesis, metastasis and within the tumour microenvironment, reflected in mixed clinical responses to chloroquine derivatives^126,127^.

In our study, cells rendered resistant by chronic olaparib treatment were re-sensitized to PARPi by TEX264 or ATG7 knockdown (Fig. 6f,g), suggesting that specifically inhibiting nucleophagy of trapped PARP1 may overcome resistance while limiting off-target effects of broader autophagy inhibitors. Moreover, analysis of clinical data indicates that TEX264 expression significantly influences overall survival in patients with HRD breast cancer (Fig. 6h,i). These findings highlight the importance of TEX264-mediated nucleophagy in maintaining genome stability and its potential utility for predicting therapeutic responses and overcoming acquired PARPi resistance.

## Methods

Most unique materials, if not commercially available, are readily available from the authors upon reasonable request.

A full list of materials used is provided in Supplementary Table 8.

### Ethical statement

This research complies with all ethical regulations.

### Cell culture, transfection and drug treatment

CAL51 (DSMZ, ACC 302), HeLa (ATCC, CCL-2), MDA-MB231 (ATCC, Htb-26) and RPE *TP53*^−/−^ hTERT *BRCA1*^−/−^ were maintained in Dulbecco’s modified Eagle medium (DMEM), supplemented with 10% fetal bovine serum and 1× penicillin–streptomycin (Sigma-Aldrich). Cells were tested for *Mycoplasma* regularly. Cells were transfected with siRNA using Lipofectamine RNAiMAX according to the manufacturer’s instructions, with experiments carried out 72 h after transfection. Transient transfection with plasmids was carried out using FuGENE HD Transfection reagent for microscopy-based experiments and polyethyleneimine (PEI) transfection reagent for all other experiments, with both used according to the manufacturer’s instructions. Drug treatments were as indicated in the figure legends, with a vehicle control used where appropriate. Talazoparib was used at 200 nM, MMS at 0.01% and bafilomycin A1 at 50 nM unless otherwise stated.

### Generation of plasmids and stable cell lines

To generate pLX313-V5_EV, two overlapping oligos were ligated into a pLX313 backbone cleaved at the EcoRV/NHeI restriction digest sites to insert a STOP codon after the V5 tag. Ligation was performed using NEBuilder HiFi DNA Assembly, then transformed into NEB stable competent *E.* *coli* cells. Successfully transformed colonies were identified by colony PCR and sequenced by Source BioScience. pLX313-TEX264-V5 variant plasmids were generated similarly, using the oligonucleotides listed in the Supplementary Table 8. TEX264 fragments were PCR amplified using Q5 High-Fidelity DNA polymerase. Full-length PARP1 was cloned into NcoI and BamHI restriction sites in the pNIC28 vector in a similar way. TEX264 (34-313; 34-185; 186-313) carrying a TEV cleavage sequence on the 5′-end was cloned by the Phanta Flash Super-Fidelity DNA Polymerase. The fragment was then inserted into the pCold-I vector at the NdeI site using the ClonExpress Ultra One Step Cloning kit V2. pmCherry–PARP1–eGFP was generated by the Genome Engineering and Transgenics facility at the MRC Wetherall Institute of Molecular Medicine. PARP1 complementary DNA was amplified from an existing plasmid and cloned by InFusion (Takara) into pmCherry–eGFP (Addgene, #86639) at AgeI restriction sites. InFusion reactions were transformed into Stable2 competent *E.* *coli*, and correct colonies were identified by AgeI digestion and single-molecule sequencing (Oxford Nanopore).

CAL51 *TEX264*^−/−^ cells were created by CRISPR–Cas9 knockout of TEX264 using TEX264 CRISPR–Cas9 KO Plasmid (h) and TEX264 HDR Plasmid (h). Plasmids were transfected into CAL51 cells using Fugene, and then the transfected cells were selected with 2 µg ml^−1^ puromycin. Single-cell colonies were isolated by limiting dilution and validated for TEX264 loss by western blot. Cells were induced to stably express TMEM192–3HA, TEX264^WT^–V5, TEX264^SHP*^–V5, TEX264^LIR*^–V5 or eEV–V5 using lentiviral transduction with the following plasmids: pLJC5-Tmem192-3xHA (Addgene, 02930), pLX313-TEX264–V5, pLX313-TEX264-SHP*–V5, pLX313-TEX264-LIR*–V5, pLX313-V5_EV–V5. Lentiviral particles were generated in HEK293T cells by transfection with transfer plasmid for gene of interest, pAmphoR envelope plasmid and Δ8.2R packaging plasmid (both a gift from V. D’Angiolella), using PEI transfection. After 72 h, viral particles were collected, filtered and added to CAL51 PARP1^WT^–GFP, CAL51 PARP1^KS^–GFP, CAL51 *TEX264*^−/−^, HeLa *TEX264*^−/−^ cells, as required, with 16 µg ml^−1^ Polybrene. Puromycin or hygromycin was used to isolate TEX264 or TMEM192–3HA transduced cells, respectively, before isolating and expanding single-cell colonies by limiting dilution. Colonies were screened by western blot and immunofluorescence to select cell lines that were stably expressing TEX264 variants at a similar level to the endogenous.

Human retinal pigment epithelial RPE1 *BRCA1*^−/−^ cells transduced with hTERT and TP53-deleted^129^ were cultivated in monolayers in DMEM supplemented with 10% fetal bovine serum in the presence of 2 μg ml^−1^ blasticidin. To generate PARP inhibitor-resistant cells, BRCA1^−/−^ RPE1 cells were grown in the presence of increasing doses of olaparib for 3 months. In brief, cells were seeded to 50% confluency and initially treated with 20 nM olaparib. Cells were allowed to grow to 80–90% confluency, while fresh medium supplemented with the drug was replaced every 2–3 days. The concentration of olaparib was increased by 25% each time the cells were passaged to a final concentration of 76.25 nM. Viability assays were conducted to confirm resistance to PARP inhibitors.

The sources of previously generated plasmids and cell lines are listed in Supplementary Table 8.

### Purification of recombinant proteins

#### TEX264 purification

All the TEX264 proteins were expressed by the pCold cold shock system. *E.* *coli* Rosetta (DE3) strain carrying the corresponding plasmid was grown in LB medium at 37 °C with constant shaking until OD600 reached ∼0.8. The culture was cold-shocked at 15 °C for 30 min without shaking. It was induced by IPTG at a final concentration of 0.5 mM at 15 °C for 24 h. The collected cell pellet was resuspended in Eq Buffer (50 mM Tris-HCl, pH 7.4, 150 mM NaCl and 0.5 mM TCEP–HCl) containing 0.2 mM phenylmethyl sulfonyl fluoride (PMSF) and one tablet EDTA-free protease inhibitor cocktail (Roche) and then processed by sonication. The lysate was centrifuged at 20,000 rpm for 30 min.

To purify His–TEX264 (34-313), the supernatant was loaded onto a 5-ml HiTrap Q FF column (Cytiva, 17515601) pre-equilibrated with Eq Buffer. A linear 8 CV gradient of 150 mM to 1 M NaCl was performed. TEX264 was eluted at a relatively lower salt concentration. Purer fractions were concentrated for the final SEC purification via a HiLoad 16/600 Superdex 200 pg column (Cytiva, 28-9893-35) with the SEC Buffer (50 mM HEPES, pH 7.4, 150 mM NaCl and 0.5 mM TCEP–HCl). His–TEX264-Gyrl (34-185) was purified similarly, except that the Eq Buffer was adjusted to pH 8.5. A linear 8 CV gradient of 50 mM to 1 M NaCl was performed. Purer fractions were concentrated for the SEC purification via a HiLoad 16/600 Superdex 75-pg column (Cytiva, 28-9893-33) with the same SEC Buffer. It was further concentrated as the final product.

His–TEX264–Cter (186-313) was purified by a one-step Ni-affinity chromatography. In brief, the collected cell pellet was resuspended in His-Eq Buffer (50 mM Tris-HCl, pH 7.4, 300 mM NaCl and 0.5 mM TCEP–HCl) containing 0.2 mM PMSF, 30 mM imidazole and one tablet of EDTA-free protease inhibitor cocktail (Roche) and then processed by sonication. The lysate was centrifuged at 20,000 rpm for 30 min. The supernatant from centrifugation was then loaded onto a 10-ml HisTrap HP column (Cytiva, 17524802). His–TEX264-Cter was eluted with His-Eq Buffer containing 300 mM imidazole. It was further concentrated as the final product.

#### PARP1 purification

The *E.* *coli* Rosetta (DE3) strain carrying the pNIC28–PARP1 plasmid was grown in LB medium at 37 °C with constant shaking until OD_600_ reached ∼1.0. The culture was induced by IPTG at a final concentration of 0.5 mM at 20 °C overnight. The collected cell pellet was resuspended in His-Eq Buffer (50 mM Tris-HCl, pH 7.4, 300 mM NaCl and 0.5 mM TCEP–HCl) containing 0.2 mM PMSF, 30 mM imidazole and one tablet of EDTA-free protease inhibitor cocktail (Roche) and then processed by sonication. The lysate was centrifuged at 20,000 rpm for 30 min. The supernatant from centrifugation was then loaded onto a 15-ml HisTrap HP column (Cytiva). His–PARP1 was eluted with His-Eq Buffer containing 300 mM imidazole. The elution was further concentrated for the SEC purification via a HiLoad 16/600 Superdex 200-pg column (Cytiva) with the SEC Buffer (50 mM HEPES, pH 7.4, 150 mM NaCl and 0.5 mM TCEP–HCl). It was further concentrated as the final product.

### Immunofluorescence

Immunofluorescence was carried out as previously described^39^ with detergent pre-extraction. Cells were seeded and grown on glass coverslips to 70–90% confluency. After one wash with PBS, pre-extraction buffer (25 mM HEPES, pH 7.5, 50 mM NaCl, 1 mM EDTA, 3 mM MgCl_2_, 300 mM sucrose and 0.5% (v/v) Triton X-100) was added on ice for 2–2.5 min. Cells were washed once with the same buffer without Triton X-100 before fixing for 15 min on ice with 4% formaldehyde in PBS. Coverslips were blocked with 5% BSA for 1 h at 37 °C, then sequentially incubated with antibodies diluted in 2.5% BSA for 1 h at room temperature. Antibodies used were anti-PARP1, anti-γH2AX, anti-RPA, anti-53BP1, donkey anti-mouse Alexa Fluor 555 and donkey anti-rabbit Alexa Fluor 488. Images were acquired using either the Andor Dragonfly confocal or Nikon Ni-E widefield microscopes and analysed with custom CellProfiler pipelines. For live-cell imaging, cells were transfected with Lipofectamine 3000 (L3000001, Invitrogen) to express the mCherry–PARP1–GFP reporter fusion protein, and were seeded in 35-mm glass-bottom dishes (FD35-100, WPI). LysoView680 (70086, Biotium) was added 30 min before beginning the live imaging assay. Cells were imaged in FluoroBrite DMEM (A1896701, Gibco) to reduce background fluorescence. Confocal images were captured on an Olympus SpinSR SoRa spinning disc confocal microscope using a 50-µm pinhole. A ×60/1.30 NA lens was used, and images were obtained using a Hamamatsu ORCA-Fusion camera. The 3.2-µm *z*-stacks were captured using a z-spacing of 0.4 µm, over a 1-h time course at 30-s intervals. The images obtained have a 2,048 × 2,048-pixel resolution, with a pixel size of 0.107676 µm. Maximum intensity projections (MIPs) were generated using CellSens software. Post-acquisition image processing and analysis were performed using ImageJ/Fiji, and renderings were created in Imaris (Oxford Instruments).

### Western blot

Standard protocols were used for SDS–PAGE and subsequent immunoblotting using either 0.22-μm pore size PVDF (Bio-Rad) or nitrocellulose (GE Healthcare) membranes to transfer proteins from homemade polyacrylamide gels.

### Cell survival assays

For colony-formation assays, cells were seeded in six-well plates at 1,000 cells per well for WT and 1,500 cells per well for TEX264^−/−^ cells. After 16 h, cells were treated for 24 h, then allowed to grow in recovery medium for 6–10 days until colonies were 20–50 cells in diameter. Wells are washed with PBS, then fixed in 100% methanol for 10 min before staining in crystal violet (1.23 mM crystal violet, 1% formaldehyde, 1% methanol and 1× PBS). Colonies were scanned and counted using GelCount (Oxford Optronix). For resazurin assays, 500–1,000 cells per well were seeded in black-well flat-bottom 96-well plates. The following day, treatment was added for the time described in the figure legends. After treatment was complete, the medium was replaced with fresh medium containing 30 µg ml^−1^ resazurin for 4–6 h. Resazurin medium was added to three empty wells to serve as a blank control. Fluorescence was measured at 570 nm using a plate reader. The average fluorescence detected in the blank sample was subtracted from each reading to normalize for background signal. For both assays, all conditions were in technical triplicate. Resazurin assays were more commonly used when experiments required depletion by RNAi, as this often affected colony formation. The shorter time course of resazurin assays was also more suited to depletion-based experiments, as low protein levels could be maintained more reliably than in longer colony-formation assays.

### Chromatin fractionation and co-immunoprecipitation

Chromatin fractionation and co-immunoprecipitation were performed as previously described^39^. In brief, sub-confluent cells were collected in PBS containing 3 mM EDTA. The nuclear pellet was isolated by lysis in buffer A (10 mM HEPES, pH 7.45, 10 mM KCl, 340 mM sucrose, 3 mM EDTA, 10% glycerol, protease and phosphatase inhibitors, NEM and 0.1% Triton X-100), then chromatin was isolated in buffer B (3 mM EDTA, 0.2 mM EGTA, 5 mM HEPES, pH 7.9, protease and phosphatase inhibitors and NEM). Soluble chromatin was recovered by digestion in Benzonase buffer (50 mM Tris-HCl, pH 7.9, 100 mM NaCl, 10 mM MgCl_2_ and 125 U ml^−1^ benzonase). An input sample was taken, and 50 µg ml^−1^ ethidium bromide was added to the remainder. PARP1–GFP was captured from the soluble chromatin fraction on GFP-trap beads, previously blocked in 5% BSA. Beads were washed three times for 15 min with IP wash buffer (50 mM Tris-HCl, pH 7.4, 150 mM NaCl, 0.5 mM EDTA, 0.05% Triton X-100, protease and phosphatase inhibitors and NEM), before elution with Laemmli buffer.

### Biochemical isolation of aggregates

The isolation of aggregates was performed as previously described^49^. In brief, CAL51 cells were treated for 1 h with talazoparib and MMS, followed by talazoparib with or without bafilomycin A1 for 18 h. Cells were collected and lysed with Benzonase to collect the soluble fraction. The pellet was washed with a buffer containing 1.5% SDS to collect the SDS-soluble fraction. The SDS insoluble fraction was solubilized with 100% formic acid and sonication, before evaporating the formic acid and resuspending the pellet in Laemmli buffer for running by western blot.

### Proximity ligation assay

PLA was performed using the Duolink In Situ PLA kits, following the manufacturer’s protocol. Either CAL51 cells stably expressing both PARP1–GFP and TEX264–V5, or HeLa cells expressing lamin A/C–GFP stably and TEX264–V5 transiently, were seeded and grown on glass coverslips to 70–90% confluency. After treatment, cells were fixed with 4% formaldehyde in PBS for 10 min, then permeabilized with 0.25% Triton X-100 for 10 min. After three washes in buffer A (150 mM NaCl, 10 mM Tris, pH 7.4 and 0.05% Tween 20), cells were blocked in the provided blocking reagent. Further washes in buffer A were followed by incubation with anti-GFP and anti-V5 primary antibodies (1:500 dilution). Coverslips were then incubated with PLA PLUS and MINUS probes. Cells were incubated with ligase followed by polymerase, with washes in buffer A between each step. Coverslips were then washed twice with wash buffer B (100 mM NaCl and 250 mM Tris, pH 7.5) before staining with DAPI (1:1,000 dilution) for 10 min. Finally, coverslips were washed twice with buffer A and once with 0.01× buffer B before mounting using ProLong Glass Antifade Mountant. Images were acquired using a TCS SP8 laser scanning confocal microscope (Leica). A ×63 1.2 NA water immersion objective lens was used for acquisition, with the confocal pinhole size set to 111.4 μm. Images were scanned at 2,048 × 2,048, with a pixel size of 90 nm. Analysis was carried out using ImageJ and a bespoke pipeline on CellProfiler.

### Immunoprecipitation of intact lysosomes

LysoIP was performed as previously described^88^. In brief, cells expressing TMEM192 were collected and washed in KPBS (136 mM KCl and 10 mM KH_2_PO_4_, adjusted pH 7.25 with KOH) before an input sample was taken. Cells were homogenized with 15 strokes in a Dounce homogenizer, then centrifuged at 1,000*g* for 2 min. Supernatant containing organelles, including lysosomes, was loaded onto anti-HA magnetic beads and incubated for 15 min on a rotating wheel at 4 °C. Beads were washed five times in KPBS, then eluted in Laemmli for analysis by western blotting. Quantification of band intensity was performed using ImageJ. PARP1 levels in the LysoIP fraction were normalized by dividing by the HA level in the LysoIP fraction. Each set of biological replicates was then normalized to a single reference condition to express PARP1 levels as fold-change values.

### mCherry–PARP1–GFP assay

For fixed imaging, cells transfected with mCherry–PARP1–GFP were seeded on glass coverslips, as for immunofluorescence. After treatment, coverslips were washed once in PBS and fixed with 4% formaldehyde in PBS for 15 min, then washed three times with 0.01% BSA in PBS, before incubation with 4,6-diamidino-2-phenylindole (DAPI) for 30 min in the dark. After a further three washes, coverslips were mounted on glass slides using Invitrogen ProLong Glass Antifade Mountant. Images were acquired using a TCS SP8 laser scanning confocal microscope (Leica), as described for PLAs, and analysed using ImageJ. Quantification was performed manually by observing the number of red cytosolic puncta per cell, with only puncta in proximity to the nucleus counted.

For live-cell imaging, cells transfected with Lipofectamine 3000 (L3000001, Invitrogen) to express the mCherry–PARP1–GFP reporter fusion protein were seeded in 35-mm glass-bottom dishes (FD35-100, WPI). LysoView680 (70086, Biotium) was added 30 min before beginning the live imaging assay. Cells were imaged in FluoroBrite DMEM (A1896701, Gibco) to reduce background fluorescence. Confocal images were captured on an Olympus SpinSR SoRa spinning disc confocal microscope using 50 µm pinhole. A ×60/1.3 0NA lens was used, and images were obtained using a Hamamatsu ORCA-Fusion camera. The 3.2-µm *z*-stacks were captured using a *z*-spacing of 0.4 µm over a 1-h time course at 30-s intervals. The images obtained have a 2,048 × 2,048-pixel resolution, with a pixel size of 0.107676 µm. MIPs were generated using the CellSens software. Post-acquisition image processing and analysis were performed using ImageJ/Fiji, and renderings were created in Imaris. Further details on image acquisition and processing are provided in Supplementary Table 9.

### Proteostat aggregates assay

For FACS, cells in six-well plates were treated with talazoparib and MMS for 2 h, followed by treatment with talazoparib only for 18 h. After collecting with trypsin, cells were washed in PBS, then the cell pellet was resuspended in 200 µl PBS. This was added dropwise into 1 ml of 4% formaldehyde with slow vortexing. After 30 min of fixation at room temperature and one wash with PBS, cells were resuspended and added as for fixation into 1 ml of permeabilization buffer (0.5% Triton X-100 and 3 mM EDTA, pH 8.0) in PBS. After 30 min on ice, cells were washed with PBS. Cells were transferred through the cell strainer cap of a FACS tube (352235, Corning) to remove debris, then centrifuged at 800*g* for 10 min. The cell pellet was resuspended in 500 µl of PROTEOSTAT Aggresome Red Detection Reagent diluted 2,500-fold in 1× Assay Buffer and incubated for 30 min in the dark. Samples were analysed in the FL3 channel of an LSR Fortessa. Immunofluorescence of aggregates was carried out in a very similar way to standard immunofluorescence, with the proteostat dye applied to coverslips after fixation and permeabilization.

### In vitro immunoprecipitation

The purification of required proteins is described in detail above. PARP1-trap beads were washed in permissive buffer (50 mM HEPES, pH 7.4, 500 mM NaCl, 0.01% Triton X-100 and 1 mM TCEP–HCl), followed by blocking in the same buffer containing 5% BSA for 1 h. After two further washes in permissive buffer, PARP1 was loaded in 150 µl permissive buffer to a concentration of 2.5 µM and incubated with rotation for 1 h at 4 °C. Beads were washed three times with permissive buffer, followed by one wash with a more stringent buffer containing 0.1% Triton X-100. TEX264 was incubated with blocked beads in parallel to remove proteins prone to non-specific binding. TEX264 in the supernatant was added to PARP1-bound beads in 150 µl permissive buffer to a concentration of 2.5 µM, and an input sample was collected before incubating with rotation for 2 h at 4 °C. Beads were washed twice with permissive buffer, then three times with stringent buffer before resuspending in Laemmli buffer containing 100 mM dithiothreitol (DTT) for analysis by western blot.

### Hydrogen-deuterium exchange mass spectrometry

#### Sample preparation

We designed HDX-MS experiments to map the PARP1 interaction interface on TEX264, while also obtaining structural and mechanistic insights into the complex formation itself. TEX264 protein stocks were provided at 20 μM in 50 mM TRIS and 10 mM reduced glutathione. PARP1 protein stocks were provided at 19 μM in 50 mM HEPES, 150 mM NaCl and 0.5 mM TCEP. TEX264 was incubated with (holo-state) and without (apo-state) PARP1 for 60 min at room temperature to enable the complete formation of the complex. A molar ratio of 3:1 (PARP1:TEX264) was used. Samples were diluted over the course of the labelling experiment to achieve a final TEX264 concentration of 20 pmol on the column.

#### Data acquisition

We implemented an HDX-MS strategy similar to that, which we recently described^130^. In brief, we prepared a deuterium oxide D_2_O (99+ %D, Cambridge Isotope Laboratories) labelling buffer supplemented with identical buffer conditions to those of the protein stocks. The pH was corrected to pD 7.42 (pD = pH + 0.4). A quenching buffer of 0.8% formic acid, pH 1.08 in H_2_O, was prepared. For the labelling reaction, samples were diluted in the deuterium labelling buffer in a 1:10 ratio to achieve a final excess D_2_O concentration of 90%. Labelling time points of 0.5, 10 and 60 min were sampled at 20 °C, with matching non-deuterated controls in H_2_O buffer. A minimum of triplicate analyses was obtained for each time point and condition. At the end of each labelling time, the reaction was stopped by adding quench buffer (1:1 ratio) to reach a final pH of 2.49. Protein samples were digested with a pepsin and protease XIII acidic dual protease column (2.1 × 3.0 mm; NovaBioAssays) at 8 °C for 3 min. Peptides were subsequently trapped on a 1.0 × 5.0 mm, 5.0-µm trap cartridge (Thermo Scientific Acclaim PepMap100) for desalting using a flow rate of 150 µl min^−1^. Peptides were separated on a Thermo Scientific Hypersil Gold column (50 × 1 mm, 1.9 μm, C18) by a linear gradient of 5% to 40% Buffer B (A, water and 0.1% FA; B, ACN and 0.1% FA) and a flow rate of 40 µl min^−1^. To limit peptide carry-over, a protease wash of 2 M guanidine and 0.8% formic acid, pH 2.3 in H_2_O was performed after each injection. To minimize back-exchange, the liquid chromatography system was maintained at a temperature of 1.5 °C. Labelling, quenching and online digestion steps were performed with the aid of an automated HDX robot from Trajan Scientific and Medical, which was guided by Chronos software (v.5.4.1). Samples were acquired in MS1 mode on a Thermo Scientific Orbitrap Exploris 480 Hybrid mass spectrometer.

#### Data analysis

In the first instance, an unspecific digested database of non-deuterated TEX264 and PARP1 peptides was generated in BioPharma Finder (v.5.2) using a data-dependent and targeted HCD-MS2 acquisition regime. Processing and curation of the labelling data were performed with the aid of HDExaminer v.3.4.2 (Trajan Scientific and Medical). The charge state with the highest quality spectra for all replicates for each peptide across all HDX-MS labelling times was used in the final analysis. AlphaFold was used to compare apo- and holo-states of TEX264 and the impact of PARP1 binding. Significant differences observed at each residue of the protein were used to map HDX-MS consensus effects (based on overlapping peptides) onto the AlphaFold model of TEX264. All MS raw files were deposited in ProteomeXchange (https://www.proteomexchange.org/) under the unique identifier PXD071389.

### RNA extraction and sequencing

For RNA-seq experiments, RNA was extracted from *TEX264*^−/−^ HeLa and CAL51 cells using the GeneJet RNA purification kit, carried out according to the manufacturer’s instructions. RNA was quantified by nano-drop to ensure the concentration was higher than 20 ng µl^−1^, and both the 260/230 and 260/280 ratios are above 2.0 to indicate sufficient purity. Three biological repeats were sent to Novogene for sequencing, RNA sample quality control, mRNA library preparation (polyA enrichment), Illumina sequencing and bioinformatic analysis. Bioinformatic analysis performed by Novogene included (1) data quality control and filtering; (2) mapping to reference genome GRCh38/hg38; (3) gene expression quantification and correlation analysis; (4) differential expression analysis; (5) enrichment analysis; and (6) gene set enrichment analysis.

### Genome-wide CRISPR–Cas9 screens analysis

Genome-wide CRISPR/–Cas9 screens were previously published^31,76^. For the analysis in this study, quality control was performed using R software (R Core Team, 2024). Sequence alignment and enrichment analysis (day 0 versus PARPi-treated population) were carried out using the MAGeCK Maximum Likelihood Estimation (MLE) module^131^ and the R package MAGeCKFlute^132^. The dataset of MAGeCK MLE analysis results of the CRISPR–Cas9 screen on RPE1-hTERT cells was extracted from the Supplementary Table 1 of Noordermeer et al.^31^. Datasets were robustly *z*-normalized and filtered against an untreated control population. Genes were considered depletion hits only when scoring at least two s.d. under the median of each screen, under the treated condition and not in an untreated control population. Functional enrichment analysis was performed using the R package clusterProfiler^133^ on the KEGG Pathways^134^, Reactome^135^, GO: Biological Process^136^ and Complex^137^ databases.

### Survival analysis based on TEX264 expression and HRD status

The unadjusted RNA-seq gene counts from the SCAN-B cohort^118^ were obtained (https://data.mendeley.com/datasets/yzxtxn4nmd/4), and read count normalization using DESeq2 (v.1.38.3) was conducted. The analysis was limited to TNBC (*n* = 712) in the SCAN-B cohort, as HRD survival trends were previously observed to differ between TNBC and HER2^+^ and ER^+^ subtypes^118,119^.

To annotate the HRD status of each patient in the SCAN-B cohort, a 228-HRD gene set from Jacobson et al.^128^ was used, to carry out non-negative matrix factorization (NMF package v.0.26, method = brunet, nrun = 100) to identify gene expression signatures associated with HRD. In addition, FPKM-normalized gene expression data from patients with TNBC (*n* = 312) in the TCGA-BRCA cohort were obtained from the GDC Data Portal (https://portal.gdc.cancer.gov/) to correlate HRD gene expression signatures in tumours previously annotated^128^ as HRD and HRP. Signature tnbc.2 correlated with HRD tumours in the TCGA cohort (Extended Data Fig. 10e) and matched signature scan-b 4, with a Pearson correlation of 0.86 (Extended Data Fig. 10f) in the SCAN-B cohort. SCAN-B TNBCs with proportion of scan-b 4 signature weights >0.25 were annotated as HRD.

SCAN-B tumours were stratified using *TEX264* expression based on quartiles, with the highest quartile as TEX264-high, the lowest quartile as TEX264-low and the remaining tumours labelled as TEX264-intermediate.

Survival analysis was performed in R using the survival package (v.3.5-5) with overall survival as the endpoint. Survival curves were compared using Kaplan–Meier curves generated using the survminer (v.0.5.0) package, and statistical tests were performed using a log-rank test.

### Statistics and reproducibility

Image data analysis and representation were carried out using CellProfiler (Broad Institute) and ImageJ (National Institutes of Health (NIH); https://imagej.net/Fiji/Downloads). Images are shown with scale bars of 10 µm unless otherwise stated. Graphs were plotted and statistical analysis was performed using Prism v.10 (GraphPad Software). A minimum of 150 cells was quantified per condition. No statistical method was used to predetermine the sample size for any experiment, but sample sizes were comparable with those reported in similar studies. CellProfiler ensured blinding during quantification to limit bias. Data collection and analysis were not performed blind to the conditions of other experiments, and investigators were not blinded to allocation during experiments and outcome assessment. For some imaging experiments with large datasets, Prism’s inbuilt rapid regression and outlier removal method was used. A *q* value of 1 was applied to ensure any outliers were detected with an FDR < 1%. No other data were excluded from the analyses. Unless otherwise stated, experiments were not randomized. All experiments were performed at least twice, with the number of replicates indicated in the figure legends. Error bars show s.e.m. Tukey box plots are standard, where the centre line represents the median, the box indicates the interquartile range, and whiskers indicate 1.5 × interquartile range. Statistical significance was assessed using two-sided Student’s *t*-tests, one-way ANOVA or two-way ANOVA, as indicated in the figure legends. Where appropriate, multiple comparison post hoc tests were applied as indicated in the figure legends. Asterisks are used to indicate *P* values (NS, *P* > 0.05; *P ≤ 0.05; **P ≤ 0.01; ***P ≤ 0.001; ****P ≤ 0.0001). Data distribution was assumed to be normal, but this was not formally tested.

### Reporting summary

Further information on research design is available in the Nature Portfolio Reporting Summary linked to this article.

## Online content

Any methods, additional references, Nature Portfolio reporting summaries, source data, extended data, supplementary information, acknowledgements, peer review information; details of author contributions and competing interests; and statements of data and code availability are available at 10.1038/s41556-026-01961-5.

## Supplementary information

Reporting Summary

Peer Review File

Supplementary Video 1 Live imaging of HeLa cell expressing the mCherry–PARP1–GFP reporter fusion protein 3 min after treatment with talazoparib (200 nM) and MMS (0.01%), redefined as 0 min in the video. Video related to Fig. 2I. Nucleus appears yellow as both mCherry and GFP fluoresce. Only red-only puncta are visible when the reporter protein is in the acidic environment of the lysosomal lumen. Lysosomes are labelled with LysoView680. Squares depict zoomed views in the insets. Scale bar, 10 μm; scale bar inset, 1 μm. Frame rate, 0.6f ps.

Supplementary Video 2 Live imaging of HeLa cell expressing the mCherry–PARP1–GFP reporter fusion protein 24 min 30 s after treatment with talazoparib and MMS, redefined as 0 min in the video. Movie related to Extended Data Figure 3f,g. Nucleus appears yellow as both mCherry and GFP fluoresce. Only red-only puncta are visible when the reporter protein is in the acidic environment of the lysosomal lumen. Lysosomes are labelled with LysoView680. Squares depict zoomed views in the insets. Scale bar, 10 μm; scale bar inset, 1 μm. Frame rate, 0.6 fps.

Supplementary Tables 1–9 Supplementary Table 1 GO terms from gene set enrichment analysis by RNA-seq in CAL51 talazoparib-treated versus untreated cells, shown in Extended Data Fig. 1a. Supplementary Table 2 RNA-seq in CAL51 talazoparib versus untreated cells as shown in Fig 1a. Autophagy-related genes^71^ highlighted in grey. Genes related to positive regulation of the proteasome (GO:1901800) are highlighted in red. Supplementary Table 3 The list of 22 autophagy genes^71^ identified in the PARP1–APEX proteome of trapped PARP1 as described. Supplementary Table 4 RNA-seq in HeLa TEX264^−/−^ versus WT cells for volcano plot in Extended Data Fig. 7a. Genes of interest highlighted in accordance with colours in the volcano plot. Supplementary Table 5 RNA-seq in CAL51 TEX264^−/−^ versus WT cells for volcano plot in Extended Data Fig 7a. Genes of interest highlighted in accordance with colours in the volcano plot. Supplementary Table 6 HDX summary table for data shown in Fig. 5d. The table includes key results for the HDX-MS experiment in both the apo- and holo- state of TEX264 in the presence or absence of PARP1, including the number of peptides identified, sequence coverage, peptide redundancy and experimental reproducibility. Supplementary Table 7 HDX uptake summary for the peptides detected in HDX-MS shown in Extended Data Fig. 5b. Supplementary Table 8 Key Resource Table. Supplementary Table 9 Light microscopy reporting table.

## Source data

Source Data Fig. 1 Statistical Source Data.

Source Data Fig. 2 Unprocessed western blots.

Source Data Fig. 2 Statistical Source Data.

Source Data Fig. 3 Unprocessed western blots.

Source Data Fig. 3 Statistical Source Data.

Source Data Fig. 4 Unprocessed western blots.

Source Data Fig. 4 Statistical Source Data.

Source Data Fig. 5 Unprocessed western blots.

Source Data Fig. 5 Statistical Source Data.

Source Data Fig. 6 Unprocessed western blots.

Source Data Fig. 6 Statistical Source Data.

Source Data Extended Data Fig. 2 Unprocessed western blots.

Source Data Extended Data Fig. 2 Statistical Source Data.

Source Data Extended Data Fig. 3 Unprocessed western blots.

Source Data Extended Data Fig. 3 Statistical Source Data.

Source Data Extended Data Fig. 4 Unprocessed western blots.

Source Data Extended Data Fig. 4 Statistical Source Data.

Source Data Extended Data Fig. 5 Unprocessed western blots.

Source Data Extended Data Fig. 6 Unprocessed western blots.

Source Data Extended Data Fig. 6 Statistical Source Data.

Source Data Extended Data Fig. 7 Unprocessed western blots.

Source Data Extended Data Fig. 7 Statistical Source Data.

Source Data Extended Data Fig. 8 Unprocessed western blots.

Source Data Extended Data Fig. 8 Statistical Source Data.

Source Data Extended Data Fig. 9 Unprocessed western blots.

Source Data Extended Data Fig. 9 Statistical Source Data and gating strategy.

## Data Availability

All data generated, analysed and used in this study are included in this published article and its Supplementary Information and Source Data. All other data supporting the findings of this study are available from the corresponding author on reasonable request. All plasmids generated in this manuscript will be deposited in Addgene (https://www.addgene.org/browse/). Plasmids should be requested directly from Addgene. Cell lines created in this study are available from the corresponding author upon reasonable request. Source data from published CRISPR screens are available in the European Nucleotide Archive (ENA) under accession number PRJEB74933 for data from Dibitetto et al.^76^ and in supplementary table 1 of Noordermeer et al.^31^. Published mass spectrometry data^39^ are available in the ProteomeXchange Consortium via the PRIDE partner repository (dataset identifier PXD024337). RNA-seq data that support the findings of this study have been deposited in the Gene Expression Omnibus (GEO) under accession codes GSE277366. Mass spectrometry data have been deposited in ProteomeXchange with the primary accession code PXD071389. Microscopy data generated in this study have been deposited in the BioImage Archive under accession number S-BIAD3162 (https://www.ebi.ac.uk/biostudies/bioimages/studies/S-BIAD3162), where they are freely available in a lossless format. Source data are provided with this paper.

## References

[1] Plummer, E. R. et al. Temozolomide pharmacodynamics in patients with metastatic melanoma: DNA damage and activity of repair enzymes O6-alkylguanine alkyltransferase and poly(ADP-ribose) polymerase-1. *Clin. Cancer Res.* **11**, 3402–3409 (2005). 15867241 10.1158/1078-0432.CCR-04-2353

[2] Deeks, E. D. Olaparib: first global approval. *Drugs* **75**, 231–240 (2015). 25616434 10.1007/s40265-015-0345-6

[3] Hoy, S. M. Talazoparib: first global approval. *Drugs* **78**, 1939–1946 (2018). 30506138 10.1007/s40265-018-1026-z

[4] Wicks, A. J. et al. Opinion: PARP inhibitors in cancer-what do we still need to know? *Open Biol.* **12**, 220118 (2022). 35892198 10.1098/rsob.220118 PMC9326299

[5] Bryant, H. E. et al. Specific killing of BRCA2-deficient tumours with inhibitors of poly(ADP-ribose) polymerase. *Nature* **434**, 913–917 (2005). 15829966 10.1038/nature03443

[6] Farmer, H. et al. Targeting the DNA repair defect in BRCA mutant cells as a therapeutic strategy. *Nature* **434**, 917–921 (2005). 15829967 10.1038/nature03445

[7] Lord, C. J. & Ashworth, A. PARP inhibitors: synthetic lethality in the clinic. *Science* **355**, 1152–1158 (2017). 28302823 10.1126/science.aam7344 PMC6175050

[8] Kanev, P.-B. et al. PARP1 roles in DNA repair and DNA replication: the basi(c)s of PARP inhibitor efficacy and resistance. *Semin. Oncol.* **51**, 2–18 (2024). 37714792 10.1053/j.seminoncol.2023.08.001

[9] Maya-Mendoza, A. et al. High speed of fork progression induces DNA replication stress and genomic instability. *Nature* **559**, 279–284 (2018). 29950726 10.1038/s41586-018-0261-5

[10] Hanzlikova, H. et al. The importance of poly(ADP-ribose) polymerase as a sensor of unligated Okazaki fragments during DNA replication. *Mol. Cell* **71**, 319–331.e3 (2018). 29983321 10.1016/j.molcel.2018.06.004 PMC6060609

[11] Pettitt, S. J. et al. Genome-wide and high-density CRISPR-Cas9 screens identify point mutations in PARP1 causing PARP inhibitor resistance. *Nat. Commun.* **9**, 1849 (2018). 29748565 10.1038/s41467-018-03917-2 PMC5945626

[12] Helleday, T. The underlying mechanism for the PARP and BRCA synthetic lethality: clearing up the misunderstandings. *Mol. Oncol.* **5**, 387–393 (2011). 21821475 10.1016/j.molonc.2011.07.001 PMC5528309

[13] Murai, J. et al. Trapping of PARP1 and PARP2 by clinical PARP inhibitors. *Cancer Res.* **72**, 5588–5599 (2012). 23118055 10.1158/0008-5472.CAN-12-2753 PMC3528345

[14] Murai, J. et al. Stereospecific PARP trapping by BMN 673 and comparison with olaparib and rucaparib. *Mol. Cancer Ther.* **13**, 433–443 (2014). 24356813 10.1158/1535-7163.MCT-13-0803 PMC3946062

[15] Pommier, Y., O’Connor, M. J. & de Bono, J. Laying a trap to kill cancer cells: PARP inhibitors and their mechanisms of action. *Sci. Transl. Med.* **8**, 362ps17 (2016). 27797957 10.1126/scitranslmed.aaf9246

[16] Shen, Y. et al. BMN 673, a novel and highly potent PARP1/2 inhibitor for the treatment of human cancers with DNA repair deficiency. *Clin. Cancer Res.* **19**, 5003–5015 (2013). 23881923 10.1158/1078-0432.CCR-13-1391 PMC6485449

[17] D’Andrea, A. D. Mechanisms of PARP inhibitor sensitivity and resistance. *DNA Repair* **71**, 172–176 (2018). 30177437 10.1016/j.dnarep.2018.08.021

[18] Li, H. et al. PARP inhibitor resistance: the underlying mechanisms and clinical implications. *Mol. Cancer* **19**, 107 (2020). 32563252 10.1186/s12943-020-01227-0 PMC7305609

[19] Audeh, M. W. et al. Oral poly(ADP-ribose) polymerase inhibitor olaparib in patients with *BRCA1* or *BRCA2* mutations and recurrent ovarian cancer: a proof-of-concept trial. *Lancet* **376**, 245–251 (2010). 20609468 10.1016/S0140-6736(10)60893-8

[20] Fong, P. C. et al. Poly(ADP)-ribose polymerase inhibition: frequent durable responses in BRCA carrier ovarian cancer correlating with platinum-free interval. *J. Clin. Oncol.* **28**, 2512–2519 (2010). 20406929 10.1200/JCO.2009.26.9589

[21] Kristeleit, R. et al. A phase I-II study of the oral PARP inhibitor rucaparib in patients with germline *BRCA1/2*-mutated ovarian carcinoma or other solid tumors. *Clin. Cancer Res.* **23**, 4095–4106 (2017). 28264872 10.1158/1078-0432.CCR-16-2796

[22] Drost, R. et al. *BRCA1*^*185delAG*^ tumors may acquire therapy resistance through expression of RING-less BRCA1. *J. Clin. Invest.* **126**, 2903–2918 (2016). 10.1172/JCI70196 PMC4966325 27454287

[23] Johnson, N. et al. Stabilization of mutant BRCA1 protein confers PARP inhibitor and platinum resistance. *Proc. Natl Acad. Sci. USA* **110**, 17041–17046 (2013). 24085845 10.1073/pnas.1305170110 PMC3801063

[24] Wang, Y. et al. RING domain-deficient BRCA1 promotes PARP inhibitor and platinum resistance. *J. Clin. Invest.* **126**, 3145–3157 (2016). 27454289 10.1172/JCI87033 PMC4966309

[25] Harvey-Jones, E. et al. Longitudinal profiling identifies co-occurring BRCA1/2 reversions, TP53BP1, RIF1 and PAXIP1 mutations in PARP inhibitor-resistant advanced breast cancer. *Ann. Oncol.* **35**, 364–380 (2024). 38244928 10.1016/j.annonc.2024.01.003

[26] Chu, Y. Y. et al. Biomarkers beyond BRCA: promising combinatorial treatment strategies in overcoming resistance to PARP inhibitors. *J. Biomed. Sci.* **29**, 86 (2022). 36284291 10.1186/s12929-022-00870-7 PMC9594904

[27] Tobalina, L. et al. A meta-analysis of reversion mutations in *BRCA* genes identifies signatures of DNA end-joining repair mechanisms driving therapy resistance. *Ann. Oncol.* **32**, 103–112 (2021). 33091561 10.1016/j.annonc.2020.10.470

[28] Dev, H. et al. Shieldin complex promotes DNA end-joining and counters homologous recombination in BRCA1-null cells. *Nat. Cell Biol.* **20**, 954–965 (2018). 30022119 10.1038/s41556-018-0140-1 PMC6145444

[29] Jaspers, J. E. et al. Loss of 53BP1 causes PARP inhibitor resistance in *Brca1*-mutated mouse mammary tumors. *Cancer Discov.* **3**, 68–81 (2013). 23103855 10.1158/2159-8290.CD-12-0049 PMC7518105

[30] Xu, G. et al. REV7 counteracts DNA double-strand break resection and affects PARP inhibition. *Nature* **521**, 541–544 (2015). 25799992 10.1038/nature14328 PMC4671316

[31] Noordermeer, S. M. et al. The shieldin complex mediates 53BP1-dependent DNA repair. *Nature* **560**, 117–121 (2018). 30022168 10.1038/s41586-018-0340-7 PMC6141009

[32] Ray Chaudhuri, A. et al. Replication fork stability confers chemoresistance in BRCA-deficient cells. *Nature* **535**, 382–387 (2016). 27443740 10.1038/nature18325 PMC4959813

[33] Gogola, E. et al. Selective loss of PARG restores PARylation and counteracts PARP inhibitor-mediated synthetic lethality. *Cancer Cell* **33**, 1078–1093 e12 (2018). 29894693 10.1016/j.ccell.2018.05.008

[34] Christie, E. L. et al. Multiple ABCB1 transcriptional fusions in drug resistant high-grade serous ovarian and breast cancer. *Nat. Commun.* **10**, 1295 (2019). 30894541 10.1038/s41467-019-09312-9 PMC6426934

[35] Patch, A. M. et al. Whole-genome characterization of chemoresistant ovarian cancer. *Nature* **521**, 489–494 (2015). 26017449 10.1038/nature14410

[36] Rottenberg, S. et al. High sensitivity of BRCA1-deficient mammary tumors to the PARP inhibitor AZD2281 alone and in combination with platinum drugs. *Proc. Natl Acad. Sci. USA* **105**, 17079–17084 (2008). 18971340 10.1073/pnas.0806092105 PMC2579381

[37] Zandarashvili, L. et al. Structural basis for allosteric PARP-1 retention on DNA breaks. *Science* **368**, eaax6367 (2020). 32241924 10.1126/science.aax6367 PMC7347020

[38] Saha, L. K., et al. Replication-dependent cytotoxicity and Spartan-mediated repair of trapped PARP1-DNA complexes. *Nucleic Acids Res*. 10.1093/nar/gkab777 (2021). 10.1093/nar/gkab777 PMC8501961 34551432

[39] Krastev, D. B. et al. The ubiquitin-dependent ATPase p97 removes cytotoxic trapped PARP1 from chromatin. *Nat. Cell Biol.* **24**, 62–73 (2022). 35013556 10.1038/s41556-021-00807-6 PMC8760077

[40] Fielden, J. et al. TEX264 coordinates p97- and SPRTN-mediated resolution of topoisomerase 1-DNA adducts. *Nat. Commun.* **11**, 1274 (2020). 32152270 10.1038/s41467-020-15000-w PMC7062751

[41] Kilgas, S. & Ramadan, K. Inhibitors of the ATPase p97/VCP: from basic research to clinical applications. *Cell Chem. Biol*. **30**, 3–21 (2023). 36640759 10.1016/j.chembiol.2022.12.007

[42] Ramadan, K. et al. Strategic role of the ubiquitin-dependent segregase p97 (VCP or Cdc48) in DNA replication. *Chromosoma* **126**, 17–32 (2016). 27086594 10.1007/s00412-016-0587-4

[43] van den Boom, J. & Meyer, H. VCP/p97-mediated unfolding as a principle in protein homeostasis and signaling. *Mol. Cell* **69**, 182–194 (2018). 29153394 10.1016/j.molcel.2017.10.028

[44] Torrecilla, I., Oehler, J. & Ramadan, K. The role of ubiquitin-dependent segregase p97 (VCP or Cdc48) in chromatin dynamics after DNA double strand breaks. *Phil. Trans. R. Soc. Lond. B Biol. Sci.* **372**, 20160282 (2017). 28847819 10.1098/rstb.2016.0282 PMC5577460

[45] Noireterre, A. & Stutz, F. Cdc48/p97 segregase: spotlight on DNA-protein crosslinks. *DNA Repair* **139**, 103691 (2024). 38744091 10.1016/j.dnarep.2024.103691

[46] Franz, A., Ackermann, L. & Hoppe, T. Ring of change: CDC48/p97 drives protein dynamics at chromatin. *Front. Genet.* **7**, 73 (2016). 27200082 10.3389/fgene.2016.00073 PMC4853748

[47] Dantuma, N. P. & Hoppe, T. Growing sphere of influence: Cdc48/p97 orchestrates ubiquitin-dependent extraction from chromatin. *Trends Cell Biol.* **22**, 483–491 (2012). 22818974 10.1016/j.tcb.2012.06.003

[48] Buchberger, A., Schindelin, H. & Hanzelmann, P. Control of p97 function by cofactor binding. *FEBS Lett*. **589**, 2578–2589 (2015). 26320413 10.1016/j.febslet.2015.08.028

[49] Lascaux, P. et al. TEX264 drives selective autophagy of DNA lesions to promote DNA repair and cell survival. *Cell* **187**, 1–21 (2024). 39265577 10.1016/j.cell.2024.08.020

[50] Morishita, H. & Mizushima, N. Diverse cellular roles of autophagy. *Annu. Rev. Cell Dev. Biol.* **35**, 453–475 (2019). 31283377 10.1146/annurev-cellbio-100818-125300

[51] An, H. et al. TEX264 is an endoplasmic reticulum-resident ATG8-interacting protein critical for ER remodeling during nutrient stress. *Mol. Cell* **74**, 891–908.e10 (2019). 31006537 10.1016/j.molcel.2019.03.034 PMC6747008

[52] Chino, H. et al. Intrinsically disordered protein TEX264 mediates ER-phagy. *Mol. Cell* **74**, 909–921.e6 (2019). 31006538 10.1016/j.molcel.2019.03.033

[53] Slobodkin, M. R. & Elazar, Z. The Atg8 family: multifunctional ubiquitin-like key regulators of autophagy. *Essays Biochem.* **55**, 51–64 (2013). 24070471 10.1042/bse0550051

[54] Vargas, J. N. S. et al. The mechanisms and roles of selective autophagy in mammals. *Nat. Rev. Mol. Cell Biol.* **24**, 167–185 (2023). 36302887 10.1038/s41580-022-00542-2

[55] Hama, Y., Ogasawara, Y. & Noda, N. N. Autophagy and cancer: basic mechanisms and inhibitor development. *Cancer Sci*. **114**, 2699–2708 (2023). 37010190 10.1111/cas.15803 PMC10323110

[56] Cahuzac, M. et al. Pre-activation of autophagy impacts response to olaparib in prostate cancer cells. *Commun. Biol.* **5**, 251 (2022). 35318456 10.1038/s42003-022-03210-5 PMC8940895

[57] Elshazly, A. M., Nguyen, T. V. V. & Gewirtz, D. A. Is autophagy induction by PARP inhibitors a target for therapeutic benefit? *Oncol. Res.* **30**, 1–12 (2022). 37304006 10.32604/or.2022.026459 PMC10208061

[58] Liu, Y. et al. Targeting autophagy potentiates the anti-tumor effect of PARP inhibitor in pediatric chronic myeloid leukemia. *AMB Express* **9**, 108 (2019). 31309361 10.1186/s13568-019-0836-z PMC6629728

[59] Pai Bellare, G. & Sankar Patro, B. Resveratrol sensitizes breast cancer to PARP inhibitor, talazoparib through dual inhibition of AKT and autophagy flux. *Biochem. Pharmacol*. **199**, 115024 (2022). 35367197 10.1016/j.bcp.2022.115024

[60] Pai Bellare, G., Saha, B. & Patro, B. S. Targeting autophagy reverses de novo resistance in homologous recombination repair proficient breast cancers to PARP inhibition. *Br. J. Cancer* **124**, 1260–1274 (2021). 33473172 10.1038/s41416-020-01238-0 PMC8007595

[61] Ren, H. et al. Design, synthesis, and characterization of an orally active dual-specific ULK1/2 autophagy inhibitor that synergizes with the PARP inhibitor olaparib for the treatment of triple-negative breast cancer. *J. Med. Chem.* **63**, 14609–14625 (2020). 33200929 10.1021/acs.jmedchem.0c00873 PMC8064294

[62] Santiago-O’Farrill, J. M. et al. Poly(adenosine diphosphate ribose) polymerase inhibitors induce autophagy-mediated drug resistance in ovarian cancer cells, xenografts, and patient-derived xenograft models. *Cancer* **126**, 894–907 (2020). 31714594 10.1002/cncr.32600 PMC6992526

[63] Uddin, M. H. et al. Proteomic analysis identifies p62/SQSTM1 as a critical player in PARP inhibitor resistance. *Front. Oncol.* **12**, 908603 (2022). 35847859 10.3389/fonc.2022.908603 PMC9277186

[64] Hopkins, T. A. et al. PARP1 trapping by PARP inhibitors drives cytotoxicity in both cancer cells and healthy bone marrow. *Mol. Cancer Res.* **17**, 409–419 (2019). 30429212 10.1158/1541-7786.MCR-18-0138

[65] Shen, Y., Aoyagi-Scharber, M. & Wang, B. Trapping poly(ADP-ribose) polymerase. *J. Pharmacol. Exp. Ther.* **353**, 446–457 (2015). 25758918 10.1124/jpet.114.222448

[66] Götz, M. J. & Stingele, J. Releasing the trap: How the segregase p97 extracts PARP1 from chromatin. *Mol. Cell* **82**, 889–890 (2022). 35245455 10.1016/j.molcel.2022.02.012

[67] Brownlee, P. M., Provencher, L. & Goodarzi, A. A. Unsprung traps keep PARP inhibitors effective. *Nat. Cell Biol.* **24**, 2–4 (2022). 35013555 10.1038/s41556-021-00819-2

[68] Chang, B. D. et al. Molecular determinants of terminal growth arrest induced in tumor cells by a chemotherapeutic agent. *Proc. Natl Acad. Sci. USA* **99**, 389–394 (2002). 11752408 10.1073/pnas.012602599 PMC117570

[69] Nag, S. et al. The MDM2-p53 pathway revisited. *J. Biomed. Res.* **27**, 254–271 (2013). 23885265 10.7555/JBR.27.20130030 PMC3721034

[70] Youle, R. J. & Strasser, A. The BCL-2 protein family: opposing activities that mediate cell death. *Nat. Rev. Mol. Cell Biol*. **9**, 47–59 (2008). 18097445 10.1038/nrm2308

[71] Bordi, M. et al. A gene toolbox for monitoring autophagy transcription. *Cell Death Dis.* **12**, 1044 (2021). 34728604 10.1038/s41419-021-04121-9 PMC8563709

[72] Ambrosio, S. & Majello, B. Autophagy roles in genome maintenance. *Cancers* **12**, 1793 (2020). 32635505 10.3390/cancers12071793 PMC7407194

[73] Arun, B. et al. The PARP inhibitor AZD2281 (Olaparib) induces autophagy/mitophagy in BRCA1 and BRCA2 mutant breast cancer cells. *Int. J. Oncol.* **47**, 262–268 (2015). 25975349 10.3892/ijo.2015.3003 PMC6904111

[74] Macrae, T. et al. RNA-seq reveals spliceosome and proteasome genes as most consistent transcripts in human cancer cells. *PLoS ONE* **8**, e72884 (2013). 24069164 10.1371/journal.pone.0072884 PMC3775772

[75] Evers, B. et al. Selective inhibition of BRCA2-deficient mammary tumor cell growth by AZD2281 and cisplatin. *Clin. Cancer Res.* **14**, 3916–3925 (2008). 18559613 10.1158/1078-0432.CCR-07-4953

[76] Dibitetto, D. et al. H2AX promotes replication fork degradation and chemosensitivity in BRCA-deficient tumours. *Nat. Commun.* **15**, 4430 (2024). 38789420 10.1038/s41467-024-48715-1 PMC11126719

[77] Cui, L. et al. Deubiquitinase USP7 regulates *Drosophila* aging through ubiquitination and autophagy. *Aging* **12**, 23082–23095 (2020). 33221768 10.18632/aging.104067 PMC7746378

[78] Peng, H. et al. The ubiquitin-specific protease USP8 directly deubiquitinates SQSTM1/p62 to suppress its autophagic activity. *Autophagy* **16**, 698–708 (2020). 31241013 10.1080/15548627.2019.1635381 PMC7138243

[79] Durcan, T. M. & Fon, E. A. USP8 and PARK2/parkin-mediated mitophagy. *Autophagy* **11**, 428–429 (2015). 25700639 10.1080/15548627.2015.1009794 PMC4502724

[80] Li, X. et al. CUL3 (cullin 3)-mediated ubiquitination and degradation of BECN1 (beclin 1) inhibit autophagy and promote tumor progression. *Autophagy* **17**, 4323–4340 (2021). 33977871 10.1080/15548627.2021.1912270 PMC8726624

[81] Ferrari, V. et al. Valosin containing protein (VCP): a multistep regulator of autophagy. *Int. J. Mol. Sci.* **23**, 1939 (2022). 35216053 10.3390/ijms23041939 PMC8878954

[82] Gatti, M. et al. The ubiquitin ligase TRIP12 limits PARP1 trapping and constrains PARP inhibitor efficiency. *Cell Rep.* **32**, 107985 (2020). 32755579 10.1016/j.celrep.2020.107985 PMC7408484

[83] Li, P. et al. Nimbolide targets RNF114 to induce the trapping of PARP1 and synthetic lethality in BRCA-mutated cancer. *Sci. Adv.* **9**, eadg7752 (2023). 37878693 10.1126/sciadv.adg7752 PMC10599614

[84] Sun, X. et al. Loss of the receptors ER, PR and HER2 promotes USP15-dependent stabilization of PARP1 in triple-negative breast cancer. *Nat. Cancer* **4**, 716–733 (2023). 37012401 10.1038/s43018-023-00535-w

[85] Itakura, E., Kishi-Itakura, C. & Mizushima, N. The hairpin-type tail-anchored SNARE syntaxin 17 targets to autophagosomes for fusion with endosomes/lysosomes. *Cell* **151**, 1256–1269 (2012). 23217709 10.1016/j.cell.2012.11.001

[86] Viret, C. & Faure, M. Regulation of syntaxin 17 during autophagosome maturation. *Trends Cell Biol.* **29**, 1–3 (2019). 30415939 10.1016/j.tcb.2018.10.003

[87] Cahuzac, M. et al. Development of olaparib-resistance prostate cancer cell lines to identify mechanisms associated with acquired resistance. *Cancers* **14**, 3877 (2022). 36010871 10.3390/cancers14163877 PMC9405809

[88] Abu-Remaileh, M. et al. Lysosomal metabolomics reveals V-ATPase- and mTOR-dependent regulation of amino acid efflux from lysosomes. *Science* **358**, 807–813 (2017). 29074583 10.1126/science.aan6298 PMC5704967

[89] Kimura, S., Noda, T. & Yoshimori, T. Dissection of the autophagosome maturation process by a novel reporter protein, tandem fluorescent-tagged LC3. *Autophagy* **3**, 452–460 (2007). 17534139 10.4161/auto.4451

[90] Bug, M. & Meyer, H. Expanding into new markets–VCP/p97 in endocytosis and autophagy. *J. Struct. Biol.* **179**, 78–82 (2012). 22450227 10.1016/j.jsb.2012.03.003

[91] Hänzelmann, P., Galgenmüller, C. & Schindelin, H. Structure and function of the AAA+ ATPase p97, a key player in protein homeostasis. *Subcell. Biochem.* **93**, 221–272 (2019). 31939153 10.1007/978-3-030-28151-9_7

[92] Wrobel, L. et al. p37 regulates VCP/p97 shuttling and functions in the nucleus and cytosol. *Sci. Adv.* **10**, eadl6082 (2024). 38701207 10.1126/sciadv.adl6082 PMC11068011

[93] Hill, S. M. et al. VCP/p97 regulates Beclin-1-dependent autophagy initiation. *Nat. Chem. Biol.* **17**, 448–455 (2021). 33510452 10.1038/s41589-020-00726-x

[94] Sun, Y. et al. A conserved SUMO pathway repairs topoisomerase DNA-protein cross-links by engaging ubiquitin-mediated proteasomal degradation. *Sci. Adv.* **6**, eaba6290 (2020). 33188014 10.1126/sciadv.aba6290 PMC7673754

[95] Sriramachandran, A. M. & Dohmen, R. J. SUMO-targeted ubiquitin ligases. *Biochim. Biophys. Acta* **1843**, 75–85 (2014). 24018209 10.1016/j.bbamcr.2013.08.022

[96] Martin, N. et al. PARP-1 transcriptional activity is regulated by sumoylation upon heat shock. *EMBO J.* **28**, 3534–3548 (2009). 19779455 10.1038/emboj.2009.279 PMC2782092

[97] Fielden, J., Popovic, M. & Ramadan, K. TEX264 at the intersection of autophagy and DNA repair. *Autophagy* **18**, 40–49 (2022). 33726628 10.1080/15548627.2021.1894059 PMC8865260

[98] Delorme-Axford, E., Popelka, H. & Klionsky, D. J. TEX264 is a major receptor for mammalian reticulophagy. *Autophagy* **15**, 1677–1681 (2019). 31362563 10.1080/15548627.2019.1646540 PMC6735500

[99] Yeung, H. O. et al. Insights into adaptor binding to the AAA protein p97. *Biochem. Soc. Trans.* **36**, 62–67 (2008). 18208387 10.1042/BST0360062

[100] Vyas, S. et al. A systematic analysis of the PARP protein family identifies new functions critical for cell physiology. *Nat. Commun*. **4**, 2240 (2013). 23917125 10.1038/ncomms3240 PMC3756671

[101] Johansen, T. & Lamark, T. Selective autophagy: ATG8 Family proteins, LIR motifs and cargo receptors. *J. Mol. Biol.* **432**, 80–103 (2020). 31310766 10.1016/j.jmb.2019.07.016

[102] Kucińska, M. K. et al. TMX4-driven LINC complex disassembly and asymmetric autophagy of the nuclear envelope upon acute ER stress. *Nat. Commun.* **14**, 3497 (2023). 37311770 10.1038/s41467-023-39172-3 PMC10264389

[103] Dou, Z. et al. Autophagy mediates degradation of nuclear lamina. *Nature* **527**, 105–109 (2015). 26524528 10.1038/nature15548 PMC4824414

[104] Li, Y. et al. Nuclear accumulation of UBC9 contributes to SUMOylation of lamin A/C and nucleophagy in response to DNA damage. *J. Exp. Clin. Cancer Res.* **38**, 67 (2019). 30744690 10.1186/s13046-019-1048-8 PMC6371487

[105] Kovacs, M. T. et al. DNA damage induces nuclear envelope rupture through ATR-mediated phosphorylation of lamin A/C. *Mol. Cell* **83**, 3659–3668.e10 (2023). 37832547 10.1016/j.molcel.2023.09.023 PMC10597398

[106] Joo, Y. K. et al. ATR promotes clearance of damaged DNA and damaged cells by rupturing micronuclei. *Mol. Cell* **83**, 3642–3658.e4 (2023). 37788673 10.1016/j.molcel.2023.09.003 PMC10599252

[107] da Costa, A. et al. Targeting replication stress in cancer therapy. *Nat. Rev. Drug Discov.* **22**, 38–58 (2023). 36202931 10.1038/s41573-022-00558-5 PMC11132912

[108] Gralewska, P. et al. PARP inhibition increases the reliance on ATR/CHK1 checkpoint signaling leading to synthetic lethality—an alternative treatment strategy for epithelial ovarian cancer cells independent from HR effectiveness. *Int. J. Mol. Sci.* **21**, 9715 (2020). 33352723 10.3390/ijms21249715 PMC7766831

[109] Michelena, J. et al. Analysis of PARP inhibitor toxicity by multidimensional fluorescence microscopy reveals mechanisms of sensitivity and resistance. *Nat. Commun.* **9**, 2678 (2018). 29992957 10.1038/s41467-018-05031-9 PMC6041334

[110] Belan, O. et al. POLQ seals post-replicative ssDNA gaps to maintain genome stability in BRCA-deficient cancer cells. *Mol. Cell* **82**, 4664–4680 e9 (2022). 36455556 10.1016/j.molcel.2022.11.008

[111] Cong, K. et al. Replication gaps are a key determinant of PARP inhibitor synthetic lethality with BRCA deficiency. *Mol. Cell* **81**, 3128–3144 e7 (2021). 34216544 10.1016/j.molcel.2021.06.011 PMC9089372

[112] Paes Dias, M. et al. Loss of nuclear DNA ligase III reverts PARP inhibitor resistance in BRCA1/53BP1 double-deficient cells by exposing ssDNA gaps. *Mol. Cell* **81**, 4692–4708.e9 (2021). 34555355 10.1016/j.molcel.2021.09.005 PMC9098260

[113] Roux, B. et al. Targeting acute myeloid leukemia dependency on VCP-mediated DNA repair through a selective second-generation small-molecule inhibitor. *Sci. Transl. Med.* **13**, eabg1168 (2021). 33790022 10.1126/scitranslmed.abg1168 PMC8672851

[114] Jackson, S. P. & Bartek, J. The DNA-damage response in human biology and disease. *Nature* **461**, 1071–1078 (2009). 19847258 10.1038/nature08467 PMC2906700

[115] Ravikumar, B., Duden, R. & Rubinsztein, D. C. Aggregate-prone proteins with polyglutamine and polyalanine expansions are degraded by autophagy. *Hum. Mol. Genet.* **11**, 1107–1117 (2002). 11978769 10.1093/hmg/11.9.1107

[116] Kobayashi, T., Manno, A. & Kakizuka, A. Involvement of valosin-containing protein (VCP)/p97 in the formation and clearance of abnormal protein aggregates. *Genes Cells* **12**, 889–901 (2007). 17584300 10.1111/j.1365-2443.2007.01099.x

[117] Mukkavalli, S. et al. The p97-UBXN1 complex regulates aggresome formation. *J. Cell Sci.* **134**, jcs254201 (2021). 33712450 10.1242/jcs.254201 PMC8077447

[118] Staaf, J. et al. RNA sequencing-based single sample predictors of molecular subtype and risk of recurrence for clinical assessment of early-stage breast cancer. *NPJ Breast Cancer* **8**, 94 (2022). 35974007 10.1038/s41523-022-00465-3 PMC9381586

[119] Staaf, J. et al. Whole-genome sequencing of triple-negative breast cancers in a population-based clinical study. *Nat. Med.* **25**, 1526–1533 (2019). 31570822 10.1038/s41591-019-0582-4 PMC6859071

[120] Muciño-Hernández, G. et al. Nucleophagy contributes to genome stability through degradation of type II topoisomerases A and B and nucleolar components. *J. Cell Sci.* **136**, jcs260563 (2023). 36633090 10.1242/jcs.260563 PMC10112964

[121] Aveleira, C. et al. Dynamins maintain nuclear envelope homeostasis and genome stability. *Nat. Commun.* **17**, 1380 (2026). 41507158 10.1038/s41467-025-68130-4 PMC12876909

[122] Mannino, P. J. et al. A quantitative ultrastructural timeline of nuclear autophagy reveals a role for dynamin-like protein 1 at the nuclear envelope. *Nat. Cell Biol.* **27**, 464–476 (2025). 39920277 10.1038/s41556-025-01612-1 PMC11908896

[123] Kuchitsu, Y. & Taguchi, T. Lysosomal microautophagy: an emerging dimension in mammalian autophagy. *Trends Cell Biol*. **34**, 606–616 (2024). 38104013 10.1016/j.tcb.2023.11.005

[124] Hewitt, G. & Korolchuk, V. I. Repair, reuse, recycle: the expanding role of autophagy in genome maintenance. *Trends Cell Biol*. **27**, 340–351 (2017). 28011061 10.1016/j.tcb.2016.11.011

[125] Xiao, M. et al. NLRP4 renders pancreatic cancer resistant to olaparib through promotion of the DNA damage response and ROS-induced autophagy. *Cell Death Dis.* **15**, 620 (2024). 39187531 10.1038/s41419-024-06984-0 PMC11347561

[126] Amaravadi, R. K., Kimmelman, A. C. & Debnath, J. Targeting autophagy in cancer: recent advances and future directions. *Cancer Discov.* **9**, 1167–1181 (2019). 31434711 10.1158/2159-8290.CD-19-0292 PMC7306856

[127] Debnath, J., Gammoh, N. & Ryan, K. M. Autophagy and autophagy-related pathways in cancer. *Nat. Rev. Mol. Cell Biol.* **24**, 560–575 (2023). 36864290 10.1038/s41580-023-00585-z PMC9980873

[128] Jacobson, D. H. et al. Multi-scale characterisation of homologous recombination deficiency in breast cancer. *Genome Med.* **15**, 90 (2023). 37919776 10.1186/s13073-023-01239-7 PMC10621207

[129] Zimmermann, M. et al. CRISPR screens identify genomic ribonucleotides as a source of PARP-trapping lesions. *Nature* **559**, 285–289 (2018). 29973717 10.1038/s41586-018-0291-z PMC6071917

[130] Jones, H. B. L. et al. obABPP-HT*: a precision-engineered activity proteomics pipeline for the streamlined discovery of deubiquitinase inhibitors. Preprint at *bioRxiv*10.1101/2025.05.27.656269 (2025).

[131] Li, W. et al. Quality control, modeling, and visualization of CRISPR screens with MAGeCK-VISPR. *Genome Biol.* **16**, 281 (2015). 26673418 10.1186/s13059-015-0843-6 PMC4699372

[132] Wang, B. et al. Integrative analysis of pooled CRISPR genetic screens using MAGeCKFlute. *Nat. Protoc.* **14**, 756–780 (2019). 30710114 10.1038/s41596-018-0113-7 PMC6862721

[133] Wu, T. et al. clusterProfiler 4.0: a universal enrichment tool for interpreting omics data. *Innovation* **2**, 100141 (2021). 34557778 10.1016/j.xinn.2021.100141 PMC8454663

[134] Kanehisa, M. et al. KEGG for taxonomy-based analysis of pathways and genomes. *Nucleic Acids Res.* **51**, D587–D592 (2022). 10.1093/nar/gkac963 PMC9825424 36300620

[135] Milacic, M. et al. The Reactome Pathway Knowledgebase 2024. *Nucleic Acids Res*. **52**, D672–d678 (2024). 37941124 10.1093/nar/gkad1025 PMC10767911

[136] Consortium, T. G. O. et al. The Gene Ontology knowledgebase in 2023. *Genetics* **224**, iyad031 (2023). 36866529 10.1093/genetics/iyad031 PMC10158837

[137] Meldal, B. H. M. et al. Complex Portal 2018: extended content and enhanced visualization tools for macromolecular complexes. *Nucleic Acids Res.* **47**, D550–d558 (2019). 30357405 10.1093/nar/gky1001 PMC6323931

