# Supplementary material for: Nucleophagy removes cytotoxic trapped PARP1
Source: Nat Cell Biol. 2026 Jun 2;28(6):1219–34. doi: 10.1038/s41556-026-01961-5 (PMC13278974; doi:10.1038/s41556-026-01961-5)
Supplement: Supplementary file 1 — Reporting Summary [file 41556_2026_1961_MOESM1_ESM.pdf]

nature portfolio

Corresponding author(s): Kristijan Ramadan

Last updated by author(s): Mar 31, 2026

Reporting Summary

Nature Portfolio wishes to improve the reproducibility of the work that we publish. This form provides structure for consistency and transparency in reporting. For further information on Nature Portfolio policies, see our Editorial Policies and the Editorial Policy Checklist.

Statistics

For all statistical analyses, confirm that the following items are present in the figure legend, table legend, main text, or Methods section.

n/a	Confirmed	
☐	☒	The exact sample size (*n*) for each experimental group/condition, given as a discrete number and unit of measurement
☐	☒	A statement on whether measurements were taken from distinct samples or whether the same sample was measured repeatedly
☐	☒	The statistical test(s) used AND whether they are one- or two-sided<br>*Only common tests should be described solely by name; describe more complex techniques in the Methods section.*
☐	☒	A description of all covariates tested
☐	☒	A description of any assumptions or corrections, such as tests of normality and adjustment for multiple comparisons
☐	☒	A full description of the statistical parameters including central tendency (e.g. means) or other basic estimates (e.g. regression coefficient) AND variation (e.g. standard deviation) or associated estimates of uncertainty (e.g. confidence intervals)
☐	☒	For null hypothesis testing, the test statistic (e.g. *F*, *t*, *r*) with confidence intervals, effect sizes, degrees of freedom and *P* value noted<br>*Give P values as exact values whenever suitable.*
☒	☐	For Bayesian analysis, information on the choice of priors and Markov chain Monte Carlo settings
☒	☐	For hierarchical and complex designs, identification of the appropriate level for tests and full reporting of outcomes
☐	☒	Estimates of effect sizes (e.g. Cohen's *d*, Pearson's *r*), indicating how they were calculated

Our web collection on statistics for biologists contains articles on many of the points above.

Software and code

Policy information about availability of computer code

Data collection	No code or software was used to collect data.
Data analysis	R software (R Core Team, 2024); R package clusterProfiler (DOI: 10.1016/j.xinn.2021.100141), MAGeCK Maximum Likelihood Estimation (MLE) module (DOI: 10.1186/s13059-015-0843-6); R package MAGeCKFlute (DOI: 10.1038/s41596-018-0113-7); ImageJ (NIH, https://imagej.net/Fiji/Downloads), CellProfiler (Broad Institute, https://cellprofiler.org/), GraphPad Prism (GraphPad Software, https://www.graphpad.com).

For manuscripts utilizing custom algorithms or software that are central to the research but not yet described in published literature, software must be made available to editors and reviewers. We strongly encourage code deposition in a community repository (e.g. GitHub). See the Nature Portfolio guidelines for submitting code & software for further information.

Data

Policy information about availability of data

All manuscripts must include a data availability statement. This statement should provide the following information, where applicable:

- Accession codes, unique identifiers, or web links for publicly available datasets
- A description of any restrictions on data availability
- For clinical datasets or third party data, please ensure that the statement adheres to our policy

All data generated, analysed and used in this study are included in this published article and its supplementary Information. Source data from published CRISPR screens is available in the European Nucleotide Archive under accession number PRJEB74933 (https://www.ebi.ac.uk/ena/browser/view/PRJEB74933) for data from

Dibitetto et al., (2024)73 and in Supplementary Table 1 of Noordermeer et al., (2018)30. Published mass spectrometry data39 is available in the ProteomeXchange Consortium via the PRIDE partner repository (dataset identifier PXD024337). Source data for RNA-seq experiments is deposited in Gene Expression Omnibus and can be accessed using the GEO accession number GSE277366. To review GEO accession GSE277366:
Go to https://www.ncbi.nlm.nih.gov/geo/query/acc.cgi?acc=GSE277366
Enter token ctmpawmobduxpqh into the box

Research involving human participants, their data, or biological material

Policy information about studies with human participants or human data. See also policy information about sex, gender (identity/presentation), and sexual orientation and race, ethnicity and racism.

Reporting on sex and gender	This information was not collected as no research was conducted using human participants, their data or primary biological material.
Reporting on race, ethnicity, or other socially relevant groupings	This information was not collected as no research was conducted using human participants, their data or primary biological material.
Population characteristics	This information was not collected as no research was conducted using human participants, their data or primary biological material.
Recruitment	This information was not collected as no research was conducted using human participants, their data or primary biological material.
Ethics oversight	No ethics approval was required for this study.

Note that full information on the approval of the study protocol must also be provided in the manuscript.

Field-specific reporting

Please select the one below that is the best fit for your research. If you are not sure, read the appropriate sections before making your selection.

☒ Life sciences ☐ Behavioural & social sciences ☐ Ecological, evolutionary & environmental sciences

For a reference copy of the document with all sections, see nature.com/documents/nr-reporting-summary-flat.pdf

Life sciences study design

All studies must disclose on these points even when the disclosure is negative.

Sample size	Sample size was not predetermined and varied based on experimental need. For microscopy experiments, a sample size was a minimum of 100 cells for each biological repeat, although this was often much higher due to higher availability of cells.
Data exclusions	No data were excluded from analyses.
Replication	All findings were replicated over at least two biological repeats, but more often over three. Three technical replicates were included for cell survival and viability experiments.
Randomization	This is not relevant to this study. To avoid bias during analysis, unbiased software such as CellProfiler was used where applicable.
Blinding	Blinding was not used in this study. To avoid bias during analysis, unbiased software was used wherever possible instead of manual analysis.

Reporting for specific materials, systems and methods

We require information from authors about some types of materials, experimental systems and methods used in many studies. Here, indicate whether each material, system or method listed is relevant to your study. If you are not sure if a list item applies to your research, read the appropriate section before selecting a response.

Materials & experimental systems

n/a	Involved in the study
☐	☒ Antibodies
☐	☒ Eukaryotic cell lines
☒	☐ Palaeontology and archaeology
☒	☐ Animals and other organisms
☐	☒ Clinical data
☒	☐ Dual use research of concern
☒	☐ Plants

Methods

n/a	Involved in the study
☒	☐ ChIP-seq
☐	☒ Flow cytometry
☒	☐ MRI-based neuroimaging

Antibodies

Antibodies used

Anti-PARP1 (E102), ab32138, Abcam; Anti-γH2AX (P-Ser139), 05-636, Millipore; Anti-53BP1, 4937S, Cell Signalling Technology; Anti-RPA32, 2208, Cell Signalling Technology; Anti-GFP antibody, ab290, Abcam; Anti-V5 antibody, R96025, Novex; donkey anti-Mouse Alexa Fluor 555, A-31570, Invitrogen; donkey anti-Rabbit Alexa Flour 488, A-21206, Invitrogen; Anti-HA (Y-11), sc-805, Santa Cruz; Anti-LC3B 2775S, Cell Signalling Technology; Anti-Atg7 [D12B11], 8558, Cell Signalling Technology; Anti-LAMP1, 21997-1-AP, Proteintech; Anti-TEX264, Gift Fielden et al (2020); Anti-Vinculin (7F9), sc-73614, Santa Cruz; Anti-p97, 10736-1-AP, Proteintech; Anti-histone H3, ab1791, Abcam; Anti-UFD1, 10615-1-AP, Proteintech; Anti-RNF4, NBP2-13243, Novus; Anti-STX17, HPA001204, Sigma.

Validation

Validation statements on manufacturer's websites are listed below:
Anti-PARP1 (E102), ab32138, Abcam: "Knockout Tested Rabbit Recombinant Monoclonal PARP1 antibody. Suitable for IHC-P, ICC/IF, WB, Flow Cyt (Intra) and reacts with Human samples. Cited in 133 publications."
nti-γH2AX (P-Ser139), 05-636, Millipore: "Anti-phospho-Histone H2A.X (Ser139), clone JBW301 is a well published Mouse Monoclonal Antibody validated in ChIP, ICC, IF, WB. This purified mAb is highly specific for phospho-Histone H2A.X (Ser139) also known as H2AXS139p"
Anti-53BP1, 4937S, Cell Signalling Technology: "53BP1 Antibody detects endogenous levels of total 53BP1 protein independent of phosphorylation."
Anti-RPA32, 2208, Cell Signalling Technology: "Monoclonal antibody is produced by immunizing animals with recombinant full-length human MBP-RPA32 protein. The antibody binds within the carboxy-terminal sequence of RPA32."
Anti-GFP antibody, ab290, Abcam: Rabbit Polyclonal GFP antibody. Suitable for ICC, IP, EM, ELISA, WB, IHC-FoFr, IHC-P, IHC-Fr, IHC-FrFl and reacts with Tag samples. Cited in 3369 publications. Immunogen corresponding to Recombinant Full Length Protein corresponding to Aequorea victoria GFP."
Anti-V5 antibody, R96025, Novex: "This Antibody was verified by Relative expression to ensure that the antibody binds to the antigen stated."
donkey anti-Mouse Alexa Fluor 555, A-31570: "Anti-Mouse secondary antibodies are affinity-purified antibodies with well-characterized specificity for mouse immunoglobulins and are useful in the detection, sorting or purification of its specified target."
donkey anti-Rabbit Alexa Flour 488, A-21206, Invitrogen: "Anti-Rabbit secondary antibodies are affinity-purified antibodies with well-characterized specificity for rabbit immunoglobulins and are useful in the detection, sorting or purification of its specified target"
Anti-HA (Y-11), sc-805, Santa Cruz: "epitope mapping within an internal region of the influenza hemagglutinin (HA) protein"
Anti-LC3B 2775S, Cell Signalling Technology: "LC3B detects endogenous levels of total LC3B protein. Cross-reactivity may exist with other LC3 isoforms. Stronger reactivity is observed with the type II form of LC3B."
Anti-Atg7 [D12B11], 8558, Cell Signalling Technology: "Atg7 (D12B11) Rabbit mAb recognizes endogenous levels of total Atg7 protein."
Anti-LAMP1, 21997-1-AP, Proteintech: "21997-1-AP targets CD107a / LAMP1 in WB, IHC, IP, CoIP, ELISA applications and shows reactivity with human samples."
Anti-TEX264, received as a gift but validated in Fielden et al (2020)
Anti-Vinculin (7F9), sc-73614, Santa Cruz: "vinculin Antibody (7F9) is an IgG1 κ mouse monoclonal vinculin antibody (also designated Metavinculin antibody or VCL antibody) that detects the vinculin protein of human, mouse, rat and avian origin by WB, IP, IF and IHC(P)"
Anti-p97, 10736-1-AP, Proteintech: "10736-1-AP targets VCP in WB, IHC, IF, IP, CoIP, ELISA applications and shows reactivity with human, mouse, rat samples."
Anti-histone H3, ab1791, Abcam: "A polyclonal rabbit antibody, supplied by Abcam, raised against Histone H3.1 (Human), cited in 5903 publications, with 245 published images. Applications used include WB, ChIP, IP, ICC-IF, and 17 others"
Anti-UFD1, 10615-1-AP, Proteintech: 10615-1-AP targets UFD1L in WB, IHC, IF/ICC, FC (Intra), IP, ELISA applications and shows reactivity with human, mouse samples."
Anti-RNF4, NBP2-13243, Novus: "A polyclonal rabbit antibody, supplied by Novus Biologicals (a Bio-Techne brand), raised against E3 ubiquitin-protein ligase RNF4 (Human), cited in 2 publications."
Anti-STX17, HPA001204, Sigma: A polyclonal rabbit antibody, supplied by Atlas Antibodies, raised against Syntaxin-17 (Human), cited in 32 publications."
Anti-mCherry [1C51], ab125096, Abcam: "Anti-mCherry antibody [1C51] (ab125096) is a House Mouse Monoclonal antibody and is validated for use in ICC/IF, IHC-P, WB in human samples...has been cited over 186 times in peer reviewed journals and is trusted by the scientific community."
Anti-alpha-Tubulin [TUBA1], T6199, Sigma: "...recognizes an epitope located at the C-terminal end of the α-tubulin isoform (amino acids 426-430) in a variety of organisms (e.g., human, bovine, mouse, and chicken). The antibody is specific for α-tubulin in immunoblotting assays and may be used for localization of α-tubulin in cultured cells or tissue sections."

Eukaryotic cell lines

Policy information about cell lines and Sex and Gender in Research

Cell line source(s)	CAL51 (DSMZ, ACC 302), HeLa (ATCC, CCL-2), MDA-MB231 (ATCC, Htb-26) RPE hTERT (ATCC, CRL-4000). Cell lines previously generated in other studies are indicated in materials and methods. No primary cell lines were used.
Authentication	Cell lines had been authenticated at the point of purchase from ATCC and DSMZ.
Mycoplasma contamination	All cell lines were regularly tested for mycoplasmsa.
Commonly misidentified lines (See ICLAC register)	No misidentified cell lines were used in the study.

Clinical data

Policy information about clinical studies

All manuscripts should comply with the ICMJE guidelines for publication of clinical research and a completed CONSORT checklist must be included with all submissions.

Clinical trial registration	*Provide the trial registration number from ClinicalTrials.gov or an equivalent agency.*
Study protocol	*Note where the full trial protocol can be accessed OR if not available, explain why.*
Data collection	*Describe the settings and locales of data collection, noting the time periods of recruitment and data collection.*
Outcomes	*Describe how you pre-defined primary and secondary outcome measures and how you assessed these measures.*

Plants

Seed stocks	This is not relevant to this study.
Novel plant genotypes	This is not relevant to this study.
Authentication	This is not relevant to this study

Flow Cytometry

Plots

Confirm that:

- [x] The axis labels state the marker and fluorochrome used (e.g. CD4-FITC).
- [x] The axis scales are clearly visible. Include numbers along axes only for bottom left plot of group (a 'group' is an analysis of identical markers).
- [x] All plots are contour plots with outliers or pseudocolor plots.
- [x] A numerical value for number of cells or percentage (with statistics) is provided.

Methodology

Sample preparation	Cal51 cells were seeded in 6-well plates and treated with the appropriate drugs for 18 hours. Cells were harvested with trypsin, washed in PBS, fixed with 4% formaldehyde, and permeabilised with Triton X-100 + EDTA. Cells were transferred through the cell strainer cap of a FACS tube then stained with PROTEOSTAT® Aggresome Red Detection Reagent.
Instrument	LSRFortessa X-20
Software	BD FACSDiva, FlowJo
Cell population abundance	As no cell sorting was performed, the abundance of ProteoStat-positive cells was quantified directly within the total analyzed population. Cells exhibiting protein aggregation were identified based on increased ProteoStat fluorescence intensity relative to the unstained control, which was used to define the negative population. A positive control (MG-132-treated cells) was included to confirm assay sensitivity and establish the expected range of increased aggregation.

Gating strategy	Cells were initially gated based on forward and side scatter (FSC-A versus SSC-A) to exclude debris. Doublets were excluded using FSC-A versus FSC-H gating. As no viability dye was used, all singlet events within the FSC/SSC gate were included in downstream analysis. ProteoStat fluorescence was analyzed within the singlet cell population. The boundary between ProteoStat-negative and -positive populations was defined using the unstained control, which established baseline autofluorescence. This threshold was applied consistently across all samples within each experiment. A positive control (MG-132-treated cells) was used to verify detection of increased protein aggregation and to confirm appropriate placement of the positive population. Aggregate levels were quantified as the median fluorescence intensity (MFI) of the ProteoStat signal.

☒ Tick this box to confirm that a figure exemplifying the gating strategy is provided in the Supplementary Information.
