# Supplementary material for: Nucleophagy removes cytotoxic trapped PARP1
Source: Nat Cell Biol. 2026 Jun 2;28(6):1219–34. doi: 10.1038/s41556-026-01961-5 (PMC13278974; doi:10.1038/s41556-026-01961-5)

Source Data for Figure 2

Figure 2B-C

Western blot from experiment 3. Right is with overlay to show ladders and red box shows area shown in figure.

1: HeLa WT untreated

2: HeLa TMEM192-3HA untreated

3: HeLa TMEM192-3HA Talazoparib + MMS

4: HeLa TMEM192-3HA Bafilomycin

5: HeLa TMEM192-3HA Tala + MMS + Baf

Quantification	#1	#2	#3
Utd	1	1	1
Tala+MMS	1.975648	1.007922	1.58148
Baf	1.450773	1.144468	1.217272
Tala+MMS+Baf	2.745941	1.944671	2.22748

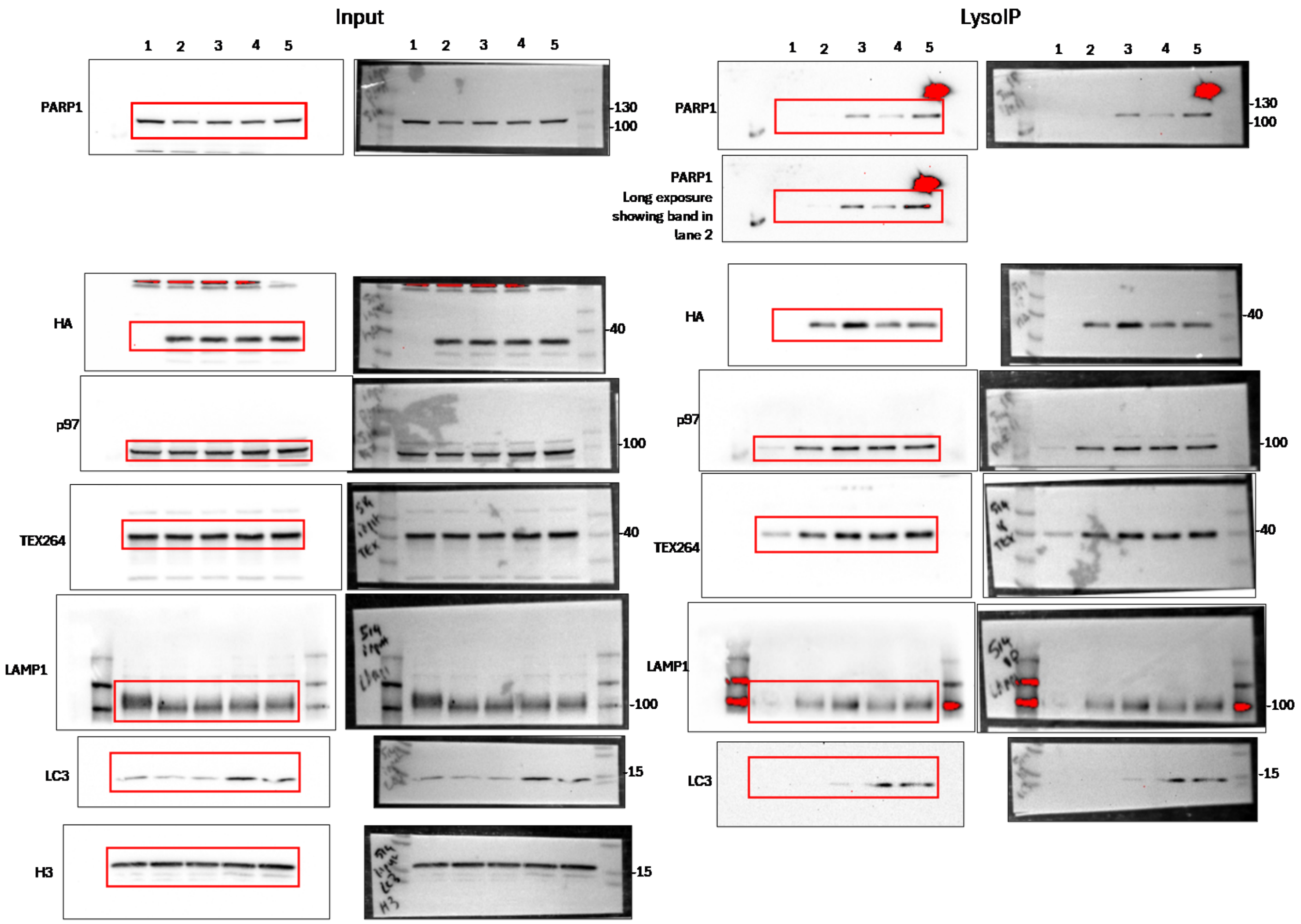

Figure 2D-E

Western blot from experiment 3. Right is with overlay to show ladders and red box shows area shown in figure.

1: HeLa WT untreated

2: HeLa TMEM192-3HA Bafilomycin

3: HeLa TMEM192-3HA Talazoparib + Bafilomycin

4: HeLa TMEM192-3HA Veilparib + Bafilomycin

Quantification	#1	#2	#3	#4	#5
Baf	0.682699	0.20325	0.297084	0.101615	0.826798
Tala + Baf	1	1	1	1	1
Veli + Baf	0.613434	0.73892	0.186708	0.325285	0.758966

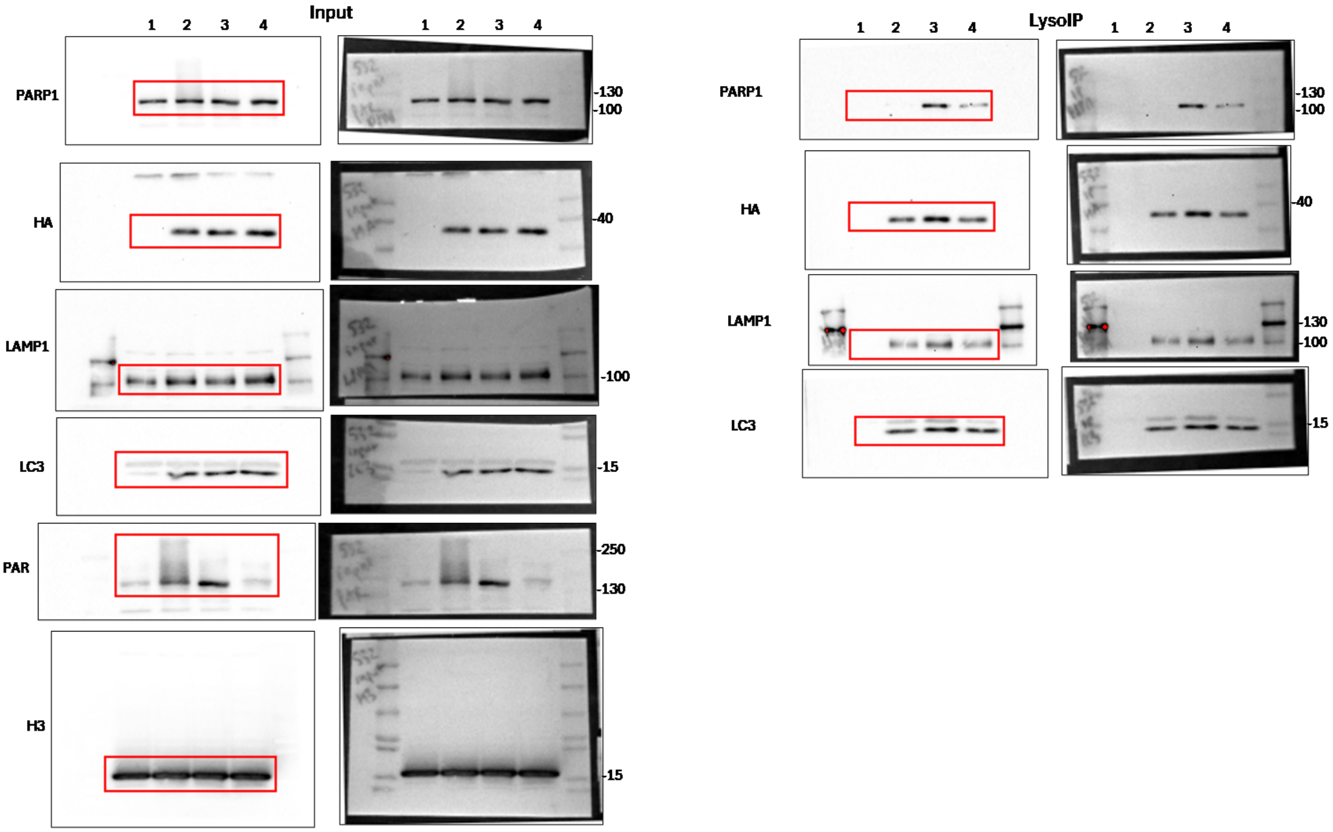

Supplement: Supplementary file 7 — Unprocessed western blots. [file 41556_2026_1961_MOESM7_ESM.pdf]
