# Supplementary material for: Nucleophagy removes cytotoxic trapped PARP1
Source: Nat Cell Biol. 2026 Jun 2;28(6):1219–34. doi: 10.1038/s41556-026-01961-5 (PMC13278974; doi:10.1038/s41556-026-01961-5)
Supplement: Supplementary file 9 — Unprocessed western blots. [file 41556_2026_1961_MOESM9_ESM.pdf]

Source Data for Figure 3

Figure 3A-B

Right is with membrane overlay to show ladder. Red box shows area in figure

1: HeLa WT
2: HeLa TMEM siCtrl Baf
3: HeLa TMEM siCtrl Tala + MMS + Baf
4: HeLa TMEM siATG7 Baf
5: HeLa TMEM siATG7 Tala + MMS + Baf

	siLUC Baf	siLUC TM+Baf	siATG7 Baf	siATG7 TM+Baf
#1	0.024211863	1	0.039003809	0.01618846
#2	0.523687094	1	0.304538197	0.62795553
#3	0.052012306	1	0.122999945	0.20851869

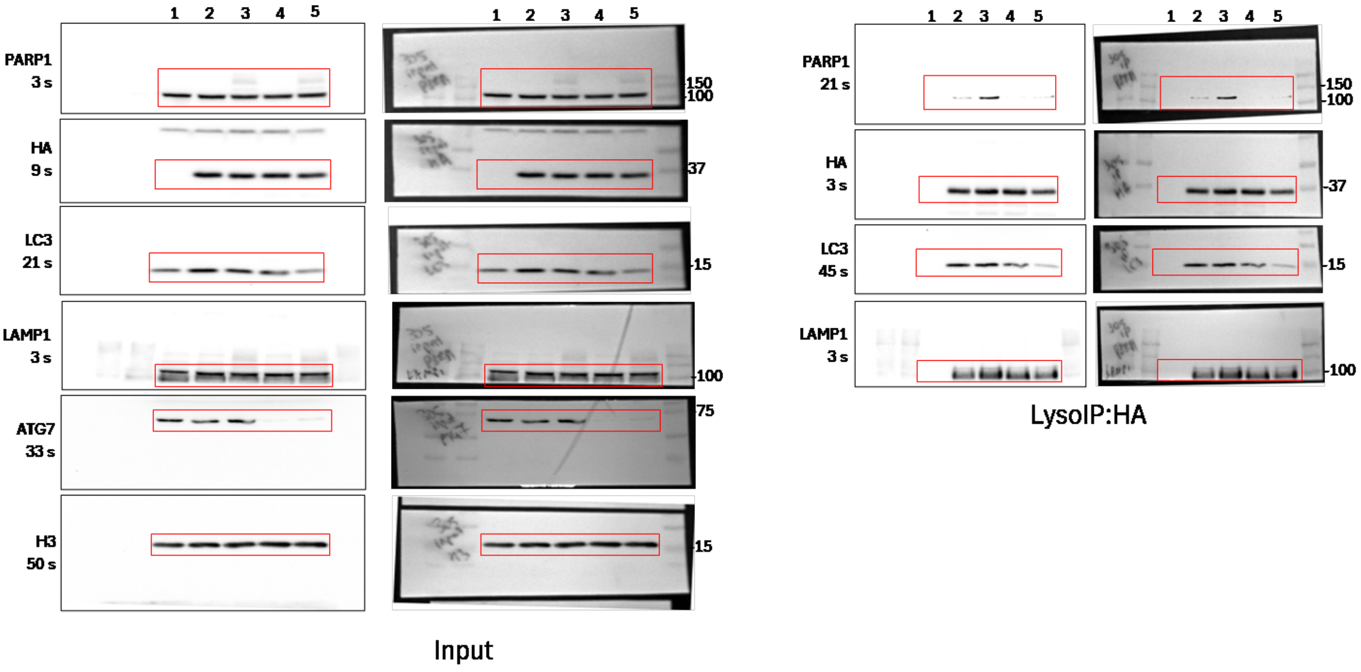

Figure 3C-D

Right is with membrane overlay to show ladder. Red box shows area in figure

1: HeLa WT

2: HeLa TMEM Baf

3: HeLa TMEM Tala + MMS + Baf

4: HeLa TMEM STX17-KD Baf

5: HeLa TMEM STX17-KD Tala + MMS + Baf

PARP1 lysoIP signal normalised by dividing by HA and normalised to ctrl Tala + MMS + Baf	EXP	Baf	Tala + MMS + Baf	STX17-KD Baf	STX17 Tala + MMS + Baf
	#1	0.654518667	1	0.374618821	0.354039698
	#2	0.54404476	1	0.21588215	0.382849657
	#3	0.238351627	1	0.070728897	0.24215132

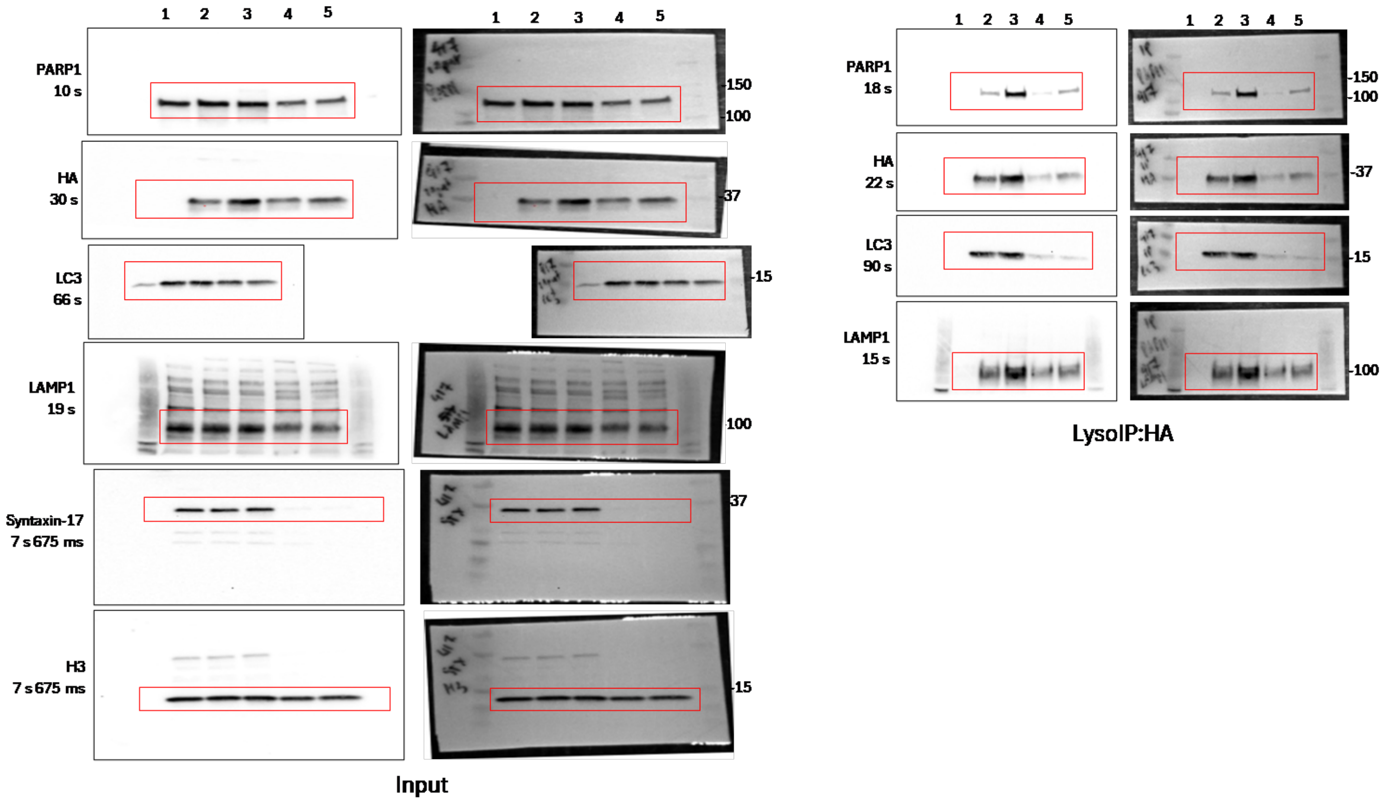

Figure 3G-H

Right is with membrane overlay to show ladder. Red box shows area in figure

1: HeLa WT

2: HeLa TMEM Baf

3: HeLa TMEM Tala + MMS + Baf

4: HeLa TMEM CB-5083 Baf

5: HeLa TMEM CB-5083 Tala + MMS + Baf

PARP1 lysoIP signal normalised by dividing by HA and normalised to ctrl Tala + MMS + Baf				
EXP	Baf	Tala + MMS + Baf	CB-5083 + Baf	CB-5083 + Baf + Tala + MMS
#1	0.307519	1	0.251040546	0.186009843
#2	0.604777	1	0.304855427	0.155004671
#3	0.06445	1	0.420326803	0.384421437

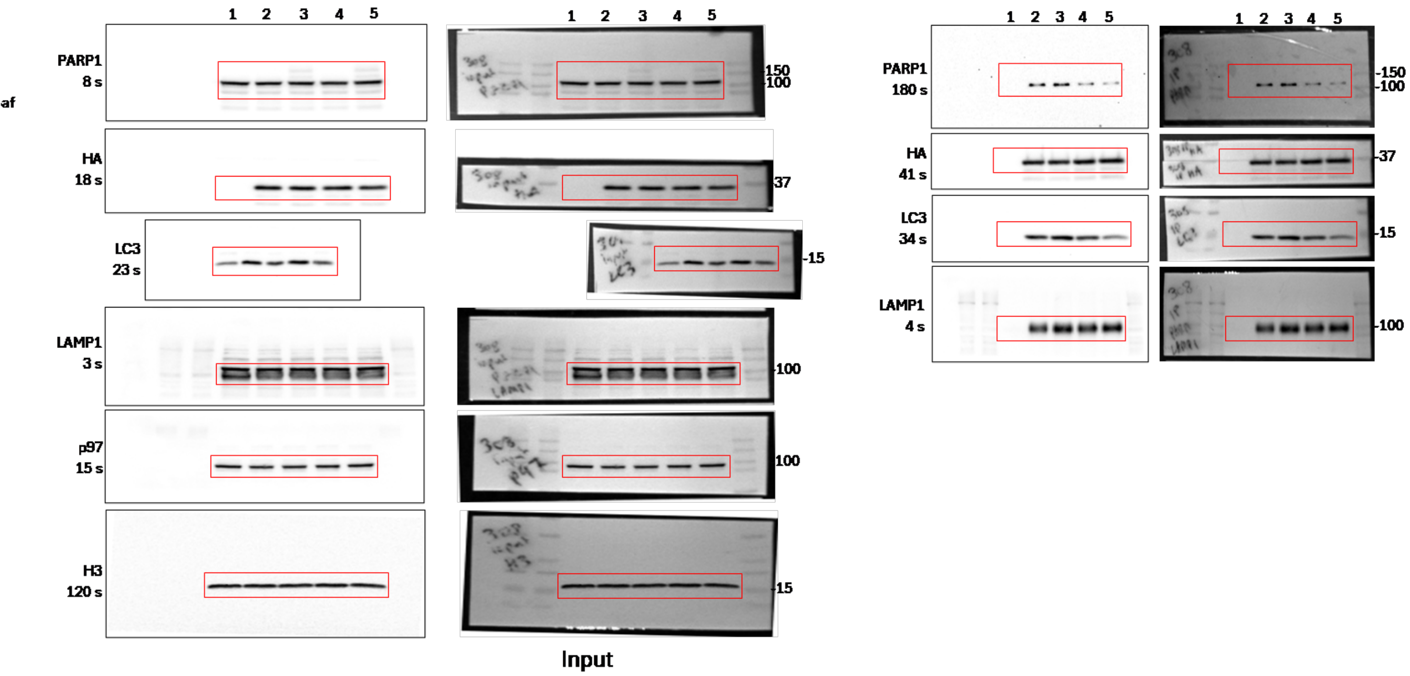

Figure 3I-J

Right is with membrane overlay to show ladder. Red box shows area in figure

1: HeLa WT

2: HeLa TMEM Baf

3: HeLa TMEM Tala + MMS + Baf

4: HeLa TMEM MLN-7243 Baf

5: HeLa TMEM MLN-7243 Tala + MMS + Baf

6: HeLa TMEM ML-792 Baf

7: HeLa TMEM ML-792 Tala + MMS + Baf

PARP1 lysoIP signal normalised by dividing by HA and normalised to ctrl Tala + MMS + Baf						
EXP	Baf	Tala + MMS + Baf	MLN-7243 Baf	MLN-7243 Tala + MMS + Baf	ML-792 Baf	ML-792 Tala + MMS + Baf
#1	0.130350431	1	0.403320303	0.076682274	0.298894811	0.070895469
#2	0.196057313	1	0.417827581	0.24582099	0.248546728	0.196640513
#3	0.263200476	1	0.447420225	0.371473092	0.481335727	0.44020558
#4	0.181184579	1	0.25043734	0.387382819	0.584839996	0.591046902

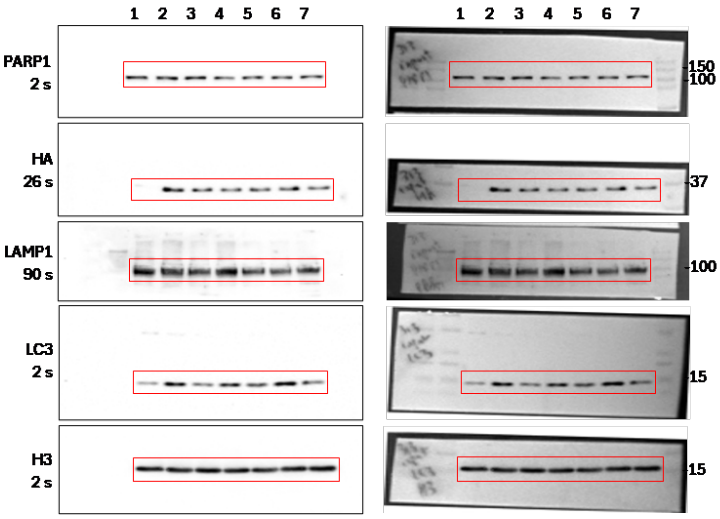

Input

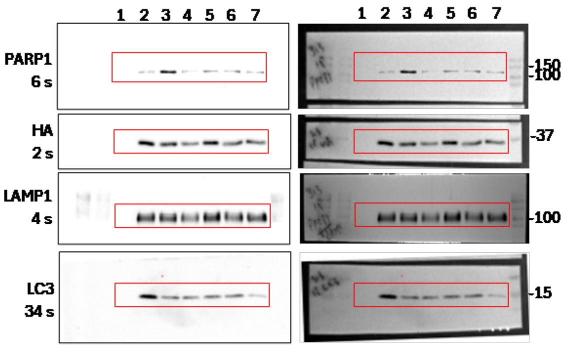

LysoIP:HA
