# Supplementary material for: Nucleophagy removes cytotoxic trapped PARP1
Source: Nat Cell Biol. 2026 Jun 2;28(6):1219–34. doi: 10.1038/s41556-026-01961-5 (PMC13278974; doi:10.1038/s41556-026-01961-5)

Source Data for Figure 4

Figure 4C
Right is with membrane overlay to show ladder. Red box shows area in figure
1: HeLa WT
2: HeLa TEX264-/-

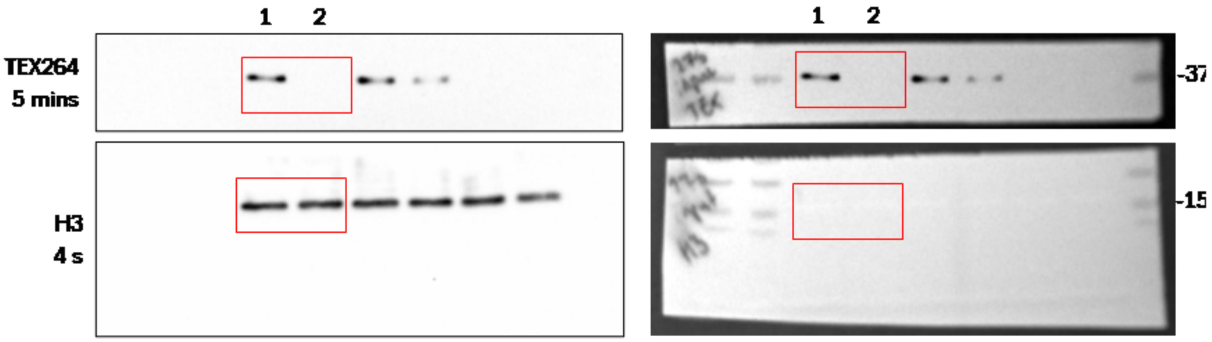

Figure 4D
Right is with membrane overlay to show ladder. Red box shows area in figure
1: CAL51 WT
2: CAL51 TEX264-/-

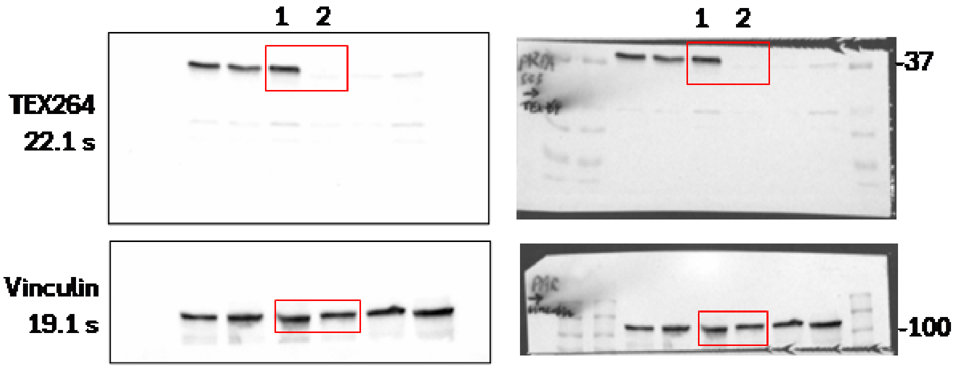

Figure 4G
1: HeLa PARP1-WT siLUC
2: HeLa PARP1-WT siTEX
3: HeLa PARP1-res siLUC
4: HeLa PARP1-res siTEX

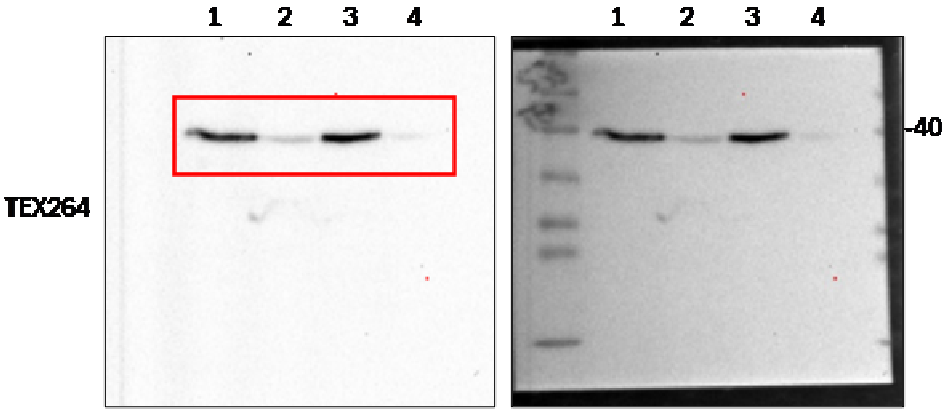

Figure 4J
Right is with membrane overlay to show ladder. Red box shows area in figure
1: CAL51 siCtrl
2: CAL51 siUFD1
3: CAL51 siTEX264 #1
4: CAL51 siTEX264 #2

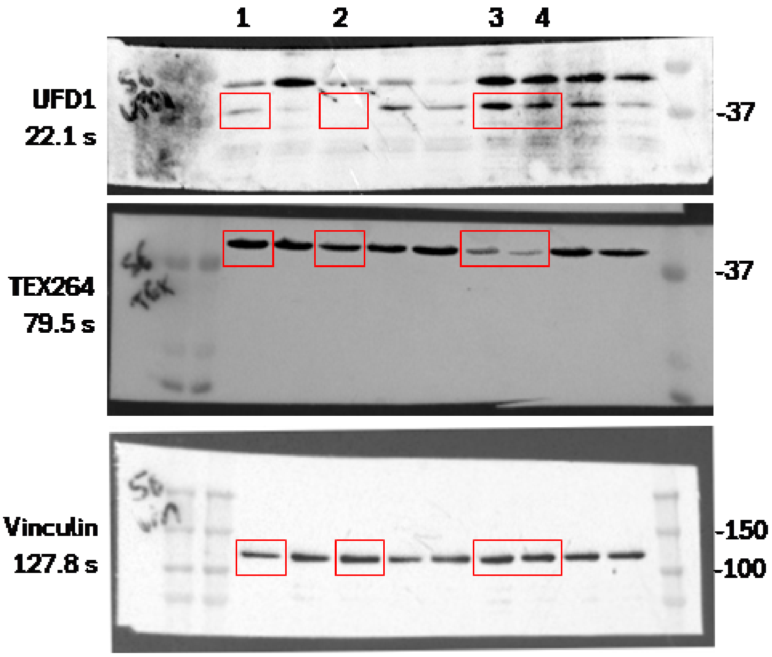

Supplement: Supplementary file 11 — Unprocessed western blots. [file 41556_2026_1961_MOESM11_ESM.pdf]
