# Supplementary material for: Nucleophagy removes cytotoxic trapped PARP1
Source: Nat Cell Biol. 2026 Jun 2;28(6):1219–34. doi: 10.1038/s41556-026-01961-5 (PMC13278974; doi:10.1038/s41556-026-01961-5)

Source Data for Figure 5

Figure 5A

Right is with membrane overlay to show ladder. Red box shows area in figure

1: CAL51 WT
2: CAL51 PARP1-GFP siLUC untreated
3: CAL51 PARP1-GFP siLUC Tala + MMS
4: CAL51 PARP1-GFP siUFD1 Tala + MMS
5: CAL51 PARP1-GFP siTEX264 Tala + MMS

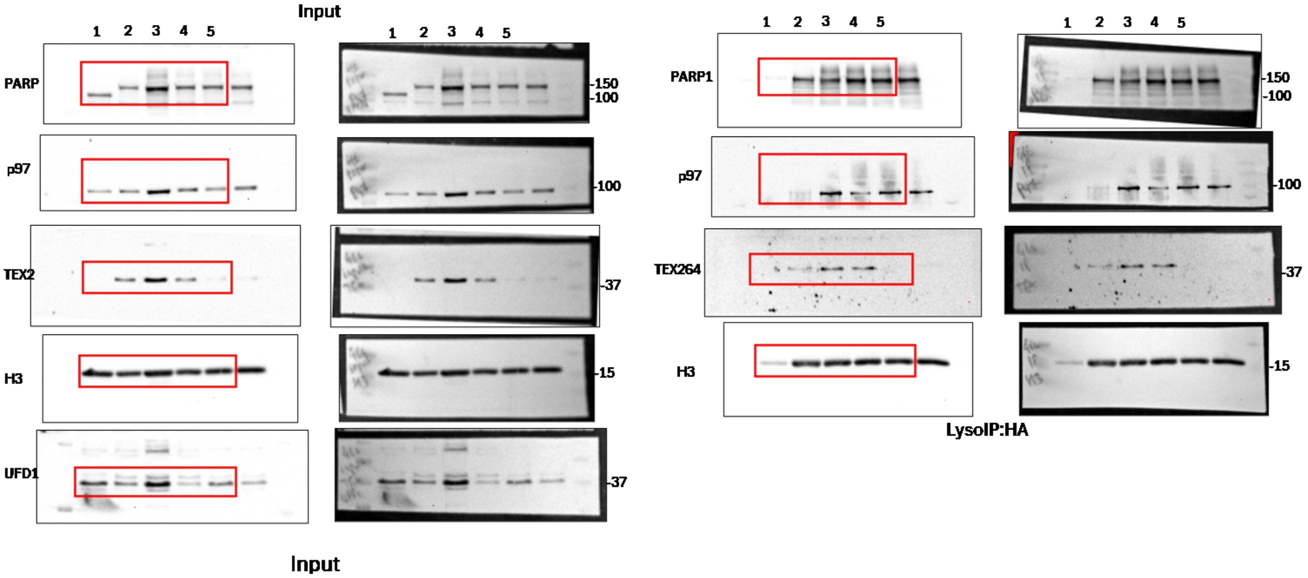

Figure 5E

Right is with membrane overlay to show ladder. Red box shows area in figure

1: HeLa (PARP1-GFP) + TEX264-wt
2: HeLa (PARP1-GFP) + TEX264-D272-end

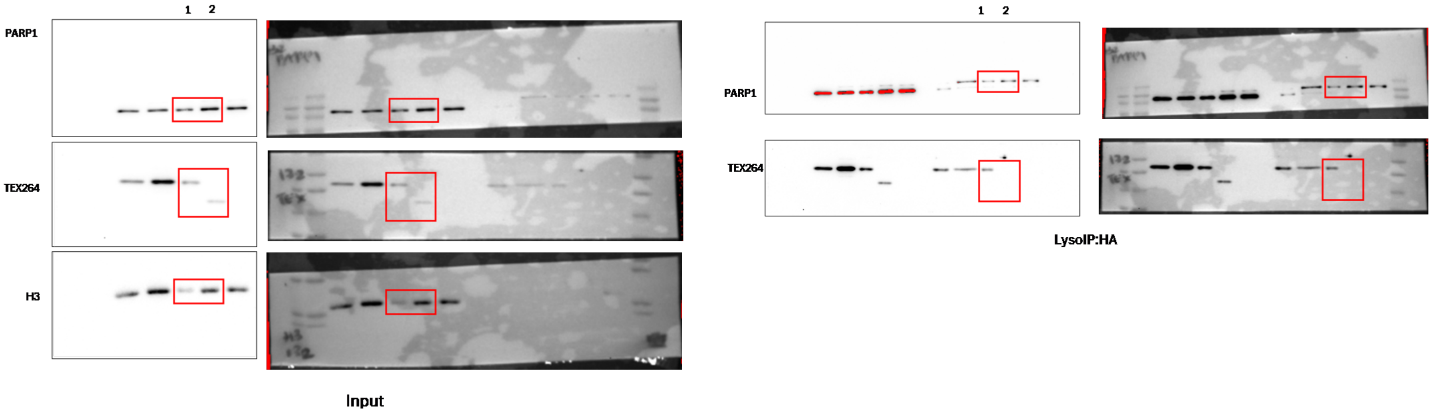

Figure 5H-I

Right is with membrane overlay to show ladder. Red box shows area in figure

1: HeLa WT
2: HeLa TMEM Baf
3: HeLa TMEM Tala + MMS + Baf
4: HeLa TMEM TEX264-/- Baf
5: HeLa TMEM TEX264-/- Tala + MMS + Baf
6: HeLa TMEM TEX264-/- TEX-WT Baf
7: HeLa TMEM TEX264-/- TEX-WT Tala + MMS + Baf
8: HeLa TMEM TEX264-/- TEX-SHP Baf
9: HeLa TMEM TEX264-/- TEX-SHP Tala + MMS + Baf
10: HeLa TMEM TEX264-/- TEX-LIR Baf
11: HeLa TMEM TEX264-/- TEX-LIR Tala + MMS + Baf

PARP1 signal normalised to HA then against ctrl TM+BAF					
EXP	WT Tala + MMS + Baf	KO Tala + MMS + Baf	KO+WT Tala + MMS + Baf	KO + SHP Tala + MMS + Baf	KO + LIR Tala + MMS + Baf
#1	1	0.198226946	0.788537611	0.024821441	0.380003357
#2	1	0.454615826	1.450437767	0.447278042	0.224710739
#3	1		1.622367021	0.315016849	0.026818418

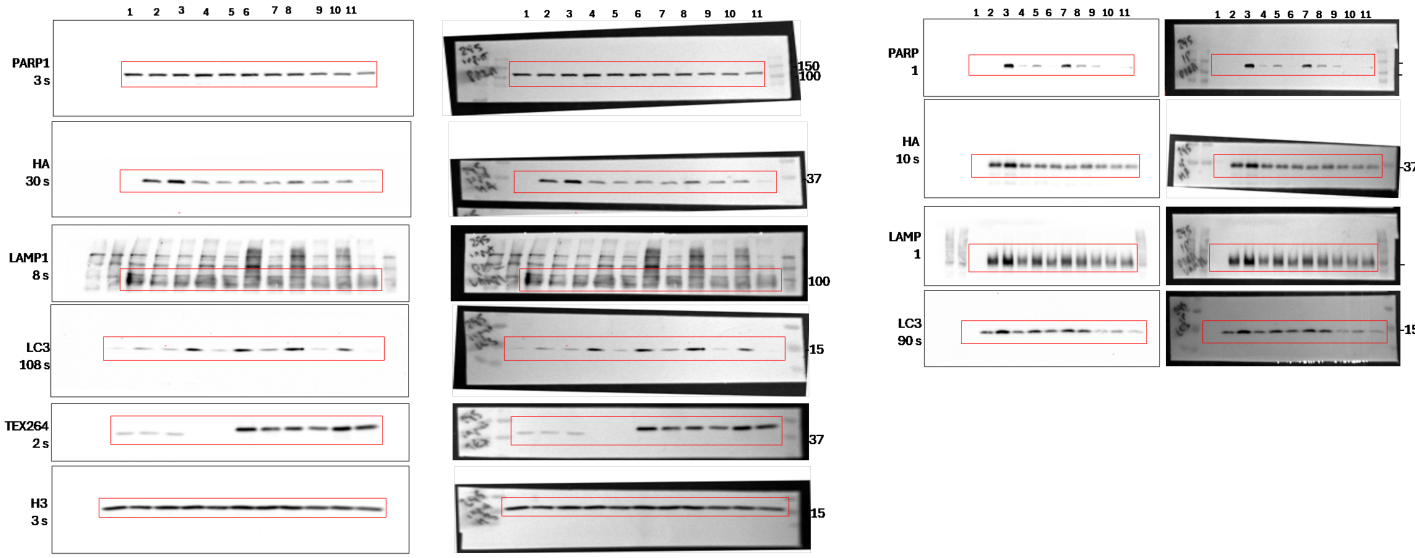

Supplement: Supplementary file 13 — Unprocessed western blots. [file 41556_2026_1961_MOESM13_ESM.pdf]
