# Supplementary material for: Nucleophagy removes cytotoxic trapped PARP1
Source: Nat Cell Biol. 2026 Jun 2;28(6):1219–34. doi: 10.1038/s41556-026-01961-5 (PMC13278974; doi:10.1038/s41556-026-01961-5)

Source Data for Figure 6

Figure 6E

Right is with membrane overlay to show ladder. Red box shows area in figure

1 PARP1-WT utd
2 PARP1-WT Tala + MMS
3 PARP1-WT Baf
4 PARP1-WT Tala + MMS + Baf
5 PARP1-KS Baf
6 PARP1-KS Baf + Tala + MMS

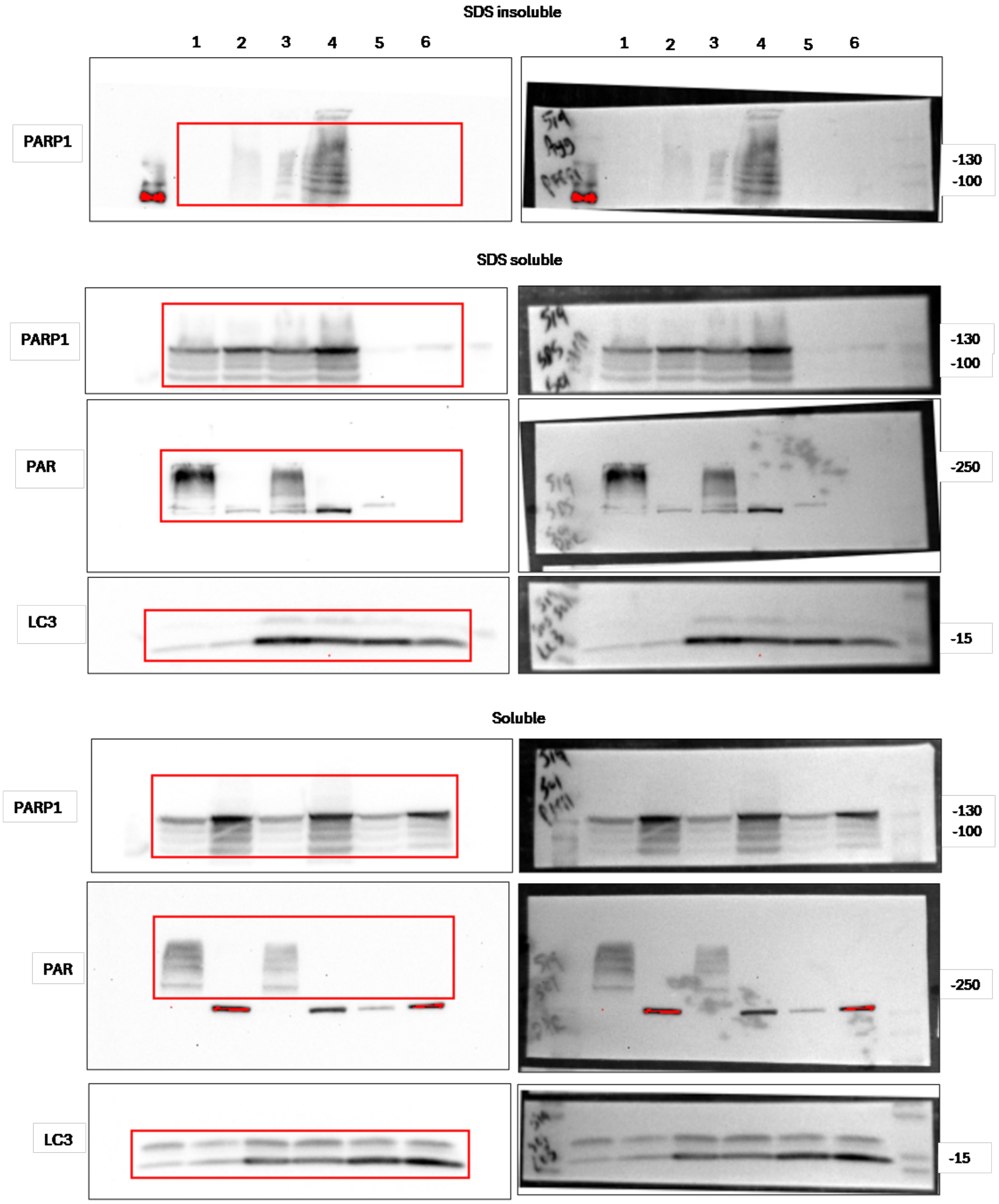

Supplement: Supplementary file 15 — Unprocessed western blots. [file 41556_2026_1961_MOESM15_ESM.pdf]
