# Supplementary material for: Nucleophagy removes cytotoxic trapped PARP1
Source: Nat Cell Biol. 2026 Jun 2;28(6):1219–34. doi: 10.1038/s41556-026-01961-5 (PMC13278974; doi:10.1038/s41556-026-01961-5)

Source Data for Extended Data Figure 2

Extended Data Figure 2B

Right is with membrane overlay to show ladder. Red box shows area in figure

1: HeLa WT
2: HeLa shATG7 +Dox
3: HeLa STX17-KD

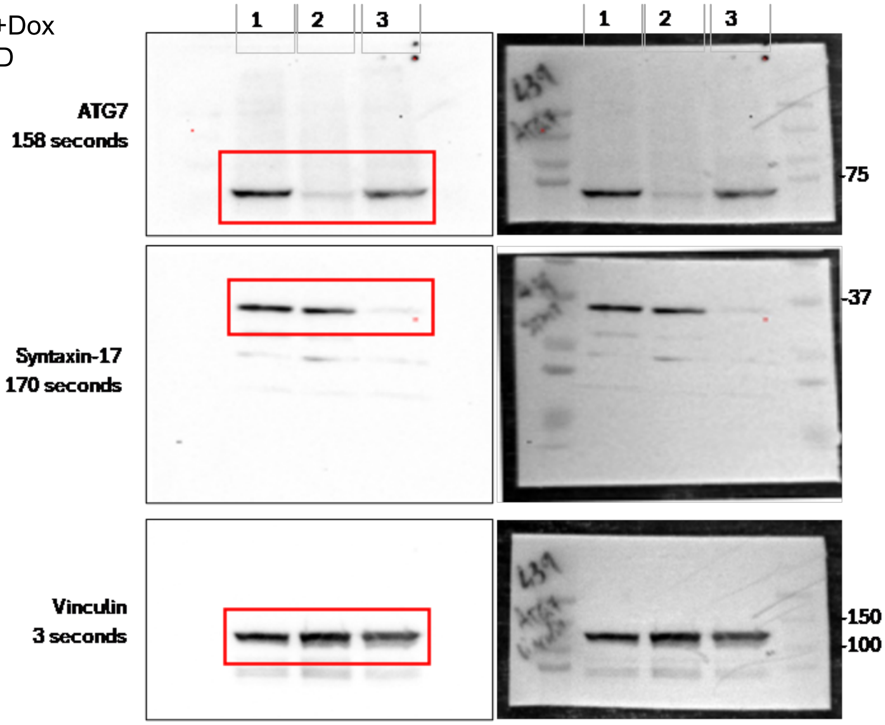

Extended Data Figure 2D

Right is with membrane overlay to show ladder. Red box shows area in figure

1: siLUC
2: siATG9A

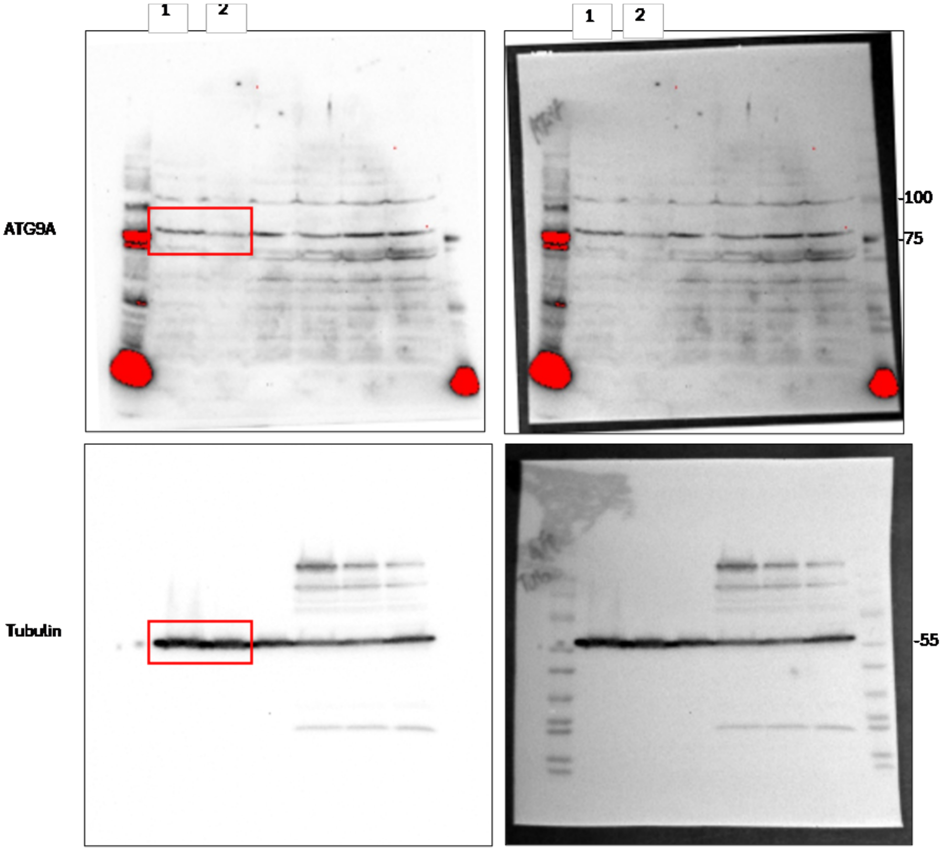

Extended Data Figure 2E

Right is with membrane overlay to show ladder. Red box shows area in figure

1: HeLa WT untreated

2: HeLa WT Talazoparib

3: HeLa WT Talazoparib + Bafilomycin

4: HeLa WT Talazoparib + Torin

5: HeLa TEX264-KO untreated

6: HeLa TEX264-KO Talazoparib

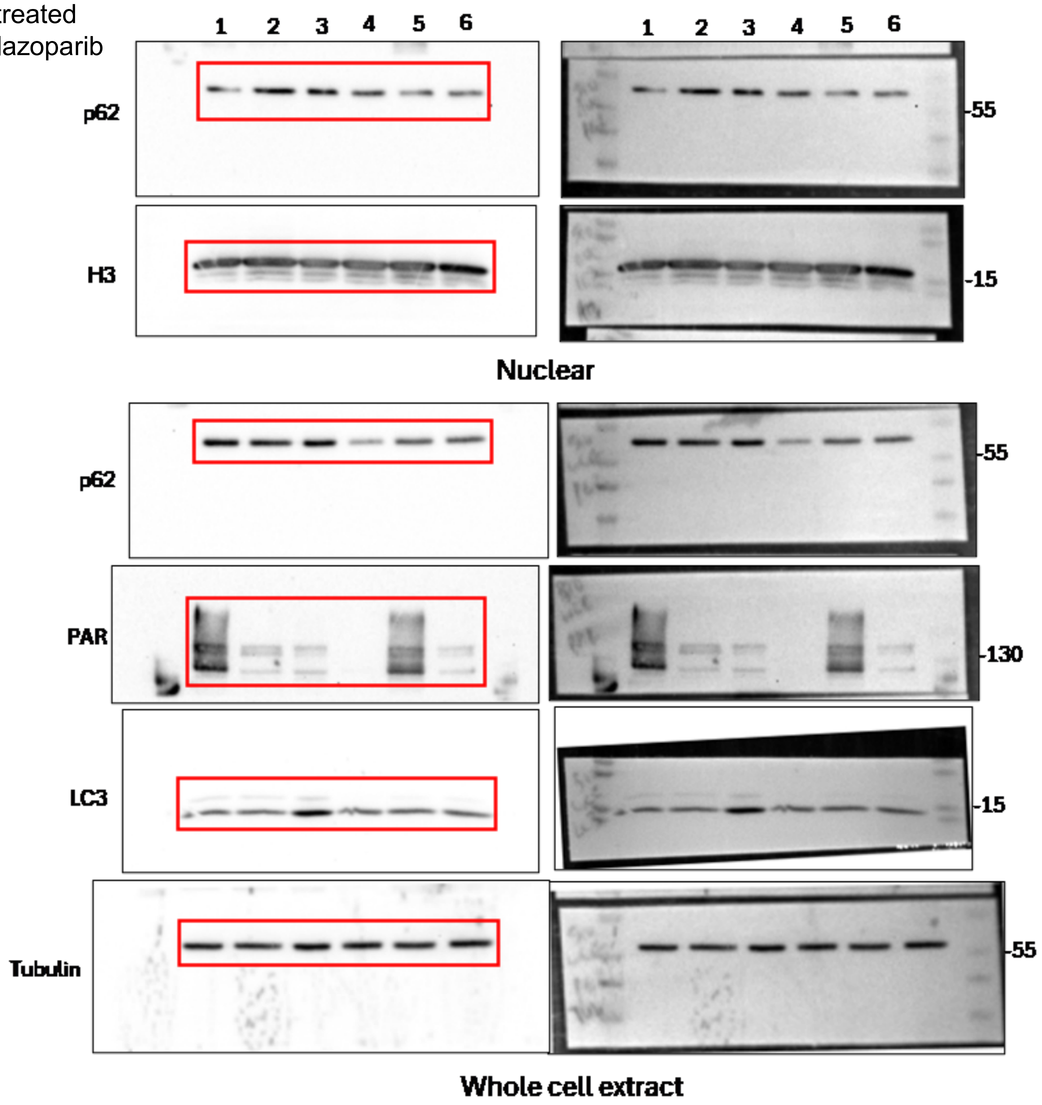

Extended Data Figure 2F

Right is with membrane overlay to show ladder. Red box shows area in figure

1: siCtrl

2: sip97

3: siATG7

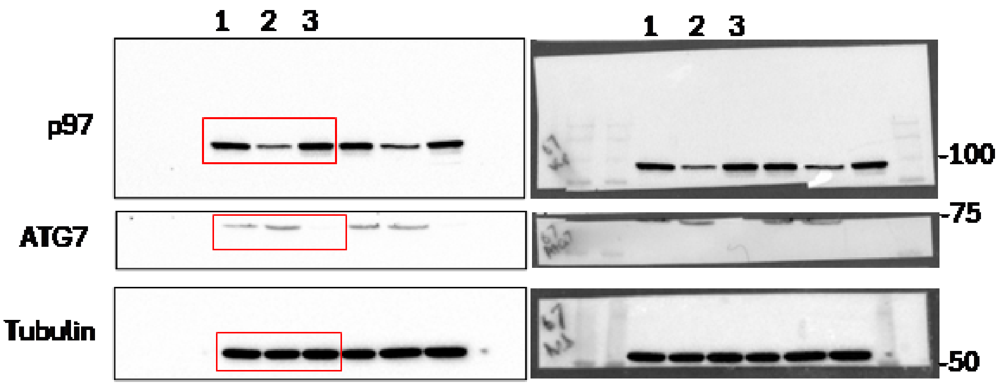

Supplement: Supplementary file 17 — Unprocessed western blots. [file 41556_2026_1961_MOESM17_ESM.pdf]
