# Supplementary material for: Nucleophagy removes cytotoxic trapped PARP1
Source: Nat Cell Biol. 2026 Jun 2;28(6):1219–34. doi: 10.1038/s41556-026-01961-5 (PMC13278974; doi:10.1038/s41556-026-01961-5)

Source Data for Extended Data Figure 3

Extended Data Figure 3A

Right is with membrane overlay to show ladder. Red box shows area in figure

1: CAL51 PARP1-WT
2: CAL51 PARP1-KS
3: CAL51 PARP1-WT TMEM Baf
4: CAL51 PARP1-WT TMEM Tala + MMS + Baf
5: CAL51 PARP1-KS Baf
6: CAL51 PARP1-KS Tala + MMS + Baf

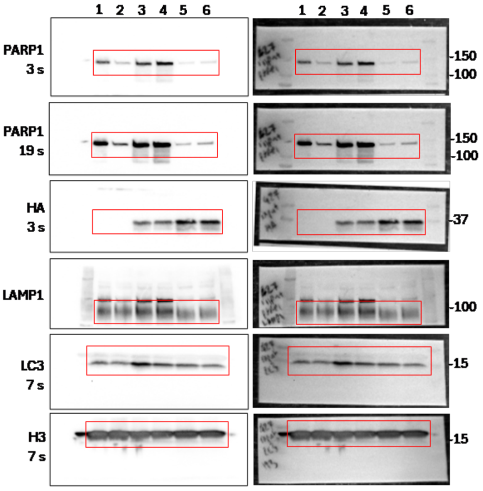

Input

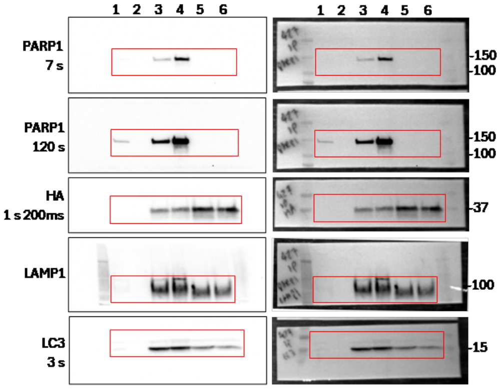

LysoIP:HA

Extended Data Figure 3C-D

LysoIP with Niraparib in HeLa WT and TMEM192-3HA cells

Lane	Sample
1	WT untreated
2	TMEM BAF
3	TMEM Baf + Tala + MMS
4	TMEM + Baf + Nira + MMS

Quantification

Exp	Baf	Nira+MMS+Baf
#1	1	1.365
#2	1	1.485
#3	1	1.745

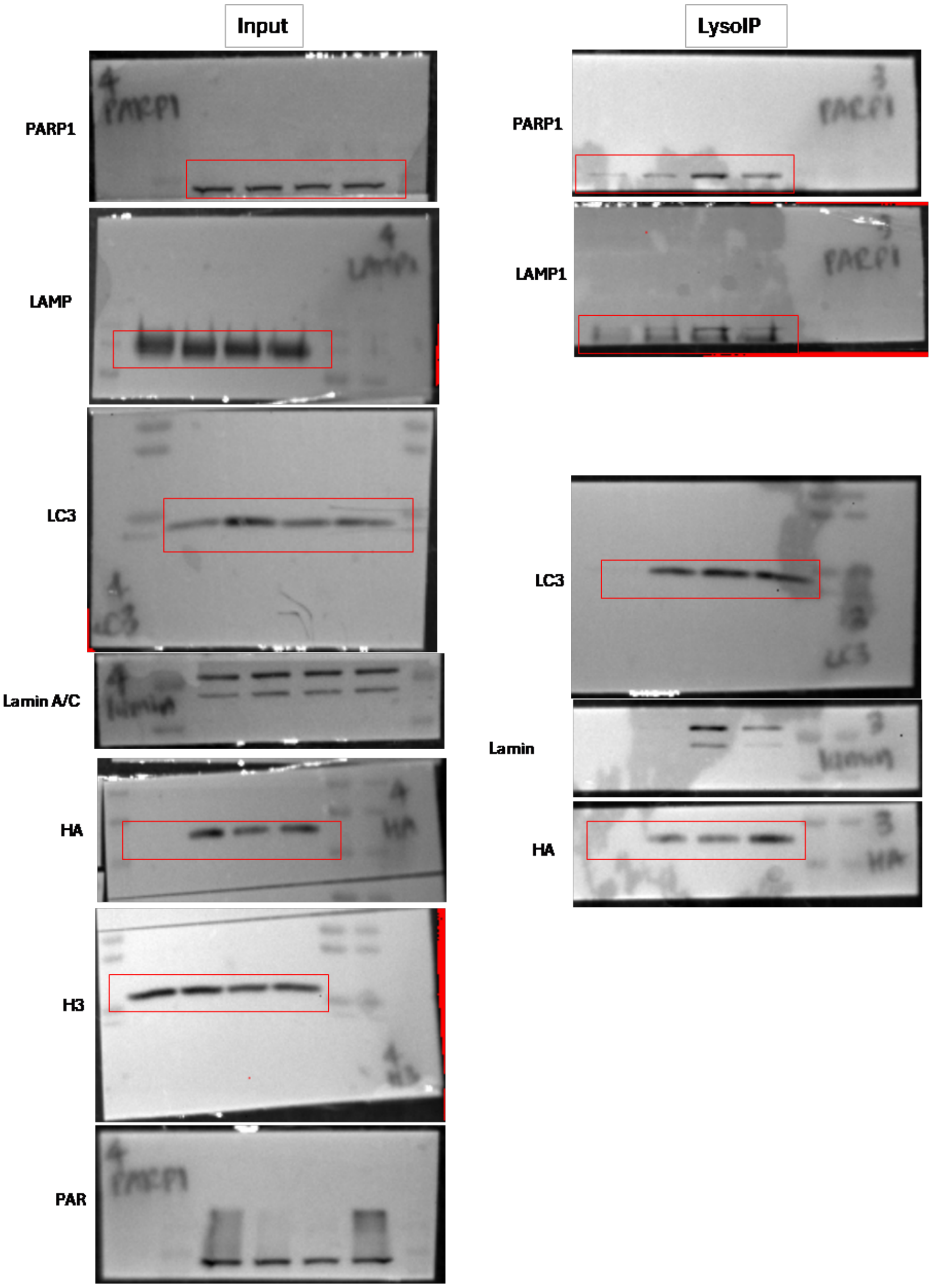

Extended Data Figure 3E

1 HeLa WT no transfection untreated
2 HeLa WT no transfection Baf
3 HeLa WT no transfection Tala
4 HeLa WT no transfection Tala + Baf
5 HeLa WT mCherry-PARP1-GFP transfection untreated
6 HeLa WT mCherry-PARP1-GFP transfection Baf
7 HeLa WT mCherry-PARP1-GFP transfection Tala
8 HeLa WT mCherry-PARP1-GFP transfection Tala + Baf

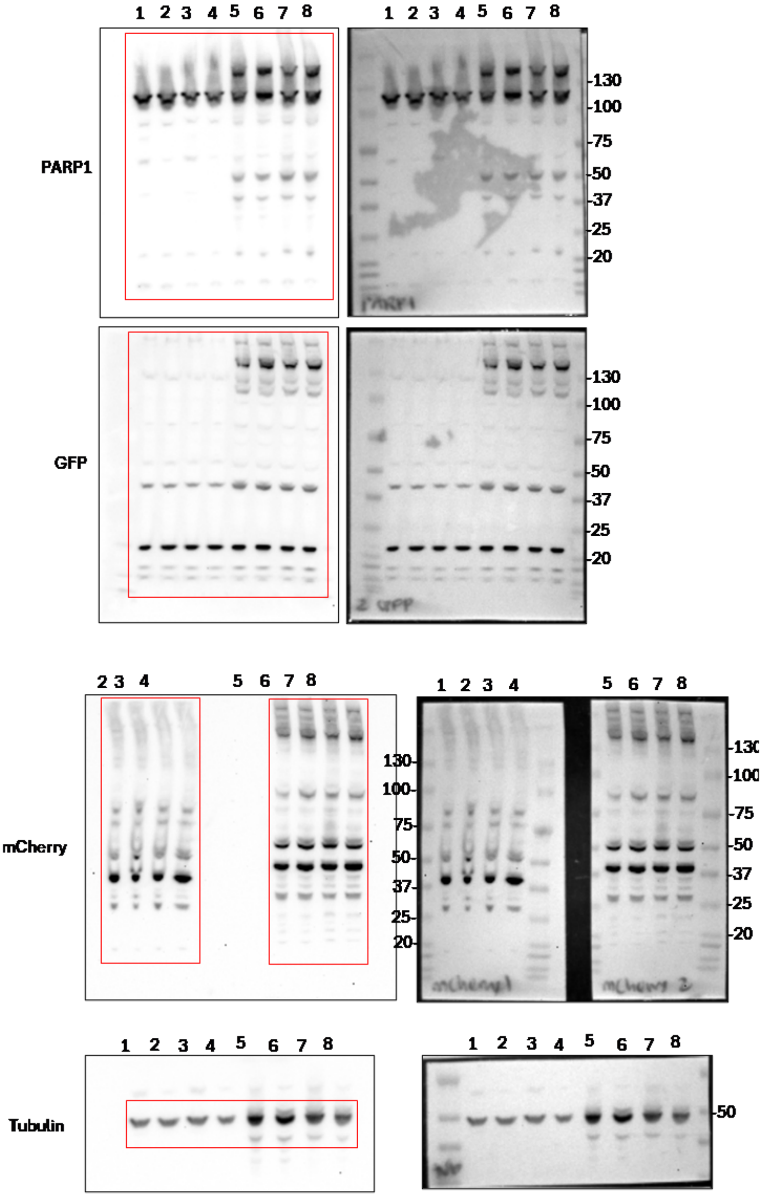

Extended Data Figure 3H-I

Right is with membrane overlay to show ladder. Red box shows area in figure

1: HeLa WT

2: HeLa TMEM Baf

3: HeLa TMEM Tala + MMS + Baf

4: HeLa TMEM RNF4-DN Baf

5: HeLa TMEM RNF4-DN Tala + MMS + Baf

6: HeLa TMEM RNF4-WT Baf

7: HeLa TMEM RNF4-WT Tala + MMS + Baf

PARP1 lysoIP signal normalised by dividing by HA and normalised to ctrl Tala + MMS + Baf						
EXP	Baf	Tala + MMS + Baf	RNF4-WT Baf	RNF4-WT Tala + MMS + Baf	RNF4-DN Baf	RNF4-DN Tala + MMS + Baf
#1	0.248613	1	0.331741011	1.263494267	0.442110216	1.00462233
#2	0.165883	1	0.163411082	1.224882573	0.281089823	1.18141173
#3	0.146166	1	0.247003775	0.348352331	0.073934914	1.053123182

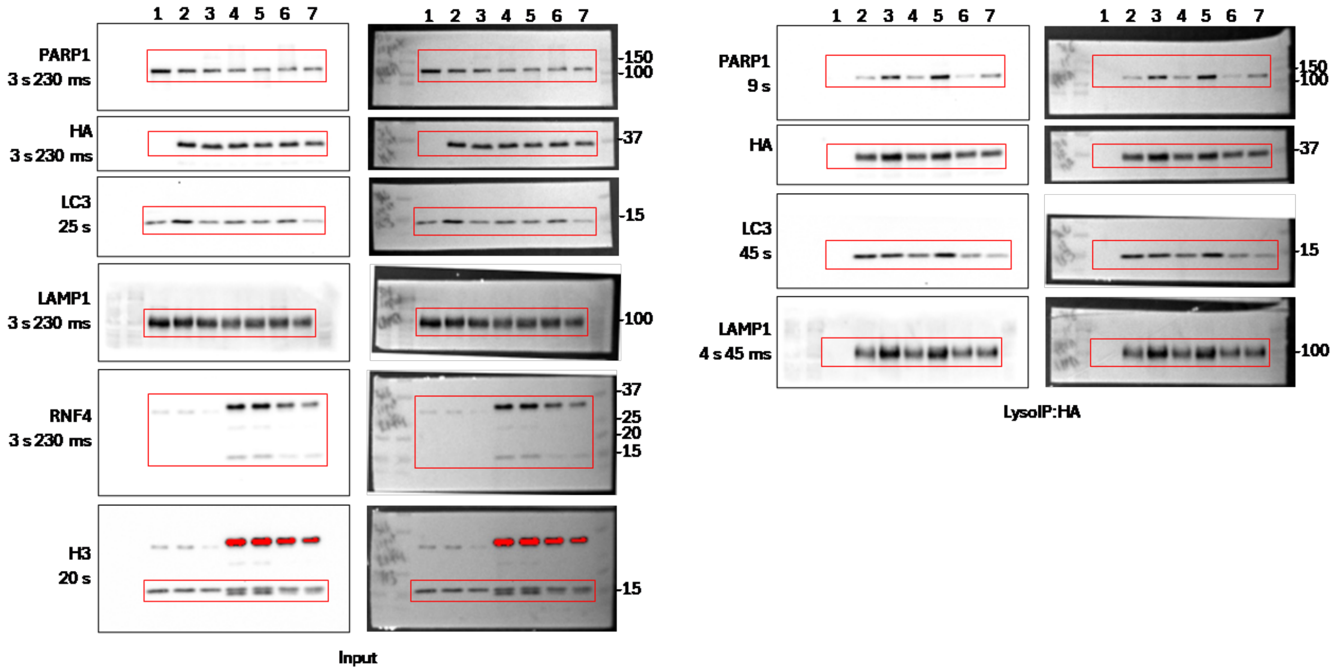

Extended Data Figure 3J-K

Right is with membrane overlay to show ladder. Red box shows area in figure

1: HeLa WT
2: HeLa TMEM siCtrl Baf
3: HeLa TMEM siCtrl Tala + MMS + Baf
4: HeLa TMEM siUFD1 #1 Baf
5: HeLa TMEM siUFD1 #1 Tala + MMS + Baf
6: HeLa TMEM siUFD1 #2 Baf
7: HeLa TMEM siUFD1 #2 Tala + MMS + Baf

PARP1 signal normalised to HA then against ctrl TM+BAF						
EXP	siCtrl Baf	siCtrl Tala + MMS + Baf	siUFD1 #1 Baf	siUFD1 #1 Tala + MMS + Baf	siUFD1 #2 Baf	siUFD1 #2 Tala + MMS + Baf
#1	0.424693	1	1.149272	2.031093	1.550054	0.574903
#2	0.430323	1	0.218841	0.841891	0.656325	0.334081
#3	0.136243	1	0.439811	0.952825	0.93208	1.916915

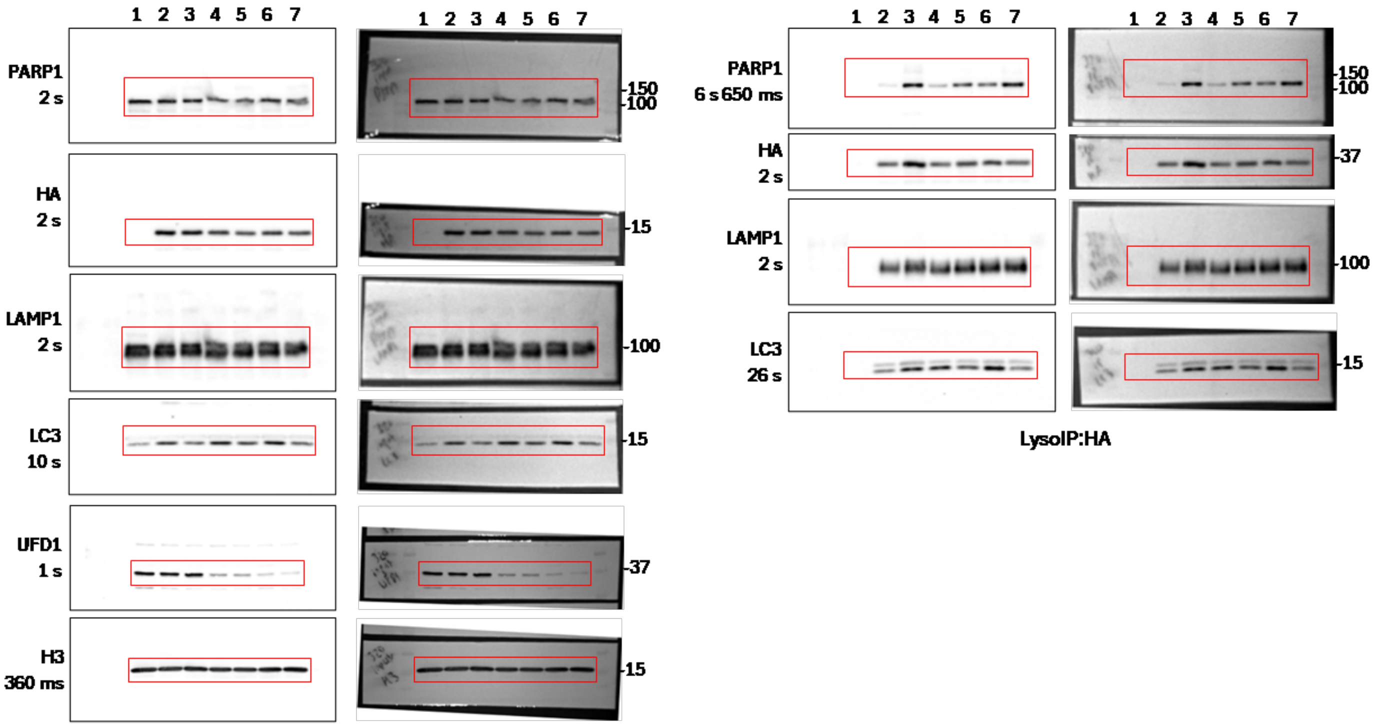

Supplement: Supplementary file 19 — Unprocessed western blots. [file 41556_2026_1961_MOESM19_ESM.pdf]
