# Supplementary material for: Nucleophagy removes cytotoxic trapped PARP1
Source: Nat Cell Biol. 2026 Jun 2;28(6):1219–34. doi: 10.1038/s41556-026-01961-5 (PMC13278974; doi:10.1038/s41556-026-01961-5)

Source Data for Extended Data Figure 4

Extended Data Figure 4D
Right is with membrane overlay to show ladder. Red box shows area in figure
1: MDA-MB231 siCtrl
2: MDA-MB231 siUFD1
3: MDA-MB231 siTEX264 #1
4: MDA-MB231 siTEX264 #2

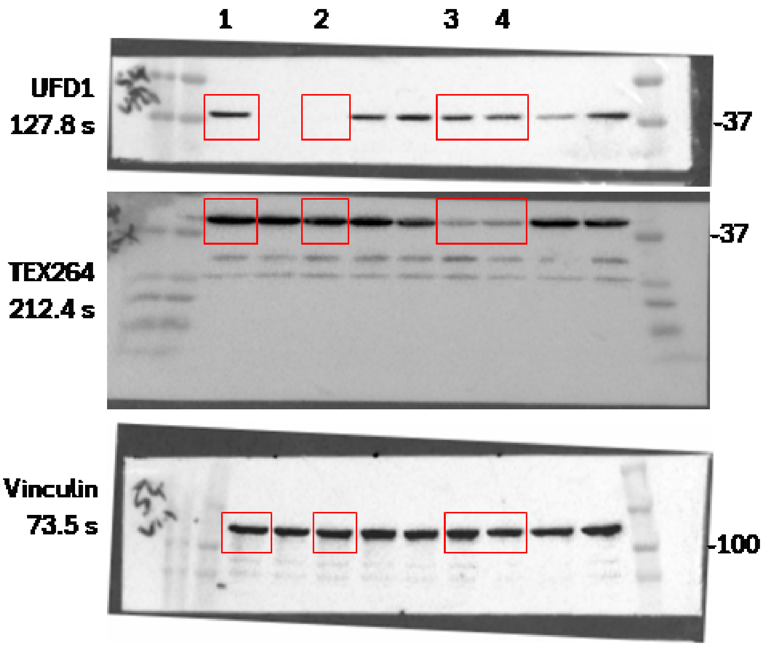

Extended Data Figure 4F
Right is with membrane overlay to show ladder. Red box shows area in figure
1: HeLa siCtrl
2: HeLa siUFD1
3: HeLa TEX264-/-

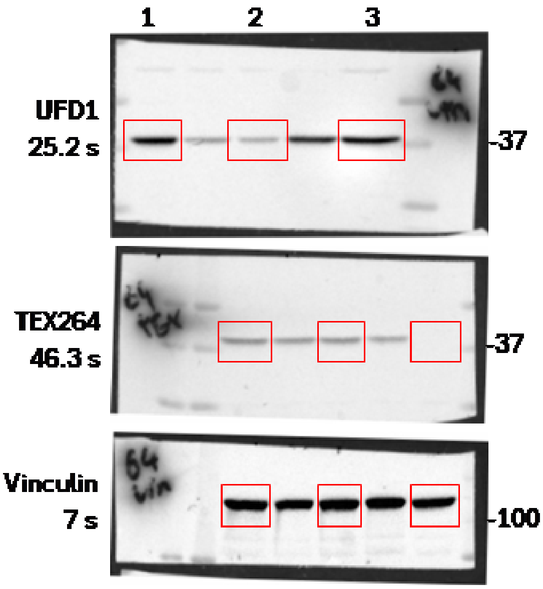

Supplement: Supplementary file 21 — Unprocessed western blots. [file 41556_2026_1961_MOESM21_ESM.pdf]
