# Supplementary material for: Nucleophagy removes cytotoxic trapped PARP1
Source: Nat Cell Biol. 2026 Jun 2;28(6):1219–34. doi: 10.1038/s41556-026-01961-5 (PMC13278974; doi:10.1038/s41556-026-01961-5)

Source Data for Extended Data Figure 5

Extended Data Figure 5A

Right is with membrane overlay to show ladder. Red box shows area in figure

1 TEX264

2 PARP1 + TEX264

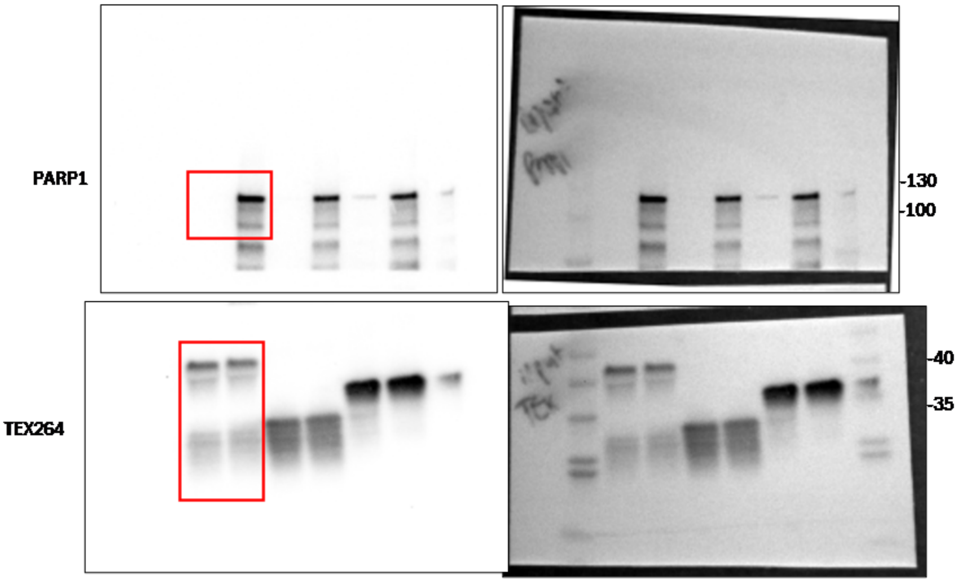

Extended Data Figure 5C Right is with membrane overlay to show ladder. Red box shows area in figure

1 Input: TEX264-WT

2 Input: PARP1 + TEX264-WT

3 Input: PARP1 + TEX264-C-term

4 Input: PARP1 + TEX264-N-term

5 IP: TEX264-WT

6 IP: PARP1 + TEX264-WT

7 IP: PARP1 + TEX264-C-term

8 IP: PARP1 + TEX264-N-term

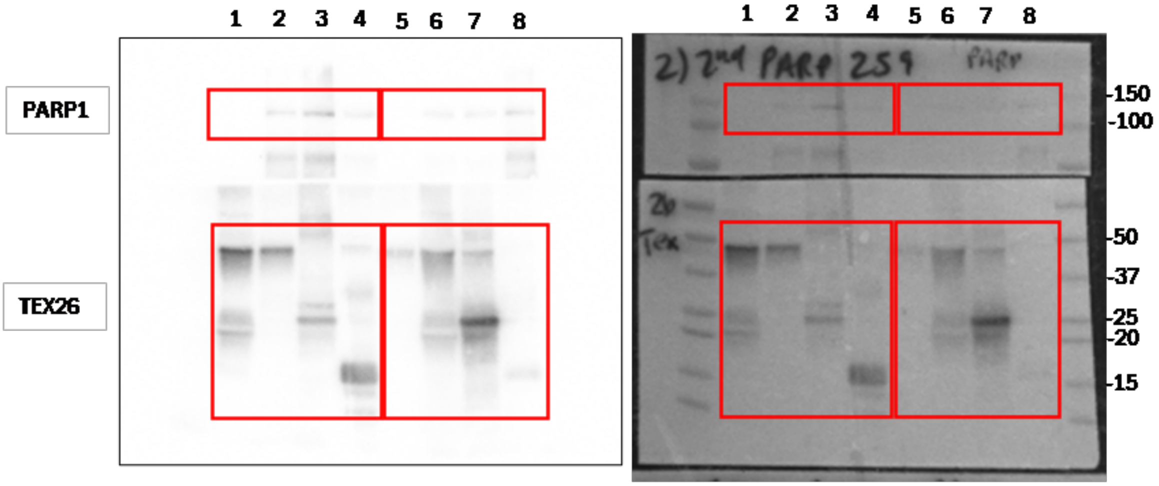

Extended Data Figure 5D

Right is with membrane overlay to show ladder. Red box shows area in figure

1	HeLa WT utd
2	HeLa PARP1-GFP TEX264-WT utd
3	HeLa P1-GFP TEX264-WT Tala+MMS
4	HeLa P1-GFP TEX264-Dhelix Tala+MMS

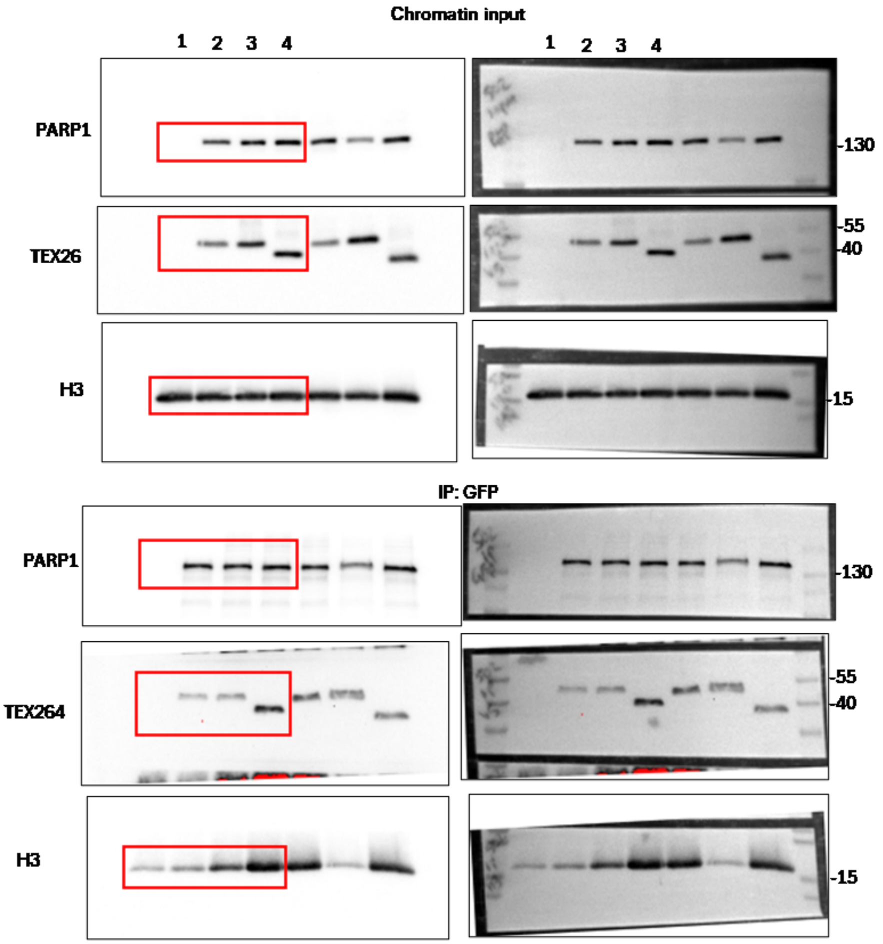

Extended Data Figure 5F

Right is with membrane overlay to show ladder. Red box shows area in figure

1: CAL51 WT
2: CAL51 TEX264-/-
3: CAL51 TEX264-/- +TEX-WT
4: CAL51 TEX264-/- +TEX-SHP*
5: CAL51 TEX264-/- +TEX-LIR*

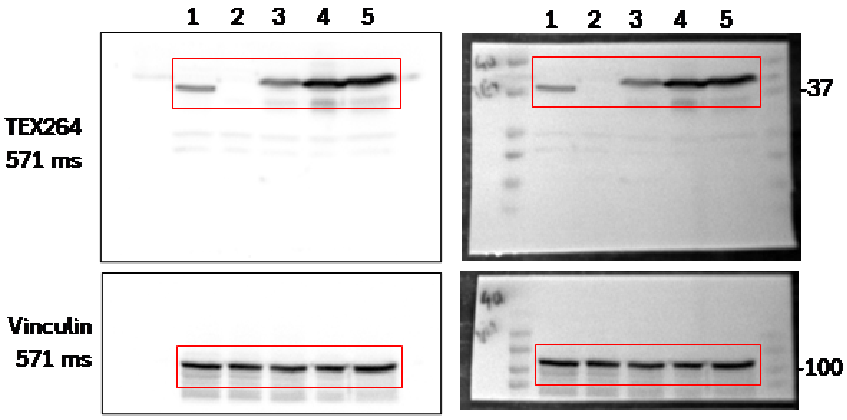

Supplement: Supplementary file 23 — Unprocessed western blots. [file 41556_2026_1961_MOESM23_ESM.pdf]
