# Supplementary material for: Nucleophagy removes cytotoxic trapped PARP1
Source: Nat Cell Biol. 2026 Jun 2;28(6):1219–34. doi: 10.1038/s41556-026-01961-5 (PMC13278974; doi:10.1038/s41556-026-01961-5)

Source Data for Extended Data Figure 6

Extended Data Figure 6B-C

LysoIP with nuclear pore inhibitor Leptomycin B in HeLa WT and TMEM192-3HA cells

Lane	Sample
1	WT utd
2	TMEM Baf
3	TMEM BAF + Tala + MMS
4	TMEM Baf + Leptomycin B
5	TMEM Baf + Tala + MMS + Leptomycin B

Exp	Baf + TM	Baf + LeptB + TM
#1	1	1.568
#2	1	1.34
#3	1	0.955

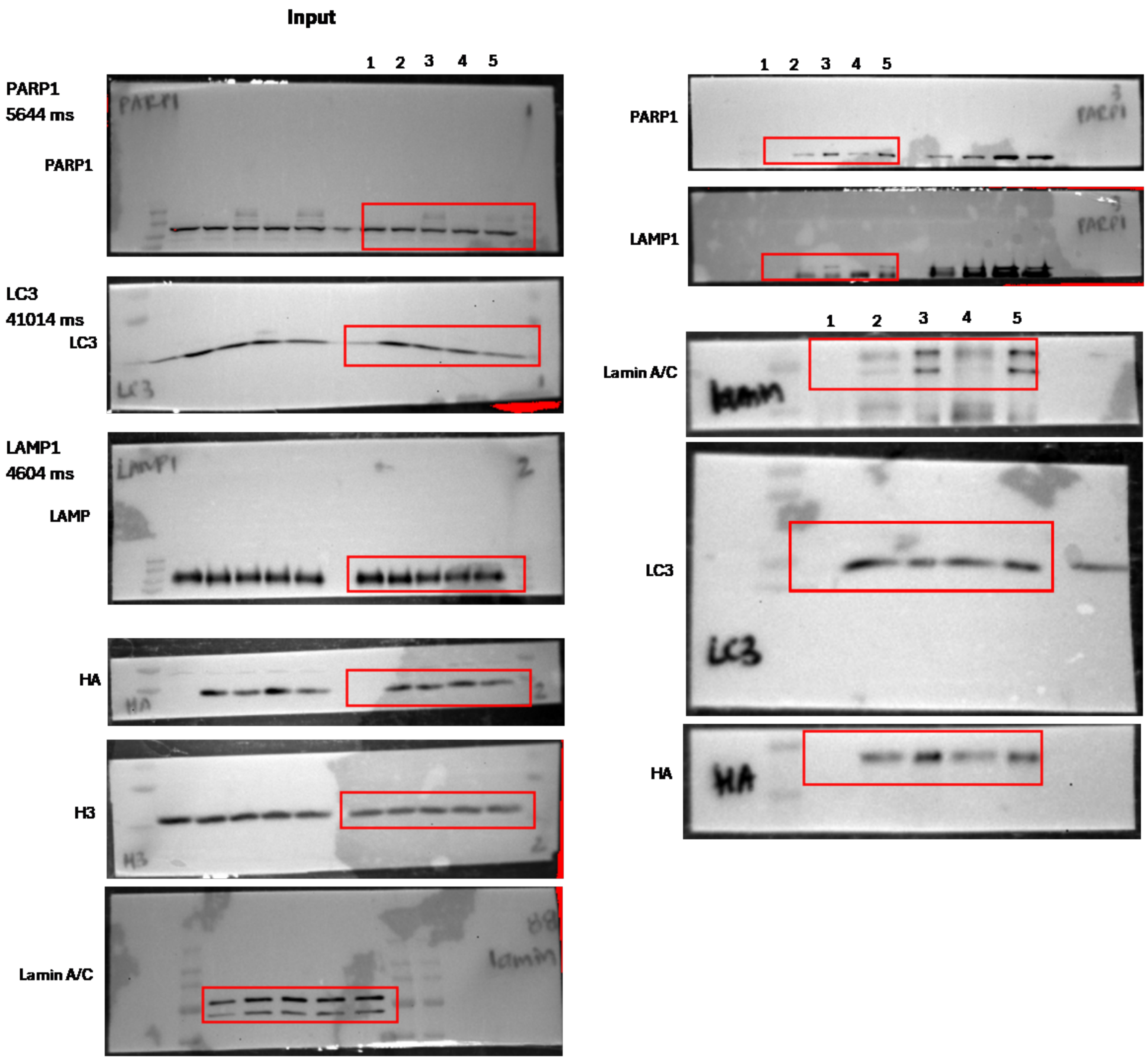

Extended Data Figure 6D-E
LysoIP with ATR inhibitor in HeLa WT and TMEM192-3HA cells

Lane	Sample
1	WT utd
2	TMEM Baf
3	TMEM BAF + Tala + MMS
4	TMEM BAF + ATRi
5	TMEM Baf + Tala + MMS + ATRi

Exp	Baf	Baf + TM	Baf + ATRi	Baf + ATRi + TM	
#1	0.149	1	0.161	0.075	
#2	0.546	1	0.465	0.917	
#3	0.268	1	0.071	0.211	

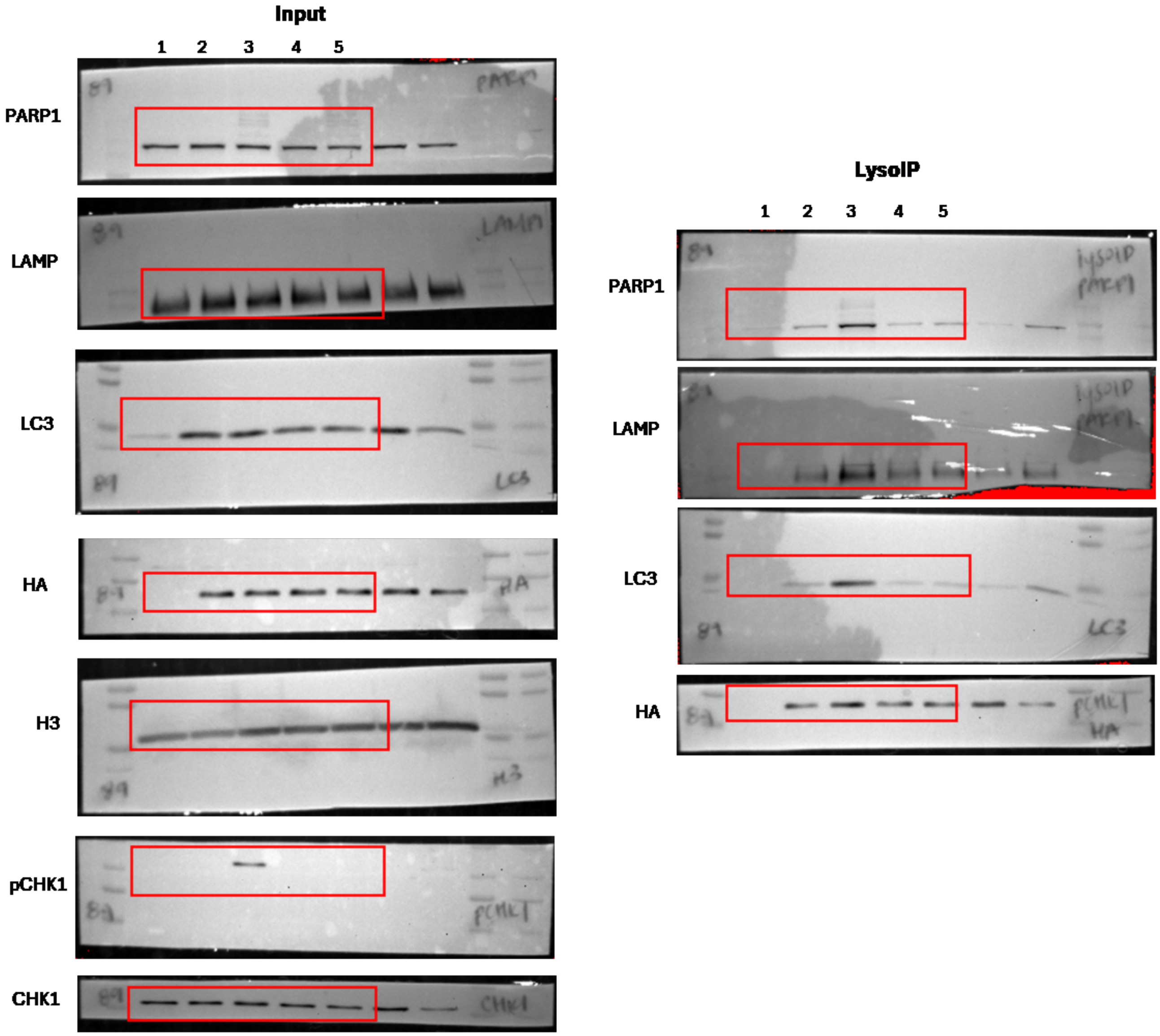

Supplement: Supplementary file 24 — Unprocessed western blots. [file 41556_2026_1961_MOESM24_ESM.pdf]
