# Supplementary material for: Nucleophagy removes cytotoxic trapped PARP1
Source: Nat Cell Biol. 2026 Jun 2;28(6):1219–34. doi: 10.1038/s41556-026-01961-5 (PMC13278974; doi:10.1038/s41556-026-01961-5)

Source Data for Extended Data Figure 7

Extended Data Figure 7B
Right is with membrane overlay to show ladder. Red box shows area in figure
1: HeLa WT utd
2: HeLa WT Tala
3: HeLa TEX264-/- utd
4: HeLa TEX264-/- Tala

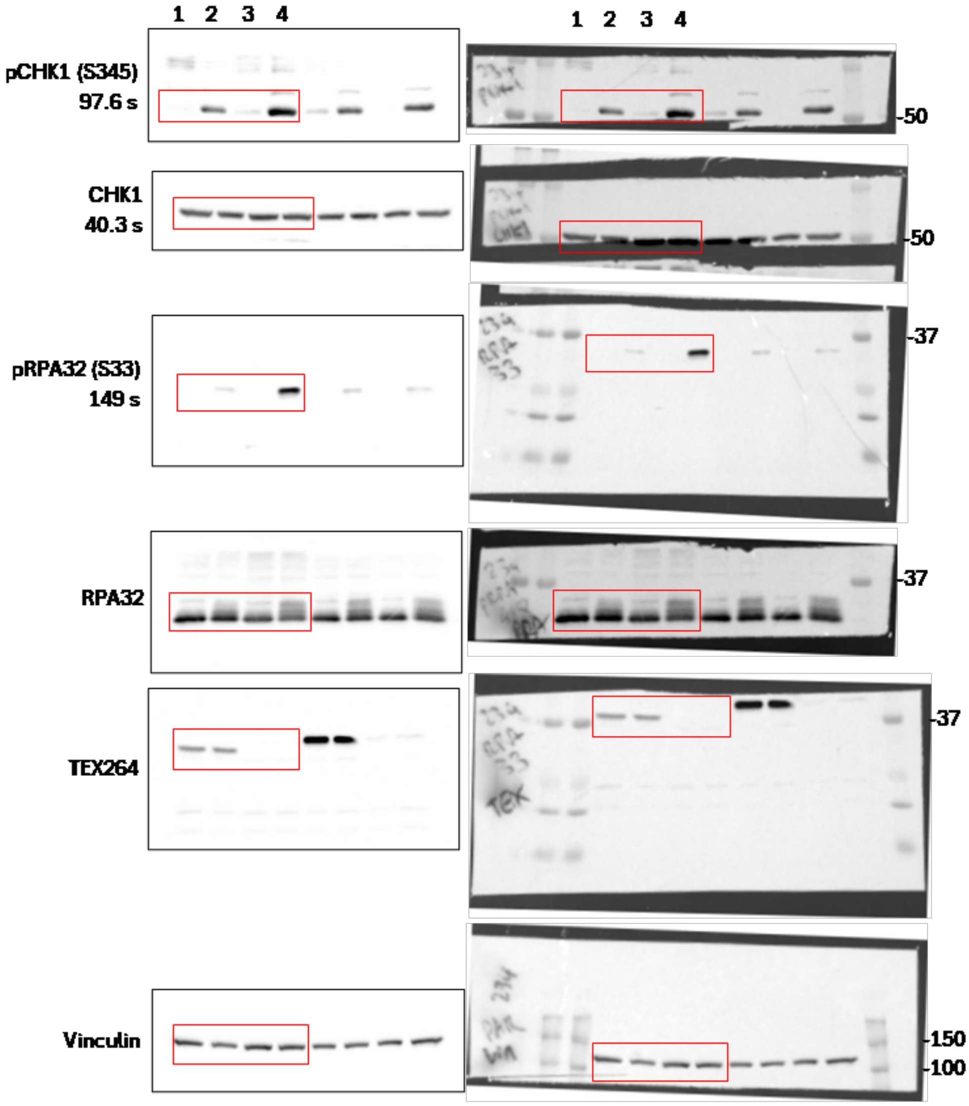

Supplement: Supplementary file 26 — Unprocessed western blots. [file 41556_2026_1961_MOESM26_ESM.pdf]
