# Supplementary material for: Nucleophagy removes cytotoxic trapped PARP1
Source: Nat Cell Biol. 2026 Jun 2;28(6):1219–34. doi: 10.1038/s41556-026-01961-5 (PMC13278974; doi:10.1038/s41556-026-01961-5)

Source Data for Extended Data Figure 8

Extended Data Figure 8F

Right is with membrane overlay to show ladder. Red box shows area in figure

1: HeLa shATG7 utd

2: HeLa shATG7 Tala

3: HeLa shATG7 Dox

4: HeLa shATG7 Dox Tala

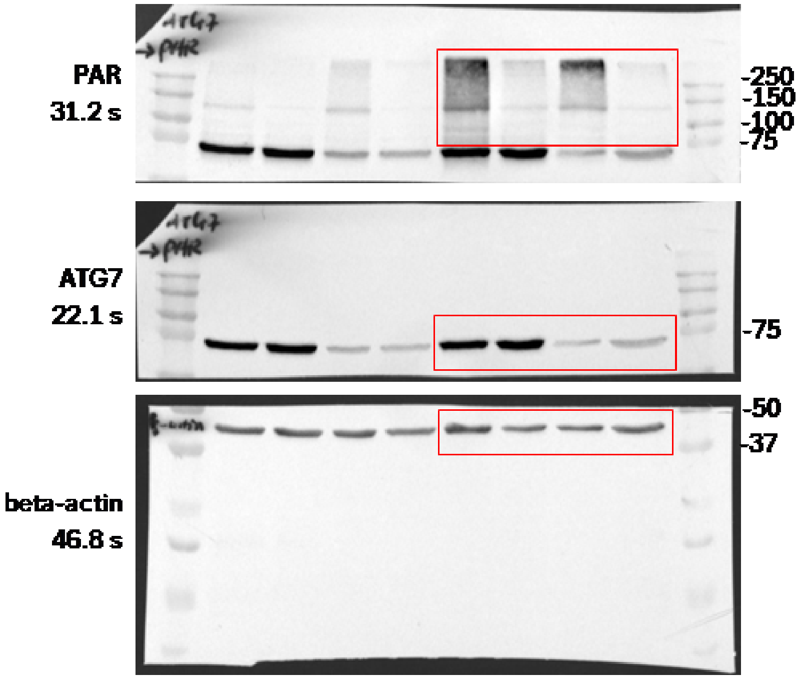

Supplement: Supplementary file 28 — Unprocessed western blots. [file 41556_2026_1961_MOESM28_ESM.pdf]
