# Supplementary material for: Nucleophagy removes cytotoxic trapped PARP1
Source: Nat Cell Biol. 2026 Jun 2;28(6):1219–34. doi: 10.1038/s41556-026-01961-5 (PMC13278974; doi:10.1038/s41556-026-01961-5)

Source Data for Extended Data Figure 9

Extended Data Figure 9A
Right is with membrane overlay to show ladder. Red box shows area in figure

1 HeLa WT siCtrl
2 HeLa WT siUFD1
3 HeLa TEX264-KO siCtrl
4 HeLa TEX264-KO siUFD1

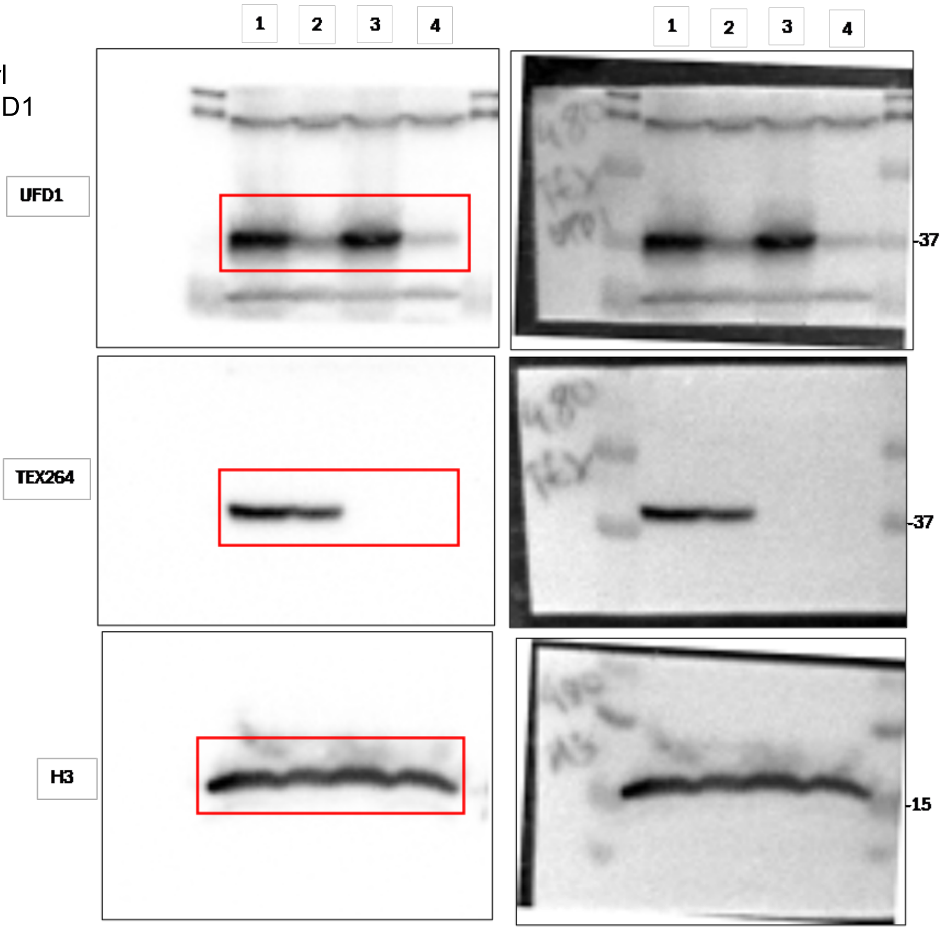

Extended Data Figure 9B
Right is with membrane overlay to show ladder. Red box shows area in figure
1: RPE BRCA1-/- resistant siCtrl
2: RPE BRCA1-/- resistant siTEX264
3: RPE BRCA1-/- resistant siATG7

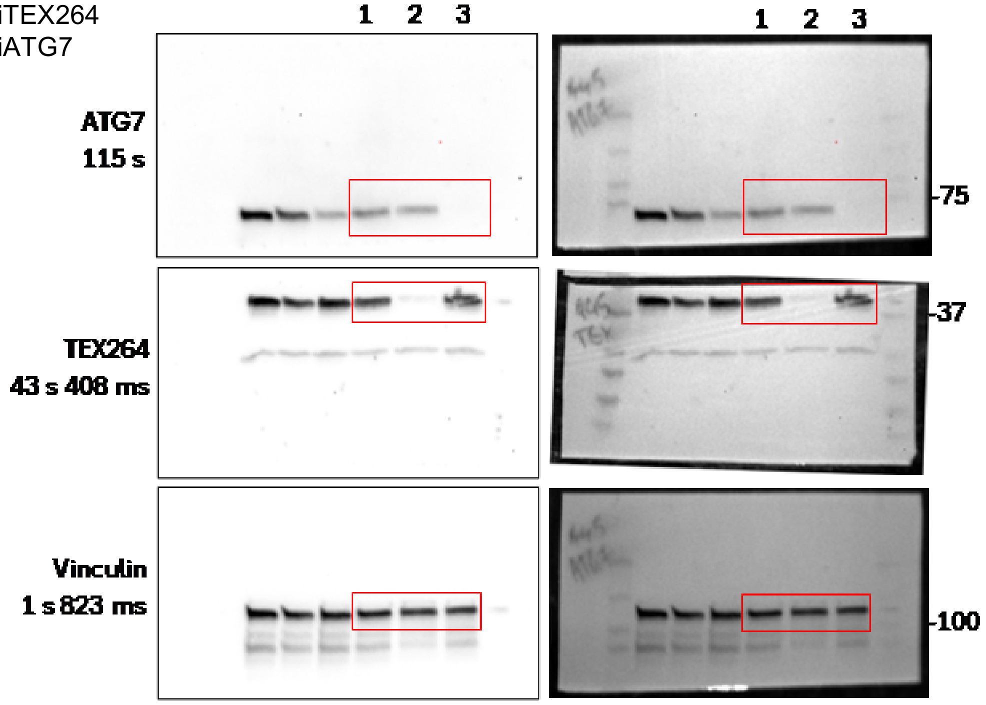

Supplement: Supplementary file 30 — Unprocessed western blots. [file 41556_2026_1961_MOESM30_ESM.pdf]
