# Supplementary material for: Nucleophagy removes cytotoxic trapped PARP1
Source: Nat Cell Biol. 2026 Jun 2;28(6):1219–34. doi: 10.1038/s41556-026-01961-5 (PMC13278974; doi:10.1038/s41556-026-01961-5)
Supplement: Supplementary file 2 — Peer Review File [file 41556_2026_1961_MOESM2_ESM.pdf]

nature portfolio

Peer Review File

Nucleophagy removes cytotoxic trapped PARP1

Corresponding Author: Professor Kristijan Ramadan

Version 0:

Decision Letter:

*Please delete the link to your author homepage if you wish to forward this email to co-authors.

Dear Professor Ramadan,

Thank you for your patience and for submitting your manuscript, "Selective nucleophagy removes cytotoxic trapped PARP1", to Nature Cell Biology. I am sorry again for the delay in sharing our decision with you. It has now been seen by 3 referees, who are experts in nucleophagy (Referee #1); nucleophagy, selective autophagy (Referee #2); and DNA repair, PARP (Referee #3). As you will see from their comments (attached below), they found the work of potential interest but have raised substantial concerns, which in our view would need to be addressed with considerable revisions before we can consider publication in Nature Cell Biology.

Nature Cell Biology editors discuss the referee reports in detail within the editorial team, including the chief editor, to identify key referee points that should be addressed with priority, and requests that are overruled as being beyond the scope of the current study. To guide the scope of the revisions, I have listed these points below. Our standard revision period is six months, and we are committed to providing a fair and constructive peer-review process, so please feel free to contact me if you would like to discuss any of the referee comments further or anticipate any issues or delays addressing the reviews.

I should stress that the referees' concerns point to a premature dataset and their concerns regarding the mechanism and model would need to be addressed thoroughly experimentally, and reconsideration of the study for this journal and re-engagement of the referees will depend on the strength of these revisions. In our view, it would be essential to dedicate efforts to address the following comments:

1- The reviewers emphasized the need to delve into the autophagic removal of trapped PARP1 much more directly and to provide more convincing evidence. We agree this level of evidence will be needed to convince readers. More direct evidence implicating nucleophagy specifically is required, including resolving the localization of TEX264:

Rev#1 paragraphs starting with “In addition to ATG7..”; “Did the authors check..”; “Lines 116-119..”; “Lines 158-161..”; “Line 251..”; “Lines 252-255..”; “Immunoblotting detection ..”; “Line 262..”; “Figure 1E..”; “Fig. 2H and ..”; “Fig. 2I..”; “Figure 4G ..”
Rev#2 points #1, #3, #7, #9, #10
Rev#3 main comments and points #1-2

2-A related concern is the need to study the interaction of TEX264 with PARP1, and to determine what other factors may be involved, such as DNA or RNA4, UFD1, in a convincing manner:

Rev#1 paragraphs starting with “My major concern is that…”; “In addition, the authors show..”
Rev#2 points #2, #8
Rev#3 points #3, #4

3- All other referee concerns pertaining to strengthening existing data, providing controls, methodological details, clarifications and textual changes, should be addressed.

4- Finally, please pay close attention to our guidelines on statistical and methodological reporting (listed below) as failure to do so may delay the reconsideration of the revised manuscript. In particular, please provide:

- a Supplementary Figure including unprocessed images of all gels/blots in the form of a multi-page pdf file. Please ensure that blots/gels are labeled and the sections presented in the figures are clearly indicated.

- a Supplementary Table including all numerical source data in Excel format, with data for different figures provided as different sheets within a single Excel file. The file should include source data giving rise to graphical representations and

statistical descriptions in the paper and for all instances where the figures present representative experiments of multiple independent repeats, the source data of all repeats should be provided.

We would be happy to consider a revised manuscript that would satisfactorily address these points, unless a similar paper is published elsewhere, or is accepted for publication in Nature Cell Biology in the meantime.

In contrast, although we agree with Reviewer #1 that this is a valuable suggestion, we do not think that mechanistic insights addressing the regulation of the pathway (as suggested in the comment "In view of the proposed importance of the TEX264-p97 axis in trapped PARP1 repair, the regulation of this mechanism should be investigated in detail.") would be needed at NCB, if all other points about the mechanism and model are addressed rigorously. Thus, addressing it experimentally will not be necessary for reconsideration of the manuscript at this journal.

When revising the manuscript please:

- ensure that it conforms to our format instructions and publication policies (see below and https://www.nature.com/nature/for-authors).

- provide a point-by-point rebuttal to the full referee reports verbatim, as provided at the end of this letter.

- provide the completed Reporting Summary (found here https://www.nature.com/documents/nr-reporting-summary.pdf). This is essential for reconsideration of the manuscript will be available to editors and referees in the event of peer review. For more information see http://www.nature.com/authors/policies/availability.html or contact me.

When submitting the revised version of your manuscript, please pay close attention to our href="https://www.nature.com/nature-portfolio/editorial-policies/image-integrity">Digital Image Integrity Guidelines. and to the following points below:

-- that unprocessed scans are clearly labelled and match the gels and western blots presented in figures.
-- that control panels for gels and western blots are appropriately described as loading on sample processing controls
-- all images in the paper are checked for duplication of panels and for splicing of gel lanes.

Finally, please ensure that you retain unprocessed data and metadata files after publication, ideally archiving data in perpetuity, as these may be requested during the peer review and production process or after publication if any issues arise.

Nature Cell Biology is committed to improving transparency in authorship. As part of our efforts in this direction, we are now requesting that all authors identified as ‘corresponding author’ on published papers create and link their Open Researcher and Contributor Identifier (ORCID) with their account on the Manuscript Tracking System (MTS), prior to acceptance. ORCID helps the scientific community achieve unambiguous attribution of all scholarly contributions. You can create and link your ORCID from the home page of the MTS by clicking on ‘Modify my Springer Nature account’. For more information please visit please visit www.springernature.com/orcid.

This journal strongly supports public availability of data. Please place the data used in your paper into a public data repository, or alternatively, present the data as Supplementary Information. If data can only be shared on request, please explain why in your Data Availability Statement, and also in the correspondence with your editor. Please note that for some data types, deposition in a public repository is mandatory - more information on our data deposition policies and available repositories appears below.

Please submit the revised manuscript files and the point-by-point rebuttal to the referee comments using this link:

Link Redacted

*This url links to your confidential home page and associated information about manuscripts you may have submitted or be reviewing for us. If you wish to forward this email to co-authors, please delete the link to your homepage.

We hope that you will find our referees' comments and editorial guidance helpful. Please do not hesitate to contact me if there is anything you would like to discuss. Thank you again for considering NCB for your work.

Best wishes,

Melina

Melina Casadio, PhD
Senior Editor, Nature Cell Biology

Consulting Editor, Nature Structural & Molecular Biology
ORCID ID: https://orcid.org/0000-0003-2389-2243

Reviewers' Comments:

Reviewer #1 (Remarks to the Author):

In this manuscript, Hoslett and colleagues aimed to elucidate the mechanisms mediating the clearance of trapped Poly (ADP-ribose) polymerase (PARP1) from chromatin, thus conferring resistance to PARPi with clinical implications for cancer treatment. Using RNA seq on triple-negative breast cancer CAL51 cells after 24 hours treatment with the strong trapping PARPi talazoparib, they identified genes that are differentially expressed upon treatment with PARP inhibitors (PARPi). Genes related to DNA damage repair, replication stress, apoptosis, G1/S cell cycle checkpoint and mitotic checkpoint were found to be upregulated in treated cells compared to controls. In addition, genes of the autophagy core machinery as well as autophagy modulators such as TP53INP1, DRAM1, PMAIP1, MYO6, EI24 and negative regulators of mTOR signaling, which stimulates autophagy, SESN1 and SESN2, were significantly upregulated in treated cells compared to controls. Unlike autophagy, genes involved in proteosomal-mediated degradation were not significantly induced by PARPi treatment. Complementary to the above-mentioned RNA seq analysis, two whole genome CRISPR screens for loss of autophagy factors affecting sensitivity to PARPi also revealed several autophagy-related genes that were significantly represented among the candidate PARPi sensitivity genes, suggesting that loss of some genes causes sensitivity to PARPi. Mass spectrometry data from authors' previous work were in agreement with the aforementioned results implicating autophagy in trapped PARP removal. Further analysis showed that autophagy stimulation by Torin-1 causes resistance to talazoparid, whereas treatment with bafilomycin increased sensitivity. These results were further validated by genetically interfering with the autophagy process through genetic inhibition of ATG7 and syntaxin-17. Furthermore, the authors linked the potential cytoprotective effect of autophagy upon PARPi treatment to the effect on PARP trapping since autophagy did not show the same effect when veliparid is used. The latter inhibits PPAR catalytic activity without causing trapping. Moreover, PARP1 was observed to accumulate in lysosomes isolated from HeLa and CAL51 cells expressing the lysosomal transmembrane protein TMEM192-3HA under trapping conditions. In cells transfected with a PARP1 construct tagged with mCherry and GFP, PARP was localized mostly in the lysosome under trapping conditions. This localization was abrogated upon depletion of either ATG7 or syntaxin-17, further supporting a role for autophagy in the processing of trapped PARP. Recruitment of PARP in the lysosome under trapping conditions appears to be mediated by p97. However, regulation of trapped PARP1 processing by autophagy was shown to be independent of RNF4-mediated or UFD-mediated ubiquitination of PARP1, indicating that the p97- RNF4-UFD pathway acts separately from autophagy for the processing of trapped PARP1. Since TEX264 is a co-factor of p97, the authors explored whether it is involved in the modulation of trapped PARP1 levels. Indeed, it was shown that depletion or knockout of TEX264 in three different human lines caused an accumulation of trapped PARP1, suggesting that this receptor acts as a modulator of trapped PARP1 levels. Previously identified TEX264 interactomes confirmed that PARP1 is among its interactors. In addition, it was shown that a mutation in TEX264, which has the characteristic of the selective autophagy receptor LIR domain, prevents PARP1 accumulation in the lysosome under trapping. This suggests that TEX264-deficient cells experience altered DDR under PARPi treatment. Similar to talazoparib, resistance to the milder PARPi olaparib, was abolished in RPE TP53-/-hTERT BRCA1-/- cells, upon depletion of TEX264 and ATG7.

Comments
My major concern is that the authors claim a prominent role for nucleophagy in mediating the removal of cytotoxic trapped PARP1, but do not provide sufficiently robust data to support this view. In fact, they provide evidence implicating the selective autophagy receptor TEX264 and the ATPase p97 in the processing of trapped PARP1 from chromatin and its delivery to the lysosome. Importantly however, the physical interaction of TEX264 with PARP1 has not been satisfactorily studied. As this is a critical part of the proposed mechanism for trapped PARP1 removal, emphasis should be placed on the identification of specific domains/motifs involved. In addition, other molecular players known to be involved in nucleophagy have not been studied in order to validate the main claim of the manuscript.

In addition, the authors show that removal of trapped PARP1 by the proposed mechanism depends on SUMOylation and ubiquitination, but is independent of the SUMO-dependent E3-ubiquitin ligase RNF4, which was previously found to regulate trapped PARP1 degradation by the proteasome. They should elaborate on this claim to provide experimental evidence for candidate E3 ligase(s) involved.

In view of the proposed importance of the TEX264-p97 axis in trapped PARP1 repair, the regulation of this mechanism should be investigated in detail.

The finding that PARP1 is processed by autophagy is not new nor is the notion that inhibiting autophagy results in increased sensitivity of cells to PARPi treatment (for example, Bellare et al.,2021, Br J Cancer. 124(7): 1260–1274; Cahuzac et al., 2022, Commun Biol 5, 251; Elshazly et al., 2022). More importantly, the function of TEX264 as a nucleophagy receptor, in addition to its role in ERphagy, has been previously reported (Fielden et al., 2022, Autophagy 18, 40–49).

In addition to ATG7, the authors should also test the effects of depleting other essential autophagy genes such as ATG16L1 etc. that are enriched under PARP1-trapping conditions.

Did the authors check whether the nuclear localization of p62/SQSTM1 plays a role in their experimental setting as was

previously shown for olaparib?

The arrangement of the panels in Figure 2 is somewhat confusing (e.g., Fig. 2D top, Fig 2C bottom and Fig 2F bottom, 2G top).

Lines 116-119: Selective autophagy may or may not involve selective autophagy receptors/adaptor proteins. However, selective autophagy may also occur through the recognition of LC3-interacting region (LIR) of autophagic cargo directly by LC3, without the implication of selective autophagy receptors. Please correct.

Lines 158-161: It has been shown that TP53INP1 can promote autophagy-dependent cell death and that DRAM1, PMAIP1, MYO6 and EI24 can act as positive regulators of autophagy. While all listed genes TP53INP1, DRAM1, PMAIP1, MYO6, EI24 regulate autophagy, particularly upon genotoxic stress, yet these proteins are not traditionally considered core autophagy proteins, i.e. essential components that directly participate in the formation of function of the autophagic machinery. This comment regards also some genes labelled as core autophagy in Figure 1C.

Line 251: The statement "PARP1 physically interacted with early-stage autophagy core machinery" needs to be toned down. The proximity labelling experiments indicate proximity of two proteins but whether they physically interact or not is not shown.

Lines 252-255: Isolation of intact lysosomes and analysis of their contents (especially under Bafilomycin A1 treatment) cannot be an assay itself to explore whether trapped PARP1 could be cleared by autophagy. It has to be accompanied by and compared to the PARP1 abundance in the flow-through fraction. Bafilomycin A1 treatment should trap PARP1 in the autophagic compartments that cannot fuse with lysosomes and not necessarily into lysosomes as it is shown in Fig. 2B, C. In addition, control conditions without Bafilomycin A1 are necessary to show the relative abundance and organellar distribution of PARP1 under physiological conditions.

Immunoblotting detection of intact (full length) mCherry-PARP1-GFP fusion protein under the experimental conditions is needed. When tandem constructs are used in this context it is preferable to utilize live cell imaging rather than fixed cell imaging (Fig 2F, G), to avoid the risk of GFP fluorescence restoration upon increase of the lysosomal pH during fixation (10.1080/15548627.2020.1797280).

Line 262: While the effort for assessing lysosomal localization of PARP1KS mutant (7 biological replicates) is appreciated, the really low levels of PARP1KS (particularly for IP) make it difficult to confidently use it as a control for concluding that trapping is required for its degradation by autophagy. Instead, the authors should use veliparib on WT PARP1.

Line 295: The author showed inhibition of p97 by CB-5083 significantly reduced the accumulation of PARP1 in the lysosome. Is this reduction accompanied by an increase on PARP1 levels on chromatin (pre-extraction protocol)? The authors previously showed (ref 39) that expression of a p97 mutant increases PARP1 levels on chromatin but does the same happen with the inhibitor treatment?

Figure 1E: What was the reason for selecting a different assay (resazurin) for assessing sensitivity genetically compared to the chemical treatments (Figs 1D & G)? If this was necessary for technical issues (please indicate why, perhaps in methods as it could be useful to others) related to depletion, could you also repeat in this set up the boosting of autophagy treatment as a control? Also, could the authors explain why a different range of talazoparid concentration was used compared to 1D & G?

Figure 1H: Given that experiments referring to Fig.1H precede the negative experiments using veliparid, I think it is essential for the immunofluorescence experiments to also include veliparid as a negative control, to validate the findings in Fig.1G.

Fig. 2H and Movie 1: It seems there are two congregation events between mCherry-PARP1-GFP-positive structures and Lysoview 680-stained lysosomes in the movie. Do these structures show GFP quenching after the presumptive lysosomal engagement? Could they represent some random proximity events between the two organelles? It is not clear how authors concluded that the above “further confirms that trapped PARP1 localizes to the lysosome” (lines 277-278).

Fig. 2I: It seems that the residual ATG7 during siATG7 is enough for to permit (ATG12 conjugation to ATG5 and) LC3 lipidation (last two lines in LC3 blot). Do these bands represent LC3II as the molecular weight and their accumulation upon Bafilomycin A1 treatment suggest? Since LC3 is detected to some extend in the lysosome fractions, it suggests that autophagosome-lysosome fusion takes place upon siATG7. Judging from the legend of Fig. 2D, PARP1 levels normalization is performed by dividing with HA signal (please specify in each legend or the methods section how each normalization takes place). Normalization towards LC3 signal is needed to show the correlation of PARP1 engagement to the lysosome with LC3 delivery to the lysosome.

Figure 4G shows almost no PARP1 in basal conditions in triple negative breast cancer cell line CAL51. Furthermore, PARP1 is almost absent in the cytoplasm in most images (in line with its well-known localization in the nucleoplasm, nucleoli and micronucleus). Nevertheless, it is surprising that there is almost no PARP1 outside the nucleus in any experimental condition, not even in lysosomes. How do the authors explain its absent in basal conditions? Are there any proposed mechanisms for its translation upon different signals? And how do they explain their absence in lysosomes?

Minor points

Authors may want to keep a constant representation of y-axis numbering and cut points in Fig. 1A, C and Fig. S4A, B. Values sometimes appear/align in some cut points.

Lines 134- 135 and 501-506: How do TEX264 and p97 mediate the delivery of PARP1 to the lysosomes? How do these complexes pass through the nuclear membrane?

The authors need to explain in more detail in the figure legend or the figures how they normalize their data. For example, in Figure 1I and 2D it is not clear what the authors normalize against (Fig 1I) or normalized lysosomal PARP1 levels. Also, it is advisable for the authors to re-arrange the panels in the respective figures in order to appear in the logical order (from up to down and from left to right).

Fig. 6E is duplicated.

The panel order in some figures (1, 2 and 5) is confusing.

Nucleophagy is a selective type of autophagy. The term “selective nucleophagy” is not appropriate in the context used by the authors, as it implies that specific nuclear components are selected and targeted, which is not shown here.

Reviewer #2 (Remarks to the Author):

PARP1 (Poly ADP-ribose polymerase) is an enzyme involved in DNA repair. When treated with some of its inhibitors (PARPi), PARP1 becomes trapped on DNA, and this trapped PARP1 causes cytotoxicity. Cells defective in homologous recombination are sensitive to PARPi, and therefore PARPi are particularly effective in treating cancers with BRCA1 or BRCA2 gene mutations. In this study, Hoslett et al. discovered that trapped PARP1 is transported to lysosomes via autophagy in a manner dependent on the AAA-ATPase p97 and TEX264, which was previously reported to serve as a receptor for autophagy of the ER. Importantly, impairing autophagic degradation of trapped PARP1 increased cell sensitivity to PARPi, suggesting that selective autophagy of trapped PARP1 could be a druggable target especially for cancer cells tha 4 t have acquired resistance to PARPi. Thus, this study provides key findings for future pharmaceutical applications, but the authors should address the following issues to strengthen the model on how trapped PARP is transported to lysosomes via autophagy.

Major comments:
1. While TEX264 was reported to be an ER-localized transmembrane protein involved in autophagic degradation of the ER, its nuclear membrane localization remains unclear. In the authors' model, TEX264 binds PARP1 in the nucleus and then interacts with autophagy proteins in the cytoplasm to its sequestration within autophagosomes. To validate this model, the authors should show that TEX264 resides in the inner nuclear membrane in addition to the ER. It is also important to show how this protein complexed with PARP1 is exposed to the cytoplasm. Does lysosomal transport of PARP1 depend on nuclear export through the nuclear pore?

2. Is DNA transported to lysosomes along with PARP1?

3. To conclude that trapped PARP1 is transported to lysosomes via macroautophagy, the colocalization of PARP1 and LC3 in the cytoplasm and its increase in STX17-knockdown cells should be shown. In addition, the authors should examine lysosomal transport of PARP1 in cells depleted for not only ATG7 but also other core ATGs such as FIP200, because recent studies reported degradation of different cellular components including the outer nuclear membrane via microautophagy, which specifically depends on the ATG8 conjugation system among core ATGs.

4. Fig. 1D: It is required to show that the effect of Torin is cancelled by ATG depletion.

5. Fig. 2B: Controls for "Baf -, Tala +" and "ATG knockdown" should be added.

6. Fig. 2F: The frequency of cells with cytoplasmic PAPR1 signals should be quantified. It is also necessary to check whether cytoplasmic PAPR1 signals disappear by ATG knockdown.

7. Fig. 2H and Movie 1: These data do not clearly show that cytosolic structures positive for PARP1 finally fuse with lysosomes.

8. Fig. 5A: How PARP1, p97, and TEX264 interact with each other should be clarified by depleting one of them and using mutants defective in the interactions. Does trapped PARP1 indeed interact with TEX264 directly?

9. Fig. 5B: How do the authors explain why lots of signals appear within the nucleus in the proximity ligation assay, even though TEX264 should be anchored to the inner nuclear membrane (if the authors' model is correct), and why these signals are much more than cytoplasmic PARP1 signals shown in Fig. 2F.

10. Fig. 5D: Examining the co-isolation of TEX264 and p97 with lysosomes will provide further molecular insights into trapped PARP1 transport to lysosomes.

Minor comments:

1. Lines 158-159, "core autophagy machinery, namely TP53INP1, DRAM1, PMAIP1, MYO6, EI24": This description should be corrected because core autophagy machinery or core autophagy-related proteins are used to represent proteins required for autophagosome formation such as FIP200, ATG7, and ATG8.

2. Lines 188-190, "Loss of regulators of autophagy USP7, USP8, CUL3, MTOR and VCP are also implicated in cell sensitivity to PARPi (Fig 1B).": References for the relationship with autophagy should be added to each factor. In addition, MTOR is inappropriate in this context because it is a negative regulator of autophagy. The authors should check other factors in this regard as well.

3. While sequestrating PARP1 within autophagosomes seems to be sufficient to decrease the cytotoxicity of trapped PARP1, not only ATG7 depletion but bafilomycin treatment and STX17 depletion also increased cell sensitivity to talazoparib. How do the authors interpret these results?

4. Lines 460-462, "TEX264 was previously described to mediate selective autophagy of the ER (reticulophagy) through association with the damaged ER membrane via its N-terminal leucine-rich region.": The authors may want to check whether these papers describe that TEX264 mediates degradation of "damaged" ER membranes.

Reviewer #3 (Remarks to the Author):

In their manuscript, Hostlett et al. describe a potentially novel mechanism that mediates the clearance of trapped PARP1 and contributes to PARPi resistance. Through data mining, they found that blocking or loss of autophagy synergizes with PARPi, specifically PARP trapping. Correspondingly, blocking lysosome acidification using Bafilomycin A1 or knocking down autophagy factors sensitizes cells to the PARPi Talazoparib, whereas the mTOR inhibitor Torin confers resistance.

Using various cell biology assays, the authors showed that trapped PARP1 accumulates in lysosomes in an autophagy-dependent manner, which also requires p97. However, in contrast to their previous findings, where E3 ligase RNF4 and UFD1 mediated the recruitment of p97 to trapped PARP1, they found that modulating RNF4 and UFD1 does not impact lysosomal accumulation of PARP1. This prompted them to seek an alternative mechanism linking trapped PARP1 to p97 and the lysosome.

Drawing an analogy between Top1cc and trapped PARP1, the authors tested whether TEX264 might mediate the recruitment of p97 to trapped PARP1. Based on their results, they proposed that TEX264 directly interacts with trapped PARP1 and, together with its partner protein p97, transports the trapped PARP1 to the autophagosomal resident protein LC3 and subsequently to the autophagosome. Furthermore, they found that interfering with the autophagy pathway can partially restore PARPi sensitivity in resistant clones.

Overall, the manuscript presents a wealth of data and offers a potentially intriguing twist on the mechanism of trapped PARP1 degradation. However, much of the evidence appears more correlative than indicative of specific interactions. Given that PARPi sensitivity is closely tied to cell proliferation, identifying the specific interaction surfaces and generating site-dependent mutants will be necessary to establish a clear cause-and-effect relationship and uncover the molecular link between trapped PARP1 and autophagy. The paper would benefit from providing more mechanistic insights/order into the processing of trapped PARP1 on DNA, relevant signaling pathways, and clear evidence of a direct interaction between PARP1 and TEX264 prior to lysosomal transfer.

Conceptual concerns:
1. Pleiotropic Effects of Reagents: Many of the reagents used in the study, including Bafilomycin A1, Torin, siATG, p97, and TEX264, have pleiotropic effects on cell proliferation and the clearance of other replication blockades, such as Top1cc, which the authors mention. Depletion of ATG7, a core autophagy factor, likely blocks lysosomal import of most proteins targeted for lysosomal degradation. As a result, it is not surprising that ATG7 depletion would also block PARP1 clearance, making the findings more correlative than specific.
2. Tankyrase Inhibition and PARPi Specificity: Talazoparib, which was used in most experiments, also inhibits Tankyrase, a protein that interacts with ATG9A, an autophagy factor. In normal cells, Tankyrase levels are kept low through PARylation-dependent proteasomal degradation. Inhibiting Tankyrase activity—either through specific inhibitors or Talazoparib—causes Tankyrase accumulation. To exclude the possibility that the observed phenotypes after Talazoparib treatment are due to Tankyrase inhibition rather than specific inhibition of PARP1, the authors should repeat key experiments with other PARP inhibitors, such as Niraparib (a potent PARP1 trapper) or the PARP1-specific inhibitor Saruparib (AZD5305).
3. Selective Nucleophagy and TEX264-PARP1 Interaction: The proposed model for removing trapped PARP1 via selective nucleophagy requires more details. Specifically, the model would be strengthened by identifying the interaction surface between TEX264 and trapped PARP1. Additionally, it is important to clarify whether the TEX264-PARP1 interaction is DNA-mediated (i.e., whether it is sensitive to DNase or Benzonase). In this context, Figure 5 suggests that the TEX264-PARP1 interaction can occur in the absence of PARPi, raising questions about whether TEX264 specifically removes trapped PARP1 for clearance.
4. RNF4, UFD1, and TEX264 Pathway: The authors, in collaboration with Dr. Chris Lord, previously showed that RNF4 and UFD1 sense trapped PARP1, deposit Ub and SUMO, and recruit p97 to remove trapped PARP1. In this manuscript, they report that lysosomal enrichment of PARP1 depends on p97 but not on RNF4 or UFD1. They further state (lines 333-336):

“Indeed, either depletion or knockout of TEX264 in three different human cell lines, including HeLa cervical carcinoma cell line and two triple-negative breast cancer cell lines, CAL51 and MDA-MB231, caused a considerable accumulation of trapped PARP1, to a similar extent as previously identified co-factor UFD1.”
It would be important to delete both UFD1 and TEX264, or RNF4 and TEX264, to determine whether this autophagy pathway is epistatic or additive to the previously identified Ub/SUMO-dependent p97 recruitment pathway. Furthermore, the authors should clarify whether TEX264 acts upstream of p97. If p97 is responsible for removing trapped PARP1 from chromatin for autophagy, why does blocking lysosome function after PARP1 is no longer trapped at chromatin (i.e., after p97 removal) impede DNA replication and repair?
Additional Technical comments
1) PARP1del.p.119K120S Mutant: The PARP1del.p.119K120S mutant was introduced as a non-trapping variant of PARP1. It would be important to measure its trapping ability compared to wild-type (WT) PARP1 in the presence of different PARP inhibitors.
2) Clarification of "Mildly Depleted Genes": At line 190, the authors refer to “mildly depleted genes” in the CRISPR screen of Figure 1B. Could they clarify the criteria used to define "mildly depleted genes"?
3) Potential Pleiotropic Effects of Torin1: In Fig. 1D, Torin1 might exhibit pleiotropic effects, particularly in blocking cell proliferation. It is well-documented that PARPi sensitivity depends on cell proliferation and DNA replication. Is there an alternative molecule that can activate autophagy without affecting cell growth?
4) Co-localization of PARP1 with Lysosome Tracker: In Fig. 2H, it appears that only a small fraction of PARP1 co-localizes with the lysosome tracker. Could the authors explain how frequently nucleophagy-induced PARP1 removal occurs and whether this effect is widespread?
5) PARP1 Expression in Input Group: In Fig. 2K, the expression level of PARP1 in the last two lanes of the input group appears slightly decreased. Have the authors investigated whether autophagy-mediated removal of PARP1 affects the total amount of PARP1 in cells as a result of degradation?
6) Survival Experiments with PARP1 Mutant: While Figure 4 is convincing, could the survival experiment be performed in cells expressing a PARP1 mutant that impairs trapping? This would help determine if the effect is entirely PARP1-dependent, as TEX264 and p97 have multiple targets. Additionally, have the authors investigated whether TEX264-/- cells proliferate at the same rate as WT?
7) Error in Fig. 6E: There appears to be an error in Fig. 6E, with overlapping duplicate diagrams.
8) Relationship of p97 and TEX264: To further confirm that p97 and TEX264 act in the same pathway, the authors could use a p97 inhibitor (p97i) in TEX264-/- cells and assess whether there is a cumulative effect.
9) Line 458-460 Clarification: The authors found that TEX264 is implicated in both TOP1cc and trapped PARP1, but this does not necessarily mean TEX264 is specialized in DNA repair pathways.
10) Microscopy Foci Quantification: For greater clarity, microscopy foci quantification could be presented as a violin plot, with the median exposed.

GUIDELINES FOR MANUSCRIPT SUBMISSION TO NATURE CELL BIOLOGY

READABILITY OF MANUSCRIPTS – Nature Cell Biology is read by cell biologists from diverse backgrounds, many of whom are not native English speakers. Authors should aim to communicate their findings clearly, explaining technical jargon that might be unfamiliar to non-specialists, and avoiding non-standard abbreviations. Titles and abstracts should concisely communicate the main findings of the study, and the background, rationale, results and conclusions should be clearly explained in the manuscript in a manner accessible to a broad cell biology audience. Nature Cell Biology uses British spelling.

MANUSCRIPT FORMAT – please follow the guidelines listed in our Guide to Authors regarding manuscript formats at Nature Cell Biology.

TITLE – should be no more than 100 characters including spaces, without punctuation and avoiding technical terms, abbreviations, and active verbs..

AUTHOR NAMES – should be given in full.

AUTHOR AFFILIATIONS – should be denoted with numerical superscripts (not symbols) preceding the names. Full addresses should be included, with US states in full and providing zip/post codes. The corresponding author is denoted by: "Correspondence should be addressed to [initials]."

ABSTRACT AND MAIN TEXT – please follow the guidelines that are specific to the format of your manuscript, as listed in our Guide to Authors (http://www.nature.com/ncb/pdf/ncb_gta.pdf) Briefly, Nature Cell Biology Articles, Resources and Technical Reports have 3500 words, including a 150 word abstract, and the main text is subdivided in Introduction, Results, and Discussion sections. Nature Cell Biology Letters have up to 2500 words, including a 180 word introductory paragraph

(abstract), and the text is not subdivided in sections.

ACKNOWLEDGEMENTS – should be kept brief. Professional titles and affiliations are unnecessary. Grant numbers can be listed.

AUTHOR CONTRIBUTIONS – must be included after the Acknowledgements, detailing the contributions of each author to the paper (e.g. experimental work, project planning, data analysis etc.). Each author should be listed by his/her initials.

FINANCIAL AND NON-FINANCIAL COMPETING INTERESTS – the authors must include one of three declarations: (1) that they have no financial and non-financial competing interests; (2) that they have financial and non-financial competing interests; or (3) that they decline to respond, after the Author Contributions section. This statement will be published with the article, and in cases where financial and non-financial competing interests are declared, these will be itemized in a web supplement to the article. For further details please see https://www.nature.com/licenceforms/nrg/competing-interests.pdf.

REFERENCES – are limited to a total of 70 for Articles, Resources, Technical Reports; and 40 for Letters. This includes references in the main text and Methods combined. References must be numbered sequentially as they appear in the main text, tables and figure legends and Methods and must follow the precise style of Nature Cell Biology references. References only cited in the Methods should be numbered consecutively following the last reference cited in the main text. References only associated with Supplementary Information (e.g. in supplementary legends) do not count toward the total reference limit and do not need to be cited in numerical continuity with references in the main text. Only published papers can be cited, and each publication cited should be included in the numbered reference list, which should include the manuscript titles. Footnotes are not permitted.

METHODS – Nature Cell Biology publishes methods online. The methods section should be provided as a separate Word document, which will be copyedited and appended to the manuscript PDF, and incorporated within the HTML format of the paper.

Methods should be written concisely, but should contain all elements necessary to allow interpretation and replication of the results. As a guideline, Methods sections typically do not exceed 3,000 words. The Methods should be divided into subsections listing reagents and techniques. When citing previous methods, accurate references should be provided and any alterations should be noted. Information must be provided about: antibody dilutions, company names, catalogue numbers and clone numbers for monoclonal antibodies; sequences of RNAi and cDNA probes/primers or company names and catalogue numbers if reagents are commercial; cell line names, sources and information on cell line identity and authentication. Animal studies and experiments involving human subjects must be reported in detail, identifying the committees approving the protocols. For studies involving human subjects/samples, a statement must be included confirming that informed consent was obtained. Statistical analyses and information on the reproducibility of experimental results should be provided in a section titled "Statistics and Reproducibility".

All Nature Cell Biology manuscripts submitted on or after March 21 2016 must include a Data availability statement as a separate section after Methods but before references, under the heading "Data Availability". . For Springer Nature policies on data availability see http://www.nature.com/authors/policies/availability.html; for more information on this particular policy see http://www.nature.com/authors/policies/data/data-availability-statements-data-citations.pdf. The Data availability statement should include:

• Accession codes for primary datasets (generated during the study under consideration and designated as "primary accessions") and secondary datasets (published datasets reanalysed during the study under consideration, designated as "referenced accessions"). For primary accessions data should be made public to coincide with publication of the manuscript. A list of data types for which submission to community-endorsed public repositories is mandated (including sequence, structure, microarray, deep sequencing data) can be found here http://www.nature.com/authors/policies/availability.html#data.

• Unique identifiers (accession codes, DOIs or other unique persistent identifier) and hyperlinks for datasets deposited in an approved repository, but for which data deposition is not mandated (see here for details http://www.nature.com/sdata/data-policies/repositories).

• At a minimum, please include a statement confirming that all relevant data are available from the authors, and/or are included with the manuscript (e.g. as source data or supplementary information), listing which data are included (e.g. by figure panels and data types) and mentioning any restrictions on availability.

• If a dataset has a Digital Object Identifier (DOI) as its unique identifier, we strongly encourage including this in the Reference list and citing the dataset in the Methods.

We recommend that you upload the step-by-step protocols used in this manuscript to protocols.io. More details can found at https://www.protocols.io/help/publish-articles.

DISPLAY ITEMS – main display items are limited to 6-8 main figures and/or main tables for Articles, Resources, Technical Reports; and 5 main figures and/or main tables for Letters. For Supplementary Information see below.

FIGURES – Colour figure publication costs $600 for the first, and $300 for each subsequent colour figure. All panels of a multi-panel figure must be logically connected and arranged as they would appear in the final version. Unnecessary figures and figure panels should be avoided (e.g. data presented in small tables could be stated briefly in the text instead).

All imaging data should be accompanied by scale bars, which should be defined in the legend.
Cropped images of gels/blots are acceptable, but need to be accompanied by size markers, and to retain visible background signal within the linear range (i.e. should not be saturated). The boundaries of panels with low background have to be demarked with black lines. Splicing of panels should only be considered if unavoidable, and must be clearly marked on the figure, and noted in the legend with a statement on whether the samples were obtained and processed simultaneously. Quantitative comparisons between samples on different gels/blots are discouraged; if this is unavoidable, it should only be performed for samples derived from the same experiment with gels/blots were processed in parallel, which needs to be stated in the legend.

Figures should be provided at approximately the size that they are to be printed at (single column is 86 mm, double column is 170 mm) and should not exceed an A4 page (8.5 x 11"). Reduction to the scale that will be used on the page is not necessary, but multi-panel figures should be sized so that the whole figure can be reduced by the same amount at the smallest size at which essential details in each panel are visible. In the interest of our colour-blind readers we ask that you avoid using red and green for contrast in figures. Replacing red with magenta and green with turquoise are two possible colour-safe alternatives. Lines with widths of less than 1 point should be avoided. Sans serif typefaces, such as Helvetica (preferred) or Arial should be used. All text that forms part of a figure should be rewritable and removable.

We accept files from the following graphics packages in either PC or Macintosh format:

- For line art, graphs, charts and schematics we prefer Adobe Illustrator (.AI), Encapsulated PostScript (.EPS) or Portable Document Format (.PDF). Files should be saved or exported as such directly from the application in which they were made, to allow us to restyle them according to our journal house style.

- We accept PowerPoint (.PPT) files if they are fully editable. However, please refrain from adding PowerPoint graphical effects to objects, as this results in them outputting poor quality raster art. Text used for PowerPoint figures should be Helvetica (preferred) or Arial.

- We do not recommend using Adobe Photoshop for designing figures, but we can accept Photoshop generated (.PSD or .TIFF) files only if each element included in the figure (text, labels, pictures, graphs, arrows and scale bars) are on separate layers. All text should be editable in 'type layers' and line-art such as graphs and other simple schematics should be preserved and embedded within 'vector smart objects' - not flattened raster/bitmap graphics.

- Some programs can generate Postscript by 'printing to file' (found in the Print dialogue). If using an application not listed above, save the file in PostScript format or email our Art Editor, Allen Beattie for advice (a.beattie@nature.com).

Regardless of format, all figures must be vector graphic compatible files, not supplied in a flattened raster/bitmap graphics format, but should be fully editable, allowing us to highlight/copy/paste all text and move individual parts of the figures (i.e. arrows, lines, x and y axes, graphs, tick marks, scale bars etc.). The only parts of the figure that should be in pixel raster/bitmap format are photographic images or 3D rendered graphics/complex technical illustrations.

All placed images (i.e. a photo incorporated into a figure) should be on a separate layer and independent from any superimposed scale bars or text. Individual photographic images must be a minimum of 300+ DPI (at actual size) or kept constant from the original picture acquisition and not decreased in resolution post image acquisition. All colour artwork should be RGB format.

FIGURE LEGENDS – must not exceed 350 words for each figure to allow fit on a single printed NCB page together with the figure. They must include a brief title for the whole figure, and short descriptions of each panel with definitions of the symbols used, but without detailing methodology.

TABLES – main tables should be provided as individual Word files, together with a brief title and legend. For supplementary tables see below.

SUPPLEMENTARY INFORMATION – Supplementary information is material directly relevant to the conclusion of a paper, but which cannot be included in the printed version in order to keep the manuscript concise and accessible to the general reader. Supplementary information is an integral part of a Nature Cell Biology publication and should be prepared and presented with as much care as the main display item, but it must not include non-essential data or text, which may be removed at the editor's discretion. All supplementary material is fully peer-reviewed and published online as part of the HTML version of the manuscript. Supplementary Figures and Supplementary Notes are appended at the end of the main PDF of the published manuscript.

Supplementary items should relate to a main text figure, wherever possible, and should be mentioned sequentially in the main manuscript, designated as Supplementary Figure, Table, Video, or Note, and numbered continuously (e.g. Supplementary Figure 1, Supplementary Figure 2, Supplementary Table 1, Supplementary Table 2 etc.).

Unprocessed scans of all key data generated through electrophoretic separation techniques need to be presented in a supplementary figure that should be labelled and numbered as the final supplementary figure, and should be mentioned in every relevant figure legend. This figure does not count towards the total number of figures and is the only figure that can be displayed over multiple pages, but should be provided as a single file, in PDF or TIFF format. Data in this figure can be displayed in a relatively informal style, but size markers and the figures panels corresponding to the presented data must be indicated.

The total number of Supplementary Figures (not including the “unprocessed scans” Supplementary Figure) should not exceed the number of main display items (figures and/or tables (see our Guide to Authors and March 2012 editorial http://www.nature.com/ncb/authors/submit/index.html#suppinfo; http://www.nature.com/ncb/journal/v14/n3/index.html#ed). No restrictions apply to Supplementary Tables or Videos, but we advise authors to be selective in including supplemental data.

Each Supplementary Figure should be provided as a single page and as an individual file in one of our accepted figure formats and should be presented according to our figure guidelines (see above). Supplementary Tables should be provided as individual Excel files. Supplementary Videos should be provided as .avi or .mov files up to 50 MB in size. Supplementary Figures, Tables and Videos much be accompanied by a separate Word document including titles and legends.

GUIDELINES FOR EXPERIMENTAL AND STATISTICAL REPORTING

REPORTING REQUIREMENTS – We are trying to improve the quality of methods and statistics reporting in our papers. To that end, we are now asking authors to complete a reporting summary that collects information on experimental design and reagents. The Reporting Summary can be found here https://www.nature.com/documents/nr-reporting-summary.pdf)If you would like to reference the guidance text as you complete the template, please access these flattened versions at http://www.nature.com/authors/policies/availability.html.

STATISTICS – Wherever statistics have been derived the legend needs to provide the n number (i.e. the sample size used to derive statistics) as a precise value (not a range), and define what this value represents. Error bars need to be defined in the legends (e.g. SD, SEM) together with a measure of centre (e.g. mean, median). Box plots need to be defined in terms of minima, maxima, centre, and percentiles. Ranges are more appropriate than standard errors for small data sets. Wherever statistical significance has been derived, precise p values need to be provided and the statistical test used needs to be stated in the legend. Statistics such as error bars must not be derived from n<3. For sample sizes of n<5 please plot the individual data points rather than providing bar graphs. Deriving statistics from technical replicate samples, rather than biological replicates is strongly discouraged. Wherever statistical significance has been derived, precise p values need to be provided and the statistical test stated in the legend.

Information on how many times each experiment was repeated independently with similar results needs to be provided in the legends and/or Methods for all experiments, and in particular wherever representative experiments are shown.

We strongly recommend the presentation of source data for graphical and statistical analyses as a separate Supplementary Table, and request that source data for all independent repeats are provided when representative experiments of multiple independent repeats, or averages of two independent experiments are presented. This supplementary table should be in Excel format, with data for different figures provided as different sheets within a single Excel file. It should be labelled and numbered as one of the supplementary tables, titled “Statistics Source Data”, and mentioned in all relevant figure legends.

--------- Please don't hesitate to contact NCB@nature.com should you have queries about any of the above requirements ---------

Version 1:

Decision Letter:

*Please delete the link to your author homepage if you wish to forward this email to co-authors.

Dear Professor Ramadan,

Thank you for submitting your revised manuscript, "Nucleophagy removes cytotoxic trapped PARP1", to the journal and for your patience. As you know, the original reviewers have examined your revision and two had remaining concerns, which we felt were important as these concerns pertained to evidence central to your model and the implication of nucleophagy. We thank you for providing responses to those points, which we have now discussed with Rev#1 and Rev#2. Although we are also very interested in the study, we believe that their concerns should be addressed before we can consider publication in Nature Cell Biology.

We have discussed your feedback and theirs and encourage you to address the final comments as follows:

- Rev#2 was not convinced by your responses to their point #1 and asked that you please remove Fig. 2I, Movies 1 and 2, as your main point is already sufficiently supported by the results shown in Fig. 2G and H.

- Rev#1 provided responses on your revision plans. In response to "In Fig. S2A the authors show that knockdown of ATG7 partially rescues the increased cell viability of torin treated cells on talazoparib and conclude that increased viability conferred by torin is due to autophagy. Considering that knockdown of ATG7 reduces cell viability on talazoparib (Fig 1E), the effects could be simply additive and it is difficult to conclude that torin increases viability due to autophagy induction", please at least discuss the caveats of the ATG7 partial rescue in the manuscript itself.

For the two points "The data presented in Fig S2E do not agree with the provided text, that p62 localization to the nucleus is unaffected by any of the treatments, as differences appear among different experimental conditions, at least by eye. Please provide a quantification and information of independent experimental repeats that support this conclusion." and "To validate the novel mCherry-PARP1-GFP reporter the idea is to detect whether there are additional to full length (possibly cleavage) products identified in the blot, as the one above the 100kD marker band (Fig. S3E, GFP). Whole PARP1, RFP and GFP blots are needed in control, trapping, Baf A1 and trapping/Baf A1 conditions", the reviewer indicated that text edits would not be sufficient. Experimentally addressing these points will be important with quantifications and control gels across the conditions specified by the reviewer as detailed by the reviewer in their re-review comments.

- Please address all other points with edits to the manuscript text and along the lines suggested in your plans.

- Finally, please pay close attention to our guidelines on statistical and methodological reporting (listed below), as failure to do so may delay the reconsideration of the revised manuscript. In particular, please provide:

- a Supplementary Figure including unprocessed images of all gels/blots in the form of a multi-page pdf file. Please ensure that blots/gels are labeled and the sections presented in the figures are clearly indicated.

- a Supplementary Table including all numerical source data in Excel format, with data for different figures provided as different sheets within a single Excel file. The file should include source data giving rise to graphical representations and statistical descriptions in the paper and for all instances where the figures present representative experiments of multiple independent repeats, the source data of all repeats should be provided.

We therefore invite you to take these points into account when revising the manuscript. In addition, when preparing the revision please:

- ensure that it conforms to our format instructions and publication policies (see below and https://www.nature.com/nature/for-authors).

- provide a point-by-point rebuttal to the full referee reports verbatim, as provided at the end of this letter.

- for any revision that includes light microscopy data, we ask our authors to please include a completed light microscopy reporting table https://www.nature.com/documents/Light_microscopy_reporting_table.xlsx to ensure the methods are described thoroughly. The table will be available to reviewers and ultimately published should the manuscript be accepted at the journal.

- provide the completed Reporting Summary (found here https://www.nature.com/documents/nr-reporting-summary.pdf). This is essential for reconsideration of the manuscript and will be available to editors and referees in the event of peer review. For more information see http://www.nature.com/authors/policies/availability.html or contact me.

When submitting the revised version of your manuscript, please pay close attention to our href="https://www.nature.com/nature-portfolio/editorial-policies/image-integrity">Digital Image Integrity Guidelines. and to the following points below:

-- that unprocessed scans are clearly labelled and match the gels and western blots presented in figures.
-- that control panels for gels and western blots are appropriately described as loading on sample processing controls
-- all images in the paper are checked for duplication of panels and for splicing of gel lanes.

EXTENDED DATA FIGURES

When re-submitting your manuscript, please ensure that any supplementary figures and tables that are crucial to the manuscript's conclusions are converted into Extended Data figures and tables to increase visibility of these data. Extended Data figures and tables are online-only (present in the online PDF and full-text HTML versions of the paper), peer-reviewed display items that provide essential background to the article but are not included in the main article due to space constraints. A maximum of ten Extended Data display items (figures and tables) is permitted.

Finally, please ensure that you retain unprocessed data and metadata files after publication, ideally archiving data in perpetuity, as these may be requested during the peer review and production process or after publication if any issues arise.

Nature Cell Biology is committed to improving transparency in authorship. As part of our efforts in this direction, we are now requesting that all authors identified as ‘corresponding author’ on published papers create and link their Open Researcher and Contributor Identifier (ORCID) with their account on the Manuscript Tracking System (MTS), prior to acceptance. ORCID helps the scientific community achieve unambiguous attribution of all scholarly contributions. You can create and link your ORCID from the home page of the MTS by clicking on ‘Modify my Springer Nature account’. For more information please visit please visit www.springernature.com/orcid.

This journal strongly supports public availability of data. Please place the data used in your paper into a public data repository, or alternatively, present the data as Supplementary Information. If data can only be shared on request, please explain why in your Data Availability Statement, and also in the correspondence with your editor. Please note that for some data types, deposition in a public repository is mandatory - more information on our data deposition policies and available repositories appears below.

Please submit the revised manuscript files and the point-by-point rebuttal to the referee comments using this link:

Link Redacted

*This url links to your confidential home page and associated information about manuscripts you may have submitted or be reviewing for us. If you wish to forward this email to co-authors, please delete the link to your homepage.

We would like to receive the revision within four weeks. If submitted within this time period, reconsideration of the revised manuscript will not be affected by related studies published elsewhere, or accepted for publication in Nature Cell Biology in the meantime. We would be happy to consider a revision even after this timeframe, but in that case we will consider the published literature at the time of resubmission when assessing the file.

We hope that you will find our referees' comments and editorial guidance helpful. Please do not hesitate to contact me if there is anything you would like to discuss. Thank you again for your patience.

Best wishes,

Melina

Melina Casadio, PhD
Senior Editor, Nature Cell Biology
ORCID ID: https://orcid.org/0000-0003-2389-2243

Reviewers' Comments:

Reviewer #1 (Remarks to the Author):

In their revised manuscript, now entitled “Nucleophagy removes cytotoxic trapped PARP1”, Hoslett and colleagues conducted several additional experiments to address the reviewers’ concerns.

Specifically, they provided additional evidence of the physical interaction between PARP1 and TEX264 and identify a region at the C-terminal end of TEX264 that is essential for binding to PARP1, among other functions. Further, more detailed analysis will be necessary to identify the specific amino acids involved in this interaction. Moreover, the authors examined additional autophagy factors to further support the role of autophagy in processing trapped PARP1. In fact, they tested whether components of the core autophagy machinery, which also function in nucleophagy, were necessary. Proteins for which emerging evidence suggests a role in this selective type of autophagy such as ESCRT components involved in nuclear envelop remodeling, including the ATPase VPS4, which has already been implicated in cancer development, could also have been examined.

The authors used Torin-1 to boost autophagy, noting that they were unable to identify a gene depletion approach that is routinely used for this purpose. An alternative genetic approach would be Rheb RNAi, given that Rheb is the primary direct activator of mTORC1. It is true, however, that Rheb RNAi is not without its own drawbacks.

Although the potential involvement of specific E3 ligases in removing trapped PARP1 has not been examined, I would agree that this is worth exploring in future studies.

Regarding the question of how TEX264 and p97 complexed with PARP1 cross the nuclear membrane to deliver PARP1 to lysosomes, the authors discuss several possibilities but provide no real experimental evidence.

A few additional points deserve careful consideration:

In Fig. S2A the authors show that knockdown of ATG7 partially rescues the increased cell viability of torin treated cells on talazoparib and conclude that increased viability conferred by torin is due to autophagy. Considering that knockdown of ATG7 reduces cell viability on talazoparib (Fig 1E), the effects could be simply additive and it is difficult to conclude that torin increases viability due to autophagy induction.

The data presented in Fig S2E do not agree with the provided text, that p62 localization to the nucleus is unaffected by any of the treatments, as differences appear among different experimental conditions, at least by eye. Please provide a quantification and information of independent experimental repeats that support this conclusion.

In Fig 2B PARP1 is not visible in the LysoIP sample of untreated cells (right panel second lane), while in Fig. 2C basal precipitated PARP1 levels are normalized to 1. Please provide an additional exposure setting, where PAPR1 is visible.

To validate the novel mCherry-PARP1-GFP reporter the idea is to detect whether there are additional to full length (possibly cleavage) products identified in the blot, as the one above the 100kD marker band (Fig. S3E, GFP). Whole PARP1, RFP and GFP blots are needed in control, trapping, Baf A1 and trapping/Baf A1 conditions.

The event of mCherry-PARP1 (red only) engulfment into the lysosome mentioned at the time frame of 9min (Fig. 2I) is followed by clear and robust appearance or restoration of GFP fluorescence at least at the time frames of 12-13-18min (surprisingly within the lysosome signal). What could this observation reflect? This result further illustrates the need for deeper validation of the novel mCherry-PARP1-GFP reporter assay.

The authors report that TEX264 plays a role both in recruiting p97 to trapped PARP1 facilitating unfolding and detachment from chromatin (before aggregation) as well as acting as an autophagy receptor to aggregated PARP1 at the nuclear membrane. It seems that PARP1-TEX264 interaction preexists PARP1 aggregation. While, a role of TEX264 in PARP1 autophagic degradation is supported, the TEX264 engagement to non-aggregated trapped PARP1 contradicts the notion of a p97-TEX264-nucleophagy axis, selective for degradation of aggregated PARP1 (summary point 4 of rebuttal).

Previous and current findings propose that the C-terminal region of TEX264 is essential for both PARP1 and LC3 interaction, among other interactions. Are then TEX264-PARP1 and TEX264-LC3 interactions mutually exclusive? If the above aggregation is PARP1-dependent (not TEX264-dependent), a ternary LC3 (or an Atg8 family member)-TEX264-PARP1 complex cannot be formed and therefore TEX264 cannot serve as an autophagy receptor for PARP1. Does a C-terminal deletion mutant that does not include the LIR motif interact with PARP1 (Fig. 5E)? Does LC3 compete the PARP-1 interaction with either of these two TEX264 truncated constructs (with and without the LIR motif)? Similar concerns about mutual exclusive competition can be raised regarding the TEX264-p97 and TEX264-PARP1 interactions. Alternatively, does the C-terminal region of TEX264 constitute a loosely structured or contain an intrinsically disordered domain, prone to unspecific interactions at least in vitro?

Minor:
Autophagophore membrane / autophagophore formation are not the standard terminology. Instead, the term "phagophore" is predominantly used to describe the early autophagic membrane structure, while usually "autophagosome formation" is used to describe the generation of autophagic vesicles.

Please provide in text citations for lines 277-279.

In line 264 the authors introduce veliparib as a PARPi that causes no PARP1 trapping and functions through catalytic inactivation of PARP1. However, in line 293 the authors state that veliparib is a weaker trapper of PARP1.

In Fig. 3H the authors may want to change mean puncta per cell to average number of puncta per cell.
In Fig 3G correct HA label to HA (TMEM192).

The schematic in Fig S5C is illegible. Please increase font size. Please also include a label that these are TEX264 constructs

Fig 6 requires some alignment / rearrangement for clarity and adequate panel separation.

Line 530: Provide the (ref)

Fig. S6C: Please include the normalized PARP1 levels for the non-Tala+MMS condition as in Fig. S6D to show PARP1 lysosomal enrichment upon PARP1 trapping.

The criticism of reviewer#1 concerned the term "selective nucleophagy", not nucleophagy per se, which is already a selective type of autophagy targeting nuclear material for degradation. In accordance with comment no.24, please make the necessary correction on page 18, line 582, "TEX264-driven selective nucleophagy of trapped…".

The concept of autophagy inhibition at the level of either ATG7, ATG9A or Beclin-1 to "evidence that PARP1 localization to the lysosome is autophagy dependent without relying on changes to LC3 (lipidation)" is problematic (last paragraph, last sentence). Shouldn't genetic inhibition of autophagy in these three different stages have some impact on the downstream LC3 lipidation? The authors also stated at the same time that the "purpose of ATG7 depletion was to inhibit autophagy by reducing LC3 lipidation" (same last paragraph, first sentence).

While lysosomal acidification may not be a prerequisite for autophagosome-lysosome fusion as V-ATPase-deficient lysosomes remain competent to fuse with autophagosomes and endosomes, Bafilomycin A1 (which is utilized here, Hoslett et al) still prevents autophagosome-lysosome fusion under V-ATPase-deficiency (Mauvezin, Nagy et al. 2015). Therefore, authors in the cited study (Mauvezin, Nagy et al. 2015) suggested that BafilomycinA1 inhibits fusion independent of its effect on lysosomal pH. Thus, to our current understanding, Bafilomycin A1 treatment possibly cannot facilitate accumulation and detection of autophagic cargo in the lysosome in the described experimental setting here, and the respective findings may deserve reconsideration.

Reviewer #2 (Remarks to the Author):

The authors have addressed most of the issues I raised in the review of the original manuscript. However, I still have a few remaining concerns.

1. Fig. 2I, Movies 1 and 2: The replaced images are still unconvincing and do not adequately support the authors' claim. Why do the mCherry-PARP1-GFP puncta appear in red from the nucleus, rather than as puncta positive for both green and red signals? It is also unclear whether the puncta originated from the nucleus. Although it is reasonable to interpret the loss of the PARP1 signal as reflecting its degradation, why does the lysosomal signal also disappear?

2. Fig. 2H: In the middle graph, the "+" should be removed from the "TalA+MMS" row of the fourth bar.

Reviewer #3 (Remarks to the Author):

The revised manuscript addressed all of my major concerns.

GUIDELINES FOR SUBMISSION OF NATURE CELL BIOLOGY ARTICLES

READABILITY OF MANUSCRIPTS – Nature Cell Biology is read by cell biologists from diverse backgrounds, many of whom are not native English speakers. Authors should aim to communicate their findings clearly, explaining technical jargon that might be unfamiliar to non-specialists, and avoiding non-standard abbreviations. Titles and abstracts should concisely communicate the main findings of the study, and the background, rationale, results and conclusions should be clearly explained in the manuscript in a manner accessible to a broad cell biology audience. Nature Cell Biology uses British spelling.

ARTICLE FORMAT

TITLE – should be no more than 100 characters including spaces, without punctuation and avoiding technical terms, abbreviations, and active verbs..

AUTHOR NAMES – should be given in full.

AUTHOR AFFILIATIONS – should be denoted with numerical superscripts (not symbols) preceding the names. Full addresses should be included, with US states in full and providing zip/post codes. The corresponding author is denoted by: "Correspondence should be addressed to [initials]."

ABSTRACT – should not exceed 150 words and should be unreferenced. This paragraph is the most visible part of the paper and should briefly outline the background and rationale for the work, and accurately summarize the main results and conclusions. Key genes, proteins and organisms should be specified to ensure discoverability of the paper in online searches.

TEXT – the main text consists of the Introduction, Results, and Discussion sections and must not exceed 3500 words including the abstract. The Introduction should expand on the background relating to the work. The Results should be divided in subsections with subheadings, and should provide a concise and accurate description of the experimental findings. The Discussion should expand on the findings and their implications. All relevant primary literature should be cited, in particular when discussing the background and specific findings.

ACKNOWLEDGEMENTS – should be kept brief. Professional titles and affiliations are unnecessary. Grant numbers can be

listed.

AUTHOR CONTRIBUTIONS – must be included after the Acknowledgements, detailing the contributions of each author to the paper (e.g. experimental work, project planning, data analysis etc.). Each author should be listed by his/her initials.

FINANCIAL AND NON-FINANCIAL COMPETING INTERESTS – the authors must include one of three declarations: (1) that they have no financial and non-financial competing interests; (2) that they have financial and non-financial competing interests; or (3) that they decline to respond, after the Author Contributions section. This statement will be published with the article, and in cases where financial and non-financial competing interests are declared, these will be itemized in a web supplement to the article. For further details please see https://www.nature.com/licenceforms/nrg/competing-interests.pdf.

REFERENCES – are limited to a total of 70 in the main text and Methods combined,. They must be numbered sequentially as they appear in the main text, tables and figure legends and Methods and must follow the precise style of Nature Cell Biology references. References only cited in the Methods should be numbered consecutively following the last reference cited in the main text. References only associated with Supplementary Information (e.g. in supplementary legends) do not count toward the total reference limit and do not need to be cited in numerical continuity with references in the main text. Only published papers can be cited, and each publication cited should be included in the numbered reference list, which should include the manuscript titles. Footnotes are not permitted.

METHODS – Nature Cell Biology publishes methods online. The methods section should be provided as a separate Word document, which will be copyedited and appended to the manuscript PDF, and incorporated within the HTML format of the paper.

Methods should be written concisely, but should contain all elements necessary to allow interpretation and replication of the results. As a guideline, Methods sections typically do not exceed 3,000 words. The Methods should be divided into subsections listing reagents and techniques. When citing previous methods, accurate references should be provided and any alterations should be noted. Information must be provided about: antibody dilutions, company names, catalogue numbers and clone numbers for monoclonal antibodies; sequences of RNAi and cDNA probes/primers or company names and catalogue numbers if reagents are commercial; cell line names, sources and information on cell line identity and authentication. Animal studies and experiments involving human subjects must be reported in detail, identifying the committees approving the protocols. For studies involving human subjects/samples, a statement must be included confirming that informed consent was obtained. Statistical analyses and information on the reproducibility of experimental results should be provided in a section titled “Statistics and Reproducibility”.

All Nature Cell Biology manuscripts submitted on or after March 21 2016, must include a Data availability statement as a separate section after Methods but before references, under the heading "Data Availability”. For Springer Nature policies on data availability see http://www.nature.com/authors/policies/availability.html; for more information on this particular policy see http://www.nature.com/authors/policies/data/data-availability-statements-data-citations.pdf. The Data availability statement should include:

• Accession codes for primary datasets (generated during the study under consideration and designated as "primary accessions") and secondary datasets (published datasets reanalysed during the study under consideration, designated as "referenced accessions"). For primary accessions data should be made public to coincide with publication of the manuscript. A list of data types for which submission to community-endorsed public repositories is mandated (including sequence, structure, microarray, deep sequencing data) can be found here http://www.nature.com/authors/policies/availability.html#data.

• Unique identifiers (accession codes, DOIs or other unique persistent identifier) and hyperlinks for datasets deposited in an approved repository, but for which data deposition is not mandated (see here for details http://www.nature.com/sdata/data-policies/repositories).

• At a minimum, please include a statement confirming that all relevant data are available from the authors, and/or are included with the manuscript (e.g. as source data or supplementary information), listing which data are included (e.g. by figure panels and data types) and mentioning any restrictions on availability.

• If a dataset has a Digital Object Identifier (DOI) as its unique identifier, we strongly encourage including this in the Reference list and citing the dataset in the Methods.

We recommend that you upload the step-by-step protocols used in this manuscript to protocols.io. More details can found at https://www.protocols.io/help/publish-articles.

DISPLAY ITEMS – main display items are limited to 6-8 main figures and/or main tables. For Supplementary Information see below.

FIGURES – Colour figure publication costs $395 per colour figure. All panels of a multi-panel figure must be logically connected and arranged as they would appear in the final version. Unnecessary figures and figure panels should be avoided (e.g. data presented in small tables could be stated briefly in the text instead).

All imaging data should be accompanied by scale bars, which should be defined in the legend.
Cropped images of gels/blots are acceptable, but need to be accompanied by size markers, and to retain visible background signal within the linear range (i.e. should not be saturated). The boundaries of panels with low background have to be demarked with black lines. Splicing of panels should only be considered if unavoidable, and must be clearly marked on the figure, and noted in the legend with a statement on whether the samples were obtained and processed simultaneously. Quantitative comparisons between samples on different gels/blots are discouraged; if this is unavoidable, it has be performed for samples derived from the same experiment with gels/blots were processed in parallel, which needs to be stated in the legend.

Figures should be provided at approximately the size that they are to be printed at (single column is 86 mm, double column is 170 mm) and should not exceed an A4 page (8.5 x 11"). Reduction to the scale that will be used on the page is not necessary, but multi-panel figures should be sized so that the whole figure can be reduced by the same amount at the smallest size at which essential details in each panel are visible. In the interest of our colour-blind readers we ask that you avoid using red and green for contrast in figures. Replacing red with magenta and green with turquoise are two possible colour-safe alternatives. Lines with widths of less than 1 point should be avoided. Sans serif typefaces, such as Helvetica (preferred) or Arial should be used. All text that forms part of a figure should be rewritable and removable.

We accept files from the following graphics packages in either PC or Macintosh format:

- For line art, graphs, charts and schematics we prefer Adobe Illustrator (.AI), Encapsulated PostScript (.EPS) or Portable Document Format (.PDF). Files should be saved or exported as such directly from the application in which they were made, to allow us to restyle them according to our journal house style.

- We accept PowerPoint (.PPT) files if they are fully editable. However, please refrain from adding PowerPoint graphical effects to objects, as this results in them outputting poor quality raster art. Text used for PowerPoint figures should be Helvetica (preferred) or Arial.

- We do not recommend using Adobe Photoshop for designing figures, but we can accept Photoshop generated (.PSD or .TIFF) files only if each element included in the figure (text, labels, pictures, graphs, arrows and scale bars) are on separate layers. All text should be editable in 'type layers' and line-art such as graphs and other simple schematics should be preserved and embedded within 'vector smart objects' - not flattened raster/bitmap graphics.

- Some programs can generate Postscript by 'printing to file' (found in the Print dialogue). If using an application not listed above, save the file in PostScript format or email our Art Editor, Allen Beattie for advice (a.beattie@nature.com).

Regardless of format, all figures must be vector graphic compatible files, not supplied in a flattened raster/bitmap graphics format, but should be fully editable, allowing us to highlight/copy/paste all text and move individual parts of the figures (i.e. arrows, lines, x and y axes, graphs, tick marks, scale bars etc). The only parts of the figure that should be in pixel raster/bitmap format are photographic images or 3D rendered graphics/complex technical illustrations.

All placed images (i.e. a photo incorporated into a figure) should be on a separate layer and independent from any superimposed scale bars or text. Individual photographic images must be a minimum of 300+ DPI (at actual size) or kept constant from the original picture acquisition and not decreased in resolution post image acquisition. All colour artwork should be RGB format.

FIGURE LEGENDS – must not exceed 350 words for each figure to allow fit on a single printed NCB page together with the figure. They must include a brief title for the whole figure, and short descriptions of each panel with definitions of the symbols used, but without detailing methodology.

TABLES – main tables should be provided as individual Word files, together with a brief title and legend. For supplementary tables see below.

SUPPLEMENTARY INFORMATION – Supplementary information is material directly relevant to the conclusion of a paper, but which cannot be included in the printed version in order to keep the manuscript concise and accessible to the general reader. Supplementary information is an integral part of a Nature Cell Biology publication and should be prepared and presented with as much care as the main display item, but it must not include non-essential data or text, which may be removed at the editor's discretion. All supplementary material is fully peer-reviewed and published online as part of the HTML version of the manuscript. Supplementary Figures and Supplementary Notes are appended at the end of the main PDF of the published manuscript.

Supplementary items should relate to a main text figure, wherever possible, and should be mentioned sequentially in the main manuscript, designated as Supplementary Figure, Table, Video, or Note, and numbered continuously (e.g. Supplementary Figure 1, Supplementary Figure 2, Supplementary Table 1, Supplementary Table 2 etc.).

Unprocessed scans of all key data generated through electrophoretic separation techniques need to be presented in a supplementary figure that should be labeled and numbered as the final supplementary figure, and should be mentioned in every relevant figure legend. This figure does not count towards the total number of figures and is the only figure that can be

displayed over multiple pages, but should be provided as a single file, in PDF or TIFF format. Data in this figure can be displayed in a relatively informal style, but size markers and the figures panels corresponding to the presented data must be indicated.

The total number of Supplementary Figures (not including the “unprocessed scans” Supplementary Figure) should not exceed the number of main display items (figures and/or tables (see our Guide to Authors and March 2012 editorial http://www.nature.com/ncb/authors/submit/index.html#suppinfo; http://www.nature.com/ncb/journal/v14/n3/index.html#ed). No restrictions apply to Supplementary Tables or Videos, but we advise authors to be selective in including supplemental data.

Each Supplementary Figure should be provided as a single page and as an individual file in one of our accepted figure formats and should be presented according to our figure guidelines (see above). Supplementary Tables should be provided as individual Excel files. Supplementary Videos should be provided as .avi or .mov files up to 50 MB in size. Supplementary Figures, Tables and Videos much be accompanied by a separate Word document including titles and legends.

GUIDELINES FOR EXPERIMENTAL AND STATISTICAL REPORTING

REPORTING REQUIREMENTS – We ask authors to complete a Reporting Summary that collects information on experimental design and reagents. We hope this will aid in your evaluation of the paper. The Reporting Summary can be found here https://www.nature.com/documents/nr-reporting-summary.pdf) Please note that these forms are dynamic ‘smart pdfs’ and must therefore be downloaded and completed in Adobe Reader. We will then flatten them for ease of use. If you would like to reference the guidance text as you complete the template, please access these flattened versions at http://www.nature.com/authors/policies/availability.html.

STATISTICS – Wherever statistics have been derived the legend needs to provide the n number (i.e. the sample size used to derive statistics) as a precise value (not a range), and define what this value represents. Error bars need to be defined in the legends (e.g. SD, SEM) together with a measure of centre (e.g. mean, median). Box plots need to be defined in terms of minima, maxima, centre, and percentiles. Ranges are more appropriate than standard errors for small data sets. Wherever statistical significance has been derived, precise p values need to be provided and the statistical test used needs to be stated in the legend. Statistics such as error bars must not be derived from n<3. For sample sizes of n<5 please plot the individual data points rather than providing bar graphs. Deriving statistics from technical replicate samples, rather than biological replicates is strongly discouraged. Wherever statistical significance has been derived, precise p values need to be provided and the statistical test stated in the legend.

Information on how many times each experiment was repeated independently with similar results needs to be provided in the legends and/or Methods for all experiments, and in particular wherever representative experiments are shown.

We strongly recommend the presentation of source data for graphical and statistical analyses as a separate Supplementary Table, and request that source data for all independent repeats are provided when representative experiments of multiple independent repeats, or averages of two independent experiments are presented. This supplementary table should be in Excel format, with data for different figures provided as different sheets within a single Excel file. It should be labelled and numbered as one of the supplementary tables, titled “Statistics Source Data”, and mentioned in all relevant figure legends.

--------- Please don't hesitate to contact NCB@nature.com should you have queries about any of the above requirements ---------

Version 2:

Decision Letter:

Our ref: NCB-A55402B

4th March 2026

Dear Dr. Ramadan,

Thank you for submitting your revised manuscript "Nucleophagy removes cytotoxic trapped PARP1" (NCB-A55402B). It has now been seen by the original referees and their comments are below. The reviewers find that the paper has improved in revision, and therefore we'll be happy in principle to publish it in Nature Cell Biology, pending minor revisions to satisfy the referees' final requests and to comply with our editorial and formatting guidelines. We agree with Rev#2's suggestion to add discussion of the potential for micro-nucleophagy to be at work.

We are now performing detailed checks on your paper and will send you a checklist detailing our editorial and formatting requirements in about 1-2 weeks. Please do not upload the final materials and make any revisions until you receive this additional information from us.

Thank you again for your interest in Nature Cell Biology. Please do not hesitate to contact me if you have any questions.

Sincerely,

Melina Casadio, PhD
Senior Editor, Nature Cell Biology
ORCID ID: https://orcid.org/0000-0003-2389-2243

Reviewer #1 (Remarks to the Author):

The authors have now adequately addressed my remaining concerns and I have no further comments.

Reviewer #2 (Remarks to the Author):

In the new figures and movies, the lysosome signal is retained after the disappearance of the red PARP1 signal, consistent with the notion that PARP1 is degraded within the lysosome. Meanwhile, again, an mCherry-positive but GFP-negative punctum emerges in the vicinity of the nucleus where a lysosome already awaits. This image appears to capture a microautophagy process, in which the lysosome directly engulfs PARP1 without its sequestration within an autophagosome, rather than macroautophagy the authors assume in this study. If this observation is reproducible and represents a major pathway for lysosomal degradation of PARP1, the authors should clearly discuss, based on this observation, the possibility that PARP1 degradation may be mediated by micronucleophagy, also given that there is no conclusive morphological evidence showing that PARP1 is sequestered within autophagosomes before being transported to lysosomes.

Version 3:

Decision Letter:

Dear Dr Ramadan,

I am pleased to inform you that your manuscript, "Nucleophagy removes cytotoxic trapped PARP1", has now been accepted for publication in Nature Cell Biology.

Thank you for sending us the final manuscript files to be processed for print and online production, and for returning the manuscript checklists and other forms. Your manuscript will now be passed to our production team who will be in contact with you if there are any questions with the production quality of supplied figures and text.

Over the next few weeks, your paper will be copyedited to ensure that it conforms to Nature Cell Biology style. Once your paper is typeset, you will receive an email with a link to choose the appropriate publishing options for your paper and our Author Services team will be in touch regarding any additional information that may be required.

After the grant of rights is completed, you will receive a link to your electronic proof via email with a request to make any corrections within 48 hours. If, when you receive your proof, you cannot meet this deadline, please inform us at rjsproduction@springernature.com immediately.

You will not receive your proofs until the publishing agreement has been received through our system.

Due to the importance of these deadlines, we ask that you please let us know now whether you will be difficult to contact over the next month. If this is the case, we ask you provide us with the contact information (email, phone and fax) of someone who will be able to check the proofs on your behalf, and who will be available to address any last-minute problems.

If you have any questions about our publishing options, costs, Open Access requirements, or our legal forms, please contact ASJournals@springernature.com

Once your paper has been scheduled for online publication, the Nature press office will be in touch to confirm the details. An online order form for reprints of your paper is available at https://www.nature.com/reprints/author-reprints.html. All co-authors, authors' institutions and authors' funding agencies can order reprints using the form appropriate to their geographical region.

Publication is conditional on the manuscript not being published elsewhere and on there being no announcement of this work to any media outlet until the online publication date in Nature Cell Biology.

You may wish to make your media relations office aware of your accepted publication, in case they consider it appropriate to organize some internal or external publicity. Once your paper has been scheduled you will receive an email confirming the

publication details. This is normally 3-4 working days in advance of publication. If you need additional notice of the date and time of publication, please let the production team know when you receive the proof of your article to ensure there is sufficient time to coordinate. Further information on our embargo policies can be found here: https://www.nature.com/authors/policies/embargo.html

Please note that *Nature Cell Biology* is a Transformative Journal (TJ). Authors may publish their research with us through the traditional subscription access route or make their paper immediately open access through payment of an article-processing charge (APC). Authors will not be required to make a final decision about access to their article until it has been accepted. Find out more about Transformative Journals

Authors may need to take specific actions to achieve compliance with funder and institutional open access mandates. If your research is supported by a funder that requires immediate open access (e.g. according to Plan S principles or the NIH public access policy) then you should select the gold OA route, and we will direct you to the compliant route where possible. Because authors warrant under our subscription licensing terms that they haven't committed to licensing any version of their article under a licence inconsistent with the terms of our agreement – including the applicable embargo period – publication under the subscription model isn't suitable for authors whose funders require no embargo.

To assist our authors in disseminating their research to the broader community, our SharedIt initiative provides you with a unique shareable link that will allow anyone (with or without a subscription) to read the published article. Recipients of the link with a subscription will also be able to download and print the PDF.

If your paper includes color figures, please be aware that in order to help cover some of the additional cost of four-color reproduction, Nature Portfolio charges our authors a fee for the printing of their color figures. Please contact our offices for exact pricing and details.

As soon as your article is published, you will receive an automated email with your shareable link.

If you have not already done so, we strongly recommend that you upload the step-by-step protocols used in this manuscript to protocols.io (https://protocols.io), an open online resource that allows researchers to share their detailed experimental know-how. All uploaded protocols are made freely available and are assigned DOIs for ease of citation. Protocols and Nature Portfolio journal papers in which they are used can be linked to one another, and this link is clearly and prominently visible in the online versions of both. Authors who performed the specific experiments can act as primary authors for the Protocol as they will be best placed to share the methodology details, but the Corresponding Author of the present research paper should be included as one of the authors. By uploading your Protocols onto protocols.io, you are enabling researchers to more readily reproduce or adapt the methodology you use, as well as increasing the visibility of your protocols and papers. You can also establish a dedicated workspace to collect your lab Protocols. Further information can be found at https://www.protocols.io/help/publish-articles.

You can use a single sign-on for all your accounts, view the status of all your manuscript submissions and reviews, access usage statistics for your published articles and download a record of your refereeing activity for the Nature Portfolio.

Nature Cell Biology encourages authors presenting evidence for cell, biological, molecular, and genetic interactions to consider communicating these findings using Biofactoid (https://biofactoid.org/). This tool helps users share a searchable representation of interactions (e.g. binding, gene expression, post-translational modification) between genes, gene products, or chemicals. Information added to Biofactoid, with author attribution, is shared on social media and public databases, such as Pathway Commons, where it can be discovered and analyzed in the context of a large and growing corpus of knowledge.

Please feel free to contact us if you have any questions.

With kind regards,

Melina Casadio, PhD
Senior Editor, Nature Cell Biology
ORCID ID: https://orcid.org/0000-0003-2389-2243

Click here if you would like to recommend Nature Cell Biology to your librarian
http://www.nature.com/subscriptions/recommend.html#forms

** Visit the Springer Nature Editorial and Publishing website at www.springernature.com/editorial-and-publishing-jobs for more information about our career opportunities. If you have any questions please click here.**

Open Access This Peer Review File is licensed under a Creative Commons Attribution 4.0 International License, which permits use, sharing, adaptation, distribution and reproduction in any medium or format, as long as you give appropriate credit to the original author(s) and the source, provide a link to the Creative Commons license, and indicate if changes were made.
In cases where reviewers are anonymous, credit should be given to 'Anonymous Referee' and the source.
The images or other third party material in this Peer Review File are included in the article's Creative Commons license, unless indicated otherwise in a credit line to the material. If material is not included in the article's Creative Commons license and your intended use is not permitted by statutory regulation or exceeds the permitted use, you will need to obtain permission directly from the copyright holder.
To view a copy of this license, visit https://creativecommons.org/licenses/by/4.0/

We would like to sincerely thank the referees for their constructive comments on our manuscript and suggestions on how to improve our discovery of selective autophagy in the processing of trapped PARP1, a specific type of DNA lesion important for cancer therapy of HR-deficient cancers. We have seriously taken their comments/criticisms/suggestions, as well as the suggestions from the editorial team on how to improve and prepare the revised manuscript. We believe we have experimentally addressed the majority of their criticisms in the revised manuscript, which further supports our earlier findings.

Specifically, in the revised version, we have experimentally addressed as follows:

1. Demonstrated a direct physical interaction between PARP1 and TEX264 and mapped the interaction sites (Figures 5D and E, Sup. Figs 5A-D)
2. Demonstrated the role of additional autophagy factors (ATG9A and Beclin-1) in processing trapped PARP1. By the inactivation of several well-known autophagy factors in our experiments, we have demonstrated their role in cell survival in response to PARP inhibitors (trapped PARP) and delivery of trapped PARP to the lysosome (Figure 3E). We further confirmed that the resistance to talazoparib caused by Torin treatment was autophagy dependent, as it is reversed by depletion of ATG7(Fig. S2A).
3. Addressed the key question in the field of nucleophagy: Why do cells need to use this mechanism for removing nuclear-trapped PARP1 if there are already many other well-described DNA repair mechanisms, including the p97-Ufd1-SPRTN-proteasome degradation pathway(s)? We now show by biochemical and cell biological methods that trapped PARP1 causes aggregates. As the aggregated proteins can not be resolved by the SPRTN or proteasomal proteases, nucleophagy is the key process to clear cytotoxic PARP1 aggregates (Fig. 6C-E).
4. To support our claims in point 3, we also depleted UFD1 (a component of p97-UFD1 complex previously described for the removal of trapped PARP1 by the proteasome) in TEX264-knock-out cells and observed that UFD1 inactivation has an additive effect in cell sesntivity to the PARP1 inhibitor Talazoparib (Fig. 6A). A similar additive effect was observed when TEX264-knock-out cells were treated with p97 inhibitor in combination with PARP inhibitor (Fig 6B). Altogether, this experiment suggests two parallel pathways for the removal of trapped PARP1 by the p97 system: (I) p97-UFD1-Proteasome degradation of soluble trapped PARP1, (ii) p97-TEX264-nucleophagy degradation of aggregated PARP1.
5. We showed that here described nucelophagy is also induced by Niraparib, another clinically relevant PARPinhibitor that causes strong PARP1 trapping, further demonstrating that this is a general phenomenon for trapped PARP1 and not only induced by Talazoparib. In contrast, we demonstrated that a poor PARP1 trapper, Veliparib, does not induce delivery of PARP to the lysosome (Figures 1F, 2D, S3C-D, S4A).
6. We demonstrated that the exit of trapped PARP from the nucleus to the lysosome is independent of the nuclear pore complex, as inhibiting nuclear pore transport by Leptomicin B did not affect the delivery of trapped PARP1 to the lysosome (Fig. S6B and C). However, we observed lamin-A/C accumulation in the lysosome in response to PARPi in a leptomycin-B-independent manner, and that PARP1 localisation to the lysosome was impaired by inhibition of ATR (Figures S6D and E). As ATR has recently been shown to induce localised nuclear envelope rupture in response to DNA damage through phosphorylation of the nuclear lamina, we suggested that PARP exit from the nucleus is through localised ruptures, as was shown for TOP1cc in our previous work.
7. We further confirmed the localisation of TEX264 at the inner nuclear envelope and improved live imaging of PARP1 exit from the nucleus towards the lysosome after PARP inhibitor treatments (Figures S6A). We further improved the mCherry-GFP

reporter assay by adding additional controls (Fig. 2G and H) and improving the live imaging by optimising the assay and performing image rendering (Fig. 2I, Movies 1 and 2).

8. We included additional controls for lyso-IP experiments (Fig. 2B-E).
9. We further demonstrated that sensitivity caused by TEX264-knock-out is trapping-dependent by showing that sensitivity is not observed in cells carrying a trapping-defective variant of PARP1 (F44) (Fig. 4G).
10. We demonstrated that TEX264 is key for recruitment of p97 to trapped PARP1 by showing reduced p97-PARP1 interaction on chromatin when TEX264 is depleted (Fig. 5A- C).
11. We analysed clinical data and demonstrated that TEX264 expression in homologous recombination-deficient breast cancer patients significantly and strongly correlates with the overall patient survival (Fig. 6H, I and Suppl. Fig. 10). These clinical data strengthen the relevance of our discovery for breast cancer patients.

Together, this provided further evidence and clarification for our model. In short, TEX264 acts to recruit p97 to trapped PARP1, allowing it to be unfolded and cleared from chromatin. Unfolded PARP1 is prone to form aggregates, which are cleared by selective autophagy through TEX264 acting as an autophagy receptor at the nuclear membrane. If this process is impaired, cells are hypersensitised to PARPi through increased DNA damage and accumulation of cytotoxic protein aggregates.

Please see below our specific response to each of the reviewers' comments in blue.

Reviewer #1 (Remarks to the Author):

In this manuscript, Hoslett and colleagues aimed to elucidate the mechanisms mediating the clearance of trapped Poly (ADP-ribose) polymerase (PARP1) from chromatin, thus conferring resistance to PARPi with clinical implications for cancer treatment. Using RNA seq on triple-negative breast cancer CAL51 cells after 24 hours treatment with the strong trapping PARPi talazoparib, they identified genes that are differentially expressed upon treatment with PARP inhibitors (PARPi). Genes related to DNA damage repair, replication stress, apoptosis, G1/S cell cycle checkpoint and mitotic checkpoint were found to be upregulated in treated cells compared to controls. In addition, genes of the autophagy core machinery as well as autophagy modulators such as TP53INP1, DRAM1, PMAIP1, MYO6, EI24 and negative regulators of mTOR signaling, which stimulates autophagy, SESN1 and SESN2, were significantly upregulated in treated cells compared to controls. Unlike autophagy, genes involved in proteosomal-mediated degradation were not significantly induced by PARPi treatment. Complementary to the above-mentioned RNA seq analysis, two whole genome CRISPR screens for loss of autophagy factors affecting sensitivity to PARPi also revealed several autophagy-related genes that were significantly represented among the candidate PARPi sensitivity genes, suggesting that loss of some genes causes sensitivity to PARPi. Mass spectrometry data from authors' previous work were in agreement with the aforementioned results implicating autophagy in trapped PARP removal. Further analysis showed that autophagy stimulation by Torin-1 causes resistance to talazoparid, whereas treatment with bafilomycin increased sensitivity. These results were further validated by genetically interfering with the autophagy process through genetic inhibition of ATG7 and syntaxin-17. Furthermore, the authors linked the potential cytoprotective effect of autophagy upon PARPi treatment to the effect on PARP trapping since autophagy did not show the same effect when veliparid is used. The latter inhibits PPAR catalytic activity without causing trapping. Moreover, PARP1 was observed to accumulate in lysosomes isolated from HeLa and CAL51 cells expressing the lysosomal transmembrane protein TMEM192-3HA under

trapping conditions. In cells transfected with a PARP1 construct tagged with mCherry and GFP, PARP was localized mostly in the lysosome under trapping conditions. This localization was abrogated upon depletion of either ATG7 or syntaxin-17, further supporting a role for autophagy in the processing of trapped PARP. Recruitment of PARP in the lysosome under trapping conditions appears to be mediated by p97. However, regulation of trapped PARP1 processing by autophagy was shown to be independent of RNF4-mediated or UFD-mediated ubiquitination of PARP1, indicating that the p97- RNF4-UFD pathway acts separately from autophagy for the processing of trapped PARP1. Since TEX264 is a co-factor of p97, the authors explored whether it is involved in the modulation of trapped PARP1 levels. Indeed, it was shown that depletion or knockout of TEX264 in three different human lines caused an accumulation of trapped PARP1, suggesting that this receptor acts as a modulator of trapped PARP1 levels. Previously identified TEX264 interactomes confirmed that PARP1 is among its interactors. In addition, it was shown that a mutation in TEX264, which has the characteristic of the selective autophagy receptor LIR domain, prevents PARP1 accumulation in the lysosome under trapping. This suggests that TEX264-deficient cells experience altered DDR under PARPi treatment. Similar to talazoparib, resistance to the milder PARPi olaparib, was abolished in RPE TP53-/-hTERT BRCA1-/- cells, upon depletion of TEX264 and ATG7.

Comments

My major concern is that the authors claim a prominent role for nucleophagy in mediating the removal of cytotoxic trapped PARP1, but do not provide sufficiently robust data to support this view. In fact, they provide evidence implicating the selective autophagy receptor TEX264 and the ATPase p97 in the processing of trapped PARP1 from chromatin and its delivery to the lysosome. Importantly however, the physical interaction of TEX264 with PARP1 has not been satisfactorily studied. As this is a critical part of the proposed mechanism for trapped PARP1 removal, emphasis should be placed on the identification of specific domains/motifs involved. In addition, other molecular players known to be involved in nucleophagy have not been studied in order to validate the main claim of the manuscript.

Answer 1: We appreciate the reviewer's concerns that: (A) the interaction of PARP1 and TEX264 has not been properly addressed, and (B) other molecular players known to be involved in nucleophagy should be studied. In relation to the former, we have shown a clear interaction between PARP1 and TEX264 on the chromatin that is increased under trapping conditions by both co-immunoprecipitation (Fig. 5A and B) and proximity ligation assay (Fig. 5F and G). To further clarify whether the interaction between TEX264 and PARP1 is direct, we expressed and purified PARP1 and TEX264 from the E. coli expression system and performed a pull-down experiment (Fig. S5B). The pull-down experiment clearly demonstrated that PARP1 and TEX264 physically interact. We also used mutants of TEX264 that contain only the N-terminal gyrase inhibitory-like domain (Gyr), or the C-terminal domain. This clearly showed that only the C-terminal region interacts (Fig. S5B). To further clarify where the interacting region is, we performed hydrogen-deuterium exchange mass spectrometry (HDX-MS), where the difference in deuterium uptake between TEX264 alone and TEX264 with PARP1 indicates a peptide interaction. This highlighted two key areas of interest in the C-terminal half of TEX264, an αhelix immediately following the Gyr domain and residues 272-313 (Fig. 5D, S5A). It was difficult to further investigate the specific function of each interacting region or identify exactly where they interact with TEX264 due to the lack of a crystal structure that clearly defines the structure of TEX264. To clarify the importance of these two regions, we generated TEX264 mutants with either the αhelix region mutated or the C-terminal end truncated from residue 272. By isolating GFP-PARP1 from the chromatin fraction in co-

immunoprecipitation experiments, we were able to show that the C-terminal region is essential for TEX264 binding to PARP1 (Fig. 5E), whilst the interaction persists independent of α-helix (Fig. S5D). This demonstrates that TEX264 binds directly to PARP1 through a motif between residues 272 and 313. This region is also essential for binding to LC3 and p97 (An et al., Mol. Cell, 2019; Chino et al., Mol. Cell, 2019; Fielden et al., Nat Commun, 2020; and Lascaux et al., Cell, 2024), as well as for DNA repair of TOP1cc foci (Lascaux et al., Cell, 2024) and trapped PARP-induced DNA damage (this manuscript, Suppl. Fig. 8A, B). Therefore, the direct PARP-interacting region on TEX264 has multiple binding functions, and further studies will be essential to delineate these details at the amino acid level. Nevertheless, our findings that PARP1 directly interacts with TEX264 suggest a direct role for the TEX264-PARP1 axis in trapped PARP repair by autophagy.

(B) To address other molecular players known to be involved in nucleophagy, we assessed the impact of impairing autophagy on PARP1 localisation to the lysosome. Depletion of ATG7 and Syntaxin-17 was shown to impair this and cause sensitivity to PARPi in our initial manuscript, so we also investigated ATG9A and Beclin-1. Beclin-1 acts as a scaffold protein in the PI3K complex active during autophagy initiation, whilst ATG9A is a lipid scramblase involved in the growth of autophagophores, and also found in close proximity to PARP1 in our mass spectrometry screen. Depletion of both these proteins impaired localisation of PARP1 to the lysosome, shown by the mCherry-GFP assay (Fig. 3E and F). In summary, four proteins involved in autophagy at different stages and in distinct pathways (ATG7, STX17, ATG9A and Beclin-1) were shown to be involved in the processing of trapped PARP1 by autophagy using multiple different techniques. This, in addition to evidence shown in RNA-seq, CRISPR and mass spec screens, strongly supports that trapped PARP1 is processed by autophagy.

In addition, the authors show that removal of trapped PARP1 by the proposed mechanism depends on SUMOylation and ubiquitination, but is independent of the SUMO-dependent E3-ubiquitin ligase RNF4, which was previously found to regulate trapped PARP1 degradation by the proteasome. They should elaborate on this claim to provide experimental evidence for candidate E3 ligase(s) involved.

In view of the proposed importance of the TEX264-p97 axis in trapped PARP1 repair, the regulation of this mechanism should be investigated in detail.

Answer 2: We agree that the study of E3 ligases involved in this pathway deserves further exploration. However, we feel it is outside of the scope of this study and is something we plan to focus on in future research. This follows our communications with the editor, who stated the following: "We do not think that mechanistic insights addressing the regulation of the pathway would be needed at NCB". However, by individual depletion of p97 cofactors UFD1 (the p97-Ufd1 complex; previously shown to process trapped PARP by the proteasome) and TEX264 (p97-TEX264 complex for nucleophagy) and their co-depletion, we can now clearly show that are two separate p97-dependent degradation pathways (Ufd1/proteasome and TEX264/autophagy) for cellular response to trapped PARP1 (Figures 6A and 5A).

The finding that PARP1 is processed by autophagy is not new nor is the notion that inhibiting autophagy results in increased sensitivity of cells to PARPi treatment (for example, Bellare et al.,2021, Br J Cancer. 124(7): 1260–1274; Cahuzac et al., 2022, Commun Biol 5, 251; Elshazly et al., 2022). More importantly, the function of TEX264 as a nucleophagy receptor, in addition to its role in ERphagy, has been previously reported (Fielden et al., 2022, Autophagy 18, 40–49).

Answer 3: We agree that the notion that autophagy inhibition increases sensitivity to PARPi has been shown before, which is supportive of our model and acknowledged throughout our

manuscript. To our knowledge, our manuscript is the first study to demonstrate that autophagy directly processes trapped PARP1 and demonstrates the mechanistic insight into how the autophagy receptor TEX264 directly binds PARP and delivers it to the lysosome (Figure 5). A few other studies into the association of autophagy and PARPi have studied the mechanistic details behind this. Cahuzac *et al* found an indirect mechanism where changes to nuclear p62 levels affect HR through modulation of filamin A levels (Cahuzac, Langlois et al. 2022). We have ruled out this mechanism with the treatments and cell lines used, as we were unable to detect changes in p62 nuclear localisation. Moreover, in our case, the nuclear p62 seems even to be decreased in TEX264-knockout (ko) cells (Fig. S2E). For example, in Cahuzac et al., the inactivation of autophagy (ko-ATG16L1) leads to a significant efflux of p62 to the nucleus, and the lipidation of LC3 is severely reduced. Similarly, other studies show that increased nuclear p62 in autophagy-deficient conditions suppresses DNA repair by sequestering the E3 ubiquitin ligase RNF168, consequently abolishing the ubiquitination signal at sites of DNA damage for the recruitment of downstream DNA repair molecules, including 53BP1. Therefore, the knockout of the selective autophagy receptor TEX264 (ko-TEX264) in our manuscript: (i) does not affect the lipidation of LC3 and (ii) does not cause a significant efflux of nuclear p62. Despite this, DNA repair mechanisms are severely impaired in TEX264-ko cells under PARP-trapping conditions (Suppl. Fig. 7C- F), although 53BP1 recruitment remains unaffected (as it depends on the RNF168 E3 ubiquitin ligase activity at sites of DNA lesions being intact). These experiments clearly indicate that our discovered mechanism of selective autophagy of PARP1 (TEX264-dependent) is completely uncoupled from the previously described p62 mechanisms (Wang et al., Mol. Cell, 2016; Hewitt et al., Autophagy, 2016; Cahuzac et al., Commun. Biology, 2022).

Importantly, this mechanism (Cahuzac *et al.)* would also not explain our phenotypes of seeing PARP1 localisation to the lysosome or explain the role of TEX264, suggesting we have uncovered a new mechanism governed by the selective autophagy receptor, TEX264, which directly binds to PARP1. TEX264 has very recently been identified as a nucleophagy receptor (Lascaux, Hoslett et al. 2024), alongside a receptor of ER-phagy (An, Ordureau et al. 2019, Chino, Hatta et al. 2019), but this is the first paper to expand its role from only TOP1cc to other DNA lesions (trapped PARP1). To show how our findings fit with previous work on the association of PARPi and autophagy, we have contextualised our results at various points, with some examples shown below.

Introduction: "All four approved PARPi have been shown to upregulate autophagy, giving a cytoprotective effect, in a multitude of cell lines and patient-derived xenographs56-63, suggesting it as a good target for combination therapies."

Results: "The finding that PARPi sensitivity is heightened by autophagy impairment has been demonstrated before, and various explanations have been tendered for the importance of autophagy upregulation upon PARPi treatment. This includes PARPi-induced upregulation of PTEN to promote cytoprotective autophagy in response to PARPi-induced ROS62 and nuclear localisation of p62, indirectly causing upregulated homologous recombination87. In any of the conditions which induced either sensitivity or resistance to PARPi, we observed no effect on nuclear localisation of p62 (Fig S2E), implying an alternative role of autophagy."

Results: "The role of autophagy in cancer is complex, with different functions and effects depending on the type and stage of the disease55. There is equal confusion in the PARPi context, with conflicting roles of autophagy proposed57. Despite this, the evidence presented in our work suggests that autophagy is induced by PARPi treatment as a protective mechanism (Fig. 1), with most literature supporting this claim (56-63). Until now, autophagy has been proposed to be induced by PARPi and act indirectly to promote cell survival, in line

with other genotoxic agents72. PARPi-induced generation of reactive oxygen species (ROS) and upregulation of PTEN, a negative regulator of MTOR, enhanced autophagy, which acts to clear ROS, meaning its inhibition sensitised cells to Olaparib through ROS accumulation. Further, it was shown that enhanced autophagy promotes HR, leading to increased BRCA1 and RAD51 recruitment to sites of PARPi-induced DNA lesions87, 119. Whilst we describe no alternative mechanism for how PARPi induces autophagy, we observe a direct role of cytoprotective nucleophagy in processing trapped PARP1, as no effect is observed in non-trapping conditions, with veliparib (Fig. 1F, 2D) or trapping mutant PARP1del.p.119K120S (Fig. S3A and B). Of all PARPi, the most promising evidence for cytoprotective autophagy is associated with talazoparib57, the most potent trapper amongst clinically approved PARPi13, 15. Together, this strongly supports the discovery of a novel and direct role of nucleophagy in processing trapped PARP1."

In addition to ATG7, the authors should also test the effects of depleting other essential autophagy genes such as ATG16L1 etc. that are enriched under PARP1-trapping conditions.

Answer 4: Thank you for this suggestion. We focused on ATG9A, which is found in the mass spectrometry screen as a PARP1 interactor and found that its depletion caused hypersensitivity to PARPi (Fig S2C and D) and impaired localisation of PARP1 to the lysosome (Fig 3E and F). We also observed impaired localisation to the lysosome when Beclin-1 was depleted (Fig. 3E and F). Please also see our answer 1(B).

Did the authors check whether the nuclear localization of p62/SQSTM1 plays a role in their experimental setting as was previously shown for olaparib?

Answer 5: Please see answer 3 and (Fig S2E and S7B-F). Therefore, we do not consider the role of p62 in the here described pathway of selective autophagy of trapped PARP. We added the following discussion of this to the manuscript:

"The finding that PARPi sensitivity is heightened by autophagy impairment has been demonstrated before, and various explanations have been tendered for the importance of autophagy upregulation upon PARPi treatment. This includes PARPi-induced upregulation of PTEN to promote cytoprotective autophagy in response to PARPi-induced ROS and nuclear localisation of p62, indirectly causing upregulated homologous recombination. In any of the conditions which induce either sensitivity or resistance to PARPi, we observe no effect in nuclear localisation of p62 (Fig S2E), implying an alternative role of autophagy."

The arrangement of the panels in Figure 2 is somewhat confusing (e.g., Fig. 2D top, Fig 2C bottom and Fig 2F bottom, 2G top).

Answer 6: Thank you for pointing this out. We have rearranged the figures to avoid any further confusion.

Lines 116-119: Selective autophagy may or may not involve selective autophagy receptors/adaptor proteins. However, selective autophagy may also occur through the recognition of the LC3-interacting region (LIR) of autophagic cargo directly by LC3, without the implication of selective autophagy receptors. Please correct.

Answer 7: Thank you for this correction. We have corrected this in the updated manuscript, as shown below:

"Autophagy of specific cargo, termed selective autophagy, can occur through association of cargo with ATG8-family proteins conjugated to the growing autophagophore membrane, such as lipidated LC3. This can occur through direct interaction between LC3 and cargo, but often

requires SARs like TEX264, which recognise specific substrates and bridge them to ATG8-family proteins"

Lines 158-161: It has been shown that TP53INP1 can promote autophagy-dependent cell death and that DRAM1, PMAIP1, MYO6 and EI24 can act as positive regulators of autophagy. While all listed genes TP53INP1, DRAM1, PMAIP1, MYO6, EI24 regulate autophagy, particularly upon genotoxic stress, yet these proteins are not traditionally considered core autophagy proteins, i.e. essential components that directly participate in the formation of function of the autophagic machinery. This comment regards also some genes labelled as core autophagy in Figure 1C.

Answer 8: Whilst the terminology used was based upon the original gene signature categories published by Bordi *et al* (2021), we agree that it differs from the traditional classification of core autophagy proteins. We have edited the text to focus instead on the upregulation of SESN1, SESN2 and DRAM1 in the RNA-seq section, genes which are well-established to promote autophagy under genotoxic stress conditions, and on ATG16L and ATG9A in the mass spectrometry section. We have also simplified figures 1A and 1C to remove labelling which is not referred to directly in the text.

Line 251: The statement "PARP1 physically interacted with early-stage autophagy core machinery" needs to be toned down. The proximity labelling experiments indicate proximity of two proteins but whether they physically interact or not is not shown.

Answer 9: Thank you for pointing this out. We have changed the wording accordingly:

"Alongside many autophagy regulators, we identified ATG9A and ATG16L1, indicating the close proximity of trapped PARP1 with the core autophagy machinery."

Lines 252-255: Isolation of intact lysosomes and analysis of their contents (especially under Bafilomycin A1 treatment) cannot be an assay itself to explore whether trapped PARP1 could be cleared by autophagy. It has to be accompanied by and compared to the PARP1 abundance in the flow-through fraction. Bafilomycin A1 treatment should trap PARP1 in the autophagic compartments that cannot fuse with lysosomes and not necessarily into lysosomes as it is shown in Fig. 2B, C. In addition, control conditions without Bafilomycin A1 are necessary to show the relative abundance and organellar distribution of PARP1 under physiological conditions.

Answer 10: From published literature, it is unclear whether Bafilomycin A1 prevents autophagosome-lysosome fusion, which would reduce autophagy cargo levels in the lysosome (Mauvezin and Neufeld 2015), or lysosome acidification, which would cause an accumulation of autophagy cargo in the lysosome due to its inability to degrade the cargo (Mauvezin, Nagy et al. 2015). The latter is why bafilomycin was included to better allow detection of cargo in the lysosome, which may otherwise only be detectable transiently before their rapid degradation. As suggested, we performed a lysoIP assay with control conditions which omit bafilomycin and found that PARP1 levels in the lysosome are increased by Talazoparib + MMS alone but accumulate further when bafilomycin is added in combination (Fig. 2B). This is in accordance with impaired lysosome acidification, causing an accumulation of cargo in the lysosome. This assay also showed the relative abundance and organellar distribution of PARP1 under physiological conditions, with no PARP1 detected in the lysosome in untreated conditions. This is expected as literature suggests PARP1 is predominantly localised in the nucleus under physiological conditions (Vyas, Chesarone-Cataldo et al. 2013). Our mCherry-PARP1-GFP assay also serves as further validation that PARP1 localises to the lysosome. To further confirm that this is a result of processing by autophagy, we depleted the key autophagy factors ATG7, ATG9A and Beclin-1 and found that this impaired the formation

of green puncta in the cytosol (Fig. 2G, H, 3E and F). This serves as further evidence that these puncta arise as a result of PARP1 processing by autophagy.

In relation to comments that lysosomal PARP1 abundance should be compared to the flow-through fraction, we chose to compare it to the whole cell level instead, always including this as a control. This allowed us to confidently make conclusions about protein levels in the lysosome by observing stable levels in the whole cell extract. This is in accordance with the original lysoIP protocol described by Abu-Remaileh *et al (Abu-Remaileh, Wyant et al. 2017)*.

Immunoblotting detection of intact (full length) mCherry-PARP1-GFP fusion protein under the experimental conditions is needed. When tandem constructs are used in this context it is preferable to utilize live cell imaging rather than fixed cell imaging (Fig 2F, G), to avoid the risk of GFP fluorescence restoration upon increase of the lysosomal pH during fixation (10.1080/15548627.2020.1797280).

Answer 11: We have added an immunobot showing expression of the mCherry-PARP1-GFP construct at the expected approximate size of 167kDa with both PARP1 and GFP antibodies (Fig S3E).

Thank you for pointing out the concerns of using fixation in the tandem assay. This was something that was considered when optimising the fixation methods, and we chose to include a treatment + Bafilomycin control to show that both red-only and red + green puncta can be observed with this assay. We agree that live imaging is perhaps a more powerful tool for this assay, so repeated our live imaging with further optimisation to produce a higher quality video (Fig. 2I, Movies 1 and 2). The new assay shows a much clearer red puncta emerging from the nucleus after treatment, localising to lysosomes and being degraded. Using rendering, we were able to track the movement of distinct lysosomes more clearly and observe them engulfing the puncta that form after treatment.

Line 262: While the effort for assessing lysosomal localisation of PARP1KS mutant (7 biological replicates) is appreciated, the really low levels of PARP1KS (particularly for IP) make it difficult to confidently use it as a control for concluding that trapping is required for its degradation by autophagy. Instead, the authors should use veliparib on WT PARP1.

Answer 12: We agree that the $PARP1^{KS}$ alone isn't the strongest assay for showing that PARP1 processing by autophagy is trapping-dependent due to its low expression, so we repeated the lysoIP assay using veliparib, as suggested. Over four biological repeats, we found that lysosomal PARP1 levels were considerably lower in cells treated with veliparib than with talazoparib (Fig. 2D). This is in accordance with sensitivity and DDR marker assays using veliparib.

Line 295: The author showed inhibition of p97 by CB-5083 significantly reduced the accumulation of PARP1 in the lysosome. Is this reduction accompanied by an increase on PARP1 levels on chromatin (pre-extraction protocol)? The authors previously showed (ref 39) that expression of a p97 mutant increases PARP1 levels on chromatin but does the same happen with the inhibitor treatment?

Answer 13: In our previous work, we showed that CB-5083 (p97 inhibitor) caused delayed removal of trapped PARP1 from chromatin by both biochemical chromatin fractionation and WB and by PLA between PARP1 and yH2AX (Krastev, Li et al. 2022). We have added the following text to make this clearer:

"This is in accordance with the impaired removal of PARP1 from chromatin when p97 is inhibited, as observed previously [39]."

Figure 1E: What was the reason for selecting a different assay (resazurin) for assessing sensitivity genetically compared to the chemical treatments (Figs 1D & G)? If this was necessary for technical issues (please indicate why, perhaps in methods as it could be useful to others) related to depletion, could you also repeat in this set up the boosting of autophagy treatment as a control? Also, could the authors explain why a different range of talazoparib concentration was used compared to 1D & G?

Answer 14: As the reviewer suggested, resazurin was used as depletion often affected colony size during colony formation assays, making it difficult to accurately quantify. Resazurin also gave more consistent results, as depleted protein expression is better maintained for the 3-5 day time of the resazurin assay than for the longer time needed for colonies to grow. We have added the following statement in the methods section to make this clearer:

"Resazurin assays were more commonly used when experiments required depletion by RNAi, as this often affected colony formation. The shorter time course of resazurin assays was also more suited to depletion-based experiments, as low protein levels could be maintained more reliably than in longer colony formation assays."

We were unable to find a gene depletion that is routinely used to boost autophagy, hence why Torin-1 was used for this purpose. Whilst it is not the best method to boost autophagy due to its effect on other pathways, we confirmed that resistance caused by torin-1 is due to its effect of boosting autophagy by combining it with ATG7 depletion to negate this. ATG7 depletion was found to reverse the resistance to talazoparib caused by torin-1 (Fig. S2A)

Fig. 1D is treated with Talazoparib, whilst 1 1G is treated with veliparib. Far higher concentrations of veliparib are needed to cause sensitivity due to its lower potency, hence why veliparib is used in the µM range whilst Talazoparib is used in the nM range. The dosage used is within the ranges used in other literature.

Figure 1H: Given that experiments referring to Fig.1H precede the negative experiments using veliparid, I think it is essential for the immunofluorescence experiments to also include veliparid as a negative control, to validate the findings in Fig.1G.

Answer 15: As veliparib does not induce PARP1 trapping, no signal was observed during the preliminary stages of assay optimisation (data not shown), as only trapped PARP1 is seen due to the detergent pre-extraction in this assay. In our previously published work, we validated that only trapped PARP1 can be seen by observing no signal when using the non-trapping PARP1-KS mutant (Krastev, Li et al. 2022).

Fig. 2H and Movie 1: It seems there are two congregation events between mCherry-PARP1-GFP-positive structures and Lysoview 680-stained lysosomes in the movie. Do these structures show GFP quenching after the presumptive lysosomal engagement? Could they represent some random proximity events between the two organelles? It is not clear how authors concluded that the above "further confirms that trapped PARP1 localizes to the lysosome" (lines 277-278).

Answer 16: We agree that this movie/figure is confusing and provides weak evidence for our conclusion. We repeated live imaging of more cells and further optimised the treatment time and imaging conditions. We were able to obtain a much better movie and snapshots, which show red puncta emerging from the nucleus within ~5 minutes of treatment. These puncta localised with lysosomes for a period of time before their signal weakened and they disappeared, indicating degradation. Lysosomes then dispersed from the region where the puncta had been localised. We observed this in multiple cells, as well as observing several puncta forming and being degraded across the 2-hour assay. The puncta appear

predominantly red when localised to lysosomes (it is especially visible in the image frame at 9 minutes after image rendering; Fig. 2I; please note that the lysosome engulfed mCherry-PARP1/red puncta). Image rendering allowed us to better track distinct lysosomes, helping to confirm that these were not random proximity events, and to distinguish some green signal from the background, showing that it is partially quenched when the lysosomes accumulate at the puncta. Alongside lysoIP and fixed mCherry-GFP assays, this further confirms our conclusion that PARP1 localises to the lysosome upon trapping treatment.

New figures (Fig. 2I, Movie 1 and 2) and the following text have been added:

"To further confirm the localisation of PARP1 to the lysosome after treatment, we visualised mCherry-GFP-tagged PARP1 in cells stained with a lysosome dye by live imaging (Fig 2I). We observed puncta emerging from the nucleus within ~5 minutes of talazoparib and MMS treatment (treatment added at 2:30), with multiple puncta appearing across the 2-hour assay (Movie 1). These puncta localised with lysosomes, for around 20 minutes until they had been degraded and lysosomes dispersed (Fig. 2I and Movie 1). Using rendering, we were able to better distinguish the green signal from background noise and visualise lysosomes with better resolution. Through this, we observed quenching of the GFP signal at around 15 minutes, once lysosomes are strongly localising with the puncta (Fig. 2I and Movie 2). Together, this further confirms that PARP1 localises to the lysosome under PARP trapping conditions."

Fig. 2I: It seems that the residual ATG7 during siATG7 is enough for to permit (ATG12 conjugation to ATG5 and) LC3 lipidation (last two lines in LC3 blot). Do these bands represent LC3II as the molecular weight and their accumulation upon Bafilomycin A1 treatment suggest? Since LC3 is detected to some extend in the lysosome fractions, it suggests that autophagosome-lysosome fusion takes place upon siATG7. Judging from the legend of Fig. 2D, PARP1 levels normalization is performed by dividing with HA signal (please specify in each legend or the methods section how each normalization takes place). Normalization towards LC3 signal is needed to show the correlation of PARP1 engagement to the lysosome with LC3 delivery to the lysosome.

Answer 17: **(**Original manuscript Fig 2I, now 3A). The purpose of this assay is to show that lipidated LC3, and therefore autophagy, is required for PARP1 to enter the lysosome. Whilst we agree that there are still residual ATG7 levels in siATG7 conditions, ATG7 depletion does still significantly reduce lipidated LC3 (the lower band) in the autophagolysosome. As the reviewer suggested, normalisation is performed by dividing by HA, as this is what is pulled down, so it accounts for any differences in the efficacy of the pull-down. We have added clarification of this in the methods section, as this was used throughout all lysoIP assays.

"Whole cell extract and lysoIP fractions were analysed by Western blotting, and quantification of band intensity was performed using ImageJ. PARP1 levels in the lysoIP fraction were normalised by dividing by the HA level in the lysoIP fraction. Each set of biological repeats was then divided by one condition to display the PARP1 level as a fold-change."

Normalisation to LC3 in this instance wasn't performed as the purpose of the ATG7 depletion was to inhibit autophagy by reducing LC3 lipidation. To further evidence that lysosomal accumulation of PARP1 is autophagy-dependent, we showed that PARP1 localisation to the lysosome is impaired by ATG7, ATG9A or Beclin-1 depletion, using the mCherry-GFP reporter assay. By using 3 different means of inhibiting autophagy at different stages of the pathway, we further evidenced that PARP1 localisation to the lysosome is autophagy-dependent, without relying on changes to LC3 (Figs. 2G, H and S3E).

Figure 4G shows almost no PARP1 in basal conditions in triple negative breast cancer cell line CAL51. Furthermore, PARP1 is almost absent in the cytoplasm in most images (in line

with its well-known localization in the nucleoplasm, nucleoli and micronucleus). Nevertheless, it is surprising that there is almost no PARP1 outside the nucleus in any experimental condition, not even in lysosomes. How do the authors explain its absent in basal conditions? Are there any proposed mechanisms for its translation upon different signals? And how do they explain their absence in lysosomes?

Answer 18: These images are under detergent pre-extraction conditions to visualise only trapped PARP1, so it is expected that only chromatin-bound PARP1 is seen. We have strengthened this explanation in the text to avoid any further confusion.

"PARP1 is only visualised on chromatin under trapping conditions, confirming the efficacy of this assay for detecting specifically trapped PARP1."

Minor points

Authors may want to keep a constant representation of y-axis numbering and cut points in Fig. 1A, C and Fig. S4A, B. Values sometimes appear/align in some cut points.

Answer 19: As there is continuity between the two ends of the cut points, the graph includes all data.

Lines 134- 135 and 501-506: How do TEX264 and p97 mediate the delivery of PARP1 to the lysosomes? How do these complexes pass through the nuclear membrane?

Answer 20: This is a really interesting question, which we wish to address in detail in the future. Lesions could either pass through the membrane through the nuclear pore or via local and transient rupture of the nuclear envelope, recently described to be induced by ATR-directed phosphorylation of lamin A/C (Joo, Black et al. 2023, Kovacs, Vallette et al. 2023). The latter was shown in our work on TOP1cc processing by autophagy, as treatment with TOP1 inhibitors induced local changes to the nuclear envelope and lamina structure. This included the formation of 'blister'-like structures where there is an abnormal enlargement of the space between the inner and outer nuclear membrane in close proximity to autophagosomes, and regions of disrupted lamin A/C. TOP1cc localisation to the lysosome was also found to be ATR-dependent and leptomycin B-independent, supporting that transport across the nuclear membrane happens through ATR-induced rupture (Lascaux, Hoslett et al. 2024). To explore whether a similar nuclear rupture is acting with trapped PARP1 processing, lysoIP was performed with an inhibitor of ATR (VE-822), which showed that PARP1 delivery to the lysosome was impaired (Fig. S6D and E). On the other hand, leptomycin B, an inhibitor of the nuclear pore, had no impact on lysosomal PARP1 levels (Fig. SB and C). We also observed accumulation of lamin-A/C in lysoIP fractions under trapping conditions (Fig. S6B). Together, this fits the hypothesis that trapped PARP1 exits the nucleus through ATR-induced local disruptions in lamin A/C architecture.

We believe that TEX264 and p97 support this process indirectly. We have no evidence that they are involved in nuclear membrane disruption directly, but they (i) prepare PARP1 for autophagosomal processing and (ii) bridge the unfolded cargo to the lysosome. TEX264 aids in p97 recruitment to trapped PARP1, which we have shown by reduced p97 level in PARP1 co-IP when TEX264 is depleted (Fig. 5A, 5C). p97 then unfolds PARP1 to clear it from chromatin. TEX264 acts as a receptor, interacting with trapped PARP1 and LC3 to bridge the cargo to the autophagosome for degradation. To understand the importance of PARP1 clearance by autophagy, we assessed levels of protein aggregates, as these are known to be cleared by autophagy (Ravikumar, Duden et al. 2002) and can be induced by p97 unfoldase activity (Kobayashi, Manno et al. 2007, Mukkavalli, Klickstein et al. 2021). We found that protein aggregates containing PARP1 accumulate under trapping conditions and accumulate

further if autophagy is inhibited by Bafilomycin treatment (Fig. 6C-E). Therefore, we believe that TEX264 directs PARP1 for clearance by autophagy after its unfolding by p97 to prevent cytotoxic high levels of protein aggregates from forming.

The authors need to explain in more detail in the figure legend or the figures how they normalize their data. For example, in Figure 1I and 2D it is not clear what the authors normalize against (Fig 1I) or normalized lysosomal PARP1 levels. Also, it is advisable for the authors to re-arrange the panels in the respective figures in order to appear in the logical order (from up to down and from left to right).

Answer 21: Thank you for raising this concern. Normalisation has been better explained in the figure legends. For the lysoIP quantification, we have added detail to the methods section explaining how normalisation has taken place (see below). Figures have also been arranged in a more logical order.

"Whole cell extract and lysoIP fractions were analysed by Western blotting, and quantification of band intensity was performed using ImageJ. PARP1 levels in the lysoIP fraction were normalised by dividing by the HA level in the lysoIP fraction. Each set of biological repeats was then divided by one condition to display the PARP1 level as a fold-change."

Fig. 6E is duplicated.

Answer 22: We have corrected this when reassembling the paper.

The panel order in some figures (1, 2 and 5) is confusing.

Answer 23: Thank you for pointing this out. Figures have been rearranged into a more logical order.

Nucleophagy is a selective type of autophagy. The term "selective nucleophagy" is not appropriate in the context used by the authors, as it implies that specific nuclear components are selected and targeted, which is not shown here.

Answer 24: We detected trapped PARP1, a specific DNA lesion, as a selective nuclear component to be degraded by autophagy. Our statement of 'nucleophagy' has also been encouraged by our recent finding of TEX264-orchestrated autophagy of TOP1ccs. Several news and views written by different authors, some of them the leaders in the autophagy field (e.g. Klionsky, Dikic), reported on our discovery of autophagy of TOP1ccs as "Nucleophagy"(Ji, Dai et al. 2025, Lei and Klionsky 2025, Tomaskovic, Prieto-Garcia et al. 2025). Therefore, we believe processing of trapped PARP1 (a specific type of DNA lesion) also belongs to nucleophagy **–** a selective autophagy (orchestrated by autophagy receptor TEX264) of nuclear material (trapped PARP1).

Reviewer #2 (Remarks to the Author):

PARP1 (Poly ADP-ribose polymerase) is an enzyme involved in DNA repair. When treated with some of its inhibitors (PARPi), PARP1 becomes trapped on DNA, and this trapped PARP1 causes cytotoxicity. Cells defective in homologous recombination are sensitive to PARPi, and therefore PARPi are particularly effective in treating cancers with BRCA1 or BRCA2 gene mutations. In this study, Hoslett et al. discovered that trapped PARP1 is transported to lysosomes via autophagy in a manner dependent on the AAA-ATPase p97 and TEX264, which was previously reported to serve as a receptor for autophagy of the ER. Importantly, impairing autophagic degradation of trapped PARP1 increased cell sensitivity to PARPi, suggesting that selective autophagy of trapped PARP1 could be a druggable target especially for cancer cells that have acquired resistance to PARPi. Thus, this study provides key findings for future

pharmaceutical applications, but the authors should address the following issues to strengthen the model on how trapped PARP is transported to lysosomes via autophagy.

Major comments:

1. While TEX264 was reported to be an ER-localized transmembrane protein involved in autophagic degradation of the ER, its nuclear membrane localization remains unclear. In the authors' model, TEX264 binds PARP1 in the nucleus and then interacts with autophagy proteins in the cytoplasm to its sequestration within autophagosomes. To validate this model, the authors should show that TEX264 resides in the inner nuclear membrane in addition to the ER. It is also important to show how this protein complexed with PARP1 is exposed to the cytoplasm. Does lysosomal transport of PARP1 depend on nuclear export through the nuclear pore?

Answer 25: Thank you for these questions. TEX264 inner nuclear membrane localisation has previously been shown indirectly by Fielden *et al* (2020). It was shown to localise at the replication fork through co-localisation with EdU, and was observed in the nucleus by immunofluorescence when the transmembrane LRR domain was truncated, indicating it resides in the inner nuclear membrane(Fielden, Wiseman et al. 2020). Endogenous TEX264 has also been visualised within the outer and inner nuclear membrane by electron microscopy with immunogold labelling (Kucińska, Fedry et al. 2023) (see figure 7H of reference). We additionally validated this by showing TEX264 resides in close proximity to lamin-GFP by proximity ligation assay (Fig. S6A), further confirming its localisation to the INM. A section of text and a new figure have been added:

"As part of its function as a SAR, TEX264 localises to the ER and both the inner and outer nuclear membranes, as has been shown with electron microscopy(Kucińska, Fedry et al. 2023). We confirmed this using PLA between lamin-GFP and TEX264-V5 (Fig. S6A)"

For details on PARP1 nuclear export, please see answer 20.

2. Is DNA transported to lysosomes along with PARP1?

Answer 26: To test this question, we performed lysoIP and assessed DNA levels in the lysoIP fraction by either pico green DNA quantification or on an agarose gel. With both methods, we were unable to detect any DNA above background level in the lysosome after PARPi treatment (data not shown). This is opposite to what we have shown in TEX624-orchestrated nucleophagy of TOP1-ccs, where nuclear DNA fragments/TOP1ccs regions were observed in the lysosome (Lascaux, Hoslett et al. 2024). However, as trapped PARP1 is not covalently bound to DNA, as in the case of TOP1ccs, we believe TEX264 removes trapped PARP from chromatin without DNA fragments.

3. To conclude that trapped PARP1 is transported to lysosomes via macroautophagy, the colocalization of PARP1 and LC3 in the cytoplasm and its increase in STX17-knockdown cells should be shown. In addition, the authors should examine lysosomal transport of PARP1 in cells depleted for not only ATG7 but also other core ATGs such as FIP200, because recent studies reported degradation of different cellular components including the outer nuclear membrane via microautophagy, which specifically depends on the ATG8 conjugation system among core ATGs.

Answer 27: We attempted to detect colocalization of PARP1 and LC3 by both immunofluorescence and PLA using antibodies against the endogenous protein or expression of exogenous tagged proteins. Despite these efforts, technical issues meant this assay was

not an effective tool. We were able to optimise the live imaging mCherry-PARP1-GFP reporter assay to better observe red puncta emerging from the nucleus and localising to a dense area of lysosomes. These puncta disappeared within 20 minutes, and the lysosomes dispersed (Fig. 2I). Whilst not showing direct co-localisation to LC3, this assay did allow us to visualise the co-localisation of PARP1 with lysosomes after PARPi trapping. To confirm that this co-localisation is macroautophagy dependent and to rule out microautophagy as the pathway involved in lysosomal transport of trapped PARP1, we inhibited a number of pathways distinct from the ATG8 conjugation system. Using the fixed mCherry-PARP1-GFP reporter assay, we observed that both ATG9A and Beclin-1 depletion impaired the PARPi-induced formation of red cytosolic puncta, with levels detected at the untreated level (Fig. 3E, F). This implies that lysosomal transport of PARP1 induced by PARP1 trapping is dependent on macroautophagy.

4. Fig. 1D: It is required to show that the effect of Torin is cancelled by ATG depletion.

Answer 28: We performed a resazurin assay in dox-inducible shATG7 cells with or without dox and with Torin-1 combined with Talazoparib. We found that ATG7 depletion partially reversed the talazoparib effect caused by torin-1 (Fig. S2A).

5. Fig. 2B: Controls for "Baf -, Tala +" and "ATG knockdown" should be added.

Answer 29: We repeated the lysoIP with the following conditions: untreated, Baf only, Talazoparib + MMS and Tala + MMS + Baf. As expected, we observed no detectable PARP1 in the lysosome in untreated conditions, some PARP1 with either treatment alone, but a considerable accumulation when trapping conditions and Baf are combined (Fig. 2B, C). We also detected PARP1 in the lysosome with Talazoparib + Baf (no MMS) (Fig. 2D, E), showing that even at lower levels of trapping (MMS causes a large accumulation of trapped PARP1), PARP1 is still transported to the lysosome. ATG7 depletion by RNAi also impaired the PARPi-induced localisation of PARP1 to the lysosome (Fig. 3A, B), confirming that this is autophagy-dependent.

6. Fig. 2F: The frequency of cells with cytoplasmic PAPR1 signals should be quantified. It is also necessary to check whether cytoplasmic PAPR1 signals disappear by ATG knockdown.

Answer 30: This is a good suggestion. We quantified the frequency of red cytoplasmic puncta in the fixed mCherry-PARP1-GFP reporter assay and found that trapping conditions result in ~40% of cells showing red puncta, increased from ~10-15% in untreated and treated+Baf conditions (Fig. 2G, H). We also repeated this assay with depletion of either ATG7, ATG9A or Beclin-1 to impair autophagy. With all 3 depletions, the PARPi-induced formation of red puncta was impaired to untreated levels (Fig. 2G-H, 3E-F), showing that the formation of cytosolic red puncta of PARP1 is autophagy-dependent.

7. Fig. 2H and Movie 1: These data do not clearly show that cytosolic structures positive for PARP1 finally fuse with lysosomes.

Answer 31: We agree that the original video and figure don't fully show that cytosolic PARP1 appears after treatment and fuses with lysosomes. We have re-optimised and repeated this experiment and have replaced the initial figure and video (Fig 2I, Movie 1 and 2). In this, you can see in several cells that after treatment, red puncta of PARP1 emerge from the nucleus and are co-localised with the LysoView stain. This occurs within ~5 minutes of treatment (it is especially visible in the image frame at 9 minutes after image rendering; Fig. 2I; please note that the lysosome engulfed mCherry-PARP1/red puncta), and within 20 minutes the red signal disappears and lysosomes disperse, indicating that PARP1 has been degraded. The presence of mCherry but not GFP signal and the visible localisation with LysoView 680 stain support

that PARP1 puncta are localising to the lysosome after treatment in these videos. Image rendering allowed us to better distinguish between distinct lysosomes to better observe their fusion with puncta.

8. Fig. 5A: How PARP1, p97, and TEX264 interact with each other should be clarified by depleting one of them and using mutants defective in the interactions. Does trapped PARP1 indeed interact with TEX264 directly?

Answer 32: To address details of how PARP1, p97, and TEX264 interact, co-IP assays were performed in cells expressing PARP1-GFP and depleted of either TEX264 or UFD1 as a positive control. Immunoprecipitation of PARP1-GFP from the chromatin fraction and blotting for p97 showed that p97 recruitment to PARP1 is reduced by TEX264 depletion (Fig. 5A- C). We also blotted for TEX264 and further confirmed our previous co-IP assay that showed TEX264 recruitment to PARP1 is induced by trapping conditions (Fig. 5A - C). As UFD1 has been previously shown to aid in p97 recruitment to trapped PARP1, it is unsurprising that p97 recruitment in TEX264-depleted cells is reduced rather than abolished, as there is likely redundancy between UFD1 and TEX264. We previously believed that UFD1 and TEX264 act in separate pathways, as UFD1 depletion doesn't affect PARP1 recruitment to the lysosome. We confirmed this with a resazurin assay, which showed that combined UFD1 and TEX264 loss has an additive effect on sensitivity to talazoparib (Fig. 6A). Therefore, we believe that during selective autophagy, TEX264 is responsible for recruiting p97 to trapped PARP1 to allow processing by selective autophagy. Therefore, the p97-TEX264 complex processes trapped PARP1 by autophagy/nucleophagy (aggregated forms of PARP1, Fig. 6C-E) and the p97-UFD1 complex processes trapped PARP1 by the proteasome (soluble form of trapped PARP).

We further confirmed the direct interaction of TEX264 and PARP1. This is addressed in Answer 1A.

9. Fig. 5B: How do the authors explain why lots of signals appear within the nucleus in the proximity ligation assay, even though TEX264 should be anchored to the inner nuclear membrane (if the authors' model is correct), and why these signals are much more than cytoplasmic PARP1 signals shown in Fig. 2F.

Answer 33: We agree that this figure seems somewhat inconsistent with the rest of our data, given that both the nuclear and cytosolic signals seem more dispersed and at higher levels than would be expected. We re-examined the PLA images across 4 biological repeats and found the majority of cells show more signal towards the nuclear periphery (although there is always some signal within the nucleus) and limited signal in the cytosol, which is more reflective of what we see with the mCherry-PARP1-GFP reporter assay. Therefore, we have replaced this figure with one that is more representative of the full data set (Fig. 5F).

10. Fig. 5D: Examining the co-isolation of TEX264 and p97 with lysosomes will provide further molecular insights into trapped PARP1 transport to lysosomes.

Answer 34: We probed the lysoIP fraction for p97 and TEX264 levels and found that both are present and unchanged by trapping conditions (Fig. 2B). This is expected as both proteins are involved in autophagy beyond the trapped PARP-related role; TEX264 as a receptor for ER-phagy (An, Ordureau et al. 2019, Chino, Hatta et al. 2019) and p97 at various stages including initiation through Beclin-1 stabilisation (Hill, Wrobel et al. 2021), during autophagosome maturation (Cayli, Sahin et al. 2020, Desdicioglu, Sahin et al. 2021) and in mitophagy (Mengus, Neutzner et al. 2022).

Minor comments:

11. Lines 158-159, "core autophagy machinery, namely TP53INP1, DRAM1, PMAIP1, MYO6, EI24": This description should be corrected because core autophagy machinery or core autophagy-related proteins are used to represent proteins required for autophagosome formation such as FIP200, ATG7, and ATG8.

Answer 35: Whilst the terminology used was based upon the original gene signature categories established by Bordi *et al* (2021), we agree that it differs from the usual classification of core autophagy proteins. We have removed this from the text and focused instead on the upregulation of SESN1, SESN2 and DRAM1, genes which are well-established to promote autophagy under genotoxic stress conditions.

12. Lines 188-190, "Loss of regulators of autophagy USP7, USP8, CUL3, MTOR and VCP are also implicated in cell sensitivity to PARPi (Fig 1B).": References for the relationship with autophagy should be added to each factor. In addition, MTOR is inappropriate in this context because it is a negative regulator of autophagy. The authors should check other factors in this regard as well.

Answer 36: We have added references for each autophagy regulator mentioned.

13. While sequestrating PARP1 within autophagosomes seems to be sufficient to decrease the cytotoxicity of trapped PARP1, not only ATG7 depletion but also bafilomycin treatment and STX17 depletion also increased cell sensitivity to talazoparib. How do the authors interpret these results?

Answer 37: We believe that delaying the clearance and degradation of trapped PARP1 leads to sensitivity through increased cellular stress, as although p97 can unfold PARP1 to clear it from chromatin, unfolded proteins can form aggregates (Kobayashi, Manno et al. 2007) (Mukkavalli, Klickstein et al. 2021). Clearance of aggregates is a key function of autophagy (Ravikumar, Duden et al. 2002). In the case of TOP1cc processing by autophagy, aggregates of TOP1 accumulate when cells are treated with TOP1 inhibitors and lysosomal degradation is impaired with Bafilomycin A1 treatment (Lascaux, Hoslett et al. 2024). We performed similar assays in cells exposed to PARPi and assessed the formation of aggregates by FACS and immunofluorescence using the proteostat dye, as well as their contents by biochemical purification and immunoblotting. We found that protein aggregates containing PARP1 accumulate under trapping conditions and accumulate further if autophagy is inhibited by Bafilomycin treatment (Fig. 6C-E). These aggregates also co-localised with LAMP1, a lysosome marker, indicating they are degraded by autophagy (Fig. 6D). Therefore, we believe that TEX264 directs PARP1 for clearance by autophagy after its unfolding by p97 to prevent cytotoxic levels of protein aggregates from forming. This is why STX17-KD, shATG7 or Bafilomycin causes heightened sensitivity to PARPi.

In summary, the entire autophagolysosome pathway is involved in the process of clearing trapped PARP (in support of our recent discovery of autophagy of Top1-cc, Lascaux et al., Cell, 2024). This is further illustrated in our live images (Fig. 2I, this manuscript) as well as in Videos S1 and S2 from Lascaux et al., Cell, 2024, showing that lysosomes actively approach and interact with the nuclear envelope following treatment of cells with DNA-damaging agents that cause either trapped PARP or Top1-ccs.

14. Lines 460-462, "TEX264 was previously described to mediate selective autophagy of the ER (reticulophagy) through association with the damaged ER membrane via its N-terminal leucine-rich region.": The authors may want to check whether these papers describe that TEX264 mediates degradation of "damaged" ER membranes.

Answer 38: Thank you for pointing this out. We have removed the word damaged to better reflect the role of TEX264 in ER-phagy.

"TEX264 was previously described to mediate selective autophagy of the ER (reticulophagy) through association with the damaged ER membrane via its N-terminal leucine-rich region[90, 92]"

Reviewer #3 (Remarks to the Author):

In their manuscript, Hostlett et al. describe a potentially novel mechanism that mediates the clearance of trapped PARP1 and contributes to PARPi resistance. Through data mining, they found that blocking or loss of autophagy synergizes with PARPi, specifically PARP trapping. Correspondingly, blocking lysosome acidification using Bafilomycin A1 or knocking down autophagy factors sensitizes cells to the PARPi Talazoparib, whereas the mTOR inhibitor Torin confers resistance.

Using various cell biology assays, the authors showed that trapped PARP1 accumulates in lysosomes in an autophagy-dependent manner, which also requires p97. However, in contrast to their previous findings, where E3 ligase RNF4 and UFD1 mediated the recruitment of p97 to trapped PARP1, they found that modulating RNF4 and UFD1 does not impact lysosomal accumulation of PARP1. This prompted them to seek an alternative mechanism linking trapped PARP1 to p97 and the lysosome.

Drawing an analogy between Top1cc and trapped PARP1, the authors tested whether TEX264 might mediate the recruitment of p97 to trapped PARP1. Based on their results, they proposed that TEX264 directly interacts with trapped PARP1 and, together with its partner protein p97, transports the trapped PARP1 to the autophagosomal resident protein LC3 and subsequently to the autophagosome. Furthermore, they found that interfering with the autophagy pathway can partially restore PARPi sensitivity in resistant clones.

Overall, the manuscript presents a wealth of data and offers a potentially intriguing twist on the mechanism of trapped PARP1 degradation. However, much of the evidence appears more correlative than indicative of specific interactions. Given that PARPi sensitivity is closely tied to cell proliferation, identifying the specific interaction surfaces and generating site-dependent mutants will be necessary to establish a clear cause-and-effect relationship and uncover the molecular link between trapped PARP1 and autophagy. The paper would benefit from providing more mechanistic insights/order into the processing of trapped PARP1 on DNA, relevant signaling pathways, and clear evidence of a direct interaction between PARP1 and TEX264 prior to lysosomal transfer.

Conceptual concerns:

1. Pleiotropic Effects of Reagents: Many of the reagents used in the study, including Bafilomycin A1, Torin, siATG, p97, and TEX264, have pleiotropic effects on cell proliferation and the clearance of other replication blockades, such as Top1cc, which the authors mention. Depletion of ATG7, a core autophagy factor, likely blocks lysosomal import of most proteins targeted for lysosomal degradation. As a result, it is not surprising that ATG7 depletion would also block PARP1 clearance, making the findings more correlative than specific.

Answer 39: We accept that many of our reagents/tools have pleiotropic roles, which is why we used multiple tools and TEX264 variants where possible to validate results as specifically as possible. As a control to show that Torin causes resistance to talazoparib by boosting

autophagy, we performed a resazurin assay in dox-inducible shATG7 cells and were able to show that the ATG7 depletion partially reverses the resistance to talazoparib caused by Torin (Fig. S2A). Depletion of ATG7 was used as a means of inhibiting autophagy genetically to show that PARP1 localisation to the lysosome is dependent on autophagy, but to further confirm this, we also assessed PARP1 transport to the lysosome after ATG9A and Beclin-1 depletion. Inhibition of autophagy by depletion of either of these proteins impaired the formation of cytosolic red puncta seen in the mCherry-PARP1-GFP reporter assay, further suggesting that PARP1 is transported to the lysosome via autophagy (Fig. 3E). Overall, the reagents and tools we have used are all common in the field, and our conclusions have been drawn based on multiple different assays carried out with a range of different reagents and tools. Most importantly, we demonstrated a physical interaction between PARP1 and TEX264 in vivo (PLA assay, Co-IP and pull-down) (Fig. 5) and with the purified proteins (Fig. S5A-D). This set of data strongly suggests that the role of TEX264 in the processing of PARP1 is direct and specific. In addition, (i) TEX264 brings p97 to chromatin when cells are exposed to trapped PARP1 and DNA damage (Fig. 5A-C), (ii) TEX264 inactivation only causes DNA lesions in the presence of PARP inhibitors (Fig. S7 and S8). Altogether, this strongly suggests the direct role of TEX264 in the processing of PARP1 and the prevention of DNA replication stress and cell survival.

In conclusion, our findings demonstrate a direct physical interaction between PARP1 and TEX264. In cell lines stably expressing either TEX264-wt or its variants that hinder the delivery of PARP1 to the lysosome, there is a significant impact on cell survival in response to talazoparib, as well as on the repair of DNA damage. This strongly suggests that TEX264 plays a crucial role in the autophagy of trapped PARP.

2. Tankyrase Inhibition and PARPi Specificity: Talazoparib, which was used in most experiments, also inhibits Tankyrase, a protein that interacts with ATG9A, an autophagy factor. In normal cells, Tankyrase levels are kept low through PARylation-dependent proteasomal degradation. Inhibiting Tankyrase activity—either through specific inhibitors or Talazoparib—causes Tankyrase accumulation. To exclude the possibility that the observed phenotypes after Talazoparib treatment are due to Tankyrase inhibition rather than specific inhibition of PARP1, the authors should repeat key experiments with other PARP inhibitors, such as Niraparib (a potent PARP1 trapper) or the PARP1-specific inhibitor Saruparib (AZD5305).

Answer 40: As suggested, we repeated lysoIP with niraparib and, in accordance with our talazoparib data, observed lysosomal accumulation of PARP1 by ~1.5-fold compared to Bafilomycin alone (Fig S3C-D). We also performed colony formation assays and observed heightened sensitivity to niraparib in TEX264$^{-/-}$ cells compared to WT cells, as was observed with talazoparib (Fig S4A).

3. Selective Nucleophagy and TEX264-PARP1 Interaction: The proposed model for removing trapped PARP1 via selective nucleophagy requires more details. Specifically, the model would be strengthened by identifying the interaction surface between TEX264 and trapped PARP1. Additionally, it is important to clarify whether the TEX264-PARP1 interaction is DNA-mediated (i.e., whether it is sensitive to DNase or Benzonase). In this context, Figure 5 suggests that the TEX264-PARP1 interaction can occur in the absence of PARPi, raising questions about whether TEX264 specifically removes trapped PARP1 for clearance.

Answer 41: The direct interaction of TEX264 and PARP1 has been further explored, as addressed in answer 1A. All our co-IP experiments are performed in the chromatin fraction, which has been treated with benzonase and in the presence of ethidium bromide to disrupt

protein-DNA interactions. Whilst TEX264 interaction with PARP1 is increased by Tala+MMS, it is interesting that it also interacts under untreated conditions. In previous MS experiments, PARP1 was found as an interactor of TEX264 under untreated conditions (An, Ordureau et al. 2019), so they may interact in the absence of damage. Our new co-IP data (Fig. 5A) better show the increased interaction of TEX264 with chromatin-bound PARP1 under trapping conditions.

Please also refer to our answer 1, where we provided a detailed explanation of what we have included in the manuscript to address the direct interaction between TEX264 and PARP1.

4. RNF4, UFD1, and TEX264 Pathway: The authors, in collaboration with Dr. Chris Lord, previously showed that RNF4 and UFD1 sense trapped PARP1, deposit Ub and SUMO, and recruit p97 to remove trapped PARP1. In this manuscript, they report that lysosomal enrichment of PARP1 depends on p97 but not on RNF4 or UFD1. They further state (lines 333-336): "Indeed, either depletion or knockout of TEX264 in three different human cell lines, including HeLa cervical carcinoma cell line and two triple-negative breast cancer cell lines, CAL51 and MDA-MB231, caused a considerable accumulation of trapped PARP1, to a similar extent as previously identified co-factor UFD1."
   It would be important to delete both UFD1 and TEX264, or RNF4 and TEX264, to determine whether this autophagy pathway is epistatic or additive to the previously identified Ub/SUMO-dependent p97 recruitment pathway. Furthermore, the authors should clarify whether TEX264 acts upstream of p97. If p97 is responsible for removing trapped PARP1 from chromatin for autophagy, why does blocking lysosome function after PARP1 is no longer trapped at chromatin (i.e., after p97 removal) impede DNA replication and repair?

Answer 42: Thank you for pointing out these important questions. To assess if the TEX264 and UFD1 functions are additive or epistatic, we performed sensitivity assays combining siUFD1 and TEX264$^{-/-}$. We observed an additive effect, with UFD1 depletion causing further increased sensitivity to talazoparib in TEX264$^{-/-}$ cells (Fig. 6A), suggesting the two proteins are acting in separate pathways.

To clarify if TEX264 acts upstream of p97, we depleted either TEX264 or UFD1 and performed co-IP of GFP-PARP1 from the chromatin fraction, immunoblotting for p97 to assess the recruitment of p97 to trapped PARP1. We observed that TEX264 depletion impaired recruitment of p97 to both trapped PARP1 and the chromatin fraction as a whole (Fig. 5A, C). This implies that TEX264 acts upstream of p97. However, we believe that TEX264 remains localised at the trapped PARP1 lesion after p97 recruitment and PARP1 unfolding. TEX264 then acts to bridge unfolded PARP1 to the autophagophore via its PARP1 interaction and LIR domain.

We understand the confusion surrounding why impaired autophagic processing of PARP1 is so damaging to DNA repair if it has already been cleared from chromatin. A major role of autophagy is in the clearance of protein aggregates, and protein unfolding by p97 is thought to contribute to aggregate formation. As our previous work found that TOP1cc can accumulate as cytotoxic aggregates when not processed by selective autophagy (Lascaux, Hoslett et al. 2024), we explored if the same is true for trapped PARP1. By FACS and immunofluorescence using the proteostat dye that stains protein aggregates, we found that aggregates accumulated in response to extended trapping conditions and that they localised to lysosomes (Fig. 6C, D), suggesting degradation by autophagy. By biochemical purification of aggregates, we detected the presence of PARP1 in aggregates, with this accumulating considerably when autophagy was impaired by bafilomycin treatment (Fig. 6E). Overall, this shows the

importance of selective autophagy downstream of PARP1 processing by p97 to prevent the accumulation of PARP1-containing protein aggregates. Protein aggregates are known to cause genomic instability (Ainslie, Huiting et al. 2021, Wen, He et al. 2023), with this likely to be the cause of the heightened DDR and replication stress markers we observed when TEX264 function or autophagy was impaired.

Additional Technical comments

5. PARP1del.p.119K120S Mutant: The PARP1del.p.119K120S mutant was introduced as a non-trapping variant of PARP1. It would be important to measure its trapping ability compared to wild-type (WT) PARP1 in the presence of different PARP inhibitors.

Answer 43: This mutant was originally developed as a result of a CRISPR-Cas9 screen searching for in-frame *PARP1* mutations that caused PARPi resistance. PARP1del.p.119K120S was identified, and further investigation showed that it exhibits impaired recruitment to chromatin due to its two residue deletions occurring in the DNA contacting region of the second ZnF domain (Pettitt, Krastev et al. 2018). In our previous work, we validated this, showing that the level of PARP1del.p.119K120S on chromatin is considerably lower than the WT and not increased by trapping conditions, shown both by chromatin fractionation and immunofluorescence with detergent pre-extraction (Krastev, Li et al. 2022).

6. Clarification of "Mildly Depleted Genes": At line 190, the authors refer to “mildly depleted genes” in the CRISPR screen of Figure 1B. Could they clarify the criteria used to define "mildly depleted genes"?

Answer 44: These genes were depleted less than the others, but still to a significant degree.

7. Potential Pleiotropic Effects of Torin1: In Fig. 1D, Torin1 might exhibit pleiotropic effects, particularly in blocking cell proliferation. It is well-documented that PARPi sensitivity depends on cell proliferation and DNA replication. Is there an alternative molecule that can activate autophagy without affecting cell growth?

Answer 45: We aren’t aware of an alternative strategy for boosting autophagy that is well established in the field, but we understand the concerns surrounding the pleiotropic roles of Torin-1. To address this, we performed sensitivity assays in dox-inducible shATG7 cells. We found that depletion of ATG7 partially reversed the talazoparib resistance caused by Torin-1, implying that resistance is caused, at least in part, by the function of Torin-1 in boosting autophagy (Fig. S2A).

8. Co-localization of PARP1 with Lysosome Tracker: In Fig. 2H, it appears that only a small fraction of PARP1 co-localizes with the lysosome tracker. Could the authors explain how frequently nucleophagy-induced PARP1 removal occurs and whether this effect is widespread?

Answer 46: We repeated the live imaging, optimising treatment time and imaging settings and were able to obtain much clearer videos. We observed red puncta emerging from the nucleus, localised to a dense area of lysosomes within ~5 minutes of treatment. Multiple puncta appeared throughout the 2-hour assay, and all localised clearly to lysosomes before disappearing as the lysosomes dispersed (Fig. 2I). We quantified the frequency of this event by fixed imaging and observed that ~40% of cells displayed red cytosolic puncta after talazoparib and MMS treatment, significantly higher than the 10-15% of cells where red puncta were observed in either untreated or bafilomycin treated conditions (Fig 2H).

9. PARP1 Expression in Input Group: In Fig. 2K, the expression level of PARP1 in the last two lanes of the input group appears slightly decreased. Have the authors investigated whether autophagy-mediated removal of PARP1 affects the total amount of PARP1 in cells as a result of degradation?

Answer 47: The level does appear slightly lower in this blot, but this may be due to it being a different monoclonal cell line that has slightly different PARP1 expression levels. Previously, we have looked into autophagy-mediated removal of PARP1 by cycloheximide chase with and without autophagy inhibition, but didn't observe an obvious change (data not shown). This may be due to the low sensitivity of WB, but it is likely due to the high abundance and long half-life of PARP1, with only a proportionally low amount being trapped on chromatin and degraded by autophagy.

10. Survival Experiments with PARP1 Mutant: While Figure 4 is convincing, could the survival experiment be performed in cells expressing a PARP1 mutant that impairs trapping? This would help determine if the effect is entirely PARP1-dependent, as TEX264 and p97 have multiple targets. Additionally, have the authors investigated whether TEX264-/- cells proliferate at the same rate as WT?

Answer 48: Thank you for this interesting suggestion. We performed sensitivity assays in HeLa cells expressing either PARP1-WT or PARP1-F44*, a mutant shown to induce PARPi resistance by impairing PARP1 trapping (Pettitt, Krastev et al. 2018). We found that TEX264 depletion only caused increased sensitivity to talazoparib in PARP1-WT and not PARP1-F44* cells (Fig. 4G). This further confirms that the sensitivity induced by TEX264 loss is entirely dependent on trapping.

We do not observe any difference in proliferation rate between WT and TEX264$^{-/-}$ cells.

11. Error in Fig. 6E: There appears to be an error in Fig. 6E, with overlapping duplicate diagrams.

Answer 49: We have corrected it in the updated figures.

12. Relationship of p97 and TEX264: To further confirm that p97 and TEX264 act in the same pathway, the authors could use a p97 inhibitor (p97i) in TEX264-/- cells and assess whether there is a cumulative effect.

Answer 50: We performed sensitivity assays in WT and TEX264$^{-/-}$ cells and found that p97i caused increased sensitivity to PARPi in TEX264$^{-/-}$ cells (Fig. 6B). Whilst we believe that TEX264 and p97 act in the same pathway, we also know from our previous work that another pathway of trapped PARP1 repair, mediated by UFD1, also involves p97. The additive effect we have observed is likely due to both these pathways being impaired by p97i. It is important to keep in mind that there are many p97-complexes/sub-complexes on chromatin, such as p97-Ufd1-Npl4 (Meerang, Ritz et al. 2011), p97-ATX3 (Singh, Oehler et al. 2019), p97-Ufd1 (Krastev, Li et al. 2022), and p97-TEX264 (Fielden, Wiseman et al. 2020, Lascaux, Hoslett et al. 2024). So, inhibiting p97, all these complexes are affected, whereas inactivating TEX264 only the p97-TEX264 complex is affected. In case of trapped PARP1, there are two p97 complexes: p97-UFD1 for proteasomal degradation of soluble trapped PARP1 (Krastev, Li et al. 2022) and the here described p97-TEX264 complex, very likely for the removal of aggregated PARP1 (Fig. 6).

13. Line 458-460 Clarification: The authors found that TEX264 is implicated in both TOP1cc and trapped PARP1, but this does not necessarily mean TEX264 is specialized in DNA repair pathways.

Answer 51: We believe TEX264 is directly involved in DNA repair as it binds to DNA lesions (TOP1-ccs and trapped PARP) and delivers these lesions to the lysosome. Moreover, TEX264 directly repaired TOP1ccs as we showed in Fielden *et al.* (2020), and also in Lascaux *et al.* (2024). The inactivation of TEX264 causes DNA damage due to a direct defect in TEX264's interaction with TOP1 or PARP1. This causes genomic instability as visualised with many phenotypes and consequently leads to a specific mutational signature in response to TOP1 inhibitors. We have adjusted this wording to avoid making any far-reaching conclusions. Important to mention, we found that TEX264 is a constitutive part of the replisome (please see Fig. 6A in Fielden et al. (2020) and Fig. 1A- C in Lascaux et al. (2024)), so we believe TEX264 is a DNA repair factor for a new type of DNA repair-nucleophagy.

"Therefore, we propose TEX264-mediated selective nucleophagy as a novel, specialised DNA repair pathway for selected chromatin-bound protein lesions, TOP1cc and trapped PARP1."

14. Microscopy Foci Quantification: For greater clarity, microscopy foci quantification could be presented as a violin plot, with the median exposed.

Answer 52: We have presented this data as Tukey box plots, which display the median, the box indicates the interquartile range, and whiskers indicate 1.5x interquartile range.

New References added in the manuscript:

Abu-Remaileh, M., G. A. Wyant, C. Kim, N. N. Laqtom, M. Abbasi, S. H. Chan, E. Freinkman and D. M. Sabatini (2017). "Lysosomal metabolomics reveals V-ATPase- and mTOR-dependent regulation of amino acid efflux from lysosomes." Science **358**(6364): 807-813.

Ainslie, A., W. Huiting, L. Barazzuol and S. Bergink (2021). "Genome instability and loss of protein homeostasis: converging paths to neurodegeneration?" Open Biol **11**(4): 200296.

An, H., A. Ordureau, J. A. Paulo, C. J. Shoemaker, V. Denic and J. W. Harper (2019). "TEX264 Is an Endoplasmic Reticulum-Resident ATG8-Interacting Protein Critical for ER Remodeling during Nutrient Stress." Mol Cell **74**(5): 891-908 e810.

Cahuzac, M., P. Langlois, B. Peant, H. Fleury, A. M. Mes-Masson and F. Saad (2022). "Pre-activation of autophagy impacts response to olaparib in prostate cancer cells." Commun Biol **5**(1): 251.

Cayli, S., C. Sahin, T. O. Sanci and H. Nakkas (2020). "Inhibition of p97/VCP function leads to defective autophagosome maturation, cell cycle arrest and apoptosis in mouse Sertoli cells." Theriogenology **158**: 196-206.

Chino, H., T. Hatta, T. Natsume and N. Mizushima (2019). "Intrinsically Disordered Protein TEX264 Mediates ER-phagy." Mol Cell **74**(5): 909-921 e906.

Desdicioglu, R., C. Sahin, F. Yavuz and S. Cayli (2021). "Disruption of p97/VCP induces autophagosome accumulation, cell cycle arrest and apoptosis in human choriocarcinoma cells." Mol Biol Rep **48**(3): 2163-2171.

Fielden, J., K. Wiseman, I. Torrecilla, S. Li, S. Hume, S. C. Chiang, A. Ruggiano, A. Narayan Singh, R. Freire, S. Hassanieh, E. Domingo, I. Vendrell, R. Fischer, B. M. Kessler, T. S. Maughan, S. F. El-Khamisy and K. Ramadan (2020). "TEX264 coordinates p97- and SPRTN-mediated resolution of topoisomerase 1-DNA adducts." Nat Commun **11**(1): 1274.

Hill, S. M., L. Wrobel, A. Ashkenazi, M. Fernandez-Estevez, K. Tan, R. W. Burli and D. C. Rubinsztein (2021). "VCP/p97 regulates Beclin-1-dependent autophagy initiation." Nat Chem Biol **17**(4): 448-455.

Ji, F., E. Dai, R. Kang, D. J. Klionsky, T. Liu, Y. Hu, D. Tang and K. Zhu (2025). "Mammalian nucleophagy: process and function." Autophagy: 1-17.

Joo, Y. K., E. M. Black, I. Trier, W. Haakma, L. Zou and L. Kabeche (2023). "ATR promotes clearance of damaged DNA and damaged cells by rupturing micronuclei." Mol Cell **83**(20): 3642-3658.e3644.

Kobayashi, T., A. Manno and A. Kakizuka (2007). "Involvement of valosin-containing protein (VCP)/p97 in the formation and clearance of abnormal protein aggregates." Genes Cells **12**(7): 889-901.
Kovacs, M. T., M. Vallette, P. Wiertsema, F. Dingli, D. Loew, G. P. F. Nader, M. Piel and R. Ceccaldi (2023). "DNA damage induces nuclear envelope rupture through ATR-mediated phosphorylation of lamin A/C." Mol Cell.
Krastev, D. B., S. Li, Y. Sun, A. J. Wicks, G. Hoslett, D. Weekes, L. M. Badder, E. G. Knight, R. Marlow, M. C. Pardo, L. Yu, T. T. Talele, J. Bartek, J. S. Choudhary, Y. Pommier, S. J. Pettitt, A. N. J. Tutt, K. Ramadan and C. J. Lord (2022). "The ubiquitin-dependent ATPase p97 removes cytotoxic trapped PARP1 from chromatin." Nat Cell Biol **24**(1): 62-73.
Kucińska, M. K., J. Fedry, C. Galli, D. Morone, A. Raimondi, T. Soldà, F. Förster and M. Molinari (2023). "TMX4-driven LINC complex disassembly and asymmetric autophagy of the nuclear envelope upon acute ER stress." Nature Communications **14**(1): 3497.
Lascaux, P., G. Hoslett, S. Tribble, C. Trugenberger, I. Antičević, C. Otten, I. Torrecilla, S. Koukouravas, Y. Zhao, H. Yang, F. Aljarbou, A. Ruggiano, W. Song, C. Peron, G. Deangeli, E. Domingo, J. Bancroft, L. Carrique, E. Johnson, I. Vendrell, R. Fischer, A. W. T. Ng, J. Ngeow, V. D'Angiolella, N. Raimundo, T. Maughan, M. Popović, I. Milosević and K. Ramadan (2024). "TEX264 drives selective autophagy of DNA lesions to promote DNA repair and cell survival." Cell **187**: 1-21.
Lei, Y. and D. J. Klionsky (2025). "Autophagy as a way to remove DNA lesions." Autophagy **21**(3): 497-499.
Mauvezin, C., P. Nagy, G. Juhász and T. P. Neufeld (2015). "Autophagosome-lysosome fusion is independent of V-ATPase-mediated acidification." Nat Commun **6**: 7007.
Mauvezin, C. and T. P. Neufeld (2015). "Bafilomycin A1 disrupts autophagic flux by inhibiting both V-ATPase-dependent acidification and Ca-P60A/SERCA-dependent autophagosome-lysosome fusion." Autophagy **11**(8): 1437-1438.
Meerang, M., D. Ritz, S. Paliwal, Z. Garajova, M. Bosshard, N. Mailand, P. Janscak, U. Hübscher, H. Meyer and K. Ramadan (2011). "The ubiquitin-selective segregase VCP/p97 orchestrates the response to DNA double-strand breaks." Nat Cell Biol **13**(11): 1376-1382.
Mengus, C., M. Neutzner, A. Bento, C. C. Bippes, C. Kohler, S. Decembrini, J. Häusel, C. Hemion, L. Sironi, S. Frank, H. P. N. Scholl and A. Neutzner (2022). "VCP/p97 cofactor UBXN1/SAKS1 regulates mitophagy by modulating MFN2 removal from mitochondria." Autophagy **18**(1): 171-190.
Mukkavalli, S., J. A. Klickstein, B. Ortiz, P. Juo and M. Raman (2021). "The p97-UBXN1 complex regulates aggresome formation." J Cell Sci **134**(7).
Pettitt, S. J., D. B. Krastev, I. Brandsma, A. Drean, F. Song, R. Aleksandrov, M. I. Harrell, M. Menon, R. Brough, J. Campbell, J. Frankum, M. Ranes, H. N. Pemberton, R. Rafiq, K. Fenwick, A. Swain, S. Guettler, J. M. Lee, E. M. Swisher, S. Stoynov, K. Yusa, A. Ashworth and C. J. Lord (2018). "Genome-wide and high-density CRISPR-Cas9 screens identify point mutations in PARP1 causing PARP inhibitor resistance." Nat Commun **9**(1): 1849.
Ravikumar, B., R. Duden and D. C. Rubinsztein (2002). "Aggregate-prone proteins with polyglutamine and polyalanine expansions are degraded by autophagy." Hum Mol Genet **11**(9): 1107-1117.
Singh, A. N., J. Oehler, I. Torrecilla, S. Kilgas, S. Li, B. Vaz, C. Guerillon, J. Fielden, E. Hernandez-Carralero, E. Cabrera, I. D. Tullis, M. Meerang, P. R. Barber, R. Freire, J. Parsons, B. Vojnovic, A. E. Kiltie, N. Mailand and K. Ramadan (2019). "The p97-Ataxin 3 complex regulates homeostasis of the DNA damage response E3 ubiquitin ligase RNF8." EMBO J **38**(21): e102361.
Tomaskovic, I., C. Prieto-Garcia and I. Dikic (2025). "Nucleophagy repairs toxic DNA lesions." Cell Res **35**(3): 163-164.
Vyas, S., M. Chesarone-Cataldo, T. Todorova, Y. H. Huang and P. Chang (2013). "A systematic analysis of the PARP protein family identifies new functions critical for cell physiology." Nat Commun **4**: 2240.
Wen, J. H., X. H. He, Z. S. Feng, D. Y. Li, J. X. Tang and H. F. Liu (2023). "Cellular Protein Aggregates: Formation, Biological Effects, and Ways of Elimination." Int J Mol Sci **24**(10).

First of all, we would like to thank the reviewers for carefully reading and commenting on our revised manuscript. We are pleased to see that Reviewer 3 is completely satisfied with our revision and that Reviewer 2 is mostly satisfied, aside from one technical concern. Reviewer 1 appears supportive of our initial revision and how we addressed their questions. However, they have raised some additional concerns that we can clarify through further experiments or in writing.

REV#1

In their revised manuscript, now entitled "Nucleophagy removes cytotoxic trapped PARP1", Hoslett and colleagues conducted several additional experiments to address the reviewers' concerns.

Specifically, they provided additional evidence of the physical interaction between PARP1 and TEX264 and identify a region at the C-terminal end of TEX264 that is essential for binding to PARP1, among other functions. Further, more detailed analysis will be necessary to identify the specific amino acids involved in this interaction. Moreover, the authors examined additional autophagy factors to further support the role of autophagy in processing trapped PARP1. In fact, they tested whether components of the core autophagy machinery, which also function in nucleophagy, were necessary. Proteins for which emerging evidence suggests a role in this selective type of autophagy such as ESCRT components involved in nuclear envelop remodeling, including the ATPase VPS4, which has already been implicated in cancer development, could also have been examined.

…The authors used Torin-1 to boost autophagy, noting that they were unable to identify a gene depletion approach that is routinely used for this purpose. An alternative genetic approach would be Rheb RNAi, given that Rheb is the primary direct activator of mTORC1. It is true, however, that Rheb RNAi is not without its own drawbacks.

Answer 1: Whilst the genetic approach of using Rheb RNAi as an alternative to Torin-1 treatment is interesting, we agree with the reviewer that it would have drawbacks due to its pleiotropic autophagy-independent roles, such as in protein synthesis and cell cycle progression. Possibly due to this, Rheb RNAi is not routinely used as a tool for boosting autophagy, whereas Torin-1 is routinely used for this purpose and has been mechanistically proven to robustly induce autophagy (Thoreen CC et al, JBC, 2009, PMID: 19150980, Liu et al. J Med Chem, 2010, PMID: 20860370; Monther Abu-Remaileh et al., Science, 2017, PMID: 29074583). In addition, there is a report of Rheb -independent activation of mTOR in mammary tumour initiation (PMID: 32348753), hence our decision to use this drug.

We showed that mTOR inhibition by Torin-1 promotes cell survival in the presence of trapped PARP1 and that this protective effect can be suppressed by removing the key macroautophagy factors, ATG7 (Extended Data Fig. S2A). As the use of Torin-1 has drawbacks (discussed by the reviewer), these results should be interpreted alongside our other sensitivity assays where autophagy was suppressed, both genetically through ATG7 and ATG9A depletion (Fig 1E and Extended Data Fig. 2C), and chemically with Bafilomycin (Fig 1D). In all three of these conditions, cells were hypersensitised to PARPi by suppression of autophagy. Therefore, we are confident

in our overall conclusion that autophagy has a cytoprotective effect in response to PARPi, with our sensitivity assay using Torin-1 merely one of multiple experiments that support this. However, we also discussed these caveats in our manuscript (please see below)

….Although the potential involvement of specific E3 ligases in removing trapped PARP1 has not been examined, I would agree that this is worth exploring in future studies.

Answer 2: Thank you for your understanding and for not insisting on identifying the specific E3 ligases at this stage. This work is currently underway in our group, and we hope to report on it in our follow-up publication.

…Regarding the question of how TEX264 and p97 complexed with PARP1 cross the nuclear membrane to deliver PARP1 to lysosomes, the authors discuss several possibilities but provide no real experimental evidence.

Answer 3: We improved our discussion based on recent developments in the field. We also believe this represents a distinct and complex research area that has only recently emerged and must be addressed systematically. We are actively working on this concept, and our collaborative manuscript on one aspect of this process (DNA lesion transport across the nuclear envelope) has just been accepted (***Aveleira et al., Nat. Commun., 2026; PMID: 41507158***). This work shows that DNA lesions are exported from the nucleus to the cytoplasm via an active transport mechanism orchestrated by dynamin GTPases. Thus, in our manuscript (Hoslett et al.), the process of DNA lesion export, including trapped PARP, resembles that observed in mammalian cells (Aveleira et al., Nat. Commun., 2026; PMID: 41507158) or in yeast, as recently reported in Nature Cell Biology (***Mannino et al., 2025; PMID: 39920277***). Please see also our new movies (Supplementary Movies 1 and 2), Supplementary Figure1 and Fig. 2I, J.

…A few additional points deserve careful consideration:
…In Fig. S2A, the authors show that knockdown of ATG7 partially rescues the increased cell viability of torin-treated cells on talazoparib and conclude that increased viability conferred by torin is due to autophagy. Considering that knockdown of ATG7 reduces cell viability on talazoparib (Fig 1E), the effects could be simply additive, and it is difficult to conclude that torin increases viability due to autophagy induction.

Answer 4: Please see also Answer 1. We added the following paragraphs to our manuscript:

Lanes; 222-227…As mTOR has multiple functions, we combined torin-1 treatment with depletion of ATG7, an E1-like enzyme involved in conjugating LC3 to the membrane during phagophore formation and expansion[50, 55]. This suggested that the PARPi resistance induced by torin-1 is ***very likely*** *due to increased autophagy flux, as resistance to talazoparib was partially but significantly reversed by ATG7 depletion (Extended Data Fig. 2A).*

Lanes; 234-237… While the reversal of torin-1-induced PARPi resistance by ATG7 depletion may be merely an additive effect, we have demonstrated PARPi hypersensitivity by autophagy inhibition using both genetic and chemical tools.

…The data presented in Fig S2E do not agree with the provided text, that p62 localization to the nucleus is unaffected by any of the treatments, as differences appear among different experimental conditions, at least by eye. Please provide a quantification and information of independent experimental repeats that support this conclusion.

Answer 5: We quantified p62 nuclear recruitment in four independent experiments and present these data, together with the corresponding statistical analysis below the representative Western blot results, **in Extended Data Fig. 2E**. We did not observe any significant differences in nuclear p62 levels under our experimental conditions, either in WT cells or TEX264-knockout cells. We therefore believe that the previously reported role of p62 in response to PARP inhibitors in prostate cancer cell lines is not involved in the TEX264-orchestrated nucleophagy of trapped PARP1 described in our manuscript.

It is worth mentioning that the original reason (*in the first version of our manuscript*) the reviewer requested p62 analysis was the previously published study on PARP inhibitor (PARPi) response and p62 by Cahuzac et al. (PMC8940895). Cahuzac et al. proposed that high levels of autophagy (rapamycin treatment) decrease nuclear p62, leading to increased nuclear FLNA and upregulation of the HR pathway. Conversely, when autophagy is inhibited (ATG16L knockout), nuclear p62 levels are maintained in response to trapped PARP (Olaparib), resulting in reduced HR and hypersensitivity to PARPi.

In addition to our experimental evidence (Extended Data Fig. 2E), which shows that the nuclear localisation/stability of p62 is not significantly affected under our experimental conditions, our original experiments shown in **Figure 6F and 6G** completely rule out the p62-autophagy-HR repair-based model proposed by Cahuzac et al. (PMC8940895). Specifically, TEX264 or ATG7 inactivation resensitizes PARP inhibitor-resistant BRCA1-/- cells (BRCA1-/-). BRCA1 is known to be a key enzyme in HR repair, and BRCA1-knockout cells are well known to be HR-deficient (**PMID: 10549283; PMID: 11239455**). Thus, our experiments of the role of autophagy/TEX264/ATG7 in PARP inhibitor response, as in Fig. 6F and 6G, directly exclude HR-pathway involvement and the proposed p62/HR repair model in prostate cancer cell lines, as described by Cahuzac et al. (PMC8940895).

Furthermore, p62/SQSTM1 has recently been shown to possess an autophagy-independent but proteasome-dependent nuclear function (PMID: 39418304; PMID: 41543905, both from the Ciechanover lab).

In summary, our results do not support a role for p62, and consequently HR repair, in the context of the PARP1-TEX264-autophagy axis. Thus, the mechanism of nucleophagy of trapped PARP1 that we describe here appears to be genuine and direct, and not a compensatory response to elevated HR pathway activity as previously reported by Cahuzac et al. (PMC8940895).

…In Fig. 2B PARP1 is not visible in the LysoIP sample of untreated cells (right panel second lane), while in Fig. 2C basal precipitated PARP1 levels are normalised to 1. Please provide an additional exposure setting, where PAPR1 is visible.

Answer 6: Thank you for this feedback. Here is a longer exposure image where the band is more clearly visible, although still faint as is expected in untreated conditions (see the red box). These gels are included in our raw results (Supplementary Figures; original and unprocessed westernblots).

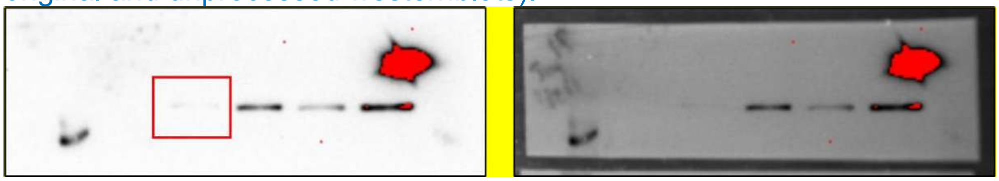

…To validate the novel mCherry-PARP1-GFP reporter, the idea is to detect whether there are additional to full length (possibly cleavage) products identified in the blot, as the one above the 100kD marker band (Fig. S3E, GFP). Whole PARP1, RFP and GFP blots are needed in control, trapping, Baf A1 and trapping/Baf A1 conditions.

Answer 7: Thank you very much for your further help in improving how we can demonstrate the validity of this powerful nucleophagy assay. To provide additional evidence that our reporter construct (mCherry-PARP1-GFP) is expressed and remains intact after different treatments, we analysed its expression as requested. We have included the full Western blot results, probed with specific antibodies against mCherry, PARP1, and GFP (Extended Data Figure 3E), as shown below.

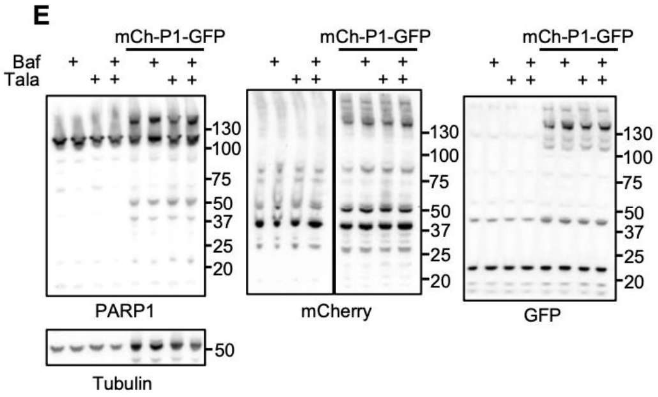

These experiments clearly demonstrate that the vast majority of the reporter remains intact (as indicated by a strong band above the 130 kDa marker) and expresses all three proteins (mCherry, PARP1, and GFP) as a single fusion product at the expected molecular weight. Most importantly, treatment with Talazoparib (Tala; trapping), Bafilomycin A1 (Baf), or their combination *(Tala and Baf) does not cause any disruption of the construct or its accelerated degradation.* Some degradation products of this construct are visible (a ~50 kDa band containing mCherry and PARP1), but they are minor and, importantly, are not further induced by the indicated treatments (Baf, Tala, or both). These results, together with our IF and live-cell imaging data (where this construct is almost completely localised in the nucleus), provide strong evidence that the mCherry-PARP1-GFP construct remains intact and is reliable for supporting the experimental conclusions presented in the manuscript.

The event of mCherry-PARP1 (red only) engulfment into the lysosome mentioned at the time frame of 9min (Fig. 2I) is followed by a clear and robust appearance or restoration of GFP fluorescence at least at the time frames of 12-13-18min

(surprisingly within the lysosome signal). What could this observation reflect? This result further illustrates the need for deeper validation of the novel mCherry-PARP1-GFP reporter assay.

Answer 8: As Rev #2 also raised concerns (see below, Answer 22) about our live imaging with this construct, we first validated the expression and stability of the construct (as suggested by Rev #1, see answer 7), and then performed a new set of experiments. We now provide new data in Figures 2I and 2J, as well as two new movies (Supplementary Movies 1 and 2) and Supplementary Figure 1. Please also see the corresponding figure legends and movie descriptions.

We believe that the new live imaging data, together with the construct validation, provide sufficient evidence to support the observed phenomenon: lysosomes approach the nucleus and extract PARP1, particularly after PARPi treatment. In the lysosome, only the red signal is present, as the GFP signal is quenched by the low lysosomal pH.

…The authors report that TEX264 plays a role both in recruiting p97 to trapped PARP1, facilitating unfolding and detachment from chromatin (before aggregation) as well as acting as an autophagy receptor to aggregated PARP1 at the nuclear membrane. It seems that PARP1-TEX264 interaction preexists PARP1 aggregation. While, a role of TEX264 in PARP1 autophagic degradation is supported, the TEX264 engagement to non-aggregated trapped PARP1 contradicts the notion of a p97-TEX264-nucleophagy axis, selective for degradation of aggregated PARP1 (summary point 4 of rebuttal).

Answer 9: Thnak you very much for being convinced about TEX264 in PARP1 autophagic degradation. This is the key finding that we want to communicate.

The most important finding regarding the aggregates is that only trapped PARP1 on DNA (both wild-type and in response to Talazoparib) forms aggregates (Fig. 6C-E). In contrast, the non-trapped form of PARP1, as clearly demonstrated by expression of the PARP1-KS variant that cannot bind DNA, does not form aggregates (Fig. 6E). This straightforward assay shows that the PARP1-DNA interaction is essential for aggregate formation.

Second, cells expressing a PARP1 variant that cannot bind DNA are completely resistant to Talazoparib treatment (the PARP inhibitor that causes a strong PARP trapping effect) (Fig. 4G). This further suggests that PARP1 trapping on DNA, and the consequent aggregate formation, is toxic to cells, and that TEX264 is essential for removing these trapped PARP1 lesions/toxic aggregates to the lysosome in a p97- and LC3-dependent manner (Fig. 5H-I). Inactivation of this mechanism causes cell lethality (Fig. 5J). However, this cell lethality is completely rescued/ not observed at all if PARP1 cannot be trapped on DNA (Fig. 4G).

The interaction between TEX264 and PARP1 in the absence of a PARP inhibitor (PARPi) was not surprising, as it had previously been observed in two unchallenged TEX264 interactomes (Fielden et al., PMID: 32152270; An et al., PMID: 31006537). Our in vitro data also suggest a strong physical interaction between TEX264 and

PARP1 (Fig. 5 and Suppl. Fig. 5). This resembles the scenario described for topoisomerase I (Fielden et al., PMID: 32152270), where TEX264 and topoisomerase I interact directly *in vitro*, but TEX264 orchestrates p97 recruitment to topoisomerase I upon treatment with a topoisomerase I inhibitor that induces covalent attachment of TOP1 to DNA (see, for example, Fig. 2d in PMID: 32152270). Despite this direct physical interaction between TOP1 and TEX264, p97 binding to TOP1 is almost completely lost in TEX264-knockout cells (Fig. 2c in PMID: 32152270). In that context, the interaction is enhanced by SUMOylation (Fig. 4d-g in PMID: 32152270), which increases upon covalent attachment of topoisomerase I to DNA and subsequently promotes its trafficking to the lysosome (Lascaux et al., PMID: 39265577). By analogy, a similar mechanism is likely for trapped PARP1, as chemical inhibition of SUMOylation abolishes delivery of trapped PARP1 to the lysosome (Fig. 3I). Moreover, trapped PARP1 is known to be SUMOylated (for example, PMCID: PMC2782092, PMCID: PMC3744807, PMCID: PMC2863168, PMCID: PMC8760077).

Although it remains unclear whether the TEX264-PARP1 interaction is required for a distinct function during unchallenged conditions, we did observe a modest increase in the interaction upon PARPi treatment (Fig. 5A-B). Importantly, we also observed reduced recruitment of p97 to chromatin and diminished interaction with trapped PARP1 upon TEX264 depletion, supporting a role for TEX264 in promoting p97 recruitment to chromatin (Fig. 5C). We consider it likely that additional factors, such as SUMOylation or ubiquitylation, contribute to this process, as these modifications were essential for the localisation of trapped PARP1 to the lysosome (Fig. 3I). A detailed investigation of the specific factors or ligases involved was beyond the scope of this study, but it is currently under intensive investigation in my lab.

...Previous and current findings propose that the C-terminal region of TEX264 is essential for both PARP1 and LC3 interaction, among other interactions. Are then TEX264-PARP1 and TEX264-LC3 interactions mutually exclusive? If the above aggregation is PARP1-dependent (not TEX264-dependent), a ternary LC3 (or an Atg8 family member)-TEX264-PARP1 complex cannot be formed and therefore TEX264 cannot serve as an autophagy receptor for PARP1. Does a C-terminal deletion mutant that does not include the LIR motif interact with PARP1 (Fig. 5E)? Does LC3 compete the PARP-1 interaction with either of these two TEX264 truncated constructs (with and without the LIR motif)? Similar concerns about mutual exclusive competition can be raised regarding the TEX264-p97 and TEX264-PARP1 interactions. Alternatively, does the C-terminal region of TEX264 constitute a loosely structured or contain an intrinsically disordered domain, prone to unspecific interactions at least in vitro?

Answer 10: Concerning the potential mutual exclusivity between the interaction of TEX264 with PARP1 and LC3, we do not believe such exclusivity exists, as PARP1-TEX264 PLA showed a PARP1-TEX264 interaction also outside of the nucleus (Fig. 5F-G). Because we observed PARP1 puncta rapidly fusing with lysosomes after emerging from the nucleus (Fig. 2I, J Supplementary Movies 1, 2 and Suppl. Fig. 1), a process that would depend on LC3 interaction with TEX264, it is unlikely that these interactions are mutually exclusive.

Moreover, two domains in the C-terminal region of TEX264, the LIR (which binds LC3) and the SHP (which binds p97), are essential for delivering trapped PARP1 to

lysosomes and for preventing trapped PARP1-induced DNA damage (Suppl. Fig. 8A, B) and consequently cell survival in response to PARPi/Talazoparib (Fig. 5H-J).

In summary, the interactions of TEX264 with LC3 and p97 are essential for the lysosomal delivery of trapped PARP1. Thus, TEX264 must simultaneously form a complex with LC3-lipidated phagophores, p97, and trapped PARP1 to deliver PARP1 to the lysosome. We hope to further explore this interaction in future work, so it is encouraging to us that the reviewer also appreciates its importance for understanding the mechanism in greater detail.

A similar mechanism was delineated for another nuclear protein, Topoisomerase 1 (Top1), when bound to DNA. Thus, the Tex264-Top1-p97-autophagy axis is strictly dependent on LC3 and p97, suggesting that the entire complex is essential for the delivery of trapped nuclear material (such as Top1 or PARP1) for nucleophagy (Lascaux et al., PMID: 39265577).

…Similar concerns about mutually exclusive competition can be raised regarding the TEX264-p97 and TEX264-PARP1 interactions.

Answer 11: TEX264 is a p97 adaptor protein/co-factor (Fielden et al., Nat. Commun., 2020; PMID: 32152270; PMID: 28451587), and PARP1 is a p97 substrate (Krastev et al., Nat. Cell Biol., 2022; PMID: 35013556). Based on our previous work and the interaction studies presented here (Fig. 5 and Suppl. Fig. 5), our work shows that TEX264 functions as a cofactor that recruits PARP1 (substrate) to p97, and this process is essential for the delivery to the lysosome in a LC3-dependent manner, including other atupgay factors too, ATG7, ATG92, BECLIN-1, STX17.

This is consistent with established concepts in the p97 field, in which adaptors/cofactors recognise and recruit p97 substrates - such as PARP1 - to p97 (PMID: 37611827; PMID: 36640759; PMID: 25146396; PMID: 22298039). In contrast, another well-characterised p97 cofactor, Ufd1, is not involved in this process of PARP1-TEX264-p97 autophagy (Suppl. Fig. 3H, I).

..Minor:
..Autophagophore membrane / autophagophore formation are not the standard terminology. Instead, the term "phagophore" is predominantly used to describe the early autophagic membrane structure, while usually "autophagosome formation" is used to describe the generation of autophagic vesicles.

Answer 12: We have revised the terminology throughout the manuscript. We now consistently use the term "phagophore" instead of "autophagophore membrane/formation."

..Please provide in text citations for lines 277-279.

Answer 13: We provided the reference in the text (PMID: 37304006)

..In line 264 the authors introduce veliparib as a PARPi that causes no PARP1 trapping and functions through catalytic inactivation of PARP1. However, in line 293 the authors state that veliparib is a weaker trapper of PARP1.

Answer 14: Thank you for pointing out this inconsistency. Because veliparib is a very weak trapper, it is generally not considered a PARP trapper. We have clarified the role of veliparib throughout the manuscript.

..In Fig. 3H the authors may want to change mean puncta per cell to average number of puncta per cell.
In Fig 3G correct HA label to HA (TMEM192).
The schematic in Fig S5C is illegible. Please increase font size. Please also include a label that these are TEX264 constructs
Fig 6 requires some alignment / rearrangement for clarity and adequate panel separation.

Answer 15: We have corrected these stylistic suggestions on our figures.

..Line 530: Provide the (ref)

Answer 16:We provided the ref.

..Fig. S6C: Please include the normalized PARP1 levels for the non-Tala+MMS condition as in Fig. S6D to show PARP1 lysosomal enrichment upon PARP1 trapping.

Answer 17: This quantification is based on three independent experiments for each condition shown. In the paper (now, Extended Data Fig. 6C), we only presented quantification for BAF + TM (bar 2) and BAF + TM + Leptomycin B (bar 4), as these are the only conditions relevant to this supplementary figure. The aim was to demonstrate that inactivation of nuclear pore complex activity (Leptomycin B/LeptB) does not affect the delivery of trapped PARP to lysosomes. The increase in lysosomal PARP1 between Baf and Baf + Tala + MMS can be seen throughout the manuscript in other lysoIP quantifications (Fig 2C, 2E, 3B, 3D, 3H, 3J, Extended Data Fig 3G, S3I, 6E).

In our recent collaborative manuscript, *Aveleira et al., Nat Commun, 2026,* PMID: 41507158, we also show that the delivery of DNA lesions outside the nucleus occurs via direct transport across the nuclear envelope with the help of Dynamins, the GTPases that mediate membrane vesicle fission, and not through the nuclear pore. A similar nuclear pore-independent process was reported for nucleophagy of Topoisomerase I (*Lascaux P, et al., Cell, 2024. PMID: 39265577*). Together, this further validates our finding that trapped PARP1 transport out of the nucleus is not dependent on the nuclear pore.

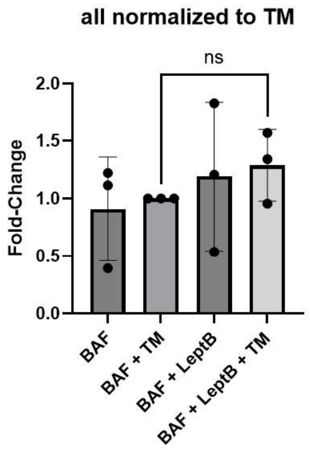

…The criticism of reviewer#1 concerned the term "selective nucleophagy", not nucleophagy per se, which is already a selective type of autophagy targeting nuclear material for degradation. In accordance with comment no.24, please make the necessary correction on page 18, line 582, "TEX264-driven selective nucleophagy of trapped…".

Answer 18: This has been corrected throughout the text. We now use only the term "nucleophagy."

…The concept of autophagy inhibition at the level of either ATG7, ATG9A or Beclin-1 to "evidence that PARP1 localization to the lysosome is autophagy dependent without relying on changes to LC3 (lipidation)" is problematic (last paragraph, last sentence). Shouldn't genetic inhibition of autophagy in these three different stages have some impact on the downstream LC3 lipidation? The authors also stated at the same time that the "purpose of ATG7 depletion was to inhibit autophagy by reducing LC3 lipidation" (same last paragraph, first sentence).

Answer 19: We apologise for any confusion here - this was a typo in the original rebuttal letter, not in the manuscript. Our figures clearly show that inactivation of ATG7, ATG9A, and Beclin-1 abolishes the delivery of PARP to lysosomes (Fig. 3A- B, 3E-F). While LC3 status was not discussed in the manuscript, it is clear from our western blots that inactivation of ATG7 (Fig. 3A) slightly reduces the lipidated forms of LC3 (band visible in the total cell extract; input), and that this lipidated LC3 is strongly diminished in lysosomes (Lyso-IP), as the reviewer expected.

…While lysosomal acidification may not be a prerequisite for autophagosome-lysosome fusion as V-ATPase-deficient lysosomes remain competent to fuse with autophagosomes and endosomes, Bafilomycin A1 (which is utilized here, Hoslett et al) still prevents autophagosome-lysosome fusion under V-ATPase-deficiency (Mauvezin, Nagy et al. 2015). Therefore, authors in the cited study (Mauvezin, Nagy et al. 2015) suggested that BafilomycinA1 inhibits fusion independent of its effect on lysosomal pH. Thus, to our current understanding, Bafilomycin A1 treatment possibly cannot facilitate the accumulation and detection of autophagic cargo in the lysosome in the described experimental setting here, and the respective findings may deserve reconsideration.

Answer 20: Our understanding is that the best-characterised role of Bafilomycin A1 is to block lysosomal acidification through V-ATPase inhibition; accordingly, in our manuscript, it is used to stabilise lysosomal contents in LysoIP experiments. However, we accept the reviewer's point that in the Mauvezin, Nagy et al. 2015 study, Bafilomycin A1 inhibits fusion by targeting SERCA and that this is independent of its role in acidification, as fusion still occurs when V-ATPase deficiency phenocopies Baf-induced impaired acidification. This study is performed in Drosophila, and it is unclear whether the same mechanism occurs in mammalian cells. Much of the understanding of lysosome-autophagosome fusion, and its relationship to lysosome acidification, originated in yeast studies of vacuolar fusion, where V-ATPases have been suggested to play a role. (Baars et al., 2007, PMID: 17652457; Strasser et al., 2011, PMID: 21934648). This further adds to confusion around the activity of Bafilomycin A1 in mammalian cells, where autophagy is known to differ. There may also be differences depending on dose and time of Bafilomycin treatment, which also differ between conflicting studies.

However, in the original manuscript that established the powerful Lyso-IP technique to detect cargos in intact lysosomes, Bafilomycin A1 was used to stabilise and thus visualise cargos in the lysosome (**Abu-Remaileh et al., Science, 2017, PMID: 29074583**). Similarly, we used the same approach in our recent publication describing Topoisomerase I - DNA damage lesions in the lysosome, and this effect is only pronounced when lysosomal acidification is inhibited by Bafilomycin A1 (**Lascaux et al., Cell, 2024, PMID: 39265577**). We do not believe it is essential to comprehensively address the mechanism of Bafilomycin within our manuscript. However, multiple results support that, at the dose and timepoints we used, Bafilomycin is primarily acting on lysosome acidification and not fusion. In Fig 2B, levels of lipidated LC3 in the lysosome were increased upon Bafilomycin treatment, supporting that it can enter the lysosome upon fusion with the autophagosome but is not degraded. In Fig 3C, syntaxin-17 depletion combined with Bafilomycin significantly reduced the level of lipidated LC3 in the lysosome compared to Bafilomycin alone. We conclude that syntaxin-17 depletion blocks fusion between autophagophore and lysosome (as reported: PMID: 23466629) and is visible in Fig 3. The entire process was abolished: delivery of trapped PARP1 to the autophagolysosome and the isolation of intact lysosomes in the amount visible in control conditions or in Bafilomycin alone. Also, this experiment supports the fact that Bafilomicin allows fusion of lysosome and autophagophore but not Syntaxin 17.

It is also important to interpret the lysoIP assays alongside the mCherry-GFP assays (Fig. 2G-J, 3E-F), which also demonstrated PARP1 localisation to the lysosome, shown by GFP quenching, in the absence of Bafilomycin.

With this combined evidence, we believe it is fair to conclude that PARP1 localisation to the lysosome does occur under PARP inhibitor-induced trapping conditions. This is the key message of our manuscript, and we demonstrated across the manuscript that this mechanism is important for cell survival in the presence of PARP inhibitors, and is also relevant for reversing PARP inhibitor-resistant BRCA1-deficient cells, thereby modulating the PARP inhibitor response.

REV#2
The authors have addressed most of the issues I raised in the review of the original manuscript.

Answer 21: Thnak you very much for acknowledging our revised version.

However, I still have a few remaining concerns.
1. Fig. 2I, Movies 1 and 2: The replaced images are still unconvincing and do not adequately support the authors' claim. Why do the mCherry-PARP1-GFP puncta appear in red from the nucleus, rather than as puncta positive for both green and red signals? It is also unclear whether the puncta originated from the nucleus. Although it is reasonable to interpret the loss of the PARP1 signal as reflecting its degradation, why does the lysosomal signal also disappear?

Answer 22: Please also see Answer 8 (to REV#1). Red mCherry puncta accumulate in lysosomes upon talazoparib treatment (with GFP quenched due to the low lysosomal pH), but this GFP quenching is clearly reversed by bafilomycin treatment (which inhibits lysosomal acidification) as visible In Fig. 2G, compare column 2 (Tala/MMS, no (−) Baf) with column 3 (Tala/MMS + Baf), and look for puncta outside the nuclei. This is explained in detail in the main manuscript text.

We therefore believe that our interpretation, that the red puncta originate from the nucleus, is supported by live imaging, including our newly acquired movies (Suppl. Movies 1 and 2) and associated images (Fig. 2I, J and Suppl. Fig. 1). Upon treatment, lysosomes move into proximity to the nuclear membrane, and puncta then appear and co-localise with lysosomes positioned directly adjacent to the nucleus.

Please note that these are 3D live images, and both cells and lysosomes are in motion. We therefore consider it more likely that the apparent loss of lysosomal signal upon PARP1 degradation is due to lysosomes moving out of the imaging plane. This is consistent with our model that lysosomes transiently "attack" the nuclear membrane at sites where PARP1 exits the nucleus following PARPi treatment.

2. Fig. 2H: In the middle graph, the "+" should be removed from the "TalA+MMS" row of the fourth bar.

Answer 22: Thank you for pointing this out. We corrected this typo

Reviewer #3 (Remarks to the Author):
The revised manuscript addressed all of my major concerns.

Answer 23: Thank you very much for acknowledging our revised version and for being convinced by our new set of experiments.

Point-by-point response to reviwers' comments on the 2nd Revision
NCB-A55402B (March 2026)

We would like to thank the reviewers again for their willingness to comment on our second revised manuscript, which was prepared in response to their suggestions. We are pleased to see that both remaining reviewers are now satisfied with the revised version. However, Reviewer 2 raised one remaining comment/suggestion regarding the inclusion of the possibility of microautophagy in the processing of trapped PARP1. We have now incorporated this possibility into the Discussion section of the manuscript.

Specifically;

Reviewer #1 (Remarks to the Author):
The authors have now adequately addressed my remaining concerns and I have no further comments.

Answer No 1: Thnak you very much.

Reviewer #2 (Remarks to the Author):
In the new figures and movies, the lysosome signal is retained after the disappearance of the red PARP1 signal, consistent with the notion that PARP1 is degraded within the lysosome. Meanwhile, again, an mCherry-positive but GFP-negative punctum emerges in the vicinity of the nucleus where a lysosome already awaits. This image appears to capture a microautophagy process, in which the lysosome directly engulfs PARP1 without its sequestration within an autophagosome, rather than macroautophagy the authors assume in this study. If this observation is reproducible and represents a major pathway for lysosomal degradation of PARP1, the authors should clearly discuss, based on this observation, the possibility that PARP1 degradation may be mediated by micronucleophagy, also given that there is no conclusive morphological evidence showing that PARP1 is sequestered within autophagosomes before being transported to lysosomes.

Answer No 2: Thank you for highlighting the possibility of microautophagy as a potential mechanism for the lysosomal processing of trapped PARP1. Based on our experimental data, we interpret PARP1 degradation to occur primarily through macroautophagy. Supporting this, TEX264 acts as a receptor mediating PARP1-lysosomal degradation in an LC3-dependent manner. TEX264 has been shown to function as a key receptor in ER-phagy, a form of macroautophagy (PMID: 31006538; PMID: 35417087; PMID: 31006537). Additionally, PARP1-lysosomal degradation relies on Syntaxin 17, a SNARE protein essential for autophagosome-lysosome fusion and closely linked to macroautophagic flux (PMID: 32264736; PMID: 35613317; PMID: 40479053). Although these molecular requirements align best with a macroautophagic mechanism, we agree that, without direct ultrastructural evidence of a double-membrane surrounding the PARP1 substrate, a clear distinction between macroautophagy and microautophagy remains open to interpretation. Therefore, we have revised the Discussion to acknowledge that, although our findings support the selective autophagy of trapped PARP1 (nucleophagy as a component of macroautophagy), we cannot entirely exclude the possibility that, under certain conditions, lysosomal processing of trapped PARP1 might also occur via microautophagy, potentially without phagophore formation.

We wrote: … Alternatively, the autophagy process we identified could represent microautophagy [123], in which lysosomes directly engulf PARP1 without prior sequestration in an autophagosome. Visualising the phagophore by electron microscopy surrounding trapped PARP1 would clarify whether trapped PARP1 is processed by the selective autophagy (nucleophagy) pathway we propose or by microautophagy.
